# Synthesis and Application of PN-Supported Mn(I) Carbonyl Alkyl Complexes

**DOI:** 10.1021/acs.organomet.5c00095

**Published:** 2025-04-30

**Authors:** Claudia Rabijasz, Stefan Weber, Berthold Stöger, Karl Kirchner

**Affiliations:** † Institute of Applied Synthetic Chemistry, TU Wien, Getreidemarkt 9/163-AC, A-1060 Wien, Austria; ‡ X-Ray Center, TU Wien, Getreidemarkt 9, A-1060 Vienna, Austria

## Abstract

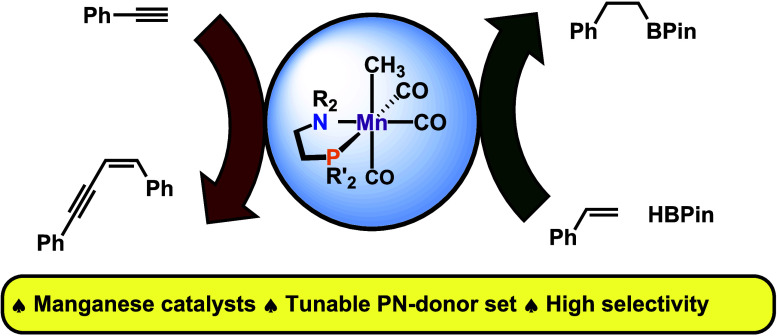

This work comprises the synthesis and characterization of aminophosphine (PN)-derived Mn(I) carbonyl complexes and the preliminary investigation of their alkylated congeners for catalytic applications. The complexes *fac*-[Mn(PN)(CO)_3_Br] are obtained from the reaction of Mn(CO)_5_Br with the bidentate ligand PN = R_2_N(CH_2_)_2_PR′_2_, where R = Me, Et, and pyrrolidine and R′ = Ph, *i*Pr, and Cy. Treatment of *fac*-[Mn(PN)(CO)_3_Br] with AgOTf yields *fac*-[Mn(PN)(CO)_3_OTf]. Upon reaction of *fac*-[Mn(PN)(CO)_3_OTf] with MeLi (R′ = alkyl) or MeMgCl (R′ = aryl), *fac*-[Mn(PN)(CO)_3_Me] is formed. *fac*-[Mn(P^Cy^N^Me^)(CO)_3_Me] and *fac*-[Mn(P^Ph^N^Me^)(CO)_3_Me] are identified as the best catalysts for the dimerization of phenylacetylene and the hydroboration of 4-chlorostyrene, respectively.

## Introduction

**Scheme 1:**
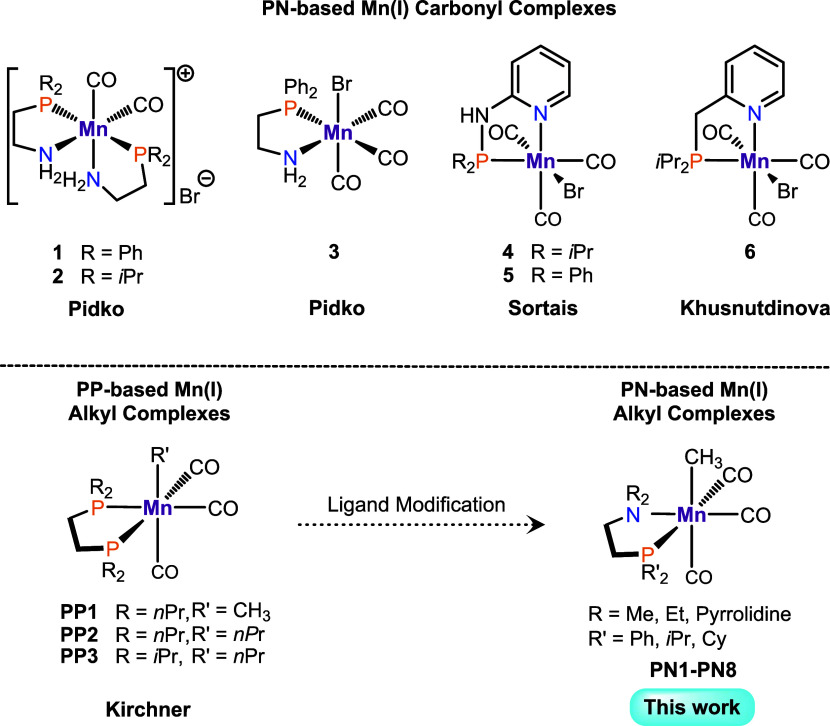
Representative Mn(I) Complexes Supported by PN Ligands

Organometallic catalysis plays an important role in industrial processes and promotes ongoing improvements in them.^1−4^ Driven by sustainability, replacement of noble metals by more abundant and less expensive counterparts attracted significant attention in recent years.^5^ Manganese, as the third most abundant transition metal in the Earth’s crust, emerged as a formidable player in homogeneous catalysis. Although pincer ligands are predominant in Mn(I)-catalyzed reactions,^6−9^ bidentate-based catalysts are encountered in several hydrogenation and hydrofunctionalization reactions. Selected examples for aminophosphine-based (PN) systems are depicted in Scheme 1 (**1–6**, top).^10−12^ All of the complexes described above were shown to operate via metal–ligand cooperation (MLC). In contrast, our group reported the bisphosphine (PP)-based Mn(I) complex *fac*-[Mn(*n*Pr_2_PCH_2_CH_2_P*n*Pr_2_)(CO)_3_Br] being capable of reduction of nitriles and ketones.^13^ By exchanging the bromide ligand with a methyl ligand to give the congener **PP1** (Scheme 1, bottom), no additives were required for the hydrogenation of nitiriles.^14^ Moreover, the modified complexes **PP2** and **PP3** (Scheme 1, bottom) exhibited good to excellent yields in various hydrogenation reactions.^15,16^ Other transformations, e.g., dimerization of alkynes,^17^ dehydrogenative silylation, and hydroboration of terminal alkenes were also catalyzed by **PP3**.^18,19^ Since MLC is not possible in these systems, the reactivity of these complexes is based on inner-sphere pathways. These can be enabled by migratory insertion of the alkyl group into the adjacent CO ligand, followed by protonation of the formed acyl and dissociation of the aldehyde.^20−23^

However, the ligand design proved to be crucial to achieving high reactivity of the active species. We attribute this to the migratory aptitude of the alkyl group since this step was found to be rate-limiting in all studied transformations. To extend this concept, we were interested in a mixed donor set to facilitate migratory insertion due to a different donor *trans* to the carbonyl ligands. Thus, we decided to investigate PN-based systems. The combination of a hard nitrogen and a soft phosphorus donor is a distinctive feature of PN ligands. Furthermore, this can enable the dissociation of the nitrogen donor, thus generating a vacant coordination site for substrate binding.^24^ While PN-based systems were proven to enhance catalytic activity in various transformations,^25−29^ investigations on PN-based manganese reactions are scarce.^30,31^

Herein, we report the synthesis and characterization of a series of PN-supported Mn(I) tricarbonyl triflate complexes as precursors for the corresponding methyl complexes. These complexes were obtained by ligation of aminophosphines with [Mn(CO)_5_Br], followed by treatment with AgOTf. A procedure for alkylation was developed to give the methyl complexes. In order to gain insight into the catalytic activity of these alkyl complexes, preliminary investigations were conducted.

## Results and Discussion

### Synthesis and Characterization

**Scheme 2:**
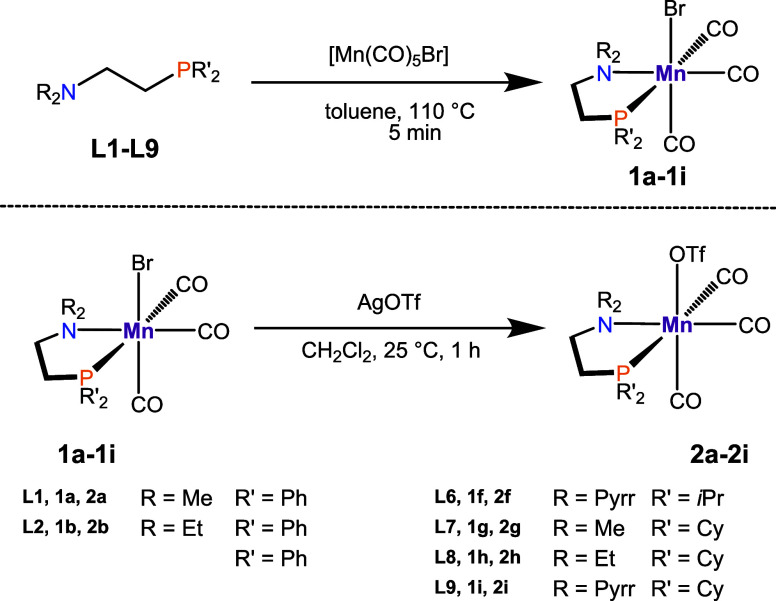
Complexation of PN Ligands with [Mn(CO)_5_Br] (Top) and Synthesis of Triflate Complexes **2** (Bottom)

Upon heating PN ligands with [Mn(CO)_5_Br], **1a**–**1i** could be synthesized in a short reaction time (Scheme 2, top). All bromide species were obtained as yellow or orange powders in a 23–91% isolated yield.

Initial attempts of alkylation of complex **1** species were carried out through reduction with Na sand, similar to the successful syntheses of PP-related systems,^14^ followed by the addition of MeI or 1-bromopropane. In the case of MeI, undesired tetracarbonyl side products were formed, whereby the formation of a hydride species when utilizing 1-bromopropane was observed. Next, the treatment of complex **1** with carbon-based nucleophiles was investigated. However, neither direct alkylation utilizing MeLi nor MeMgCl was successful.^32,33^ The reaction of **1** with AgBF_4_ followed by the subsequent addition of ZnMe_2_ or ZnEt_2_ failed as well. Thus, the method was adopted by utilizing AgOTf.^34,35^ The transformation was achieved by mixing a solution of complex **1** in CH_2_Cl_2_ with AgOTf (1.50 equiv) (Scheme 2, bottom). The triflate congeners **2a**–**2i** were obtained as yellow or orange powders, yielding 61–91%. Notably, **1** and **2** are air-stable compounds, but they are moderately light-sensitive. Regarding the ^31^P{^1^H} NMR spectra, all triflate complexes are slightly shifted downfield compared to their bromide analogues. The infrared (IR) spectra of complexes **1** and **2** display three distinctive CO signals in the carbonyl range of 2031–1874 cm^–1^, indicating a *fac* arrangement within an octahedral geometry.

**Scheme 3:**
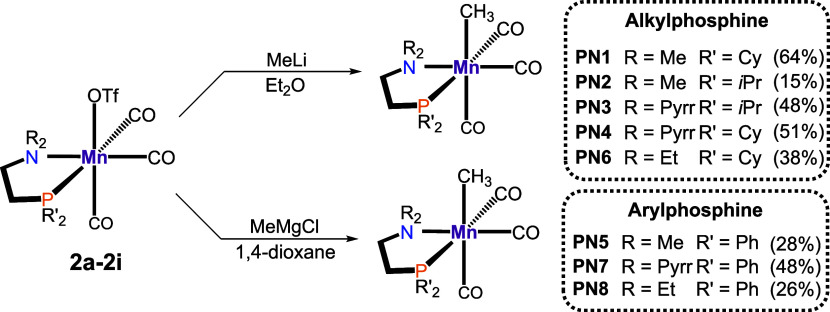
Synthesis of Methyl Complexes **PN1**–**PN8** via Lithium or Grignard Reagents

The alkylation of **2** utilizing ZnMe_2_ or ZnEt_2_ resulted in undesired byproducts, similar to the alkylation of the bromide congeners. Treatment of **2** with *n*-BuLi was even less successful; in fact, a coordinated butyl group has never been observed. However, the alkylation of **2** was possible following two different procedures (Scheme 3). For complexes containing alkyl-substituted phosphines, methylation was achieved upon treatment with MeLi at −78 °C. In the case of aryl-substituted phosphines, the alkylation was carried with MeMgCl in the presence of 1,4-dioxane. These two procedures allowed the isolation of **PN1**–**PN8** as yellow powders in moderate yields.

The alkylated Mn(I) carbonyl compounds (**PN1**–**PN8**) were identified by ^1^H, ^31^P{^1^H}, and ^13^C{^1^H} NMR and IR spectroscopy as well as HR-MS (see Supporting Information). Significant downfield-shifted ^31^P{^1^H} NMR signals compared to the triflate species were observed. The characteristic methyl group, being bonded to the manganese center, exhibits a doublet ranging between 0.03 and −0.79 ppm in the ^1^H NMR spectra. The ^13^C{^1^H} NMR resonances of alkylphosphine-based compounds appear at ca. −4 ppm and those of phenylphosphine-based compounds at ca. −1 ppm. As a result of the stronger π-back-donation, the IR signals suggest the strongest Mn–CO bond for the methylated compounds, evidenced by the notably lower wavenumbers observed for **PN**, in contrast to **1** and **2**.

Gratifyingly, we could also apply the introduced procedure for the synthesis of the bisphosphine-based complex *fac*-[Mn(P^Cy^P^Cy^)(CO)_3_Me] (**PP4**). This represents a more convenient protocol rather than reduction by Na/K followed by addition of MeI as reported for the synthesis of the similar complex **PP1**.^14^

### Structure and Bonding

**Figure 1:**
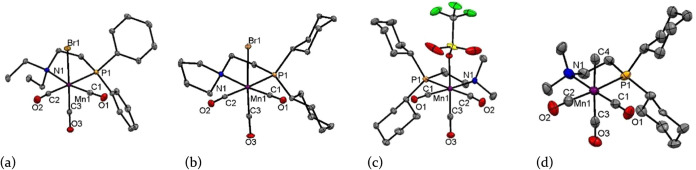
Structural views of (a) *fac*-[Mn(P^Ph^N^Et^)(CO)_3_Br] (**1b**), (b) *fac*-[Mn(P^Cy^N^Pyr^)(CO)_3_Br] (**1i**), (c) *fac*-[Mn(P^Cy^N^Me^)(CO)_3_OTf] (**2g**), and (d) *fac*-[Mn(P^Cy^N^Me^)(CO)_3_Me] (**PN1**) showing 50% ellipsoids (H atoms are omitted for clarity; for selected bond lengths (Å) and bond angles (deg), see the Supporting Information).

X-ray analysis of **1b, 1i**, and **2g** verified a slightly distorted octahedral geometry with *fac*-arranged CO ligands. The comparison of **1a**(36) and **1b** [Figure 1, (a)] highlights an interesting aspect; the Mn1–N1 distance is notably shorter in **1a** (2.204 Å) compared to **1b** (2.239 Å), while the Mn1–P1 distances are very similar (**1a**: 2.321 Å, **1b**: 2.323 Å).

Taking into account the related complex *fac*-[Mn(P^Ph^N^H^)(CO)_3_Br] as reported by Pidko et al.,^10^ one can assert that the bond distance between manganese and nitrogen increases in the order −NH_2_, −NMe_2_, and −NEt_2_. In this context, it is worth noting that the Mn1–N1 bond length in complex **1i** [Figure 1, (b)], featuring a pyrrolidine scaffold, is 2.186 Å. This falls within the range of bond distances between *fac*-[Mn(P^Ph^N^H^)(CO)_3_Br] and *fac*-[Mn(P^Ph^N^Me^)(CO)_3_Br] (**1a)**. Fortunately, we were able to confirm the presence of one triflate species **2g** using X-ray diffraction [Figure 1, (c)]. It provides a slightly distorted octahedral geometry with bond angles of 176.76(6)° (P1–Mn1–C2), 178.33(6)° (O4–Mn1–C3), and 176.12(6)° (N1–Mn1–C1). The smallest bite angle for aminophosphine of 82.17(3)° was observed in **1b** and the largest angle of 84.26(4)° in **1i**, bearing alkyl substituents on both the phosphorus and nitrogen atoms. In between were **1a** and **2g** with angles of 83.54(4)° and 83.84(4)°. A similar bite angle is known from the PP-supported complex where *fac*-[Mn(*n*Pr_2_PCH_2_CH_2_P*n*Pr_2_)(CO)_3_Br] displays an angle of 83.56(4)°.^13^ Finally, the alkyl complex **PN1** was confirmed via X-ray diffraction. A structural view is shown in Figure 1, (d). When comparing the crystal structures of **2g** and **PN1**, a noticeable shortening of the Mn and basal CO distances is observed in **PN1**. These Mn—CO bond distances are 1.767 Å (Mn1–C1) and 1.801 Å (Mn1–C2) in **PN1**, while they are 1.811 Å (Mn1–C1) and 1.849 Å (Mn1–C2) in **2g**, respectively. The apical CO distance to the Mn center is shorter in **2g** at 1.780 Å (Mn1–C3) than in **PN1** at 1.881 Å (Mn1–C3). This could be attributed to a stronger trans influence of the methyl group than that of the triflate group. The Mn–alkyl distance was 2.065 Å (Mn1–C20). The PN bite angle is nearly the same in both structures, 83.84(4)° in **2g** and 83.65(7)° in **PN1**. A slightly larger bite angle of 85.53(2)° can be observed for the alkylated species **PP3**.^15^ Selected bond distances and bite angles are presented in Table 1.

**Table 1:** Selected Bond Distances (Å) and the Bite Angle (°) of **1b**, **1i**, **2g**, and **PN1**

	**1b**	**1i**	**2g**	**PN1**
Mn1–P1	2.323	2.342	2.342	2.316
Mn1–N1	2.239	2.186	2.189	2.210
P1–Mn1–N1	82.17(3)	84.26(4)	83.84(4)	83.65(7)

### Catalytic Applications

At last, we were interested in the catalytic performance of PN-supported Mn(I) alkyl carbonyl complexes in hydrofunctionalization reactions. Preliminary investigations focused on the dimerization of phenylacetylene and the hydroboration of 4-chlorostyrene.

### Dimerization of Phenylacetylene

**Scheme 4:**
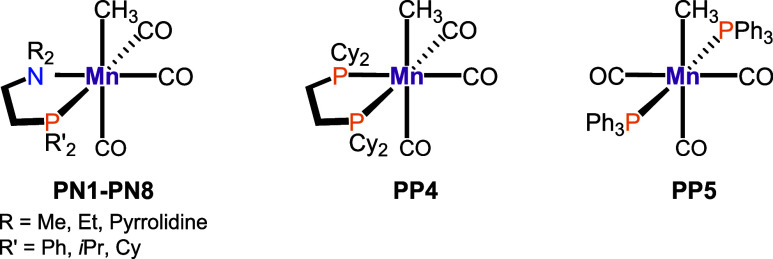
Investigated Mn(I) Methyl Complexes as Catalysts in This Study

**PN1**–**PN8, PP4**, and **PP5**(37) (Scheme 4) were applied in the dimerization of phenylacetylene; the results are summarized in Table 2 and compared with previously reported bisphosphine-based catalysts.

**Table 2:**
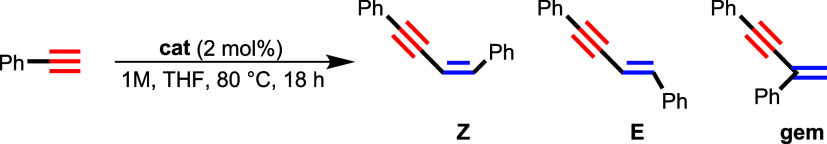
Catalytic Performance of PN- and PP-Based Mn(I) Methyl Complexes in the Dimerization of Phenylacetylene^a^

catalyst	conversion [%]	*Z*/*E*
**PN1**	75	95:5
**PN2**	35	96:4
**PN3**	14	90:10
**PN4**	12	90:10
**PN5**	n.d.	n.d.
**PN7**	n.d.	n.d.
**PN6**	trace	n.d.
**PN8**	n.d.	n.d.
**PP3**^b^	>99	96:4
**PP2**^b^	67	79:21
**PP4**	36	91:9
**PP5**	n.d	n.d.
none	n.d	n.d.

a Reaction conditions: phenylacetylene (0.600 mmol, 1.00 equiv), catalyst (0.012 mmol, 2.00 mol %), THF (1 M), 80 °C for 18 h; conversion, E/Z ratio detected by GC-MS, n.d. = not detected.

b Data taken from ref (17).

Under the given reaction conditions, **PN1** achieved the best conversion (75%) and a *Z*-selectivity of 95%. A massive drop in reactivity was observed when the cyclohexyl groups were exchanged with isopropyl groups on the P-donor (**PN2**). Moving from methyl to pyrrolidine substituents on the nitrogen donor led to an additional loss in activity (**PN3**, **PN4, PN7**). By replacing the substituents on the nitrogen atom with ethyl groups, the conversion drops to a few precent (**PN6**). Furthermore, no reactivity of **PN5** was detected. This emphasizes the significance of appropriate substituents on both, the phosphorus and nitrogen atoms. Given the lack of any conversion with aryl phosphines, it is evident that a stronger and sterically more demanding σ-donor, such as alkylphosphine, is required to catalyze this given reaction. Thus far, **PP3** still performed the best in this transformation, exhibiting excellent conversion and *Z*-selectivity.

Nonetheless, **PN1** outperformed **PP2** in terms of conversion and selectivity. Regarding migratory insertion, a propyl group is known to have a greater rate than a methyl group; therefore, a comparison of methylated species would be more equitable.^38,39^ When the methylated compounds are compared, it becomes evident that the PN-supported complex **PN1** is superior to PP-supported **PP4**, emphasizing the benefits of mixed aminophosphine bidentates. Furthermore, no catalytic transformation was observed when utilizing **PP5**, bearing two triphenylphosphine monodentate ligands. Encouraged by these findings, we decided to screen a series of conditions to improve the performance of **PN1**. The best result was obtained in EtOH (95%), when compared to MeOH, *i*-PrOH, THF, toluene, ACN, and DCE. A slight change in selectivity was observed, and no geminal product was detected.

### Hydroboration of 4-Chlorostyrene

Next, selected PN complexes were tested for the hydroboration of 4-chlorostyrene. The results are summarized in Table 3. Conducted at a catalyst loading of 1.00 mol %, the hydroboration of 4-chlorstyrene was enabled by all synthesized catalysts with a high selectivity toward the anti-Markovnikov product A. Contrary to the dimerization of phenylacetylene, the use of phenyl groups manifested the best results, reaching an excellent conversion rate of 99% when utilizing **PN5**. Hence, this complex performed almost as well as the previously reported PP complex **PP3**.^18^ A similar reactivity was observed for **PN6** (93% conversion), followed by **PN2** (67% conversion) and **PN1** (41% conversion).

**Table 3:**
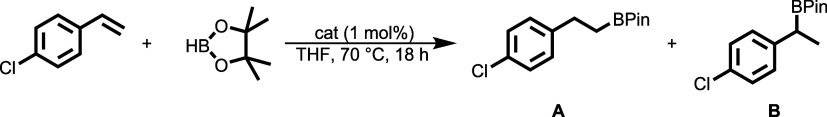
Catalytic Performance of PN- and PP-Based Mn(I) Methyl Complexes in the Hydroboration of 4-Chlorostyrene

catalyst	conversion [%]	A/B
**PN1**	41	>99:1
**PN2**	67	>99:1
**PN3**	22	>99:1
**PN4**	16	97:3
**PN5**	99	>99:1
**PN6**	93	>99:1
**PN7**	43	99:1
**PN8**	57	>99:1
**PP3**^b^	>99	97:3
**PP4**	80	>99:1
**PP5**	87	>99:1
none	6	>99:1

a Reaction conditions: 4-chlorostyrene (1.10 mmol, 1.00 equiv), pinacolborane (1.12 mmol, 1.02 equiv), catalyst (0.011 mmol, 1.00 mol %), THF (2 M), 18 h, 70 °C; conversion, A:B ratio detected by GC-MS.

b Data taken from ref (19).

Interestingly, the pyrrolidine-based complexes **PN3**, **PN4**, and **PN7** performed poorly with a conversion of 22, 16, and 43%, respectively. These findings indicate that the choice of substituents regarding the nitrogen atom is more important than for the phosphorus atom. Good conversion was achieved with complex **PP4** (80%) and **PP5** (87%). Nonetheless, a suitable combination of mixed PNs, such as in **PN5**, can outperform mono- and bisphosphine-based Mn(I) alkyl carbonyl complexes, emphasizing, again, the advantage of an unsymmetrical donor set.

## Conclusions

In summary, we developed a procedure to synthesize PN Mn(I) alkyl carbonyl complexes. The bromide species were readily transformed into the triflate analogues using AgOTf. MeLi and MeMgCl were used as alkylation agents for synthesizing initial PN-supported Mn(I) methyl carbonyl compounds. The crystal structures of **1b**, **1i**, **2g**, and **PN1** verified the octahedral coordination sphere with *fac*-arranged carbonyls, which is in alignment with the IR data. Preliminary investigations revealed that *fac*-[Mn(P^Cy^N^Me^)(CO)_3_Me] (**PN1**) is the best precatalyst for the dimerization of phenylacetylene. In addition, we demonstrated the hydroboration of 4-chlorstyrene, facilitated by all described complexes with a preferred *anti*-Markovnikov product formation. Especially, *fac*-[Mn(P^Ph^N^Me^)(CO)_3_Me] (**PN5)** gave excellent conversion and high selectivity.

## Supporting Information Available

Synthetic procedures; ^1^H, ^13^C{1H}, and ^31^P{H} NMR spectra of all compounds; and crystallographic data and the corresponding references (PDF)
