# Supplementary material for: Synthesis and Application of PN-Supported Mn(I) Carbonyl Alkyl Complexes
Source: Organometallics. 2025 Apr 30;44(9):1006–11. doi: 10.1021/acs.organomet.5c00095 (PMC12076549; doi:10.1021/acs.organomet.5c00095)
Supplement: Supplementary file 1 — om5c00095_si_001.pdf [file om5c00095_si_001.pdf]

Supporting Information

Synthesis and Application of PN-supported Mn(I) Carbonyl Alkyl Complexes

Claudia Rabijasz,[a] Stefan Weber,[a,*] Berthold Stöger,[b] Karl Kirchner [a,*]

[a]Institute of Applied Synthetic Chemistry, TU Wien, Getreidemarkt 9/163-AC, A-1060 Wien, Austria.

[b] X-Ray Center, TU Wien, Getreidemarkt 9, A-1060 Vienna, Austria.

email: stefan.e163.weber@tuwien.ac.at
email: karl.kirchner@tuwien.ac.at

Table of Content

1 General Information. 1

2 Ligand Synthesis and Characterization 2

Route A 2

2.1 2-(Diphenylphosphino)-*N*,*N*-dimethylethanamine (L1) 2

Route B 2

2.2 2-(Diphenylphosphino)-*N*,*N*-diethylethanamine (L2) 2

2.3 1-[2-(Diphenylphosphino)ethyl]pyrrolidine (L3) 3

2.4 [2-(Diisopropylphosphine)ethyl]dimethylamine (L4) 3

2.5 2-(Diisopropylphosphino)-*N,N*-diethylethanamine (L5) 3

2.6 1-[2-(Diisopropylphosphine)ethyl]pyrrolidine (L6) 3

2.7 2-(Dicyclohexylphosphino)-*N, N*-dimethylethanamine (L7) 4

2.8 2-(Dicyclohexylphosphino)-*N,N*-diethylethanamine (L8) 4

2.9 1-[2-(Dipcyclohexylsphino)ethyl]pyrrolidine (L9) 4

3 Synthesis and Characterization of PN-supported Mn(I) Bromide Complexes 4

3.1 *fac*-[Mn($P^{Ph}N^{Me}$)$(CO)_3$Br] (1a) 5

3.2 *fac*-[Mn($P^{Ph}N^{Et}$)$(CO)_3$Br] (1b) 5

3.3 *fac*-[Mn($P^{Ph}N^{Pyrr}$)$(CO)_3$Br] (1c) 5

3.4 *fac*-[Mn($P^{iPr}N^{Me}$)$(CO)_3$Br] (1d) 5

3.5 *fac*-[Mn($P^{iPr}N^{Et}$)$(CO)_3$Br] (1e) 6

3.6 *fac*-[Mn($P^{iPr}N^{Pyrr}$)$(CO)_3$Br] (1f) 6

3.7 *fac*-[Mn($P^{Cy}N^{Me}$)$(CO)_3$Br] (1g) 6

3.8 *fac*-[Mn($P^{Cy}N^{Et}$)$(CO)_3$Br] (1h) 6

3.9 *fac*-[Mn($P^{Cy}N^{Pyr}$)$(CO)_3$Br] (1i) 7

4 Synthesis and Characterization of PN-supported Mn(I) Triflate Complexes 7

4.1 *fac*-[Mn($P^{Ph}N^{Me}$)$(CO)_3$OTf] (2a) 7

4.2 *fac*-[Mn($P^{Ph}N^{Et}$)$(CO)_3$OTf] (2b) 7

4.3 *fac*-[Mn($P^{Ph}N^{Pyrr}$)$(CO)_3$OTf] (2c) 8

4.4 *fac*-[Mn($P^{iPr}N^{Me}$)$(CO)_3$OTf] (2d) 8

4.5 *fac*-[Mn($P^{iPr}N^{Et}$)$(CO)_3$OTf] (2e) 8

4.6 *fac*-[Mn($P^{iPr}N^{Pyrr}$)$(CO)_3$OTf] (2f) 8

4.7 *fac*-[Mn($P^{Cy}N^{Me}$)$(CO)_3$OTf] (2g) 8

4.8 *fac*-[Mn($P^{Cy}N^{Et}$)$(CO)_3$OTf] (2h) 9

4.9 *fac*-[Mn($P^{Cy}N^{Pyrr}$)$(CO)_3$OTf] (2i) 9

5 Synthesis and Characterization of PN-supported Mn(I) Alkyl Complexes 9

5.1 *fac*-[Mn($P^{Cy}N^{Me}$)$(CO)_3CH_3$] (PN1) 10

5.2 *fac*-[Mn($P^{iPr}N^{Me}$)$(CO)_3CH_3$] (PN2) 10

5.3 *fac*-[Mn($P^{iPr}N^{Pyrr}$)$(CO)_3CH_3$] (PN3) 10

5.4 *fac*-[Mn($P^{Cy}N^{Pyrr}$)$(CO)_3CH_3$] (PN4) 11

5.5 *fac*-[Mn($P^{Ph}N^{Me}$)$(CO)_3CH_3$] (PN5) 11

5.6 *fac*-[Mn($P^{Cy}N^{Et}$)$(CO)_3CH_3$] (PN6) 11
5.7 *fac*-[Mn($P^{Ph}N^{Pyrr}$)$(CO)_3CH_3$] (PN7) 12
5.8 *fac*-[Mn($P^{Ph}N^{Et}$)$(CO)_3CH_3$] (PN8) 12
6 Synthesis and Characterization of a PP-based bidentate ligand and the corresponding Mn(I) Carbonyl Complexes 12
7 Synthesis and Characterization of Mn(I) Carbonyl Complexes supported by monodentate Ligands 13
8 Catalytic Dimerization of Phenylacetylene 14
8.1 General Procedure 14
8.2 Optimization Reactions for the Dimerization of Phenylacetylene 14
9 Catalytic Hydroboration of 4-Chlorstyrene 15
10 NMR/IR spectra 16
10.1 PN-bidentate ligands and the corresponding Mn(I) Carbonyl Complexes 16
10.2 PP-based Mn(I) Carbonyl Complexes 86
11 Crystallographic Data 94
12 References 97

1 General Information.

All reactions were performed under inert atmosphere of argon using Schlenk techniques or in a MBraun inert-gas glovebox, unless otherwise noted. The solvents were purified according to standard procedures.[1] All used reagents and solvents were purchased from commercial suppliers and directly used without further purification, if not stated otherwise. The deuterated solvents were purchased from Aldrich and dried over 3 Å molecular sieves.

^{1}H and ^{13}C{^{1}H} and ^{31}P{^{1}H}-NMR spectra were recorded on Bruker AVANCE-250, AVANCE-400 and AVANCE-600 spectrometers. ^{1}H and ^{13}C{^{1}H}-NMR spectra were reference internally to residual protio-solvent and solvent resonance, respectively, and are reported relative to tetramethylsilane (δ = 0 ppm). ^{31}P{^{1}H}-NMR spectra were referenced externally to H_3PO_4 (85%) (δ = 0 ppm). All IR measurements were performed on a Bruker Tensor 27 with an ATR unit.

GC-MS analysis was conducted on a ISQ LT Single quadrupole MS (Thermo Fisher) directly interfaced to a TRACE 1300 Gas Chromatographic systems (Thermo Fisher), using a Rxi-5Sil MS (30 m, 0.25 mm ID) cross-bonded dimethyl polysiloxane capillary column at a carrier flow of He 1.5 mL/min.

High-resolution accurate mass spectra were acquired on an Agilent 6545 QTOF mass spectrometer (Agilent Technologies, Santa Clara, CA, USA), which is equipped with a dual electrospray ion source. The calibration of the instrument was performed utilizing the commercially available ES tuning mix from Agilent Technologies (Waldbronn, Germany). The sample solution was introduced into the ion source via direct infusion utilizing a syringe pump model 100 (KD Scientific, Holliston, MA, USA). Measured accurate mass data for confirming calculated elemental compositions were typically within ±5 ppm accuracy.

X-ray Structure Determination. X-ray diffraction data of **1a** (CCDC 2423049), **1b** (2423050), **1i** (2423051), **2g** (2423052) and **PN1** (2423053) were collected at T = 100 K in a dry stream of nitrogen on a Bruker Kappa APEX II diffractometer system using graphite-monochromatized Mo-$K\alpha$ radiation (λ = 0.71073 Å) and fine sliced φ- and ω-scans. Data were reduced to intensity values with SAINT and an absorption correction was applied with the multi-scan approach implemented in SADABS.[2] The structures were solved by the dual-space approach implemented in SHELXT[3]and refined against F^2 with SHELXL.[4] Non-hydrogen atoms were refined with anisotropic displacement parameters. H atoms were placed in calculated positions and thereafter refined as riding on the parent atoms. Molecular graphics were generated with the program MERCURY.[5]

2 Ligand Synthesis and Characterization

Route A

2.1 2-(Diphenylphosphino)-*N*,*N*-dimethylethanamine (L1)

Scheme S1. Synthesis of L1, which is a modified procedure from the literature.[6]

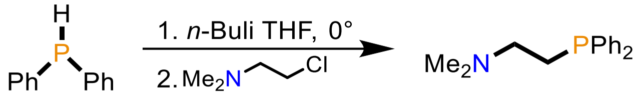

L1

Analytical data is consistent with the literature.[7,8,9] In a Schlenk flask, PPh_2H (1.70 mL, 9.72 mmol, 1.00 equiv.) was mixed with THF (25 mL). After cooling at 0 °C *n*-BuLi (6.10 mL, 1.6 M in *n*-hexane, 9.76 mmol, 1.00 equiv.) was added dropwise. Shortly afterwards, $NMe_2(CH_2)_2Cl$ (1.06 g, 9.85 mmol, 1.01 equiv.) was added slowly and the solution mixture was stirred overnight at RT. The colorless suspension was quenched with deoxygenated H_2O (5 mL) and the aqueous layer was separated. The organic layer was dried over Na_2SO_4, filtrated and the solvent was evaporated. The colorless residue was taken up in *n*-pentane (20 mL) and filtered over a pad of silica gel. After evaporation of the solvent, **L1** was obtained as colorless viscous oil (750 mg, 41 %). ^{1}H NMR (400 MHz, C_6D_6): δ = 7.49 – 7.44 (m, 4H, $Ph^{2,6}$), 7.11 – 7.04 (m, 6H, $Ph^{3,4,5}$), 2.43 – 2.36 (m, 2H, NCH_2), 2.24 – 2.17 (m, 2H, PCH_2), 2.03 (s, 6H, NCH_3). $^{13}C\{^1H\}$ NMR (101 MHz, C^6D^6): δ = 139.9 (Ph^1), 133.2 (d, J = 18.8 Hz, $Ph^{2,6}$), 128.7 (d, J = 6.5 Hz, $Ph^{3,5}$), 128.6 (Ph^4), 56.5 ($N\underline{C}H_2CH_2P$), 45.2 (NCH_3), 27.3 (d, J = 12.8 Hz, $NCH_2\underline{C}H_2P$). $^{31}P\{^1H\}$ NMR (162 MHz, C_6D_6): δ = -19.7.

Route B

Scheme S2. Synthesis of L2-L3, which is a modified procedure from the literature.[10]

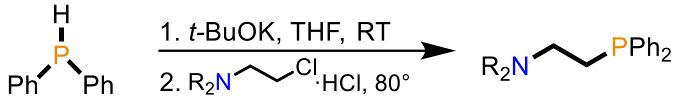

R = Et, Pyrrolidine

L2-L3

In a Schlenk flask, PPh_2H was added dropwise to a suspension of *t*-BuOK in THF and stirred for 30 minutes at RT. Afterwards the orange suspension was mixed with the respective amine salt $R_2N(CH_2)_2Cl{\cdot}HCl$ and refluxed overnight. During the reaction the colour changed from orange to white. The suspension was quenched with deoxygenated H_2O. The aqueous layer was separated and the organic layer was dried over Na_2SO_4. The product was extracted with *n*-pentane (3 x 7 mL) and filtered over a pad of silica. After evaporation of the solvent, the pure product was obtained as a colorless to orange oil (**L2, L3**).

2.2 2-(Diphenylphosphino)-*N*,*N*-diethylethanamine (L2)

Analytical data is consistent with the literature.[9] PPH_2H (1.70 mL, 9.77 mmol, 1.00 equiv.); *t*-BuOK (2.63 g, 23.4 mmol, 2.40 equiv.); 2-Chloro-N,N-diethylethanamine hydrochloride (1.78 g, 10.3 mmol, 1.06 equiv.); solvent: 30 mL THF; quenched with 10 mL deoxygenated H_2O; 820 mg (29 %) as orange oil. ^{1}H NMR (400 MHz, C_6D_6) δ = 7.52 – 7.43 (m, 4H, $Ph^{2,6}$), 7.13 – 7.00 (m, 6H, $Ph^{3,4,5}$), 2.67 – 2.59 (m, 2H, $PCH_2C\underline{H_2}N$), 2.36 (q, J = 7.1 Hz, 4H, $NC\underline{H_2}CH_3$), 2.26 – 2.18 (m, 2H, $PC\underline{H_2}CH_2N$), 0.88 (t, J = 7.1 Hz, 6H, $NCH_2C\underline{H_3}$). $^{13}C\{^1H\}$ NMR (101 MHz, C_6D_6) δ: = 140.0 (d, J = 14.8 Hz, Ph^1), 133.2 (d, J = 18.8 Hz, $Ph^{2,6}$), 128.7 ($Ph^{3,4,5}$), 128.6 (d, J = 3.3 Hz, $Ph^{3,4,5}$), 49.8 (d, J = 22.2 Hz, $PCH_2\underline{C}H_2N$), 46.9 ($N\underline{C}H_2CH_3$), 26.6 (d, J = 13.1 Hz, $P\underline{C}H_2CH_2N$), 12.4 ($NCH_2\underline{C}H_3$), $Cq_{(CO)}$ not observed.$^{31}P\{^1H\}$ NMR (162 MHz, C_6D_6) δ: = -19.4. HRMS (ESI): *m/z* for $C_{18}H_{24}NP$ $[M+H]^+$ = 286.1719 (calc.), found: 286.1718.

2.3 1-[2-(Diphenylphosphino)ethyl]pyrrolidine (L3)

PPh_2H (1.38 g, 7.41 mmol, 1.00 equiv.); *t*-BuOK (2.00 g, 17.8 mmol, 2.40 equiv.); 1-(2-Chloroethyl)pyrrolidine hydrochloride (1.52 g, 8.94 mmol, 1.21 equiv.); solvent: 22 mL THF; quenched with 6 mL deoxygenated H_2O; 1.20 mg (57 %) as yellow oil.^{1}H NMR (400 MHz, C_6D_6) δ: = 7.51 – 7.45 (m, 4H, $Ph^{2,6}$), 7.12 – 7.02 (m, 6H, Ph^{3-5}), 2.70 – 2.57 (m, 2H), 2.36 – 2.25 (m, 6H), 1.61 – 1.49 (m, 4H).$^{13}C\{^1H\}$ NMR (101 MHz, CD_2Cl_2) δ: = 140.0 (d, *J* = 14.8 Hz, Ph^1), 133.2 (d, *J* = 18.9 Hz, $Ph^{2,6}$), 128.7 (Ph^{3-5}), 128.7 (Ph^{3-5}), 128.6 (Ph^{3-5}), 54.0 ($N\underline{C}H_2CH_2CH_2\underline{C}H_2$), 53.1 (d, *J* = 21.2 Hz, $N\underline{C}H_2CH_2P$), 28.7 (d, *J* = 12.7 Hz, $NCH_2\underline{C}H_2P$), 23.9 ($NCH_2\underline{C}H_2\underline{C}H_2CH_2$).$^{31}P\{^1H\}$ NMR (162 MHz, C_6D_6) δ: = -19.4. HRMS (ESI): *m/z* for $C_{13}H_{22}NP$ $[M+H]^+$ = 284.1562 (calc.), found: 284.1531.

Route C

Scheme S3. Synthesis of L4-L9.

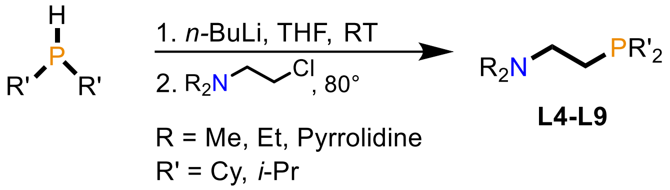

General procedure

In a Schlenk flask, *n*-BuLi was added dropwise to a solution of the phosphine dissolved in THF and stirred at RT for 2 hours. The corresponding free amine was added dropwise to the refluxing yellow solution over 10 minutes. After 1.5 hours, stirring was continued for another hour at RT. THF was evaporated under reduced pressure and the mixture was extracted with *n*-pentane or toluene. After filtration over a pad of silica, the solvent was evaporated yielding a colourless or orange viscous oil (**L4-L9**).

2.4 [2-(Diisopropylphosphine)ethyl]dimethylamine (L4)

Analytical data is consistent with the literature.[11] *i*-Pr_2PH (462 mg, 3.91 mmol, 1.00 equiv); *n*-BuLi (2.35 mL, 2.5 M, 5.89 mmol, 1.50 equiv.); $Me_2N(CH_2)_2Cl$ (548 mg, 5.09 mmol, 1.30 equiv.); solvent: 10 mL THF); extracted with *n*-pentane (4 x 10 mL); 322 mg (44 %) as colourless oil. ^{1}H NMR (400 MHz, C_6D_6) δ: = 2.52 – 2.44 (m, 2H, $PC\underline{H}CH_3$), 2.13 (s, 6H, $NC\underline{H_3}$), 1.65 – 1.50 (m, 4H, $NC\underline{H_2}C\underline{H_2}P$), 1.07 – 0.97 (m, 12H, $PCHC\underline{H_3}$). $^{13}C\{^1H\}$ NMR (101 MHz, C_6D_6) δ: = 59.4 (d, *J* = 27.9 Hz, $N\underline{C}H_2CH_2P$), 45.3 (NCH_3), 23.7 (d, *J* = 14.1 Hz, $PCH\underline{C}H_3$), 20.9 (d, *J* = 19.3 Hz, $NCH_2\underline{C}H_2P$), 20.3 (d, *J* = 16.6 Hz, $P\underline{C}HCH_3$), 19.0 (d, *J* = 10.0 Hz, $PCH\underline{C}H_3$). $^{31}P\{^1H\}$ NMR (162 MHz, C_6D_6) δ: = 0.4. HRMS (ESI): *m/z* for $C_{10}H_{24}NP$ $[M+H]^+$ = 190.1719 (calc.), found:190.1721.

2.5 2-(Diisopropylphosphino)-*N*,*N*-diethylethanamine (L5)

i-Pr_2PH (939 g, 7.94 mmol, 1.00 equiv.); *n*-BuLi (4.80 mL, 2.5 M, 12.0 mmol, 1.51 equiv.); $Et_2N(CH_2)_2Cl$ (1.21 g, 8.92 mmol, 1.12 equiv.); solvent: 20 mL THF; extracted with *n*-pentane (4x 10 mL); 688 mg (40 %) as colourless viscous oil. ^{1}H NMR (400 MHz, C_6D_6) δ: = 2.73 – 2.66 (m, 2H, $NC\underline{H_2}CH_2P$), 2.46 (q, *J* = 7.1 Hz, 4H, $NC\underline{H_2}CH_3$), 1.67 – 1.48 (m, 4H, $NCH_2C\underline{H_2}P$, $PC\underline{H}CH_3$), 1.08 – 0.97 (m, 18H, $PCHC\underline{H_3}$, $NCH_2C\underline{H_3}$). $^{13}C\{^1H\}$ NMR (101 MHz, C_6D_6) δ: = 52.6 (d, *J* = 28.7 Hz, $N\underline{C}H_2CH_2P$), 47.1 ($N\underline{C}H_2CH_3$), 23.7 (d, *J* = 14.1 Hz, $P\underline{C}HCH_3$), 20.4 (d, *J* = 16.5 Hz, $PCH\underline{C}H_3$), 20.2 (d, *J* = 19.5 Hz, $NCH_2C\underline{H_2}P$), 19.0 (d, *J* = 10.0 Hz, $PCH\underline{C}H_3$), 12.5 ($NCH_2\underline{C}H_3$). ^{31}P NMR (162 MHz, C_6D_6) δ: = 0.5. HRMS (ESI): *m/z* for $C_{12}H_{28}NP$ $[M+H]^+$ = 218. 2032 (calc.), found: 218.2032.

2.6 1-[2-(Diisopropylphosphine)ethyl]pyrrolidine (L6)

i-Pr_2PH (1.35 g, 11.4 mmol 1.00 equiv.); *n*-BuLi (6.86 mL, 2.5 M, 17.2 mmol, 1.50 equiv.); 1-(2-Chloroethyl)pyrrolidine (1.52 g, 11.83 mmol, 1.00 equiv.); solvent: 25 mL THF; extracted with *n*-pentane (3 x 15 mL); 1.23 g (50 %) as colourless viscous oil. ^{1}H NMR (400 MHz, C_6D_6) δ: = 2.73 – 2.63 (m, 2H, $NC\underline{H_2}CH_2P$), 2.48 – 2.33 (m, 4H, $NC\underline{H_2}CH_2CH_2C\underline{H_2}$), 1.68 – 1.54 (m, 8H, $PC\underline{H}$, $NCH_2C\underline{H_2}P$, $NCH_2C\underline{H_2}C\underline{H_2}CH_2$), 1.12 – 0.98 (m, 12H, $PCHC\underline{H_3}$). $^{13}C\{^1H\}$ NMR (101 MHz, C_6D_6)

δ: = 55.9 (d, J = 27.8 Hz, $N\underline{C}H_2CH_2P$), 54.2 ($N\underline{C}H_2CH_2CH_2\underline{C}H_2$), 24.0 ($NCH_2\underline{C}H_2\underline{C}H_2CH_2$), 23.7 (d, J = 13.8 Hz, $P\underline{C}H$), 22.3 (d, J = 19.0 Hz, $NCH_2\underline{C}H_2P$), 20.3 (d, J = 16.4 Hz, $PCH\underline{C}H_3$), 19.0 (d, J = 10.0 Hz, $PCH\underline{C}H_3$). $^{31}P\{^1H\}$ NMR (162 MHz, C_6D_6) δ: = 0.4. HRMS (ESI): *m/z* for $C_{12}H_{26}NP$ $[M+H]^+$ = 296.2501 (calc.), found: 296.2505.

2.7 2-(Dicyclohexylphosphino)-*N, N*-dimethylethanamine (L7)

Analytical data is consistent with the literature.[9] Cy_2PH (1.10 g, 5.55 mmol 1.00 equiv.); *n*-BuLi (2.67mL, 2.5 M, 6.66 mmol, 1.20 equiv.); $Me_2N(CH_2)_2Cl$ (776 mg, 7.21 mmol, 1.30 equiv.); solvent: 18 mL THF; extracted with toluene (3x 10 mL); 694 mg (46 %) as yellow viscous oil. ^1H NMR (400 MHz, C_6D_6) δ: = 2.58 – 2.47 (m, 2H, NCH2CH2P), 2.17 (s, 6H, NCH3), 1.90 – 1.46 (m, 18H, NCH_2CH_2P, Cy), 1.34 – 1.04 (m, 6H, NCH_2CH_2P, Cy). $^{13}C\{^1H\}$ NMR (101 MHz, C6D6) δ: = 59.8 (d, J = 29.5 Hz, NCH2CH2P), 45.4 (NCH3), 33.9 (d, J = 14.7 Hz, Cy1), 30.8 (d, J = 15.3 Hz, Cy2-6), 29.4 (d, J = 8.5 Hz, Cy2-6), 27.7 (d, J = 17.6 Hz, Cy2-6), 27.7 (Cy2-6), 27.0 (Cy2-6), 20.7 (d, J = 18.8 Hz, NCH2CH2P). $^{31}P\{1H\}$ NMR (162 MHz, C_6D_6) δ: = -7.9. HRMS (ESI): *m/z* for $C_{16}H_{32}NP$ $[M+H]^+$ = 270.2345 (calc.), found: 270.2344.

2.8 2-(Dicyclohexylphosphino)-*N,N*-diethylethanamine (L8)

Analytical data is consistent with the literature.[9] Cy_2PH (1.00 g, 5.04 mmol, 1.00 equiv.); *n*-BuLi (3.00 mL, 2.5 M, 7.50 mmol, 1.49 equiv.); $Et_2N(CH_2)_2Cl$ (820 mg, 6.05 mmol, 1.20 equiv.); solvent: 18 mL THF; extracted with toluene (3x 10 mL); 548 mg (37 %) as yellow viscous oil. ^1H NMR (400 MHz, C_6D_6) δ: = 2.80 – 2.70 (m, 2H, $NC\underline{H_2}C\underline{H_2}P$), 2.50 (q, J = 7.1 Hz, 4H, $NC\underline{H_2}CH_3$), 1.90 – 1.49 (m, 13H, $NC\underline{H_2}C\underline{H_2}P$, Cy), 1.41 – 1.09 (m, 11H, $NC\underline{H_2}C\underline{H_2}P$, Cy), 1.03 (t, J = 7.1 Hz, 6H, $NCH_2C\underline{H_3}$).$^{13}C\{^1H\}$ NMR (101 MHz, C_6D_6) δ: = 53.0 (d, J = 29.7 Hz, $N\underline{C}H_2CH_2P$), 47.1($N\underline{C}H_2CH_3$), 33.9 (d, J = 14.8 Hz, Cy^1), 30.9 (d, J = 15.3 Hz, $Cy^{2\text{-}6}$), 29.5 (d, J = 8.6 Hz, $Cy^{2\text{-}6}$), 27.7 (d, J = 16.6 Hz, $Cy^{2\text{-}6}$), 27.7 (d, J = 1.8 Hz, $Cy^{2\text{-}6}$), 27.0 ($Cy^{2\text{-}6}$), 20.0 (d, J = 19.3 Hz, $NCH_2\underline{C}H_2P$), 12.6 ($NCH_2\underline{C}H_3$). $^{31}P\{^1H\}$ NMR (162 MHz, C_6D_6) δ: = -7.8. HRMS (ESI): *m/z* for $C_{18}H_{36}NP$ $[M+H]^+$ = 298.2658 (calc.), found: 298.2661.

2.9 1-[2-(Dipcyclohexylphosphino)ethyl]pyrrolidine (L9)

Cy_2PH (1.08 g, 5.45 mmol, 1.00 equiv.); *n*-BuLi (3.00 mL, 2.5 M, 7.50 mmol, 1.49 equiv.); 1-(2-Chloroethyl)pyrrolidine (820 mg, 6.05 mmol, 1.20 equiv.); solvent: 18 mL THF; extracted with toluene (3x 10 mL); 548 mg (37 %) as colorless viscous oil. ^1H NMR (400 MHz, C_6D_6) δ: = 2.79 – 2.70 (m, 2H, $NC\underline{H_2}CH_2P$), 2.52 – 2.42 (m, 4H, $NC\underline{H_2}CH_2CH_2C\underline{H_2}$), 1.92 – 1.83 (m, 2H, Cy), 1.83 – 1.75 (m, 2H, Cy), 1.75 – 1.67 (m, 6H, Cy, $NCH_2\underline{C}H_2P$), 1.67 – 1.59 (m, 6H, Cy, $NCH_2C\underline{H_2}C\underline{H_2}CH_2$), 1.59 – 1.50 (m, 2H, Cy^1), 1.34 – 1.08 (m, 10H, Cy). ^{13}C NMR (101 MHz, C_6D_6) δ: = 56.3 (d, J = 29.1 Hz, $N\underline{C}H_2CH_2P$), 54.2 (s, $N\underline{C}H_2CH_2CH_2\underline{C}H_2$), 33.9 (d, J = 14.5 Hz, Cy^1), 30.8 (d, J = 15.2 Hz, $Cy^{2\text{-}5}$), 29.5 (d, J = 8.6 Hz, $Cy^{2\text{-}5}$), 27.8 ($Cy^{2\text{-}5}$), 27.7 ($Cy^{2\text{-}5}$), 27.6 ($Cy^{2\text{-}5}$), 27.0 ($Cy^{2\text{-}5}$), 24.0 ($NCH_2\underline{C}H_2\underline{C}H_2CH_2$), 22.1 (d, J = 18.5 Hz, $NCH_2\underline{C}H_2P$). $^{31}P\{^1H\}$ NMR (162 MHz, C_6D_6) δ: = -7.9. HRMS (ESI): *m/z* for $C_{18}H_{34}NP$ $[M+H]^+$ = 216.1875 (calc.), found: 216.1897.

3 Synthesis and Characterization of PN-supported Mn(I) Bromide Complexes

Scheme S4. Complexation of PN-Ligands with $[Mn(CO)_5Br]$.

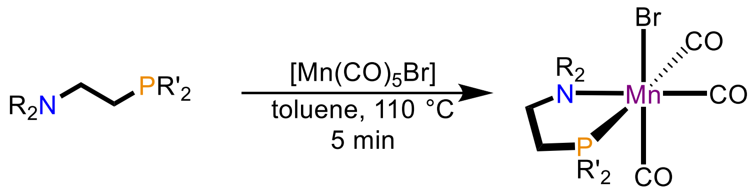

L1-L9 R = Me, Et, Pyrrolidine **1a-1i**
R' = Ph, Cy, *i*Pr

General Procedure:

In a Schlenk flask, the respective ligand **L** was dissolved in toluene and mixed with $Mn(CO)_5Br$. The suspension was refluxed for 5 minutes, thereby releasing CO gas. In the case of **1h**, it was necessary to heat at 80° for 2 hours to achieve complete complexation. The orange solution was concentrated to one third. Afterwards the complex was precipitated with MeOH or *n*-pentane and washed with the corresponding solvent (2 x 5 mL). Upon drying, the product **1** was obtained as yellow or orange powder.

3.1 *fac*-[Mn($P^{Ph}N^{Me}$)$(CO)_3$Br] (1a)

Analytical data is consistent with the literature.[12] **L1** (1.62 g, 6.30 mmol, 1.00 equiv.); $Mn(CO)_5Br$ (1.73 g, 6.29 mmol, 1.00 equiv.), solvent: 35 mL toluene; precipitated and washed with MeOH; 1.90 g (63 %) of a yellow powder. ^{1}H NMR (400 MHz, CD_2Cl_2) δ: = 7.79 – 7.67 (m, 2H, $Ph^{2,6}$), 7.64 – 7.55 (m, 2H, $Ph^{2,6}$), 7.53 – 7.47 (m, 3H, $Ph^{3,4,5}$), 7.45 – 7.39 (m, 3H, $Ph^{3,4,5}$), 3.23 (qd, *J* = 12.4, 4.2 Hz, 1H, $NC\underline{H_2}CH_2P$), 3.05 – 2.88 (m, 1H, $NC\underline{H_2}CH_2P$), 2.88 (s, 3H, NCH_3), 2.73 (s, 3H, NCH_3), 2.71 – 2.58 (m, 1H, $NCH_2C\underline{H_2}P$), 2.57 – 2.40 (m, 1H, $NCH_2C\underline{H_2}P$). $^{13}C\{^1H\}$ NMR (101 MHz, CD_2Cl_2) δ: = 135.5 (d, *J* = 41.8 Hz, Ph^1), 133.5 (d, *J* = 40.3 Hz, Ph^1), 132.9 (d, *J* = 9.1 Hz, $Ph^{3,5}$), 132.5 (d, *J* = 10.0 Hz, $Ph^{2,6}$), 131.4 (d, *J* = 2.1 Hz, Ph^4), 130.7 (d, *J* = 2.5 Hz, Ph^4), 129.8 (d, *J* = 9.3 Hz, $Ph^{3,5}$), 128.9 (d, *J* = 9.8 Hz, $Ph^{2,6}$), 61.9 (d, *J* = 7.8 Hz, $N\underline{C}H_2CH_2P$), 56.0 (NCH_3), 55.3 (NCH_3), 26.6 (d, *J* = 18.8 Hz, $NCH_2\underline{C}H_2P$), $C_{q(CO)}$ not observed. $^{31}P\{^1H\}$ NMR (162 MHz, CD_2Cl_2) δ: = 59.9.

3.2 *fac*-[Mn($P^{Ph}N^{Et}$)$(CO)_3$Br] (1b)

L2 (153 mg, 0.536 mmol, 1.00 equiv.); $Mn(CO)_5Br$ (148 mg, 0.597 mmol, 1.11 equiv.); solvent: 5 mL toluene; precipitated and washed with MeOH; 63 mg (23 %) as orange powder. ^{1}H NMR (400 MHz, CD_2Cl_2) δ: = 7.75 – 7.66 (m, 2H, $Ph^{2,6}$), 7.63 – 7.56 (m, $2H^{2,6}$), 7.56 – 7.44 (m, 3H, $Ph^{3,4,5}$), 7.44 – 7.34 (m, 3H, $Ph^{3,4,5}$), 3.49 – 2.60 (m, 8H, $NC\underline{H_2}C\underline{H_2}P$, $NC\underline{H_2}CH_3$), 1.29 (t, *J* = 7.1 Hz, 3H, $NCH_2C\underline{H_3}$), 1.07 (t, *J* = 7.2 Hz, 3H, $NCH_2C\underline{H_3}$). $^{13}C\{^1H\}$ NMR (101 MHz, CD_2Cl_2) δ: = 132.5 (d, *J* = 8.7 Hz, $Ph^{3,5}$), 132.2 (d, *J* = 9.8 Hz, $Ph^{2,6}$), 131.0 (d, *J* = 2.1 Hz, Ph^4), 130.3 (d, *J* = 2.5 Hz, Ph^4), 129.4 (d, *J* = 9.3 Hz, $Ph^{3,5}$), 128.5 (d, *J* = 9.7 Hz, $Ph^{2,6}$), 56.0 (d, *J* = 8.1 Hz, $N\underline{C}H_2CH_2P$), 53.7 ($N\underline{C}H_2CH_3$), 51.4 ($N\underline{C}H_2CH_3$), 25.5 (d, *J* = 18.8 Hz, $NCH_2\underline{C}H_2P$), 11.0 ($NCH_2\underline{C}H_3$), 9.0 ($NCH_2\underline{C}H_3$). C_q not observed. $^{31}P\{^1H\}$ NMR (162 MHz, CD_2Cl_2) δ = 55.2. IR (ATR, cm^{-1}) = 2007 (CO), 1923 (CO), 1874 (CO). HRMS (ESI): *m/z* for $C_{21}H_{24}BrMnNO_3P$ $[M-Br]^+$ = 424.0869 (calc.), found: 424.0877.

3.3 *fac*-[Mn($P^{Ph}N^{Pyrr}$)$(CO)_3$Br] (1c)

L3 (152 mg, 0.536 mmol, 1.00 equiv.); $Mn(CO)_5Br$ (146 mg, 0.531 mmol, 1.00 equiv.); solvent: 5 mL toluene; precipitated and washed with *n*-pentane; 206 mg (77 %) as yellow powder. ^{1}H NMR (400 MHz, CD_2Cl_2) δ: = 8.13 – 6.99 (m, 10H, Ph), 4.01 – 1.41 (m, 12H, NCH_2CH_2P, pyrrolidine).$^{13}C\{^1H\}$ NMR (101 MHz, CD_2Cl_2) δ: = 134.6 (Ph^1), 133.1 (Ph^1), 132.6 (d, *J* = 8.7 Hz, $Ph^{3,5}$), 132.1 (d, *J* = 9.3 Hz, $Ph^{2,6}$), 131.0 (d, *J* = 2.7 Hz, Ph^4), 130.4 (d,f ff*J* = 2.7 Hz, Ph^4), 129.4 (d, *J* = 9.1 Hz, $Ph^{3,5}$), 128.6 (d, *J* = 9.5 Hz, $Ph^{2,6}$), 64.0 ($N\underline{C}H_2CH_2CH_2\underline{C}H_2$), 62.2 ($N\underline{C}H_2CH_2CH_2\underline{C}H_2$), 58.9 (d, J = 8.4 Hz, $N\underline{C}H_2CH_2P$), 26.7 (d, J = 19.1 Hz, $NCH_2\underline{C}H_2P$), 23.7 ($NCH_2\underline{C}H_2\underline{C}H_2CH_2$), 22.3 ($NCH_2\underline{C}H_2\underline{C}H_2CH_2$), $C_{q(CO)}$ not observed. $^{31}P\{^1H\}$ NMR (162 MHz, CD_2Cl_2) δ: =57.5. IR (ATR, cm^{-1}) = 2011 (CO), 1940 (CO), 1916 (CO). HRMS (ESI): *m/z* for $C_{21}H_{22}BrMnNO_3P$ $[M-Br]^+$ = 422.0712 (calc.), found: 422.0716.

3.4 *fac*-[Mn($P^{iPr}N^{Me}$)$(CO)_3$Br] (1d)

L4 (260 mg, 1.37 mmol, 1.00 equiv.); $Mn(CO)_5Br$ (376 mg, 1.37 mmol, 1.00 equiv.); solvent: 10 mL toluene; precipitated and washed with *n*-pentane; 432 mg (77 %) as yellow powder. ^{1}H NMR (250 MHz, CD_2Cl_3) δ: = 3.10 – 2.91 (m, 2H, $NC\underline{H_2}CH_2P$, $PC\underline{H}CH_3$), 2.84 (s, 3H, NCH_3), 2.71 (s, 3H, NCH_3), 2.57 – 2.39 (m, 1H, $PC\underline{H}CH_3$), 2.39 – 2.12 (m, 1H, $NC\underline{H_2}CH_2P$), 2.11 – 1.78 (m, 2H, $NCH_2C\underline{H_2}P$), 1.60 – 1.30 (m, 12H, $PCHC\underline{H_3}$). $^{13}C\{^1H\}$ NMR (101 MHz, CD_2Cl_2) δ: = 61.5 (d, *J* = 6.4 Hz, $N\underline{C}H_2CH_2P$), 55.2 (NCH_3), 54.8 (NCH_{3f}), 27.1 (d, *J* = 22.0 Hz, $P\underline{C}HCH_3$), 25.0 (d, *J* = 18.0 Hz, $P\underline{C}HCH_3$), 23.4 (d, *J* = 14.4 Hz, $NCH_2\underline{C}H_2P$), 20.3 ($PCH\underline{C}H_3$), 20.1 (d, *J* = 1.9 Hz, $PCH\underline{C}H_3$), 19.7 (d, *J* = 1.4 Hz, $PCH\underline{C}H_3$), 18.6 (d, *J* = 4.6 Hz, $PCH\underline{C}H_3$), C_q not observed. $^{31}P\{^1H\}$ NMR (162 MHz, CD_2Cl_2) δ: = 71.6 IR (ATR, cm^{-1}) = 2010 (CO), 1930 (CO), 1884 (CO). HRMS (ESI): *m/z* for $C_{13}H_{24}BrMnNO_3P$ $[M-Br]^+$ = 328.0869 (calc.), found: 328.0875.

3.5 *fac*-[Mn(P$^{i\text{Pr}}$N^{Et})(CO)$_3$Br] (1e)

L5 (290 mg, 1.33 mmol, 1.00 equiv.); $Mn(CO)_5Br$ (368 mg, 1.33 mmol, 1.00 equiv.); solvent: 15 mL toluene; precipitated and washed with *n*-pentane; 483 mg (83 %) as yellow powder.^{1}H NMR (400 MHz, C_6D_6) δ: = 3.43 – 3.29 (m, 1H, $NC\underline{H_2}CH_3$), 3.17 – 3.03 (dq, *J* = 14.0, 7.0 Hz, 1H, $NC\underline{H_2}CH_3$), 2.98 – 2.84 (m, 1H, $PC\underline{H}CH_3$), 2.66 – 2.49 (m, 2H, $NC\underline{H_2}CH_2P$, $NC\underline{H_2}CH_3$), 2.10 – 1.71 (m, 3H, $NC\underline{H_2}CH_2P$, $NC\underline{H_2}CH_3$, $PC\underline{H}CH_3$), 1.52 – 1.40 (m, 3H, $PCHC\underline{H_3}$), 1.40 – 1.22 (m, 1H, $NCH_2C\underline{H_2}P$), 1.19 – 0.95 (m, 10H, $NCH_2C\underline{H_2}P$, $PCHC\underline{H_3}$), 0.89 (t, *J* = 7.1 Hz, 3H, $NCH_2C\underline{H_3}$), 0.56 (t, *J* = 7.1 Hz, 3H, $NCH_2C\underline{H_3}$). $^{13}C\{^1H\}$ NMR (101 MHz, C_6D_6) δ: = 55.2 (d, *J* = 7.2 Hz, $N\underline{C}H_2CH_2P$), 52.7 ($N\underline{C}H_2CH_3$), 50.9 ($N\underline{C}H_2CH_3$), 27.1 (d, *J* = 21.4 Hz, $P\underline{C}HCH_3$), 24.7 (d, *J* = 18.3 Hz, $P\underline{C}HCH_3$), 22.6 (d, *J* = 14.1 Hz, $NCH_2\underline{C}H_2P$), 20.1 (d, *J* = 1.9 Hz, $PCH\underline{C}H_3$), 19.8 ($PCH\underline{C}H_3$), 19.4 (d, *J* = 1.4 Hz, $PCH\underline{C}H_3$), 18.5 (d, *J* = 4.4 Hz, $PCH\underline{C}H_3$), 10.9 ($NCH_2\underline{C}H_3$), 8.2 ($NCH_2\underline{C}H_3$), C_q not observed. $^{31}P\{^1H\}$ NMR (162 MHz, CD_2Cl_2) δ: = 65.8. IR (ATR, cm^{-1}) = 2992 ($\nu_{C\text{-}H}$), 2933 ($\nu_{C\text{-}H}$), 2875 ($\nu_{C\text{-}H}$) 2007 (CO), 1923 (CO), 1878 (CO). HRMS (ESI): *m/z* for $C_{15}H_{28}BrMnNO_3P$ $[M+Na]^+$ = 458.0262 (calc.), found: 458.0266.

3.6 *fac*-[Mn(P$^{i\text{Pr}}$N^{Pyrr})(CO)$_3$Br] (1f)

L6 (78.0 mg, 0.362 mmol, 1.00 equiv.); $Mn(CO)_5Br$ (99.6 mg, 0.362 mmol, 1.00 equiv.); solvent: 5 mL toluene; precipitated and washed with *n*-pentane; 99 mg (63 %) as yellow powder. ^{1}H NMR (400 MHz, C_6D_6) δ: = 4.00 – 3.82 (m, 1H, $NC\underline{H_2}CH_2CH_2C\underline{H_2}$), 2.96 – 2.67 (m, 3H, $NC\underline{H_2}CH_2P$, $NC\underline{H_2}CH_2CH_2C\underline{H_2}$, $PC\underline{H}CH_3$), 2.22 – 2.03 (m, 1H, $NC\underline{H_2}CH_2CH_2C\underline{H_2}$), 2.01 – 1.85 (m, 1H, $PC\underline{H}CH_3$), 1.87 – 1.69 (m, 3H, $NCH_2CH_2CH_2CH_2$,), 1.69 – 1.50 (m, 1H, $NC\underline{H_2}CH_2P$), 1.45 (dd, *J* = 15.6, 7.2 Hz, 3H, $PCHC\underline{H_3}$), 1.36 – 1.18 (m, 3H, $NCH_2C\underline{H_2}P$, $NCH_2C\underline{H_2}C\underline{H_2}CH_2$), 1.15 – 0.92f (m, 10H, $PCHC\underline{H_3}$, $NCH_2C\underline{H_2}C\underline{H_2}CH_2$). $^{13}C\{^1H\}$ NMR (101 MHz, C_6D_6) δ: = 62.9 ($N\underline{C}H_2CH_2CH_2\underline{C}H_2$), 61.5 ($N\underline{C}H_2CH_2CH_2\underline{C}H_2$), 58.8 (d, *J* = 7.3 Hz, $N\underline{C}H_2CH_2P$), 26.6 (d, *J* = 21.6 Hz, $P\underline{C}HCH_3$), 24.6 (d, *J* = 18.3 Hz, $P\underline{C}HCH_3$), 23.7 (d, *J* = 14.0 Hz, $NCH_2\underline{C}H_2P$), 23.4 ($NCH_2\underline{C}H_2\underline{C}H_2CH_2$), 22.0 ($NCH_2\underline{C}H_2\underline{C}H_2CH_2$), 20.0 (d, *J* = 2.1 Hz, $PCH\underline{C}H_3$), 20.0 ($PCH\underline{C}H_3$), 19.3 ($PCH\underline{C}H_3$), 18.4 (d, *J* = 4.6 Hz, $PCH\underline{C}H_3$), C_q not observed. $^{31}P\{^1H\}$ NMR (162 MHz, C_6D_6) δ: = 68.9. IR (ATR, cm^{-1}) = 2003 (CO), 1916 (CO), 1884 (CO). HRMS (ESI): *m/z* for $C_{15}H_{26}BrMnNO_3P$ $[M-Br]^+$ = 354.1025 (calc.), found: 354.1027.

3.7 *fac*-[Mn(P^{Cy}N^{Me})(CO)$_3$Br] (1g)

L7 (365 mg, 1.35 mmol, 1.33 equiv.); $Mn(CO)_5Br$ (281 mg, 1.02 mmol, 1.00 equiv.); solvent: 15 mL toluene; precipitated and washed with *n*-pentane; 398 mg (80 %) as yellow powder. ^{1}H NMR (400 MHz, C_6D_6) δ: = 2.81 – 2.65 (m, 1H, Cy^1), 2.64 – 2.53 (m, 2H, $Cy^{2\text{-}6}$, $NC\underline{H_2}CH_2P$), 2.50 (s, 3H, $NC\underline{H_3}$), 1.99 (s, 3H, $NC\underline{H_3}$), 1.89 – 1.80 (m, 3H, Cy), 1.78 – 1.25 (m, 13H, $NC\underline{H_2}C\underline{H_2}P$, $Cy^{2\text{-}6}$), 1.25 – 0.93 (m, 7H, $NCH_2C\underline{H_2}P$, $Cy^{2\text{-}6}$). $^{13}C\{^1H\}$ NMR (101 MHz, C_6D_6) δ: = 60.6 (d, *J* = 6.9 Hz, $N\underline{C}H_2CH_2P$), 54.2 (bs, NCH_3), 38.5 (d, *J* = 20.4 Hz, Cy^1), 35.3 (d, *J* = 16.7 Hz, Cy^1), 30.4 (d, *J* = 14.2 Hz, Cy), 29.5 (Cy), 29.0 (d, *J* = 5.2 Hz, Cy), 27.9 (d, *J* = 7.4 Hz, Cy), 27.9 – 27.7 (m, Cy), 27.6 (d, *J* = 9.8 Hz, Cy), 26.4 (d, *J* = 31.0 Hz, Cy), 21.0 (d, *J* = 14.2 Hz, $N\underline{C}H_2CH_2P$), C_q not observed. $^{31}P\{^1H\}$ NMR (162 MHz, C_6D_6) δ: = 63.5. IR (AfTR, cm^{-1}) = 2934 ($\nu_{C\text{-}H}$), 2857 ($\nu_{C\text{-}H}$), 2007 (CO), 1915 (CO), 1884 (CO). HRMS (ESI): *m/z* for $C_{19}H_{32}BrMnNO_3P$ $[M-Br]^+$ = 408.1494 (calc.), found: 408.1500.

3.8 *fac*-[Mn(P^{Cy}N^{Et})(CO)$_3$Br] (1h)

L8 (246 mg, 0.827 mmol, 1.00 equiv.); $Mn(CO)_5Br$ (227 mg, 0.826 mmol, 1.00 equiv.); solvent: 15 mL toluene; precipitated and washed with *n*-pentane; 340 mg (80 %) as yellow powder. ^{1}H NMR (400 MHz, C_6D_6) δ: = 3.45 – 3.30 (m, 1H, $NC\underline{H_2}CH_3$), 3.12 (dq, *J* = 14.3, 7.2 Hz, 1H, $NC\underline{H_2}CH_3$), 2.81 (bs, 1H, Cy^1), 2.73 – 2.47 (m, 3H, $NC\underline{H_2}CH_3$, $NC\underline{H_2}CH_2P$, Cy), 2.13 – 1.99 (m, 2H, $NC\underline{H_2}CH_3$, $NC\underline{H_2}CH_2P$), 1.98 – 1.85 (m, 2H, Cy), 1.85 – 1.33 (m, 14H, Cy, $NCH_2C\underline{H_2}P$), 1.30 – 1.01 (m, 6H, Cy), 0.91 (t, *J* = 7.1 Hz, 3H, $NCH_2C\underline{H_3}$), 0.57 (t, *J* = 7.1 Hz, 3H, $NCH_2C\underline{H_3}$). $^{13}C\{^1H\}$ NMR (101 MHz, C_6D_6) δ: = z55.2 (d, *J* = 7.1 Hz, $N\underline{C}H_2CH_2P$), 52.8 ($N\underline{C}H_2CH_3$), 50.9 ($N\underline{C}H_2CH_3$), 38.6 (d, *J* = 20.5 Hz, Cy^1), 35.3 (d, *J* = 16.9 Hz, Cy^1), 30.4 (d, *J* = 3.6 Hz, Cy), 29.6 (Cy), 29.1 (d, *J* = 5.3 Hz, Cy), 28.1 – 27.8 (m, Cy), 27.6 (d, *J* = 9.4 Hz, Cy), 26.6 (Cy), 26.3 (Cy), 20.7 (d, *J* = 14.3 Hz, $NCH_2\underline{C}H_2P$), 10.9 ($NCH_2\underline{C}H_3$), 8.2 ($NCH_2\underline{C}H_3$), C_q not observed. $^{31}P\{^1H\}$ NMR (162 MHz, C_6D_6) δ: = 57.8. IR (ATR, cm^{-1}) = IR (ATR, cm^{-1}) = 2991 ($\nu_{C\text{-}H}$), 2923 ($\nu_{C\text{-}H}$), 2848 ($\nu_{C\text{-}H}$), 2005 (CO), 1914 (CO), 1890 (CO). HRMS (ESI): *m/z* for $C_{21}H_{36}BrMnNO_3P$ $[M-Br]^+$ = 436.1807 (calc.), found: 436.1809.

3.9 *fac*-[Mn(P^{Cy}N^{Pyr})(CO)$_3$Br] (1i)

L9 (274 mg, 0.927 mmol, 1.00 equiv.); Mn(CO)$_5$Br (255 mg, 0.928 mmol, 1.00 equiv.); solvent: 10 mL toluene; precipitated and washed with *n*-pentane; 449 mg (94 %) as yellow powder. ^{1}H NMR (400 MHz, C_6D_6) δ: = 3.99 – 3.89 (m, 1H, NC$\underline{H}_2$CH$_2$CH$_2$C$\underline{H}_2$), 2.87 – 2.66 (m, 3H, NC$\underline{H}_2$CH$_2$CH$_2$C$\underline{H}_2$, NC$\underline{H}_2$CH$_2$P, Cy^1), 2.59 – 2.52 (m, 1H, Cy), 2.19 – 2.08 (m, 1H, NC$\underline{H}_2$CH$_2$CH$_2$C$\underline{H}_2$), 2.00 – 0.97 (m, 28H). ^{13}C{^{1}H} NMR (101 MHz, CD_2Cl_2) δ: = 63.6 (N$\underline{C}$H$_2$CH$_2$CH$_2$$\underline{C}H_2$), 62.0 (N$\underline{C}H_2CH_2CH_2$$\underline{C}H_2$), 59.0 (d, *J* = 7.1 Hz, N$\underline{C}$H$_2$CH$_2$P), 38.2 (d, *J* = 21.3 Hz, Cy^1), 35.1 (d, *J* = 16.8 Hz, Cy^1), 30.8 (Cy), 30.2 (Cy), 29.6 (Cy), 29.1 (d, *J* = 5.4 Hz, Cy), 28.1 – 27.5 (m, Cy), 26.7 (Cy), 26.4 (Cy), 23.7 (NCH$_2$$\underline{C}H_2$$\underline{C}H_2CH_2$), 22.4 (NCH$_2$$\underline{C}H_2$$\underline{C}H_2CH_2$), 22.2 (d, *J* = 15.2 Hz, NCH$_2$$\underline{C}H_2$P), C$_q$ not observed. ^{31}P{^{1}H} NMR (162 MHz, C_6D_6) δ: = 61.2. IR (ATR, cm^{-1}) = 2919 (ν_{C-H}), 2849 (ν_{C-H}), 2783 (ν_{C-H}), 2007 (CO), 1929 (CO), 1880 (CO). HRMS (ESI): *m/z* for $C_{21}H_{34}BrMnNO_3P$ [M−Br]$^+$ = 434.1651 (calc.), found: 434.1665.

4 Synthesis and Characterization of PN-supported Mn(I) Triflate Complexes

Scheme S5. Synthesis of Triflate Complexes 2.

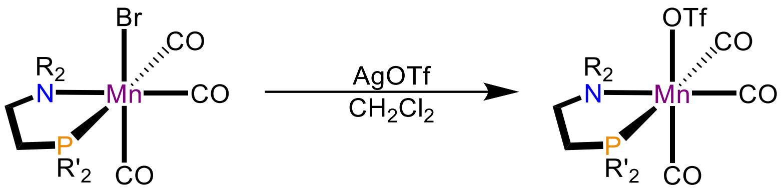

1a-1i R = Me, Et, Pyrrolidine R' = Ph, Cy, *i*Pr **2a-2i**

General Procedure:

In a Schlenk flask, covered with aluminum foil, the respective bromide complex **1** was dissolved in DCM and AgOTf was added. After stirring for 50 minutes at RT the reaction mixture was filtered over a pad of Celite as well as a syringe filter. This step was repeated until no dark precipitate in the solution was observed. The solvent was evaporated and the crude product was washed with *n*-pentane. The product was dried under vacuum, yielding a yellow or orange powder **2**.

4.1 *fac*-[Mn(P^{Ph}N^{Me})(CO)$_3$OTf] (2a)

1a (499 mg, 1.05 mmol, 1.00 equiv.); AgOTf (402 mg, 1.5 mmol, 1.49 equiv.); solvent: 15 mL DCM; washed with *n*-pentane (2 x 5 mL); 444 mg (78 %) as orange powder. ^{1}H NMR (400 MHz, CD_2Cl_2) δ: = 7.71 – 7.63 (m, 2H, Ph^{2,6}), 7.63 – 7.55 (m, 2H, Ph^{2,6}), 7.55 – 7.49 (m, 3H, Ph^{3,4,5}), 7.50 – 7.36 (m, 3H, Ph^{3,4,5}), 2.83 (s, 3H, NCH$_3$), 2.66 (s, 3H, NCH$_3$), 3.04 – 2.48 (m, 4H, PCH$_2$CH$_2$N). ^{13}C{^{1}H} NMR (101 MHz, CD2Cl2) δ: = 132.4 (d, J = 9.9 Hz, Ph^{2,3,5,6}), 132.1 (d, J = 9.9 Hz, Ph^{2,3,5,6}), 131.6 (Ph^4), 131.2 (Ph^4), 129.8 (d, J = 9.8 Hz, Ph^{2,3,5,6}), 129.5 (d, J = 9.6 Hz, Ph^{2,3,5,6}), 61.7 (d, J = 7.8 Hz, N$\underline{C}$H$_2$CH$_2$P), 55.4 (NCH$_3$), 26.3 (d, J = 19.2 Hz, NCH$_2$$\underline{C}H_2$P), C$_q$ not observed. ^{31}P{^{1}H} NMR (162 MHz, CD_2Cl_2) δ: = 60.3. ^{19}F{^{1}H} NMR (376 MHz, CD_2Cl_2) δ: = −77.9. IR (ATR, cm^{-1}) = 2026 (CO), 1940 (CO), 1907 (CO). HRMS (ESI): *m/z* for $C_{20}H_{20}F_3MnNO_6PS$ [M−OTf]$^+$ = 396.0561 (calc.), found: 396.0562.

4.2 *fac*-[Mn(P^{Ph}N^{Et})(CO)$_3$OTf] (2b)

1b (201 mg, 0.399 mmol, 1.00 equiv.); AgOTf (133 mg, 0.518 mmol, 1.30 equiv.); solvent: 5 mL DCM; washed with *n*-pentane (2 x 2 mL); 139 mg (61 %) as orange powder. ^{1}H NMR (400 MHz, CD_2Cl_2) δ: = 7.92 – 7.22 (m, 10H, Ph$^{2-6}$), 3.46 – 3.08 (m, 4H, NCH$_2$CH$_2$P, NC$\underline{H}_2$CH$_3$), 3.08 – 2.59 (m, 4H, , NCH$_2$CH$_2$P, NC$\underline{H}_2$CH$_3$), 1.48 – 1.33 (m, 3H, NCH$_2$C$\underline{H}_3$), 1.17 – 1.00i (m, 3H, NCH$_2$C$\underline{H}_3$). ^{13}C{^{1}H} NMR (101 MHz, CD_2Cl_2) δ: = 132.4 (d, *J* = 9.8 Hz, Ph^{2,3,5,6}), 132.1 (d, *J* = 9.7 Hz, Ph^{2,3,5,6}), 131.6 (Ph^4), 131.1 (Ph^4), 129.7 (d, *J* = 9.7 Hz, Ph^{2,3,5,6}), 129.4 (d, *J* = 9.6 Hz, Ph^{2,3,5,6}), 55.9 (d, *J* = 7.9 Hz, N$\underline{C}$H$_2$CH$_2$P), 53.4 (N$\underline{C}$H$_2$CH$_3$), 51.0f (N$\underline{C}$H$_2$CH$_3$), 25.8 (NCH$_2$$\underline{C}H_2$P), 11.2 (NCH$_2$$\underline{C}H_3$), 8.3 (NCH$_2$$\underline{C}H_3$), C$_q$ not observed. ^{31}P{^{1}H} NMR (162 MHz, CD_2Cl_2) δ: = 55.8. IR (ATR, cm^{-1}) = 2027 (CO), 1947 (CO), 1904 (CO). HRMS (ESI): *m/z* for $C_{22}H_{24}F_3MnNO_6PS$ [M−OTf]$^+$ = 424.0869 (calc.), found: 424.0879.

4.3 *fac*-[Mn($P^{Ph}N^{Pyrr}$)$(CO)_3$OTf] (2c)

1c (300 mg, 0.597 mmol, 1.00 equiv.); AgOTf (232 mg, 0.903 mmol, 1.51 equiv.); solvent: 7 mL DCM; washed with *n*-pentane (2 x 3 mL); 224 mg (66 %) as yellow powder. ^1H NMR (400 MHz, CD_2Cl_2) δ: = 7.6 – 7.58 (m, 4H, $Ph^{2,6}$), 7.54 – 7.36 (m, 6H, Ph^{3-5}), 3.8z4 – 3.25 (m, 2H), 3.18 – 2.42 (m, 6H), 2.42 – 1.70 (m, 4H). $^{13}C\{^1H\}$ NMR (101 MHz, CD_2Cl_2) δ: = 132.5 (d, *J* = 9.9 Hz, $Ph^{2,3,5,6}$), 132.1 (d, *J* = 9.9 Hz, $Ph^{2,3,5,6}$), 131.6 (d, *J* = 2.3 Hz, Ph^4), 131.2 (d, *J* = 2.3 Hz, Ph^4), 129.7 (d, *J* = 9.7 Hz, $Ph^{2,3,5,6}$), 129.4 (d, *J* = 9.8 Hz, $Ph^{2,3,5,6}$), 63.4 ($N\underline{C}H_2CH_2CH_2\underline{C}H_2$), 61.4 ($N\underline{C}H_2CH_2CH_2\underline{C}H_2$), 57.5 (d, *J* = 8.4 Hz, $N\underline{C}H_2CH_2P$), 26.8 (d, *J* = 19.5 Hz, $NCH_2\underline{C}H_2P$), 23.5 ($NCH_2\underline{C}H_2\underline{C}H_2CH_2$), 22.0 ($NCH_2\underline{C}H_2\underline{C}H_2CH_2$), C_q not observed. $^{31}P\{^1H\}$ NMR (162 MHz, CD_2Cl_2) δ: = 58.2. $^{19}F\{^1H\}$ NMR (376 MHz, CD_2Cl_2) δ: = -77.9. IR (ATR, cm^{-1}) = 2027 (CO), 1946 (CO), 1906 (CO). HRMS (ESI): *m/z* for $C_{22}H_{22}F_3MnNO_6PS$ [M−OTf]$^+$ = 422.0712 (calc.), found: 422.0712.

4.4 *fac*-[Mn($P^{iPr}N^{Me}$)$(CO)_3$OTf] (2d)

1d (432 mg, 1.06 mmol, 1.00 equiv.); AgOTf (400 mg, 1.55 mmol, 1.47 equiv.); solvent: 10 mL DCM; washed with *n*-pentane (2 x 3 mL); 301 mg (60 %) as orange sticky solid. ^1H NMR (400 MHz, CD_2Cl_2) δ: = 2.78 (s, 3H, NCH_3), 2.57 (s, 3H, NCH_3), 2.54 – 2.44 (m, 2H, $NC\underline{H_2}CH_2P$, $PC\underline{H}CH_3$), 2.44 – 2.31 (m, 2H, $NCH_2C\underline{H_2}P$, $NC\underline{H_2}CH_2P$), 2.12 – 1.97 (m, 1H, $NCH_2C\underline{H_2}P$), 1.60 – 1.16 (m, 12H, $PCHC\underline{H_3}$). $^{13}C\{^1H\}$ NMR (101 MHz, CD_2Cl_2) δ: = 61.8 ($N\underline{C}H_2CH_2P$), 55.2 (NCH_3), 53.0 (NCH_3), 26.2 (d, *J* = 23.7 Hz, $P\underline{C}HCH_3$), 23.7 (d, *J* = 15.9 Hz, $NCH_2\underline{C}H_2P$), 20.2 ($PCH\underline{C}H_3$), 19.6 ($PCH\underline{C}H_3$), 18.5 (d, *J* = 4.1 Hz, $PCH\underline{C}H_3$), C_q not observed. $^{31}P\{^1H\}$ NMR (162 MHz, CD_2Cl_2) δ: = 75.6. $^{19}F\{^1H\}$ NMR (376 MHz, CD_2Cl_2) δ: = −77.8. IR (ATR, cm^{-1}) = 2969 (ν_{C-H}), 2882 (ν_{C-H}), 2028 (CO), 1944 (CO), 1905 (CO). HRMS (ESI): *m/z* for $C_{14}H_{24}F_3MnNO_6PS$ [M−OTf]$^+$ = 328.0868 (calc.), found: 328.0876.

4.5 *fac*-[Mn($P^{iPr}N^{Et}$)$(CO)_3$OTf] (2e)

1e (212 mg, 0.486 mmol, 1.00 equiv.); AgOTf (186 mg, 0.724 mmol, 1.49 equiv.); solvent: 5 mL DCM; washed with *n*-pentane (2 x 5 mL); 142 (58 %) yellow powder. ^1H NMR (400 MHz, CD_2Cl_2) δ: = 3.27 – 3.16 (m, 1H, $NC\underline{H_2}CH_3$), 3.16 – 3.05 (m, 1H, $NC\underline{H_2}CH_3$), 2.89 – 2.66 (m, 3H, $NC\underline{H_2}CH_3$, $NC\underline{H_2}CH_2P$), 2.56 – 2.31 (m, 3H, $NC\underline{H_2}CH_2P$, $PC\underline{H}CH_3$), 2.20 – 1.94 (m, 2H, $NCH_2C\underline{H_2}P$), 1.52 – 1.32 (m, 15H, $PCHC\underline{H_3}$, $NCH_2C\underline{H_3}$), 1.06 (t, *J* = 7.2 Hz, 3H, $NCH_2C\underline{H_3}$). $^{13}C\{^1H\}$ NMR (101 MHz, CD_2Cl_2) δ: = 118.0 (d, J = 313.6 Hz, CF_3), 55.7 (d, *J* = 6.4 Hz, $N\underline{C}H_2CH_2P$), 53.2 ($N\underline{C}H_2CH_3$), 50.8 ($N\underline{C}H_2CH_3$), 26.5 (d, *J* = 23.9 Hz, $P\underline{C}HCH_3$), 23.5 (d, *J* = 15.3 Hz, $P\underline{C}HCH_3$), 23.0 (d, *J* = 15.3 Hz, $NCH_2\underline{C}H_2P$), 20.1 ($PCH\underline{C}H_3$), 19.8 – 19.5 (m, $PCH\underline{C}H_3$), 18.6 (d, *J* = 4.5 Hz, $PCH\underline{C}H_3$), 11.3 ($NCH_2\underline{C}H_3$), 8.1 ($NCH_2\underline{C}H_3$), C_q not observed. $^{31}P\{^1H\}$ NMR (162 MHz, CD_2Cl_2) δ: = 69.6. $^{19}F\{^1H\}$ NMR (376 MHz, CD_2Cl_2) δ: = −77.9. IR (ATR, cm^{-1}) = 2022 (CO), 1934 (CO), 1911 (CO). HRMS (ESI): *m/z* for $C_{16}H_{28}F_3MnNO_6PS$ [M−OTf]$^+$ = 356.1181 (calc.), found: 356.1187.

4.6 *fac*-[Mn($P^{iPr}N^{Pyrr}$)$(CO)_3$OTf] (2f)

1f (91.7 mg, 0.211 mmol, 1.00 equiv.); AgOTf (71.3 mg, 0.277 mmol, 1.31 equiv.); solvent: 5 mL DCM; washed with *n*-pentane (2 x 5 mL); 73.5 mg (69 %) yellow powder. ^1H NMR (400 MHz, C_6D_6) δ: = 3.40 (bs, 1H, $NC\underline{H_2}CH_2CH_2C\underline{H_2}$), 2.69 (bs, 1H, $NC\underline{H_2}CH_2CH_2C\underline{H_2}$), 2.37 – 1.97 (m, 3H, $NC\underline{H_2}CH_2CH_2C\underline{H_2}$, $NC\underline{H_2}CH_2P$, $PC\underline{H}CH_3$), 1.97 – 1.76 (m, 2H, $NC\underline{H_2}CH_2CH_2C\underline{H_2}$, $PC\underline{H}CH_3$), 1.75 – 1.61 (s, 1H, $NCH_2C\underline{H_2}C\underline{H_2}CH_2$), 1.60 – 1.48 (m, 1H, $NC\underline{H_2}CH_2P$), 1.42 – 1.12 (m, 6H, $PCHC\underline{H_3}$, $NCH_2C\underline{H_2}C\underline{H_2}CH_2$, $NCH_2C\underline{H_2}P$), 1.04 – 0.85 (m, 11H, $PCHC\underline{H_3}$, $NCH_2C\underline{H_2}C\underline{H_2}CH_2$, $NCH_2C\underline{H_2}P$). $^{13}C\{^1H\}$ NMR (101 MHz, C_6D_6) δ 120.4 (d, *J* = 319.0 Hz, CF_3), 62.3 ($N\underline{C}H_2CH_2CH_2\underline{C}H_2$), 60.9 ($N\underline{C}H_2CH_2CH_2\underline{C}H_2$), 56.9 (d, *J* = 6.6 Hz, $N\underline{C}H_2CH_2P$), 25.7 (d, *J* = 23.5 Hz, $P\underline{C}HCH_3$), 23.6 (d, *J* = 15.0 Hz, $NCH_2\underline{C}H_2P$), 23.0 ($NCH_2\underline{C}H_2\underline{C}H_2CH_2$), 23.0 (d, *J* = 15.0 Hz, $P\underline{C}HCH_3$), 21.5 ($NCH_2\underline{C}H_2\underline{C}H_2CH_2$), 19.7 ($PCH\underline{C}H_3$), 19.5 ($PCH\underline{C}H_3$), 19.2 ($PCH\underline{C}H_3$), 18.2 (d, *J* = 3.9 Hz, ($PCH\underline{C}H_3$), C_q not observed. $^{31}P\{^1H\}$ NMR (162 MHz, C_6D_6) δ: = 72.6. $^{19}F\{^1H\}$ NMR (376 MHz, CD_2Cl_2) δ: = −77. IR (ATR, cm^{-1}) = 2963 (ν_{C-H}), 2925 (ν_{C-H}), 2884 (ν_{C-H}), 2022 (CO), 1946 (CO), 1913 (CO). HRMS (ESI): *m/z* for $C_{16}H_{26}F_3MnNO_6PS$ [M−OTf]$^+$ = 354.1025 (calc.), found: 354.1033.

4.7 *fac*-[Mn($P^{Cy}N^{Me}$)$(CO)_3$OTf] (2g)

1g (300 mg, 0.614 mmol, 1.00 equiv.) AgOTf (238 mg, 0.926 mmol, 1.51 equiv.); solvent: 5 mL DCM; washed with *n*-pentane (2 x 5 mL); 283 mg (86 %) as yellow powder. ^1H NMR (400 MHz, CD_2Cl_2) δ: = 2.78 (s, 3H, NCH_3), 2.55 (s, 3H, NCH_3), 2.53 – 2.33 (m, 2H, $NC\underline{H_2}CH_2P$), 2.23 – 1.17f (m, 24H).

$^{13}C\{^1H\}$ NMR (101 MHz, CD_2Cl_2) δ: = 121.2 (CF_3), 61.8 (d, *J* = 6.5 Hz, N<u>C</u>H_2CH_2P), 55.3 (NCH_3), 52.8 (NCH_3), 37.5 (d, *J* = 22.1 Hz, Cy^1), 34.0 (d, *J* = 13.9 Hz, Cy^1), 30.7 (Cy), 29.8 (Cy), 29.7 (Cy), 29.1 (d, *J* = 5.2 Hz, Cy), 28.0 (Cy), 27.8 (Cy), 27.8 (Cy), 27.7 (Cy), 27.7 (Cy), 27.6 (Cy), 27.6 (Cy), 27.5 (Cy), 26.3 (d, *J* = 5.0 Hz, Cy), 21.8 (d, *J* = 15.5 Hz, $NCH_2\underline{C}H_2P$), C_q not observed.^{31}P NMR (162 MHz, CD_2Cl_2) δ: = 67.7. ^{19}F NMR (376 MHz, CD_2Cl_2) δ: = -77.5.IR (ATR, cm^{-1}) = = 2929 ($\nu_{C\text{-}H}$), 2854 ($\nu_{C\text{-}H}$), 2031 (CO), 1948 (CO), 1902 (CO). HRMS (ESI): *m/z* for $C_{20}H_{32}F_3MnNO_6PS$ $[M{-}OTf]^+$ = 408.1494 (calc.), found: 408.1501.

4.8 *fac*-[Mn($P^{Cy}N^{Et}$)$(CO)_3$OTf] (2h)

[Mn($P^{Cy}N^{Et}$-Et)$(CO)_3$Br] **(1h)** (179 mg, 0.347 mmol, 1.00 equiv.) AgOTf (134 mg, 0.522 mmol, 1.50 equiv.); solvent: 6 mL DCM; washed with *n*-pentane (2 x 5 mL); 167 mg (82 %) as yellow powder. ^1H NMR (400 MHz, C_6D_6) δ: = 3.0ii4 – 2.92 (m, 1H, $N\underline{CH_2}CH_3$), 2.75 – 2.52 (m, 2H, $N\underline{CH_2}CH_3$), 2.39 (s, 1H, $Cy^{2\text{-}6}$), 2.27 – 2.08 (m, 1H, Cy^1), 2.05 – 1.89 (m, 3H, $N\underline{CH_2}CH_2P$, $N\underline{CH_2}CH_3$), 1.88 – 1.68 (m, 5H, $Cy^{1\text{-}6}$,), 1.67 – 1.55 (m, 5H, $Cy^{2\text{-}6}$), 1.55 – 1.31 (m, 6H, Cy, $NCH_2\underline{CH_2}P$, $Cy_2^{2\text{-}6}$), 1.30 – 0.91 (m, 6H, $Cy^{2\text{-}6}$, $NCH_2\underline{CH_2}P$), 0.86 (t, *J* = 7.1 Hz, 3H, $NCH_2\underline{CH_3}$), 0.59 (t, *J* = 7.1 Hz, 3H, $NCH_2\underline{CH_3}$). $^{13}C\{^1H\}$ NMR (101 MHz, C_6D_6) δ: = 120.4 (d, *J* = 319.6 Hz, CF_3), 55.1 (d, *J* = 6.9 Hz, $N\underline{C}H_2CH_2P$), 52gff.7($N\underline{C}H_2CH_3$), 50.3 ($N\underline{C}H_2CH_3$), 37.8 (d, *J* = 22.2 Hz, Cy^1), 33.6 (d, *J* = 13.4 Hz, Cy^1), 30.4 (d, *J* = 2.4 Hz, $Cy^{2\text{-}6}$), 29.7 ($Cy^{2\text{-}6}$), 29.0 (d, *J* = 5.5 Hz, $Cy^{2\text{-}6}$), 27.8 ($Cy^{2\text{-}6}$), 27.7 (d, *J* = 2.8 Hz, $Cy^{2\text{-}6}$), 27.6 (d, *J* = 5.0 Hz), 27.4 (d, *J* = 2.4 Hz), 26.2 (Cy), 26.1 (Cy) 20.6 (d, *J* = 14.8 Hz, $NCH_2\underline{C}H_2P$), 10.6 ($NCH_2\underline{C}H_3$), 7.7 ($NCH_2\underline{C}H_3$), C_q not observed. ^{31}P NMR (162 MHz, C_6D_6) δ:= 61.5. ^{19}F NMR (235 MHz, CD_2Cl_2) δ: = -75.8. IR (ATR, cm^{-1}) = 2935 ($\nu_{C\text{-}H}$), 2857 ($\nu_{C\text{-}H}$), 2025 (CO), 1941 (CO), 1903 (CO). HRMS (ESI): *m/z* for $C_{22}H_{36}F_3MnNO_6PS$ $[M{-}OTf]^+$ = 436.1807 (calc.), found: 436.1809

4.9 *fac*-[Mn($P^{Cy}N^{Pyrr}$)$(CO)_3$OTf] (2i)

1i (60.0 mg, 0117 mmol, 1.00 equiv.) AgOTf (45.0 mg, 0.175 mmol, 1.50 equiv.); solvent: 5 mL DCM; washed with *n*-pentane (4 x 3 mL); 53.3 mg (78 %) as yellow powder. ^1H NMR (400 MHz, C_6D_6) δ: = 3.47 – 3.30 (m, 1H, $N\underline{CH_2}CH_2CH_2\underline{CH_2}$), 2.81 – 2.66 (m, 1H, $N\underline{CH_2}CH_2CH_2\underline{CH_2}$), 2.37 – 2.18 (m, 2H, Cy, $N\underline{CH_2}CH_2CH_2\underline{CH_2}$), 2.19 – 2.01 (m, 2H, Cy^1, $N\underline{CH_2}CH_2P$), 2.03 – 1.88 (m, 2H, $N\underline{CH_2}CH_2CH_2\underline{CH_2}$), 1.88 – 0.60z (m, 26H, $N\underline{CH_2}\underline{CH_2}P$, Cy, $NCH_2\underline{CH_2}\underline{CH_2}CH_2$). $^{13}C\{^1H\}$ NMR (101 MHz, CD_2Cl_2) δ: = 119.7 (d, *J* = 318.7 Hz, CF_3), 63.2 (s, $N\underline{C}H_2CH_2CH_2\underline{C}H_2$), 61.0 (s, $N\underline{C}H_2CH_2CH_2\underline{C}H_2$), 57.6 (d, *J* = 6.8 Hz, $N\underline{C}H_2CH_2P$), 37.5 (d, *J* = 22.5 Hz, Cy^1), 33.7 (d, *J* = 13.7 Hz, Cy^1), 30.7 (d, *J* = 1.9 Hz, Cy), 29.8 (Cy), 29.6 (Cy), 29.1 (d, *J* = 5.2 Hz, Cy), 28.0 (Cy), 27.8 (d, *J* = 3.3 Hz, Cy), 27.7 (Cy), 27.7 (Cy), 27.6 (Cy), 27.6 (Cy), 27.5 (Cy), 26.3 (d, *J* = 4.0 Hz, Cy), 23.4 (s, $NCH_2\underline{C}H_2\underline{C}H_2CH_2$) , 22.4 (d, *J* = 15.8 Hz, $NCH_2\underline{C}H_2P$), 22.1 (s, $NCH_2\underline{C}H_2\underline{C}H_2CH_2$). C_q not observed. ^{31}P NMR (162 MHz, CD_2Cl_2) δ: = 64.9. $^{19}F\{^1H\}$ NMR (376 MHz, C_6D_6) δ: = -76.7. IR (ATR, cm^{-1}) = 2928 ($\nu_{C\text{-}H}$), 2854 ($\nu_{C\text{-}H}$), 2027 (CO), 1946 (CO), 1911 (CO). HRMS (ESI): *m/z* for $C_{22}H_{34}F_3MnNO_6PS$ $[M{-}OTf]^+$ = 434.1651 (calc.), found: 434.1667.

5 Synthesis and Characterization of PN-supported Mn(I) Alkyl Complexes

Yields, $^{31}P\{^1H\}$ shifts and carbonyl frequencies for PN1-PN8 are summarized in Table S1.

Table S1. Yields, $^{31}P\{^1H\}$ shifts and carbonyl frequencies for PN1-PN8

PN-based Mn(I) Alkyl Complexes		Yield (%)	$^{31}P\{^1H\}$ [ppm]	IR CO frequencies [cm^{-1}]		
**PN1**	[Mn($P^{Cy}N^{Me}$-Et)$(CO)_3$Me]	64	[a]71.3	1976	1877	1837
**PN2**	[Mn($P^{iPr}N^{Me}$-Et)$(CO)_3$Me]	15	[a]80.0	1975	1877	1850
**PN3**	[Mn($P^{iPr}N^{Pyrr}$-Et)$(CO)_3$Me]	48	[b]77.8	1969	1889	1851
**PN4**	[Mn($P^{Cy}N^{Pyrr}$-Et)$(CO)_3$Me]	51	[b]69.2	1973	1877	1832
**PN5**	[Mn($P^{Ph}N^{Me}$-Et)$(CO)_3$Me]	28	[a]72.1	1982	1890	1851
**PN6**	[Mn($P^{Cy}N^{Et}$-Et)$(CO)_3$Me]	38	[b]65.4	1972	1874	1856
**PN7**	[Mn($P^{Ph}N^{Pyrr}$-Et)$(CO)_3$Me]	28	[a]70.0	1982	1889	1855
**PN8**	[Mn($P^{Ph}N^{Et}$-Et)$(CO)_3$Me]	26	[a]67.3	1983	1891	1858

$^{31}P\{^1H\}$-NMR spectra measured in [a]CD_2Cl_2, [b]C_6D_6.

5.1 *fac*-$[Mn(P^{Cy}N^{Me})(CO)_3CH_3]$ (PN1)

In a Schlenk flask, the triflate complex **2g** (88.0 mg, 0.158 mmol, 1.00 equiv.) was suspended in Et_2O (5 mL), cooled to -70 °C and subsequently MeLi (0.20 mL, 1.6 M in Et_2O, 0.32 mmol, 2.03 equiv.) was added dropwise. During addition, the yellow suspension turned brown. After 50 minutes, the solution mixture was allowed to reach RT and stirred for 1.5 hours. The solvent was evaporated, the remained residue was extracted with *n*-pentane (3 x 5 mL) and filtered *via* a syringe filter. After evaporation of the solvent, the solid was digested in *n*-pentane (3 mL) for 10 minutes. Upon drying of the precipitate, **PN1** was obtained as pale yellow powder (43.0 mg, 64 %). ^1H NMR (400 MHz, CD_2Cl_2) δ: = 2.64 (s, 3H, NCH_3), 2.24 (s, 3H, NCH_3), 2.38 – 2.01 (m, 4H, $NC\underline{H_2}CH_2P$, Cy^1), 1.99 – 1.79 (m, 7H, $NCH_2C\underline{H_2}P$, Cy), 1.79 – 1.53 (m, 7H, $NCH_2C\underline{H_2}P$, Cy), 1.46 – 1.24 (m, 8H, Cy), -0.46 (d, J = 8.2 Hz, 3H, Mn-CH_3). $^{13}C\{^1H\}$ NMR (101 MHz, CD_2Cl_2) δ: = 61.9 (d, J = 7.9 Hz, $N\underline{C}H_2CH_2P$), 57.9 (NCH_3), 52.0 (NCH_3), 38.2 (d, J = 19.3 Hz, Cy^1), 34.3 (d, J = 15.8 Hz, Cy^1), 31.0 (Cy), 30.5 (d, J = 2.8 Hz, Cy), 29.1(Cy), 28.8 (d, J = 5.5 Hz, Cy), 28.3 (Cy), 28.1 (Cy), 28.1 (Cy), 28.0 (Cy), 27.9 (Cy), 27.9 (Cy), 27.7 (Cy), 27.6 (Cy), 26.9 (Cy), 26.6 (Cy), 22.5 (d, J = 12.7 Hz, $NCH_2\underline{C}H_2P$), -4.2 (d, J = 18.4 Hz, Mn-CH_3), C_q not observed. $^{31}P\{^1H\}$ NMR (162 MHz, CD_2Cl_2) δ: = 71.3. IR (ATR, cm^{-1}) = 2924 (ν_{C-H}), 2852 (ν_{C-H}), 1976 (CO), 1877 (CO), 1837 (CO). HRMS (ESI): *m/z* for $C_{20}H_{35}MnNO_3P$ $[M+Na]^+$ = 446.1627 (calc.), found: 446.1629.

5.2 *fac*-$[Mn(P^{iPr}N^{Me})(CO)_3CH_3]$ (PN2)

In a Schlenk flask, the triflate complex **2d** (192 mg, 0.402 mmol, 1.00 equiv.) was suspended in Et_2O (10 mL), cooled to -70 °C followed by a dropwise addition of MeLi (0.38 mL, 1.6 M in Et_2O, 0.608 mmol, 1.51 equiv.). Stirring was continued for 1 hour and afterwards the suspension was allowed to reach RT, upon which the colour changed to red. During the reaction progress, the solution mixture turned brown. After 2 hours the solvent was evaporated, the residue extracted with *n*-pentane (5 x 5 mL) and filtered *via* a syringe filter. The solvent was evaporated, yielding **PN2** as a yellow powder (22.0 mg, 15 %). ^1H NMR (400 MHz, CD_2Cl_2) δ: = 2.65 (s, 3H, NCH_3), 2.52– 2.12 (m, 4H, $PC\underline{H}CH_3$, $NC\underline{H_2}CH_2P$), 2.24 (s, 3H, NCH_3), 1.95 – 1.86 (m, 1H, $NCH_2C\underline{H_2}P$), 1.79 – 1.70 (m, 1H, $NCH_2C\underline{H_2}P$), 1.52 – 1.13 (m, 12H, $PCHC\underline{H_3}$), -0.44 (d, J = 8.3 Hz, 3H, Mn-CH_3). $^{13}C\{^1H\}$ NMR (101 MHz, C_6D_6) δ: = 61.4 (d, J = 8.1 Hz, $N\underline{C}H_2CH_2P$), 57.2 (NCH_3), 51.5 (NCH_3), 26.1 (d, J = 19.8 Hz, $P\underline{C}HCH_3$), 23.9 (d, J = 11.5 Hz, $NCH_2\underline{C}H_2P$), 23.2 (d, J = 16.6 Hz, $P\underline{C}HCH_3$), 20.5 ($PCH\underline{C}H_3$), 20.0 – 19.5 (m, $PCH\underline{C}H_3$), 19.2 ($PCH\underline{C}H_3$), 17.9 (d, J = 5.1 Hz, $PCH\underline{C}H_3$), -3.8 (d, J = 18.6 Hz, Mn-CH_3). $^{31}P\{^1H\}$ NMR (162 MHz, CD_2Cl_2) δ: = 80.0. IR (ATR, cm^{-1}) = 2923 (ν_{C-H}), 2854 (ν_{C-H}), 1975 (CO), 1877 (CO), 1850 (CO). HRMS (ESI): *m/z* for $C_{14}H_{27}MnNO_3P$ $[M+Na]^+$ = 446.1627 (calc.), found: 446.1633.

5.3 *fac*-$[Mn(P^{iPr}N^{Pyrr})(CO)_3CH_3]$ (PN3)

In a Schlenk flask, the triflate complex **2f** (166 mg, 0.330 mmol, 1.00 equiv.) was suspended in Et_2O (10 mL), cooled to -70 °C followed by a dropwise addition of MeLi (0.42 mL, 1.6 M in Et_2O, 0.672 mmol, 2.04 equiv.). Stirring was continued for 1 hour and afterwards the suspension was allowed to reach RT, upon which the colour changed to brown. After 2 hours the solvent was evaporated, the residue extracted with toluene (3 x 5 mL) and filtered *via* a syringe filter. The solvent was evaporated and thfe residue was washed with *n*-pentane (2 x 1 mL), yielding **PN3** as a yellow powder (58.2 mg, 48 %). ^1H NMR (400 MHz, CD_2Cl_2) δ: = 3.32 – 3.19 (m, 1H, $NC\underline{H_2}CH_2CH_2C\underline{H_2}$), 3.02 – 2.93 (m, 1H, $NC\underline{H_2}CH_2CH_2C\underline{H_2}$), 2.70 – 2.29 (m, 5H, $NC\underline{H_2}CH_2CH_2C\underline{H_2}$, $NC\underline{H_2}CH_2P$, $PC\underline{H}CH_3$), 2.29 – 1.66 (m, 7H, NCH_2CH_2P, $NCH_2C\underline{H_2}C\underline{H_2}CH_2$), 1.55 – 1.06 (m, 12H, $PCHC\underline{H_3}$), -0.43 (d, J = 8.2 Hz, 3H). $^{13}C\{^1H\}$ NMR (101 MHz, C_6D_6) δ: = 65.4 ($N\underline{C}H_2CH_2CH_2\underline{C}H_2$), 59.0 ($N\underline{C}H_2CH_2CH_2\underline{C}H_2$), 57.7 (d, J = 8.6 Hz, $N\underline{C}H_2CH_2P$), 26.1 (d, J = 19.8 Hz, $P\underline{C}HCH_3$), 24.4 (d, J = 11.4 Hz, $NCH_2\underline{C}H_2P$), 23.0 (d, J = 16.9 Hz, $P\underline{C}HCH_3$), 23.0 ($N\underline{C}H_2CH_2CH_2\underline{C}H_2$), 22.0 ($N\underline{C}H_2CH_2CH_2\underline{C}H_2$), 20.5 (d, J = 2.8 Hz, $PCH\underline{C}H_3$), 19.8 (d, J = 3.5 Hz, $PCH\underline{C}H_3$), 19.1 ($PCH\underline{C}H_3$), 18.0 (d, J = 5.2 Hz, $PCH\underline{C}H_3$), -4.1 (d, J = 18.3 Hz, Mn-CH_3), C_q not observed. $^{31}P\{^1H\}$ NMR (162 MHz, C_6D_6) δ: = 77.8. IR (ATR, cm^{-1}) = 2960 (ν_{C-H}), 2869 (ν_{C-H}), 1969 (CO), 1889 (CO), 1851 (CO). HRMS (ESI): *m/z* for $C_{16}H_{29}MnNO_3P$ $[M+Na]^+$ = 392.1157 (calc.), found: 392.1159.

5.4 *fac*-[Mn($P^{Cy}N^{Pyrr}$)$(CO)_3CH_3$] (PN4)

In a Schlenk flask, the triflate complex **2i** (106 mg, 0.181 mmol, 1.00 equiv.) was suspended in Et_2O (8 mL) followed by the addition of MeLi (0.37 mL, 1.6 M in Et_2O, 0.592 mmol, 3.26 equiv.) over a time period of 2.5 hours. During the reaction, the color changed from yellow to green, then red, and finally brown. After another hour the solvent was evaporated, the residue extracted with toluene (3 x 5 mL) and filtered *via* a syringe filter. The solvent was evaporated and the residue was washed with *n*-pentane (5 mL), yielding **PN4** as a yellow powder (34.5 mg, 51 %). ^1H NMR (400 MHz, C_6D_6) δ: = 3.09 – 2.97 (m, 1H, $NC\underline{H_2}CH_2CH_2C\underline{H_2}$), 2.85 – 2.71 (m, 1H, $NC\underline{H_2}CH_2CH_2C\underline{H_2}$), 2.37 – 2.24 (m, 1H, Cy), 2.15 – 1.97 (m, 2H, Cy^1, $NC\underline{H_2}CH_2CH_2C\underline{H_2}$), 1.97 – 1.52 (m, 24H, Cy, $NC\underline{H_2}C\underline{H_2}P$), 1.50 – 1.00 (m, 22H, Cy, $NCH_2C\underline{H_2}C\underline{H_2}CH_2$), -0.01 (d, J = 8.3 Hz, 3H, Mn-CH_3). $^{13}C\{^1H\}$ NMR (101 MHz, C_6D_6) δ: = 65.4 ($N\underline{C}H_2CH_2CH_2\underline{C}H_2$), 58.9 ($N\underline{C}H_2CH_2CH_2\underline{C}H_2$), 57.6 (d, J = 8.9 Hz, $N\underline{C}H_2CH_2P$), 38.0 (d, J = 19.0 Hz, Cy^1), 34.2 (d, J = 15.5 Hz, Cy^1), 31.0 (Cy), 30.4 (d, J = 2.8 Hz, Cy), 28.9 (Cy), 28.7 (d, J = 5.5 Hz, Cy), 28.1 (Cy), 28.1 (Cy), 28.0 (Cy), 28.0 (Cy), 27.9 (Cy), 27.8 (Cy), 27.7 (Cy), 27.6 (Cy), 26.7 (Cy), 26.4 (Cy), 23.0 ($NCH_2\underline{C}H_2\underline{C}H_2CH_2$) 22.6 (d, J = 12.2 Hz, $NCH_2\underline{C}H_2P$), 22.0 ($NCH_2\underline{C}H_2\underline{C}H_2CH_2$), -3.8 (d, J = 18.3 Hz, Mn-CH_3), C_q not observed. $^{31}P\{^1H\}$ NMR (162 MHz, C_6D_6) δ: = 69.2. IR (ATR, cm^{-1}) = 2924 ν(ν_{C-H}), 2853 ν(ν_{C-H}), 1973 (CO), 1877 (CO), 1832 (CO). HRMS (ESI): *m/z* for $C_{22}H_{37}MnNO_3P$ $[M+Na]^+$ = 472.1784 (calc.), found: 472.1790.

5.5 *fac*-[Mn($P^{Ph}N^{Me}$)$(CO)_3CH_3$] (PN5)

In a Schlenk flask, triflate complex **2a** (107 mg, 0.196 mmol, 1.00 equiv.) was suspended in 1,4-dioxane (6 mL) and MeMgCl (0.13 mL, 3.0 M in THF, 0.390 mmol, 1.98 equiv.) was added dropwise, upon which the yellow suspension changed to brown. After stirring for 2 hours and 15 minutes the solvent was evaporated under reduced pressure. The reaction mixture was extracted with toluene (3 x 5 mL) and filtered via syringe filter. After evaporation of the solvent and drying, a yellow residue remained. The residue was washed with *n*-pentane (1.5 mL), yielding **PN5** as a pale yellow powder (22.7 mg, 28 %).^1H NMR (400 MHz, CD_2Cl_2) δ: = 7.80 – 7.66 (m, 2H, $Ph^{2,6}$), 7.52 – 29 (m, 8H, Ph^{2-6}), 2.88 – 2.69 (m, 2H, PCH_2CH_2N), 2.66 (s, 3H, NCH_3), 2.59 – 2.39 (m, 2H, PCH_2CH_2N), 2.34 (s, 3H, NCH_3), -0.78 (d, J = 10.3 Hz, 3H, Mn-CH_3). $^{13}C\{^1H\}$ NMR (101 MHz, CD_2Cl_2) δ: = 137.0 (d, J = 38.4 Hz, Ph^1), 133.7 (d, J = 36.5 Hz, Ph^1), 132.5 (d, J = 10.8 Hz, $Ph^{2,6}$), 131.4 (d, J = 9.2 Hz, $Ph^{3,5}$), 130.5 (d, J = 2.0 Hz, Ph^4), 129.7 (d, J = 2.3 Hz, Ph^4), 129.1 (d, J = 9.1 Hz, $Ph^{2,6}$), 128.8 (d, J = 9.0 Hz, $Ph^{3,5}$), 61.2 (d, J = 9.0 Hz, $N\underline{C}H_2CH_2P$), 57.9 (NCH_3), 52.8 (NCH_3), 26.5 (d, J = 17.1 Hz, $NCH_2\underline{C}H_2P$), -1.1 (d, J = 17.8 Hz, Mn-CH_3),), $C_{q(CO)}$ not observed. $^{31}P\{^1H\}$ NMR (162 MHz, CD_2Cl_2) δ: = 72.1. IR (ATR, cm^{-1}) = 1982 (CO), 1890 (CO), 1851 (CO), HRMS (ESI):): *m/z* for $C_{20}H_{23}MnNO_3P$ $[M+Na]^+$ = 434.0688 (calc.), found: 434.0688.

5.6 *fac*-[Mn($P^{Cy}N^{Et}$)$(CO)_3CH_3$] (PN6)

In a Schlenk flask, the triflate complex **2h** (152 mg, 0.260 mmol, 1.00 equiv.) was suspended in Et_2O (7 mL), cooled to -80 °C followed by a dropwise addition of MeLi (0.32 mL, 1.6 M in Et_2O, 0.512 mmol, 1.97 equiv.). Stirring was continued for 1 hour and afterwards the suspension was allowed to reach RT, upon which the color changed to brown. After 2 hours the solvent was evaporated, the residue extracted with toluene (3 x 5 mL) and filtered *via* a syringe filter. The solvent was evaporated and the residue was washed with *n*-pentane (2 x 3 mL), yielding **PN6** as a yellow powder (44.7 mg, 38%). ^1H NMR (400 MHz, C_6D_6) δ: = 2.83 (dq, J = 14.2, 7.1 Hz, 1H, $NC\underline{H_2}CH_3$), 2.65 – 2.53 (m, 1H, $NC\underline{H_2}CH_3$), 2.34 – 2.18 (m, 2H, $NC\underline{H_2}CH_3$, Cy), 2.13 – 2.02 (m, 2H, $NC\underline{H_2}CH_3$, Cy^1), 2.00i – 1.50 (m, 13H, $NC\underline{H_2}CH_2P$, Cy), 1.50 – 0.99 (m, 11H, $NCH_2C\underline{H_2}P$, Cy), 0.92 (t, J = 7.0 Hz, 3H, $NCH_2C\underline{H_3}$), 0.55 (t, J = 7.2 Hz, 3H, $NCH_2C\underline{H_3}$), 0.03 (d, J = 8.2 Hz, 3H, Mn-CH_3), $^{13}C\{^1H\}$ NMR (101 MHz, C_6D_6) δ: = 55.7 (d, J = 8.6 Hz, $N\underline{C}H_2CH_2P$), 54.8 ($N\underline{C}H_2CH_3$), 49.0 ($N\underline{C}H_2CH_3$), 38.6 (d, J = 19.5 Hz, Cy^1), 34.1 (d, J = 15.0zii Hz, Cy^1), 31.0 (Cy), 30.5 (d, J = 2.5 Hz, Cy), 29.2 (Cy), 28.7 (d, J = 5.5 Hz, Cy), 28.1 (d, J = 2.7 Hz, Cy), 28.0 (d, J = 4.1 Hz, Cy), 27.9 (Cy), 27.8 (Cy), 27.7 (Cy), 27.6 (Cy), 26.7 (Cy), 26.4 (Cy), 21.5 (d, J = 12.1 Hz, $NCH_2\underline{C}H_2P$), 10.2 ($NCH_2\underline{C}H_3$), 8.4 ($NCH_2\underline{C}H_3$), -3.4 (d, J = 18.3 Hz, Mn-CH_3), C_q not observed. $^{31}P\{^1H\}$ NMR (162 MHz, C_6D_6) δ: = 65.4. IR (ATR, cm^{-1}) = 2924 (ν_{C-H}), 2853 (ν_{C-H}), 1972 (CO), 1874 (CO), 1856 (CO). HRMS (ESI): *m/z* for $C_{22}H_{39}MnNO_3P$ $[M-Me]^+$ = 436.1807 (calc.), found: 436.1817.

5.7 *fac*-[Mn($P^{Ph}N^{Pyrr}$)$(CO)_3CH_3$] (PN7)

In a Schlenk flask, triflate complex **2c** (107 mg, 0.196 mmol, 1.00 equiv.) was suspended in 1,4-dioxane (6 mL) and MeMgCl (0.13 mL, 3.0 M in THF, 0.390 mmol, 1.98 equiv.) was added dropwise, upon which the yellow suspension changed to brown. After stirring for 2 hours and 15 minutes the solvent was evaporated under reduced pressure. The reaction mixture was extracted with toluene (3 x 5 mL) and filtered *via* syringe filter. After evaporation of the solvent and drying, a yellow residue remained. The residue was washed with *n*-pentane (1.5 mL), yielding **PN7** as a pale yellow powder (22.6 mg, 48 %). ^{1}H NMR (400 MHz, CD_2Cl_2) δ: = 7.80 – 7.70 (m, 2H, Ph), 7.53 – 7.31 (m, 8H, Ph), 3.31 – 3.20 (m, 1H, $NC\underline{H_2}CH_2CH_2C\underline{H_2}$), 3.04 – 2.96 (m, 1H, $NC\underline{H_2}CH_2CH_2C\underline{H_2}$), 2.86 – 2.72 (m, 1H, $NCH_2C\underline{H_2}P$), 2.72 – 2.53 (m, 2H, $NC\underline{H_2}CH_2P$, $NC\underline{H_2}CH_2CH_2C\underline{H_2}$), 2.53 – 2.29 (m, 3H, NCH_2CH_2P, $NC\underline{H_2}CH_2CH_2C\underline{H_2}$), 2.23 – 2.06 (m, 1H, $NCH_2C\underline{H_2}C\underline{H_2}CH_2$), 2.04 – 1.75 (m, 3H, $NCH_2C\underline{H_2}C\underline{H_2}CH_{2f}$), -0.73 (d, *J* = 10.2 Hz, 3H, Mn-CH_3). $^{13}C\{^1H\}$ NMR (101 MHz, CD_2Cl_2) δ: = 136.9 (d, *J* = 38.3 Hz, Ph^1), 133.7 (d, *J* = 37.0 Hz, Ph^1), 132.5 (d, *J* = 10.4 Hz, $Ph^{2,3,5,6}$), 131.5 (d, *J* = 9.1 Hz, $Ph^{2,3,5,6}$), 130.5 (d, *J* = 2.2 Hz, Ph^4), 129.7 (d, *J* = 2.2 Hz, Ph^4), 129.1 (d, *J* = 9.2 Hz, $Ph^{2,3,5,6}$), 128.8 (d, *J* = 9.1 Hz, $Ph^{2,3,5,6}$), 65.9 ($N\underline{C}H_2CH_2CH_2\underline{C}H_2$), 60.2 ($N\underline{C}H_2CH_2CH_2\underline{C}H_2$), 57.7 (d, *J* = 9.5 Hz, $N\underline{C}H_2CH_2P$), 26.9 (d, *J* = 16.9 Hz, $NCH_2\underline{C}H_2P$), 23.4 ($NCH_2\underline{C}H_2\underline{C}H_2CH_2$), 22.3 ($NCH_2\underline{C}H_2\underline{C}H_2CH_2$), -1.6 (d, *J* = 18.1 Hz, Mn-CH_3), $C_{q(CO)}$ not observed. $^{31}P\{^1H\}$ NMR (162 MHz, CD_2Cl_2) δ: = 70.0. IR (ATR, cm^{-1}) = 1982 (CO), 1889 (CO), 1855 (CO). HRMS (ESI): *m/z* for $C_{22}H_{25}MnNO_3P$ $[M+Na]^+$ = 460.0844 (calc.), found: 460.0863.

5.8 *fac*-[Mn($P^{Ph}N^{Et}$)$(CO)_3CH_3$] (PN8)

In a Schlenk flask, triflate complex **2b** (100 mg, 0.174 mmol, 1.00 equiv.) was suspended in a mixture of 1,4-dioxane (6 mL) and Et_2O (4mL), cooled to 0 °C followed by the dropwise addition of and MeMgCl (0.15 mL, 3.0 M in THF, 0.450 mmol, 2.58 equiv.), upon which the yellow suspension changed to brown. After 1h, the solution mixture was allowed to reach RT and stirred for 3 hours. The solvent was evaporated under reduced pressure and the reaction mixture was extracted with toluene (3 x 5 mL) and filtered *via* syringe filter. After evaporation of the solvent and drying, a yellow residue remained. The residue was washed with *n*-pentane (4 x 1.5 mL), yielding **PN8** as a yellow powder (20.2 mg, 26 %). ^{1}H NMR (400 MHz, CD_2Cl_2) δ: = 7.79 – 7.69 (m, 2H, $Ph^{2,6}$), 7.49 – 7.35 (m, 8H, Ph^{3-5}), 3.13 – 2.81 (m, 3H, NCH_2CH_2P, NCH_2CH_3), 2.7 – 2.30 (m, 5H, NCH_2CH_2P, NCH_2CH_3), 1.29 (t, *J* = 7.0 Hz, 3H, NCH_3), 0.96 (t, *J* = 7.2 Hz, 3H, NCH_3), -0.79 (d, *J* = 10.3 Hz, 3H, Mn-CH_3). $^{13}C\{^1H\}$ NMR (101 MHz, CD_2Cl_2) δ: = 132.6 (d, *J* = 10.4 Hz, $Ph^{2,3,5,6}$), 131.3 (d, *J* = 8.9 Hz, $Ph^{2,3,5,6}$), 130.5 (Ph^4), 129.6 (Ph^4), 129.1 (d, *J* = 9.1 Hz, $Ph^{2,3,5,6}$), 128.8 (d, *J* = 9.0 Hz, $Ph^{2,3,5,6}$), 55.7 (d, *J* = 9.0 Hz, $N\underline{C}H_2CH_2P$), 54.9 ($N\underline{C}H_2NCH_3$), 50.2 ($N\underline{C}H_2NCH_3$), 26.0 (d, *J* = 16.9 Hz, $NCH_2\underline{C}H_2P$), 11.1 ($NCH_2\underline{C}H_3$), 8.3 ($NCH_2\underline{C}H_3$), -1.0 (d, *J* = 17.7 Hz, Mn-CH_3), C_q not observed. $^{31}P\{^1H\}$ NMR (162 MHz, CD_2Cl_2) δ: = 67.3. IR (ATR, cm^{-1}) = 1983 (CO), 1891 (CO), 1858 (CO). HRMS (ESI): *m/z* for $C_{22}H_{27}MnNO_3P$ $[M-Me]^+$ = 424.0869 (calc.), found: 424.0872.

6 Synthesis and Characterization of a PP-based bidentate ligand and the corresponding Mn(I) Carbonyl Complexes

1,2-Bis(dicyclohexylphosphino)ethane (L10)

Analytical data is consistent with the literature.[13] The synthesis procedure can be taken from section 1 (Route C). Cy_2PH (1.16 g, 5.84 mmol, 1.00 equiv.); *n*-BuLi (2.34 mL, 2.5 M, 5.85 mmol, 1.00 equiv.); $Me_2N(CH_2)_2Cl$ (321 mg, 2.98 mmol, 0.51 equiv.); solvent: 20 mL THF; extracted with *n*-pentane (6x 10 mL); 388 mg (31 %) as white, crystalline solid. ^{1}H NMR (250 MHz, C_6D_6) δ: = 2.09 – 1.47 (m, 26H), 1.47 – 0.94 (m, 20H). $^{31}P\{^1H\}$ NMR (101 MHz, C_6D_6) δ: = 0.8 ppm.

fac-[Mn($P^{Cy}P^{Cy}$)$(CO)_3$Br] (1j)

Analytical data is consistent with the literature.[14] The synthesis procedure can be taken from section 2. **L10** (315 mg, 0.745 mmol, 1.00 equiv.); Mn(CO)5Br (205 mg, 0.756 mmol, 1.00 equiv.); solvent: 15 mL toluene; precipitated and washed with n-pentane; 444 mg (93 %) as yellow powder. ^{1}H NMR (400 MHz, C6D6) δ 2.71 – 2.58 (m, 1H), 2.41 (d, J = 11.9 Hz, 1H), 2.19 – 1.27 (m, 23H), 1.27 – 0.74 (m, 11H). $^{31}P\{^1H\}$ NMR (162 MHz, CD_2Cl_2) δ: = 72.4 ppm.

fac-[Mn($P^{Cy}P^{Cy}$)(CO)$_3$OTf] (2j)

The synthesis procedure can be taken from section 3. **1j** (283 mg, 0.441 mmol, 1.00 equiv.); AgOTf (196 mg, 0.763 mmol, 1.73 equiv.); solvent: 15 mL DCM; washed with n-pentane (2 x 5 mL); 152 mg (48 %) as orange powder. ^{1}H NMR (250 MHz, CD_2Cl_2) δ: = 2.43 – 0.96 (m,). ^{13}C NMR (151 MHz, CD_2Cl_2) δ: = 217.6 (CO), 119.5 (d, *J* = 319.3 Hz, CF_3), 39.9 – 39.4 (m), 38.2 – 37.8 (m), 34.2 (t, *J* = 6.8 Hz), 30.7, 30.5, 30.1, 29.8, 29.4, 28.9 (t, *J* = 3.1 Hz), 28.0 (t, *J* = 5.3 Hz), 27.8 – 27.6 (m), 27.5 – 27.0 (m), 26.3 (d, *J* = 13.6 Hz), 26.0, 21.5 (t, *J* = 17.3 Hz, PCH_2CH_2P). ^{31}P{^{1}H} NMR (101 MHz, CD_2Cl_2) δ: = 76.9. ^{19}F{^{1}H} NMR (377 MHz, CD_2Cl_2) δ: = -76.7. IR (ATR, cm^{-1}) = 2933 (ν_{C-H}), 2850 (ν_{C-H}), 2025 (CO), 1961 (CO), 1908 (CO).

fac-[Mn($P^{Cy}P^{Cy}$)(CO)$_3$Me] (PP4)

In a Schlenk flask, the triflate complex **2j** (152 mg, 0.214 mmol 1.00 equiv.) was suspended in Et_2O (8 mL), cooled to -80 °C and subsequently MeLi (0.21 mL, 1.6 M in Et_2O, 0.336 mmol, 1.57 equiv.) was added over a period of 3 minutes. During addition, the yellow suspension turned brown. After 50 minutes, the solution mixture was allowed to reach RT and stirred for 1.5 hours. The solvent was evaporated, the remained residue was extracted with toluene (3 x 10 mL) and filtered *via* a syringe filter. After evaporation of the solvent, the solid was digested in *n*-pentane (4 mL) for 1 hour. Upon drying of the precipitate, **PP4** was obtained as an off-white powder (53.0 mg, 43 %). ^{1}H NMR (400 MHz, C_6D_6) δ: = 2.32 – 2.15 (m, 2H), 2.04 – 1.80 (m, 8H), 1.79 – 0.80 (m, 32H), -0.40 (t, J = 8.2 Hz, 3H). ^{13}C{^{1}H} NMR (151 MHz, CD_2Cl_2) δ: = 227.7 (CO), 39.4 – 39.1 (m, Cy^1), 35.2 (t, *J* = 7.7 Hz, Cy^1), 30.8, 30.2, 29.3, 29.0, 28.3 – 28.1 (m), 27.9 (t), 27.7 (t, *J* = 4.7 Hz), 26.8, 26.6, 22.7 (t, *J* = 18.2 Hz, PCH_2CH_2P), -16.5 (t, *J* = 16.6, Mn-CH_3). ^{31}P{^{1}H} NMR (162 MHz, CD_2Cl_2) δ: = 84.5. IR (ATR, cm^{-1}) = 2931 (ν_{C-H}), 2852 (ν_{C-H}), 1975 (CO), 1897 (CO), 1868 (CO). HRMS (ESI): *m/z* for $C_{30}H_{51}MnNO_3P_2$ $[M-Me]^+$ = 599.2586 (calc.), found: 599.2591.

7 Synthesis and Characterization of Mn(I) Carbonyl Complexes supported by monodentate Ligands

trans-[Mn(PPh_3)$_2$)(CO)$_3$Br] (1k)

A *trans* configuration is assumed due to corresponding IR band intensities reported in 1963.[15]

In a Schlenk flask, Mn(CO)$_5$Br (354 mg, 1.29 mmol, 1.00 equiv.) was mixed excess of PPH_3 (1.84 g, 7.02 mmol, 4.86 equiv.) and heated to 120°, neat, for one hour. The reaction mixture was exposed to air, suspended with Et_2O (2 x 50 mL) and decanted after the solid settled. The yellow product was dried under reduced pressure and the residue was taken up in chloroform (100 mL). After fully precipitation with petroleum ether, the suspension was cooled to -18° and filtered over a G3-glas frit. The complex was washed with Et_2O (2 x 10 mL) and dried under vacuum, yielding the yellow powder **1k** (791 mg, 82%). **1k** was synthesized according to the literature.[16] ^{1}H NMR (400 MHz, CD_2Cl_2) δ: = 7.95 – 7.56 (m, 12H, Ph^{2,6}), 7.56 – 7.15 (m, 18H, Ph^{3,4,5}). ^{31}P{^{1}H} NMR (162 MHz, CD_2Cl_2) δ: = 54.3. IR (ATR, cm^{-1}) = 2040 (CO), 1941 (CO), 1914 (CO).

trans-[Mn(PPh_3)$_2$)(CO)$_3$Me] (PP5)

Analytical data is consistent with the literature.[17] Inside an argon flushed glove box, the bromide complex **1k** (116 mg, 0.156 mmol, 1.00 equiv.) was dissolved in THF (6 mL) in a Schlenk flask and mixed with sodium-sand (32.0 mg, 1.39 mmol, 8.94 equiv.). The reaction mixture was stirred at room temperature overnight. Meanwhile, the reaction mixture turned brown. The flask was transferred out of the glovebox, cooled to -70 °C followed by a dropwise addition of methyl iodide (0.5 mL). Stirring was continued for 1 hour and afterwards the suspension was allowed to reach RT, upon which the color changed to green. After 2 hours the solvent was evaporated, the residue extracted with *n*-pentane (3 x 8 mL) and filtered *via* a syringe filter. The solvent was removed under reduced pressure, yielding **PP5** as a yellow powder (43.0 mg, 41 %). **PP5** was synthesized by a slightly modified procedure from the literature.[17] ^{1}H NMR (250 MHz, CD_2Cl_2) δ: = 7.72 – 7.52 (m, 12H, Ph^{2,6}), 7.49 – 7.28 (m, 18H, Ph^{3,4,5}), -1.07 (t, *J* = 6.7 Hz, 3H, Mn-CH_3). ^{13}C NMR (151 MHz, THF-d_8) δ: = 138.4 (d, *J* = 12.1 Hz),

136.8, 136.8, 136.6 (d, $J = 6.0$ Hz), 136.5, 136.5, 134.4, 134.3 – 134.1 (m), 134.0, 134.0, 132.0, 131.0 (d, $J = 2.2$ Hz), 130.0, 129.5 – 128.9 (m), 128.6 (t, $J = 4.4$ Hz), -1.2 (t, $J = 9.52$ Hz, Mn-CH_3), $C_{q(CO)}$ not observed. $^{31}P\{^1H\}$ NMR (101 MHz, CD_2Cl_2) δ: = 78.7. IR (ATR, cm^{-1}) = 1919 (CO), 1875 (CO), 1850 (CO).

8 Catalytic Dimerization of Phenylacetylene

Scheme 6. Dimerization of Phenylacetylene

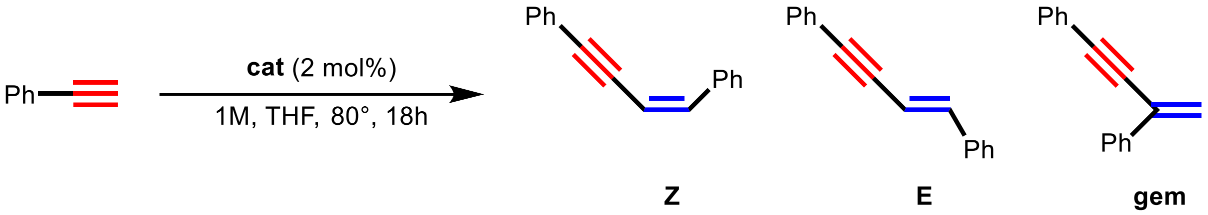

8.1 General Procedure

Inside an argon flushed glove box, a screw cap vial was charged with phenylacetylene (0.600 mmol, 1.00 equiv.), the catalyst (0.012 mmol, 2 mol%), and THF (1 M). The vial was transferred out of the glovebox and stirred for 18 hours at 80 °C. The solution was allowed to reach RT and exposed to air. An aliquot was analyzed by GC-MS. The catalytic performance is summarized in Table S2.

Table S2. Reaction Conditions and Catalytic Performance of Mn(I) methyl Complexes for the Dimerization of Phenylacetylene

Catalyst (2 mol%)	Reaction Time	Color[b]	Conversion[a] [%]	*Z:E*
**PN1**	18 h	red	75	95:5
**PN2**	18 h	red/brownish	35	96:4
**PN3**	18 h	yellow	14	90:10
**PN4**	18 h	yellow	12	90:10
**PN5**	18 h	red	n.d.	-
**PN6**	18 h	orange	trace	-
**PN7**	18 h	yellow	n.d.	-
**PN8**	18 h	orange	n.d.	-
**PP4**	18 h	orange	36	91:9
**PP5**	18 h	red	n.d	-
**-**	18 h	colorless	n.d	-

[a]Conversion and E/Z-ratio detected by GC-MS. [b]Color of the reaction mixture, n.d = not detected.

8.2 Optimization Reactions for the Dimerization of Phenylacetylene

Inside an argon flushed glove box, a screw cap vial was charged with phenylacetylene (0.600 mmol, 1.00 equiv.), the catalyst **PN1** entry 1–6 (0.012 mmol, 2 mol%) or entry 7 (0.030 mmol, 5 mol%) and the solvent (1 M). The vial was transferred out of the glovebox and stirred for 18 hours, at 80 °C. The solution was allowed to reach RT and exposed to air. An aliquot was analyzed by GC-MS. The solvent screening is depicted in Table S3.

Table S3. Optimization Reactions for the Dimerization of Phenylacetylene catalyzed by PN1

Entry	Solvent	T [°C]	Reaction Time	Color[b]	Conversion[a] [%]	*Z:E*
1	EtOH	80	18h	brown	95	88:12
2	MeOH	80	18h	red	86	93:7
3	*i*-PrOH	80	18h	brown	86	88:12
4	Toluene	80	18h	red	66	89:11
5	ACN	80	18h	black	62	92:8
6	DCE	80	18h	red	76	93:7
7	DCE	80	18h	red	93	91:9

[a]Conversion and E/Z-ratio detected by GC-MS. [b]Color of the reaction mixture.

9 Catalytic Hydroboration of 4-Chlorstyrene

Scheme 7. Hydroboration of 4-Chlorostyrene

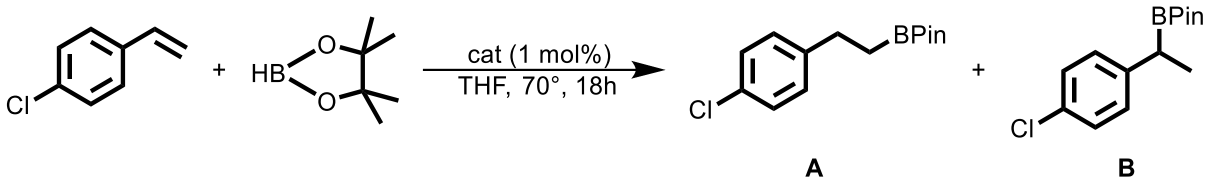

Inside an argon flushed glove box, a screw cap vial was charged with 4-chlorstyrene (1.10 mmol, 1.00 equiv.), pinacolborane (1.12 mmol, 1.02 equiv.), catalyst (0.011 mmol, 1mol%) and THF (2 M). The vial was transferred out of the glovebox and stirred for 18 hours at 70 °C. The solution was allowed to reach RT and exposed to air. An aliquot was analyzed by GC-MS. The catalytic performance is summarized in Table S4.

Table S4. Reaction Conditions and Catalytic Performance of selected Mn(I) methyl Complexes for the Hydroboration of 4-Chlorstyrene

PN-Catalyst	Cat. Loading [mol%]	T [°C]	Reaction Time	Conversiona[a] [%]	A:B[a]
**PN1**	1	70	18h	41	>99:1
**PN2**	1	70	18h	67	99:1
**PN3**	1	70	18h	22	>99:1
**PN4**	1	70	18h	16	97:3
**PN5**	1	70	18h	99	>99:1
**PN6**	1	70	18h	93	99:1
**PN7**	1	70	18h	43	>99:1
**PN8**	1	70	18h	57	>99:1
**PP4**	1	70	18h	80	>99:1
**PP5**	1	70	18h	87	>99:1
**-**	-	70	18h	6	>99:1

[a]Conversion and E/Z-ratio detected by GC-MS.

10 NMR/IR spectra

10.1 PN-bidentate ligands and the corresponding Mn(I) Carbonyl Complexes

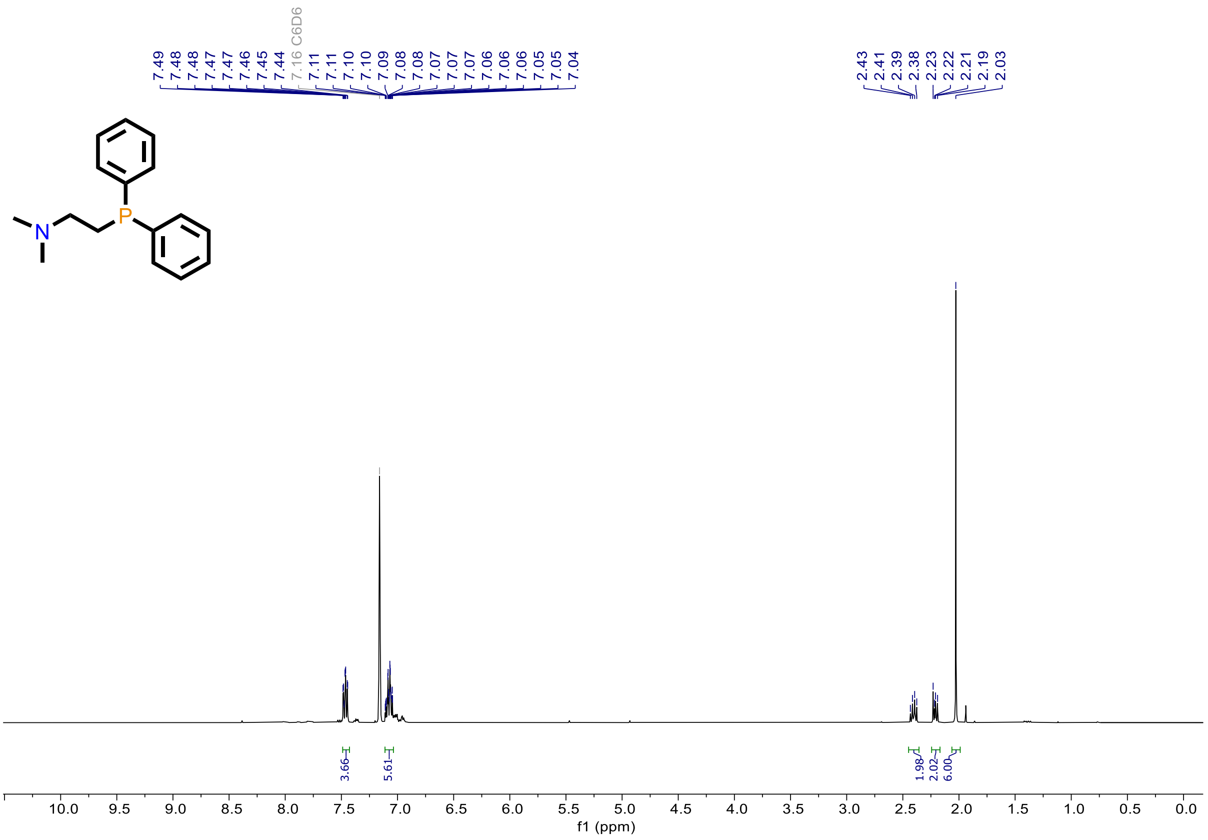

Figure S1. ^{1}H NMR (400 MHz, C_6D_6) of 2-(Diphenylphosphino)-*N*,*N*-dimethylethanamine (**L1**)

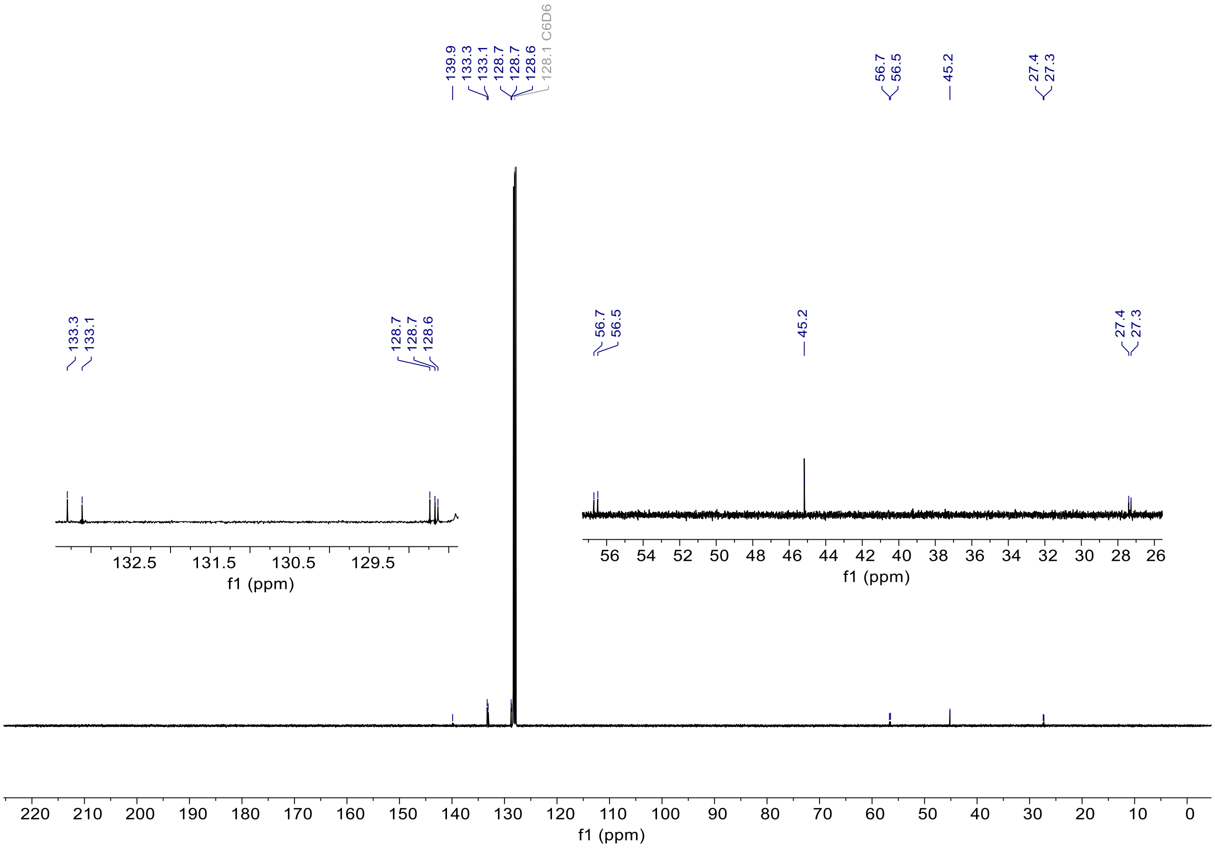

Figure S2. $^{13}C\{^1H\}$ NMR (101 MHz, C^6D^6) of 2-(Diphenylphosphino)-*N*,*N*-dimethylethanamine (**L1**)

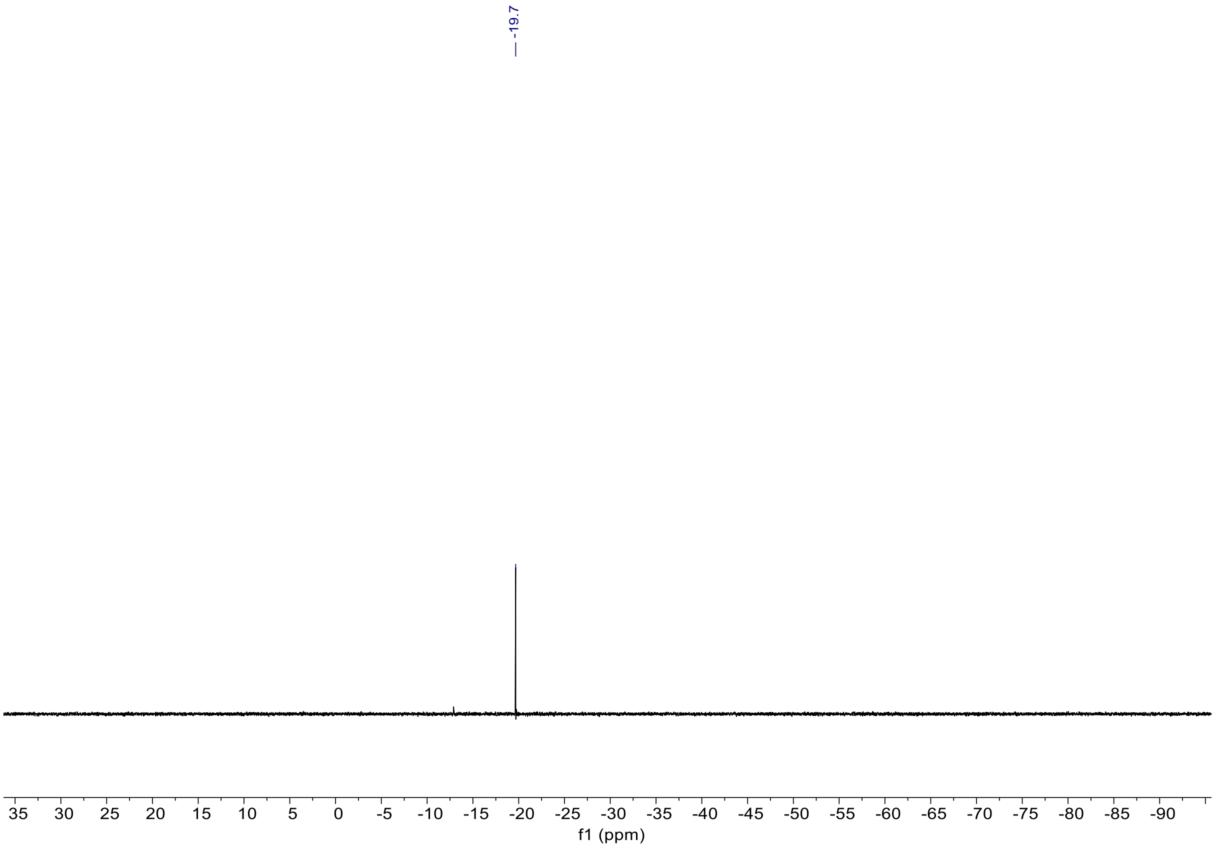

Figure S3. $^{31}P\{^1H\}$ NMR (162 MHz, C_6D_6) of 2-(Diphenylphosphino)-*N*,*N*-dimethylethanamine (**L1**)

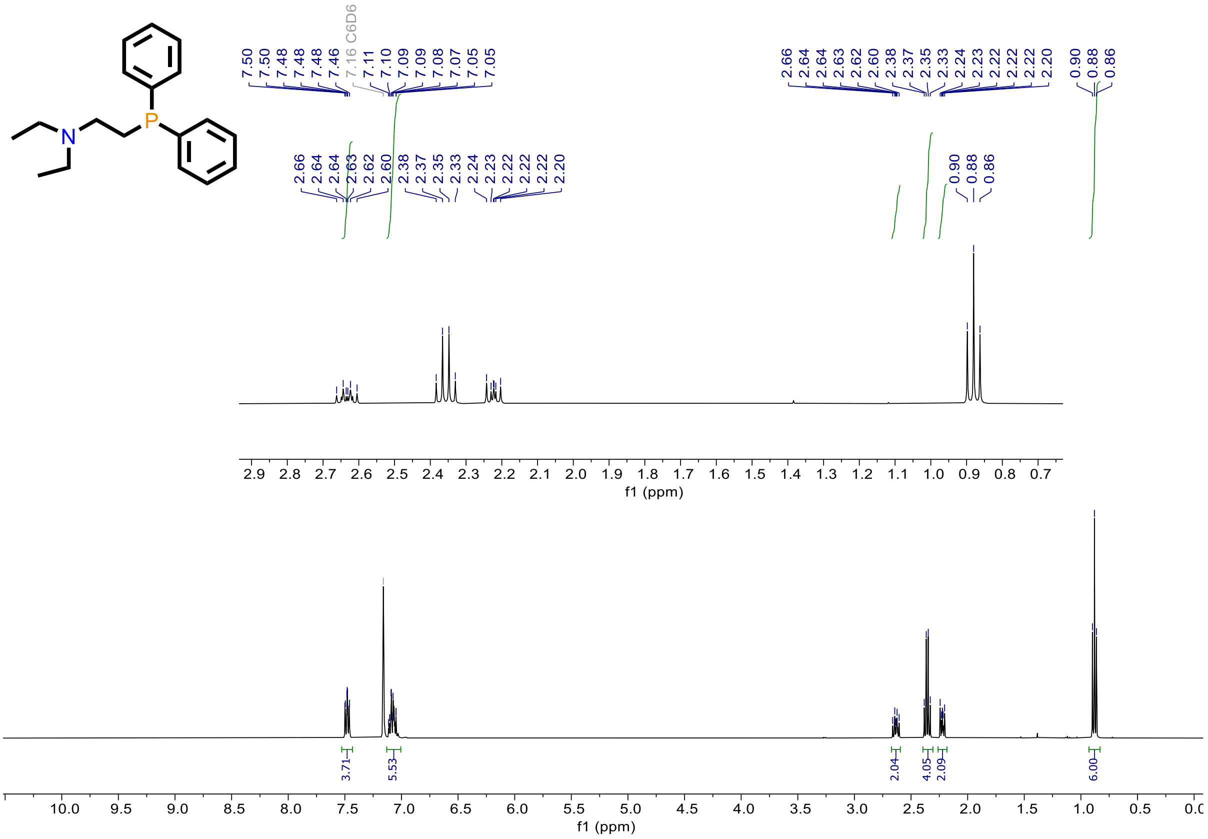

Figure S4. ^{1}H NMR (400 MHz, C_6D_6) of 2-(Diphenylphosphino)-*N*,*N*-diethylethanamine (**L2**)

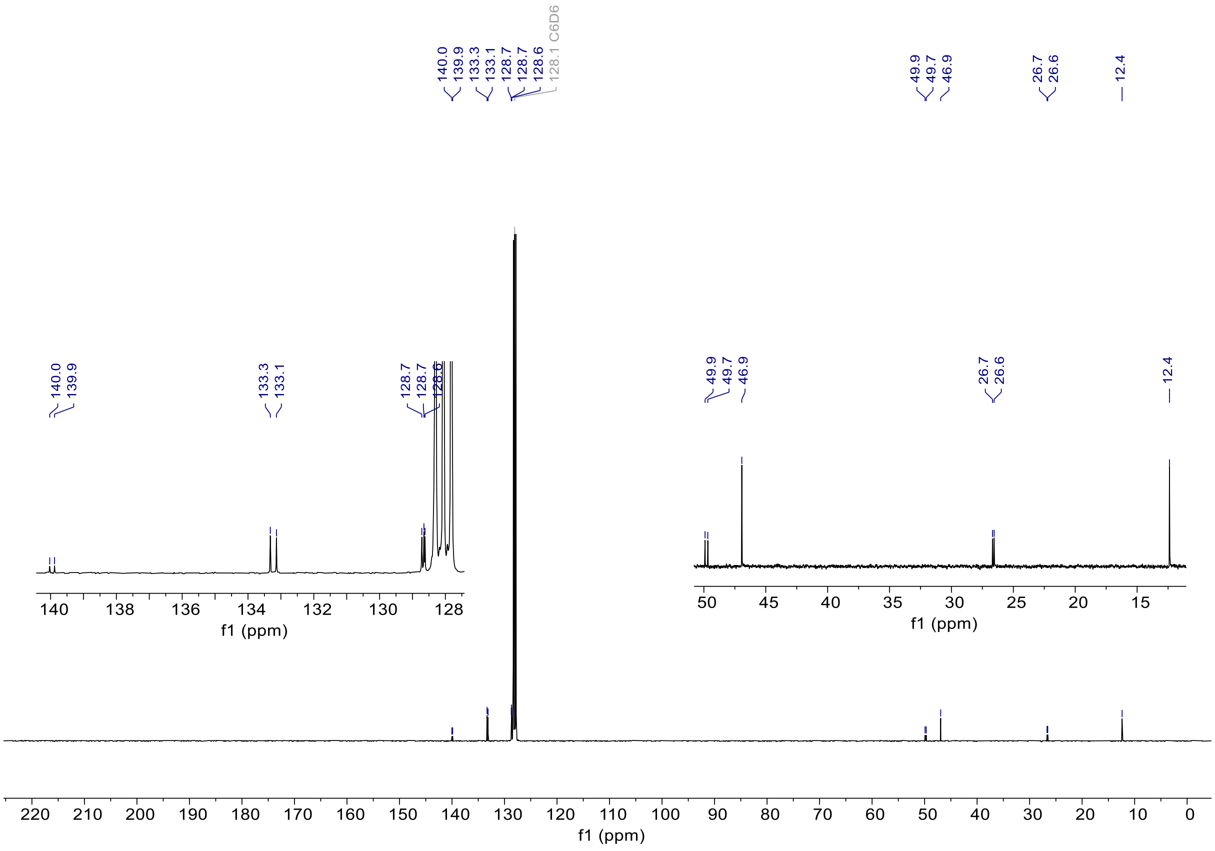

Figure S5. $^{13}C\{^{1}H\}$ NMR (101 MHz, C_6D_6) of 2-(Diphenylphosphino)-*N*,*N*-diethylethanamine (**L2**)

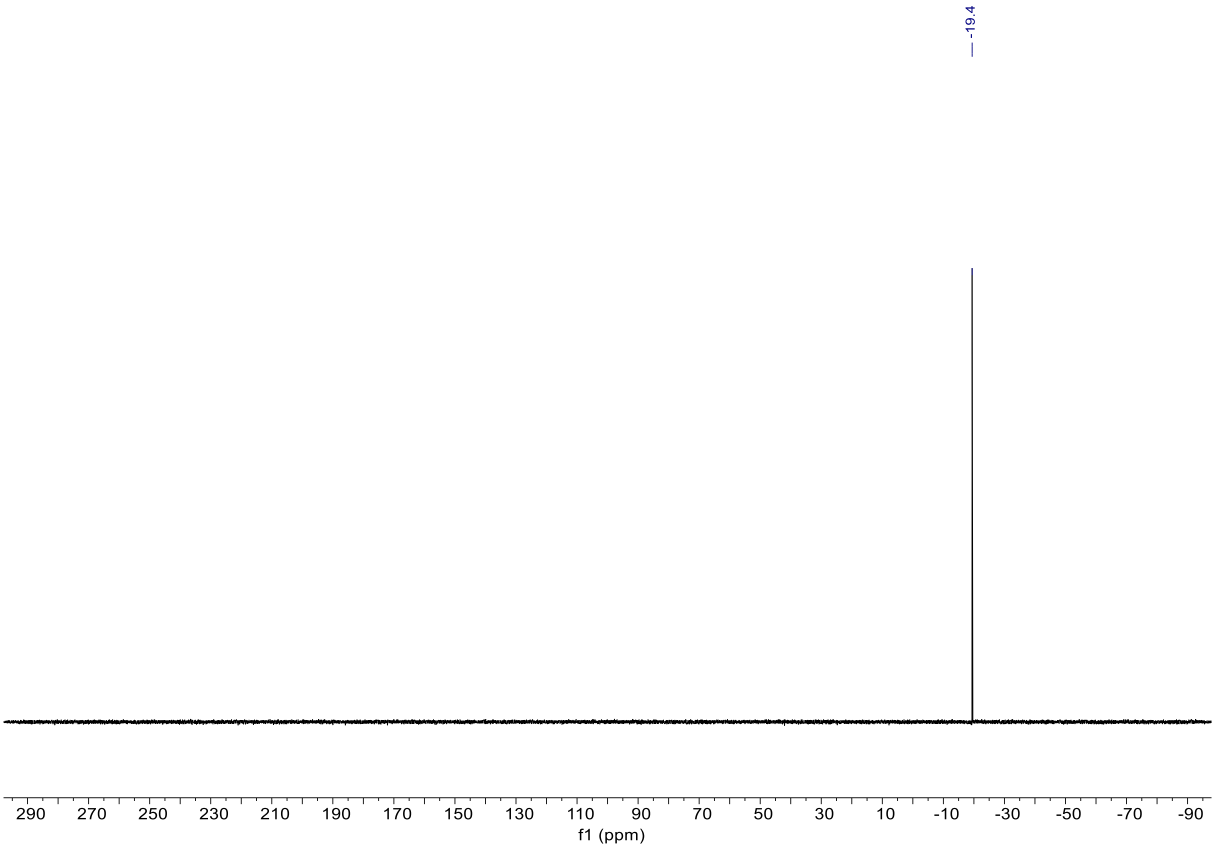

Figure S6. $^{31}P\{^{1}H\}$ NMR (162 MHz, C_6D_6) of 2-(Diphenylphosphino)-*N*,*N*-diethylethanamine (**L2**)

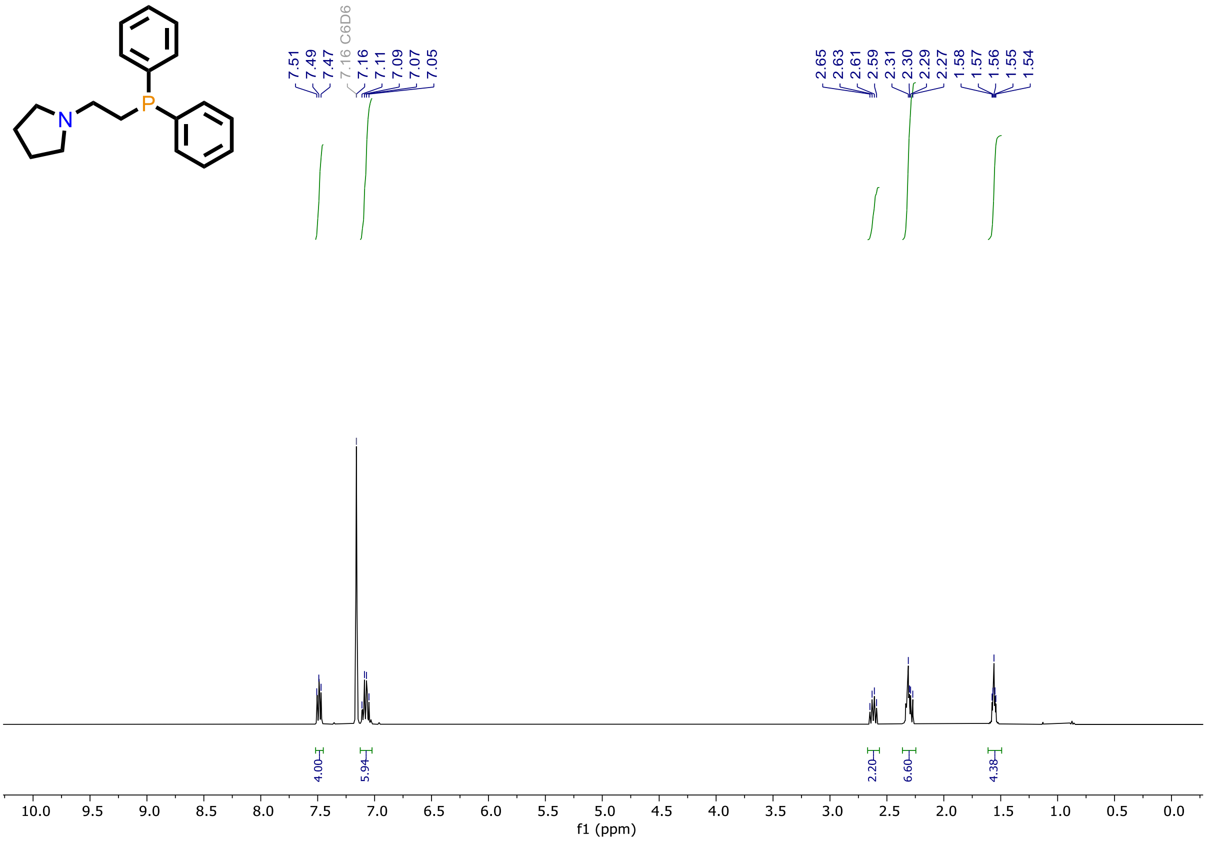

Figure S7. ^{1}H NMR (400 MHz, C_6D_6) of 1-[2-(Diphenylphosphino)ethyl]pyrrolidine (**L3**)

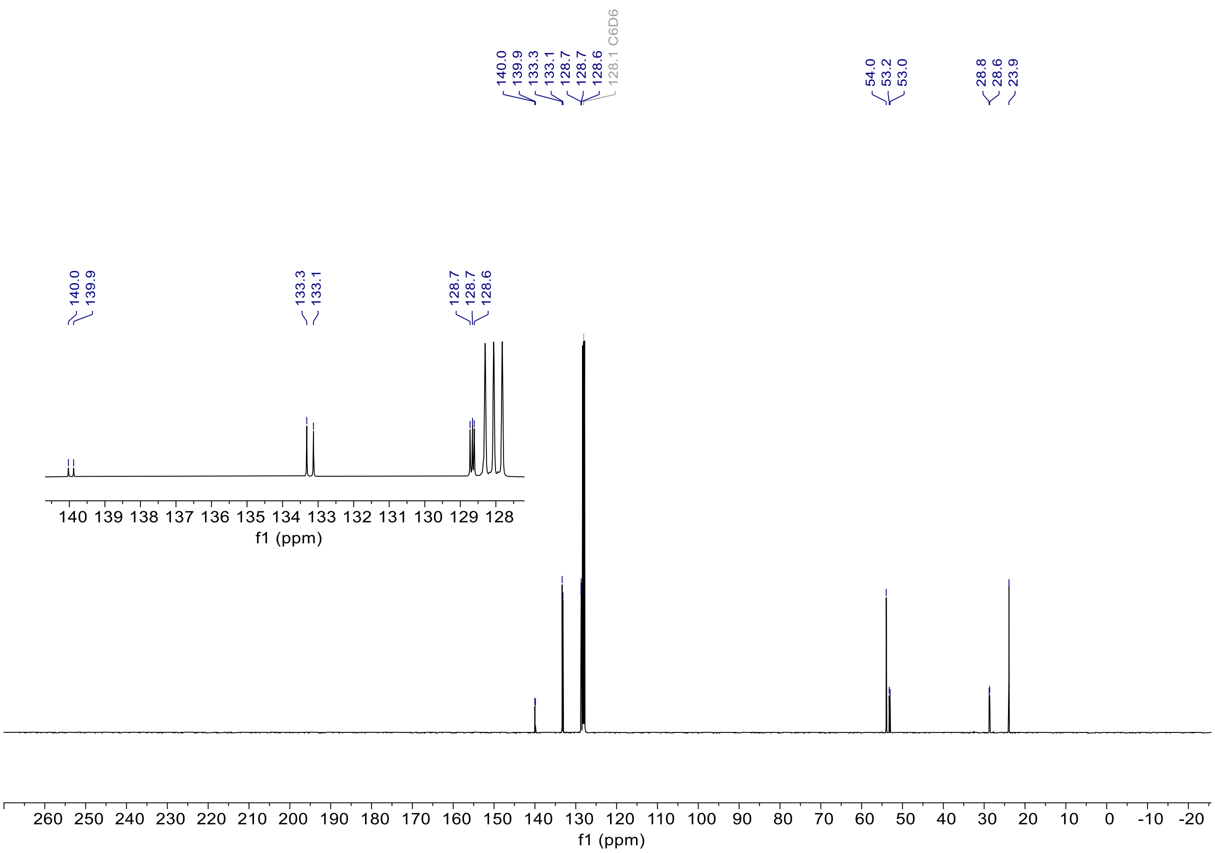

Figure S8. $^{13}C\{^1H\}$ NMR (101 MHz, CD_2Cl_2) of 1-[2-(Diphenylphosphino)ethyl]pyrrolidine (**L3**)

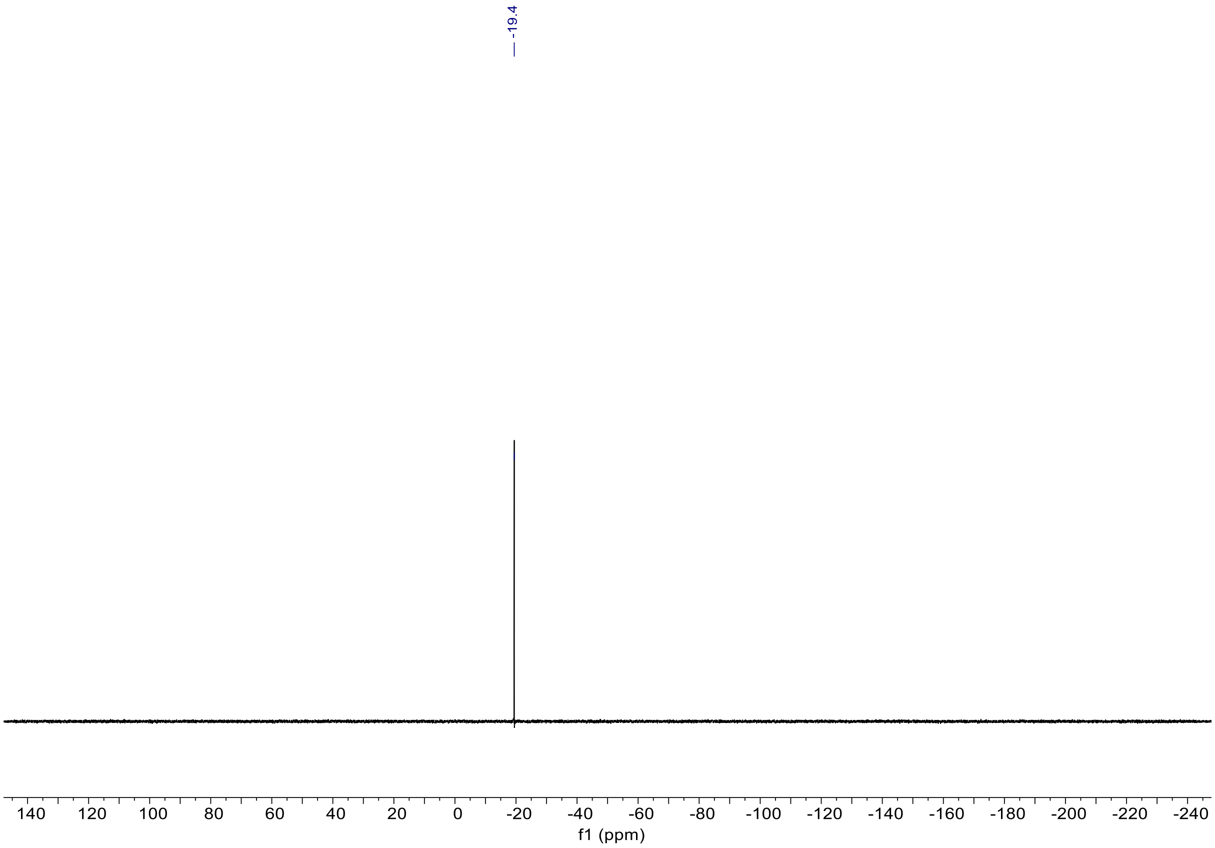

Figure S9. $^{31}P\{^1H\}$ NMR (162 MHz, C_6D_6) of 1-[2-(Diphenylphosphino)ethyl]pyrrolidine (**L3**)

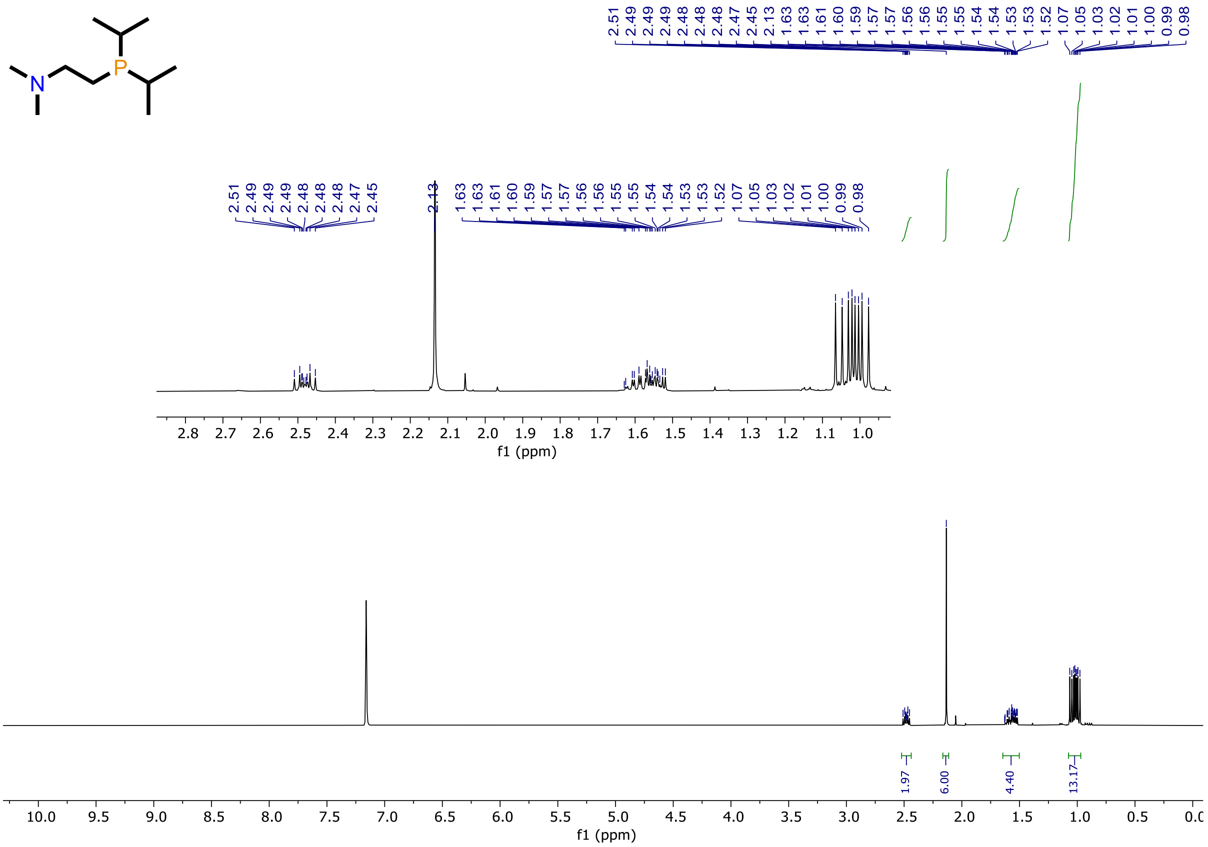

Figure S10. ^{1}H NMR (400 MHz, C_6D_6) of [2-(Diisopropylphosphine)ethyl]dimethylamine (**L4**)

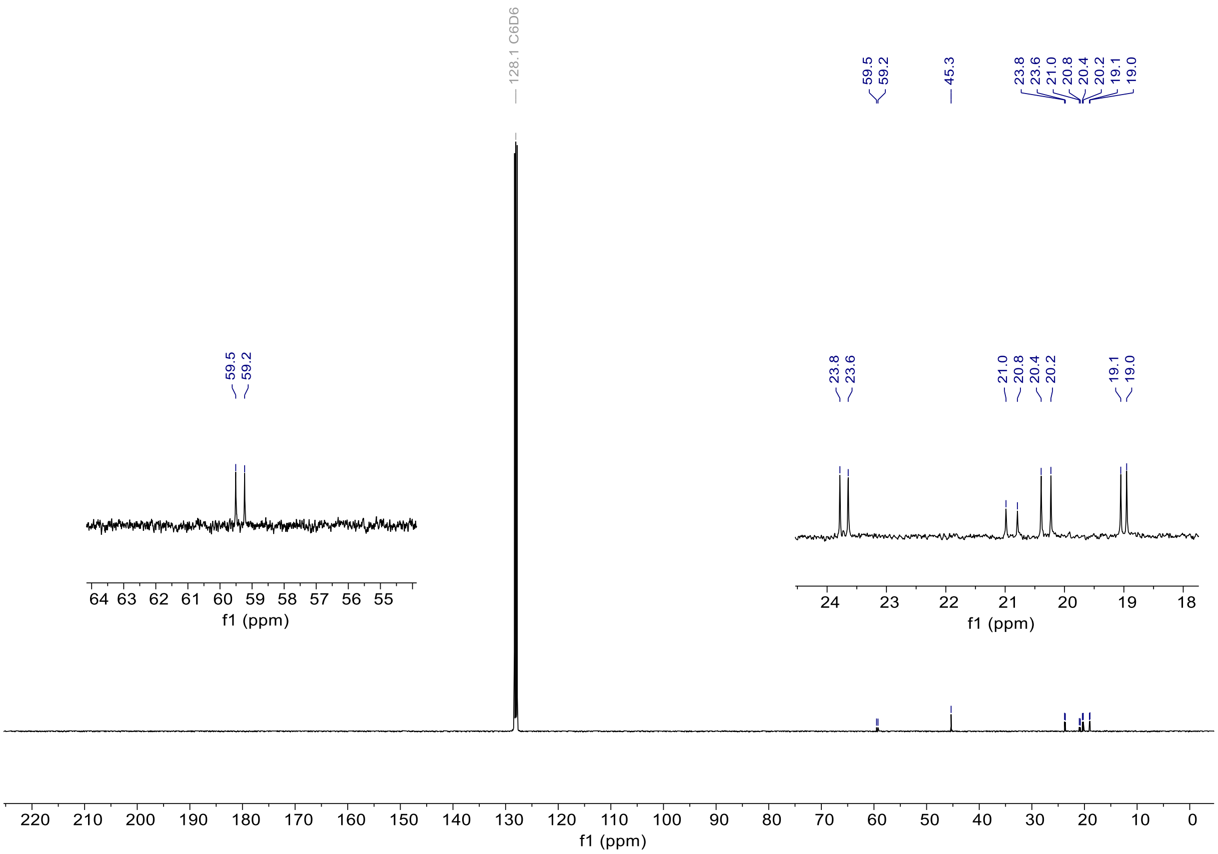

Figure S11.$^{13}C\{^{1}H\}$ NMR (101 MHz, C_6D_6) of [2-(Diisopropylphosphine)ethyl]dimethylamine (**L4**)

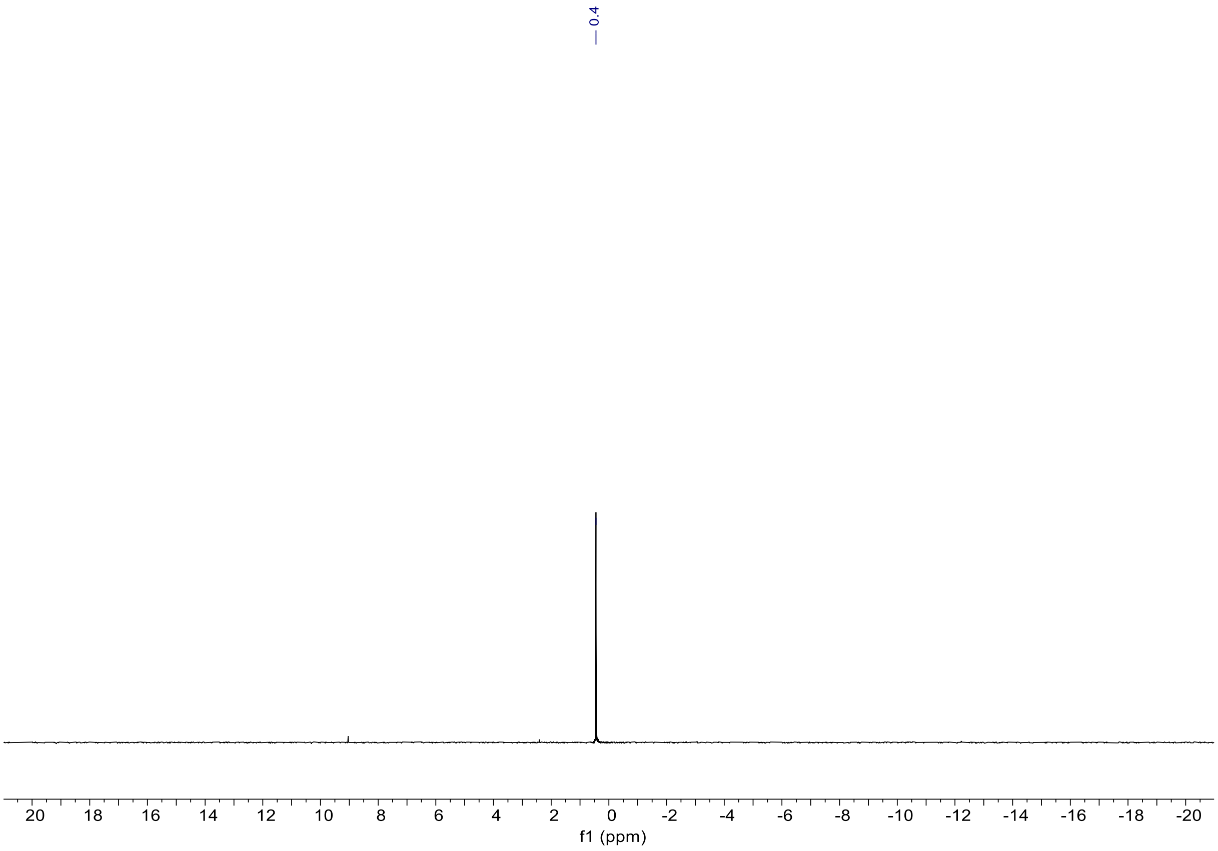

Figure S12. $^{31}P\{^{1}H\}$ NMR (162 MHz, C_6D_6) of [2-(Diisopropylphosphine)ethyl]dimethylamine (**L4**)

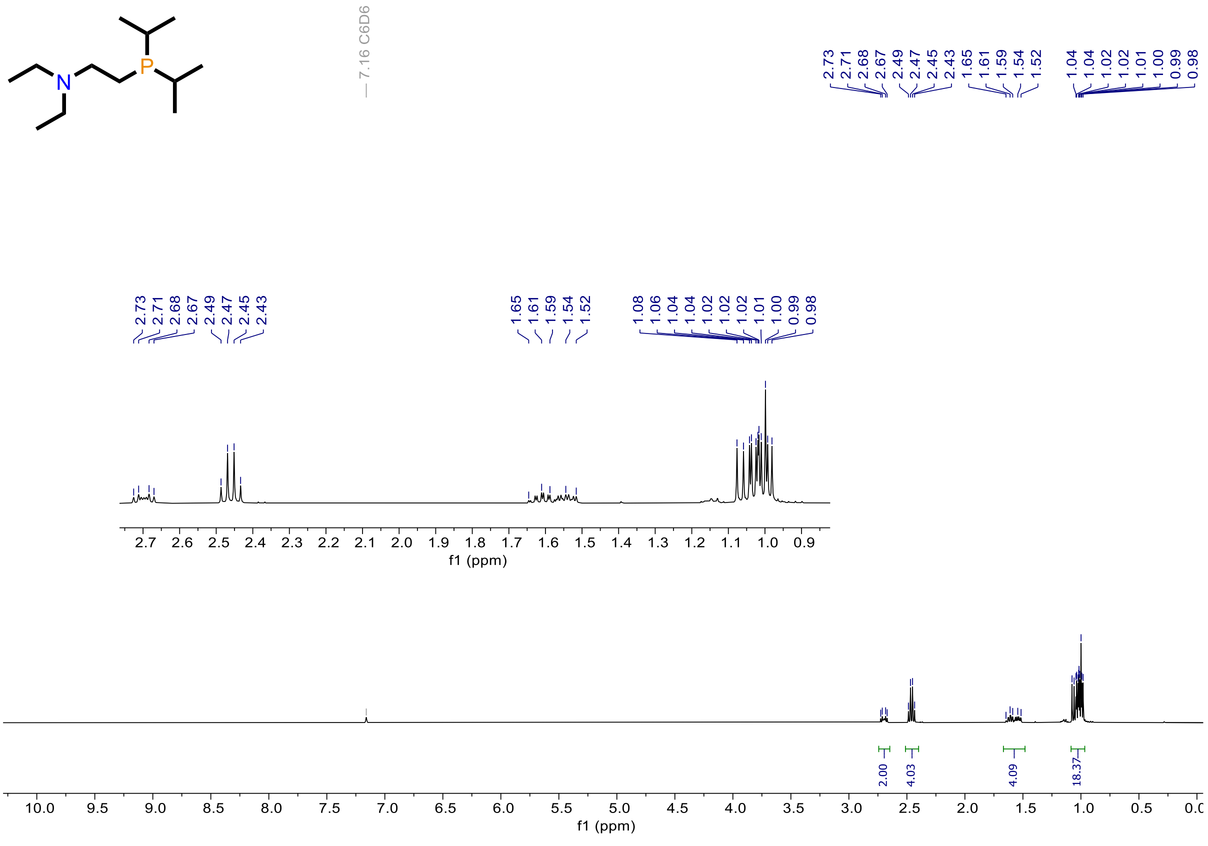

Figure S13. ^{1}H NMR (400 MHz, C_6D_6) of 2-(Diisopropylphosphino)-*N,N*-diethylethanamine (**L5**)

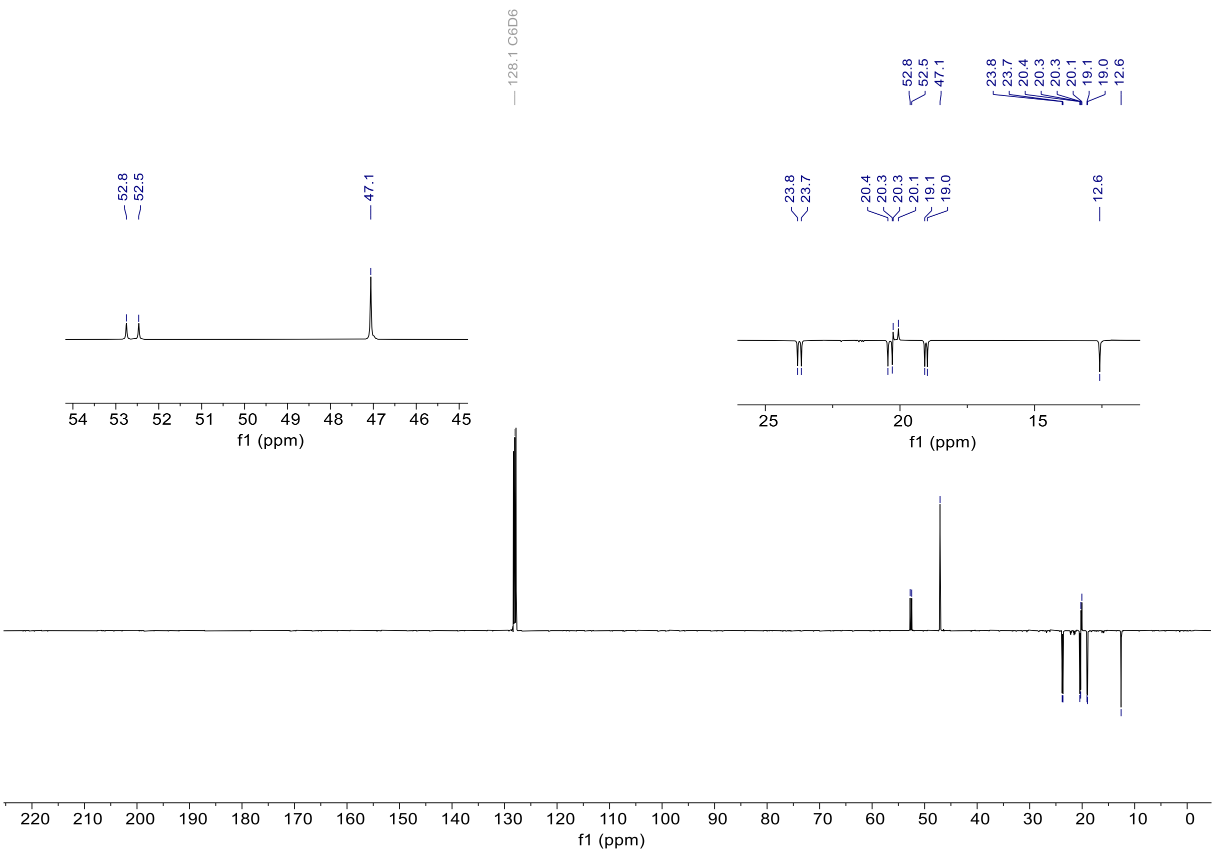

Figure S14. $^{13}C\{^1H\}$ NMR (101 MHz, C_6D_6) of 2-(Diisopropylphosphino)-*N,N*-diethylethanamine (L5).

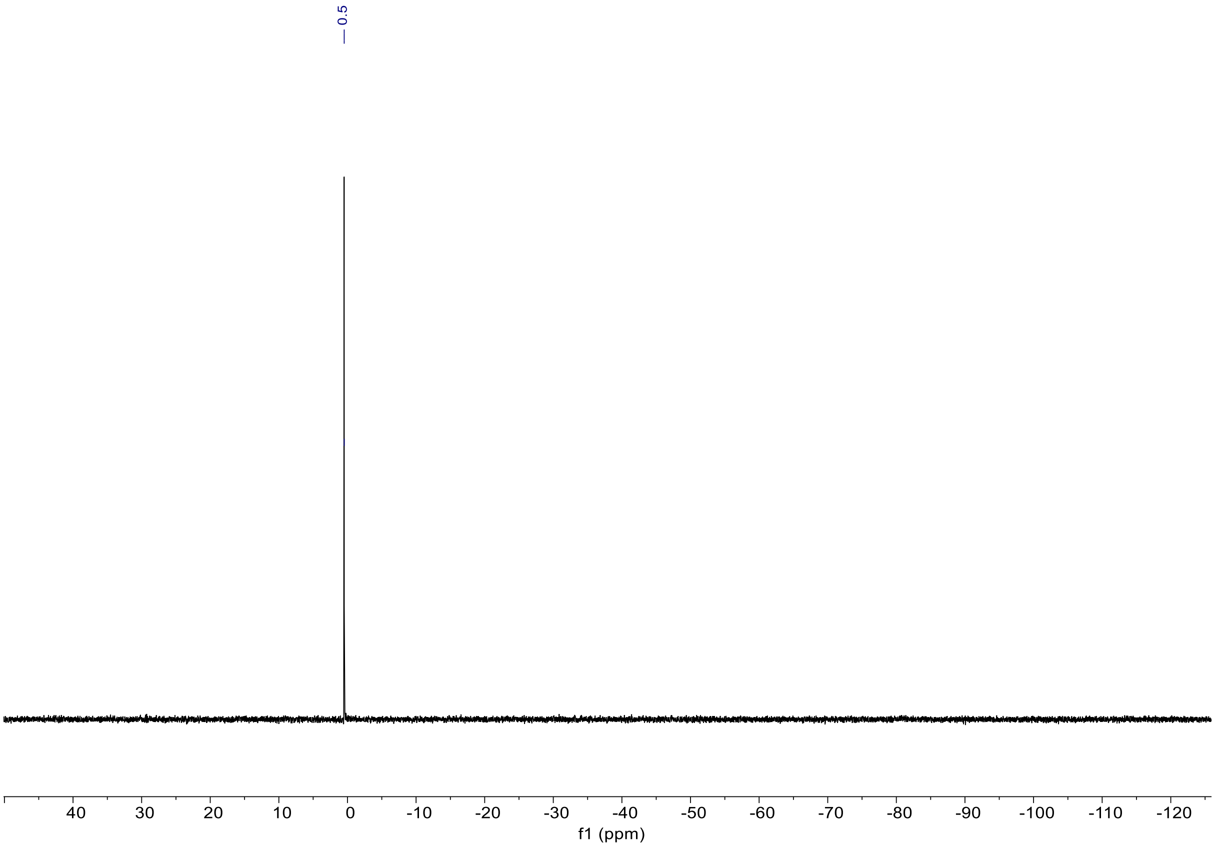

Figure S 15. ^{31}P NMR (162 MHz, C_6D_6) of 2-(Diisopropylphosphino)-*N,N*-diethylethanamine (**L5**)

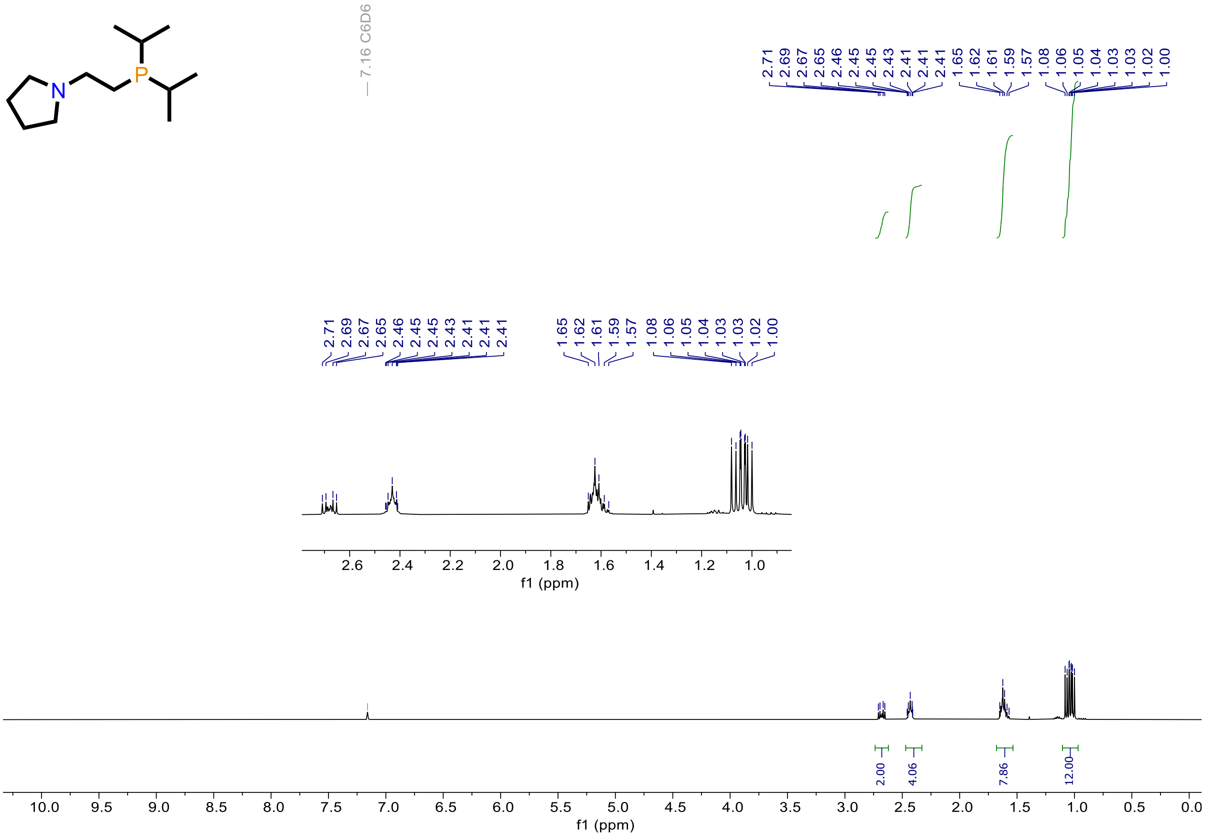

Figure S16. ^{1}H NMR (400 MHz, C_6D_6) of 1-[2-(Diisopropylphosphine)ethyl]pyrrolidine (**L6**)

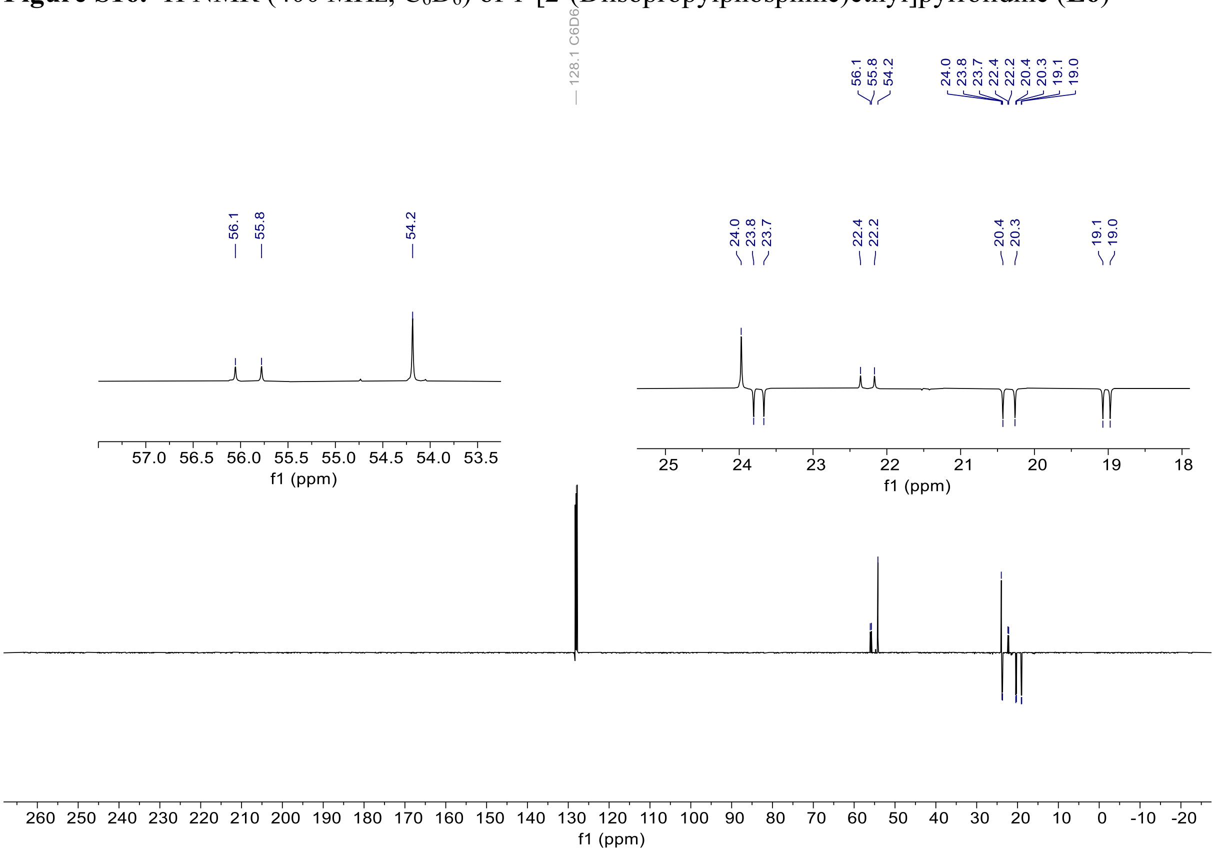

Figure S17. $^{13}C\{^{1}H\}$ NMR (101 MHz, C_6D_6) of 1-[2-(Diisopropylphosphine)ethyl]pyrrolidine (**L6**)

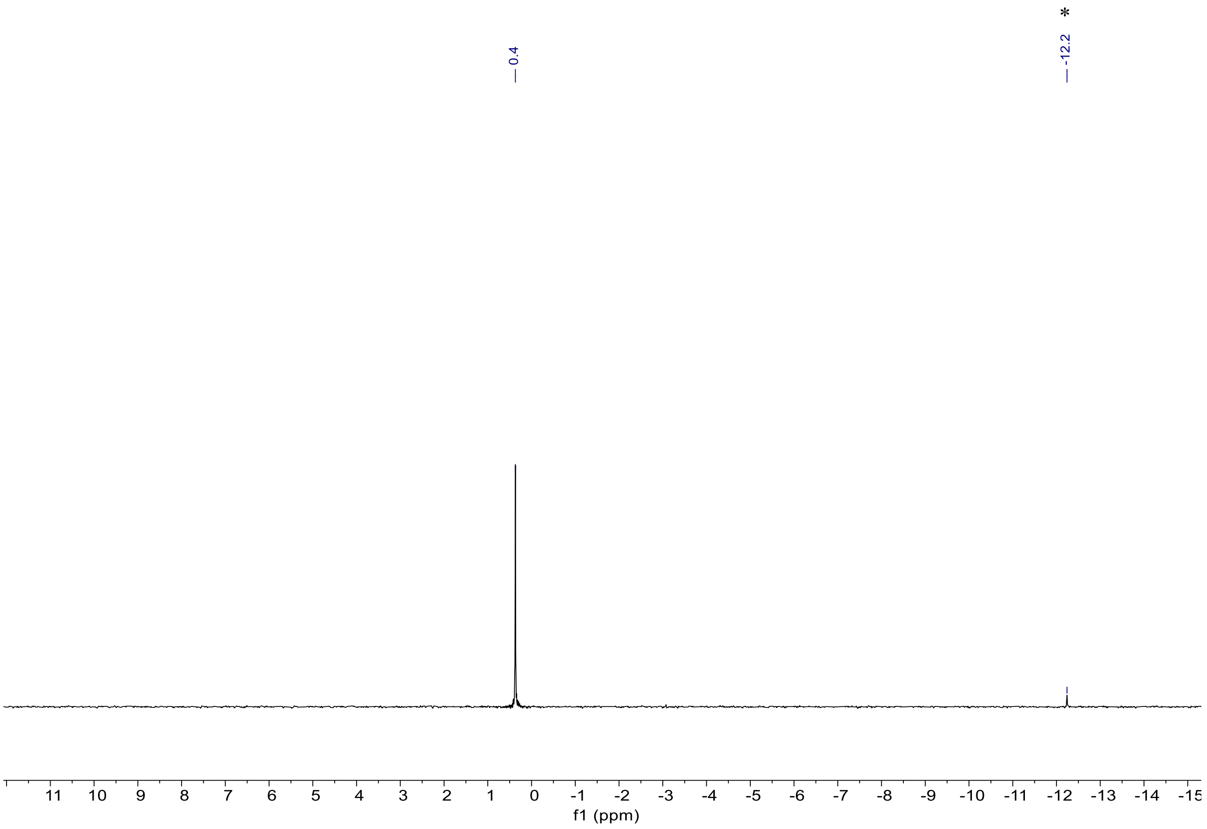

* = 1,1,2,2-Tetrakis(1-methylethyl)diphosphine

Figure S18. $^{31}P\{^{1}H\}$ NMR (162 MHz, C_6D_6) of 1-[2-(Diisopropylphosphine)ethyl]pyrrolidine (**L6**)

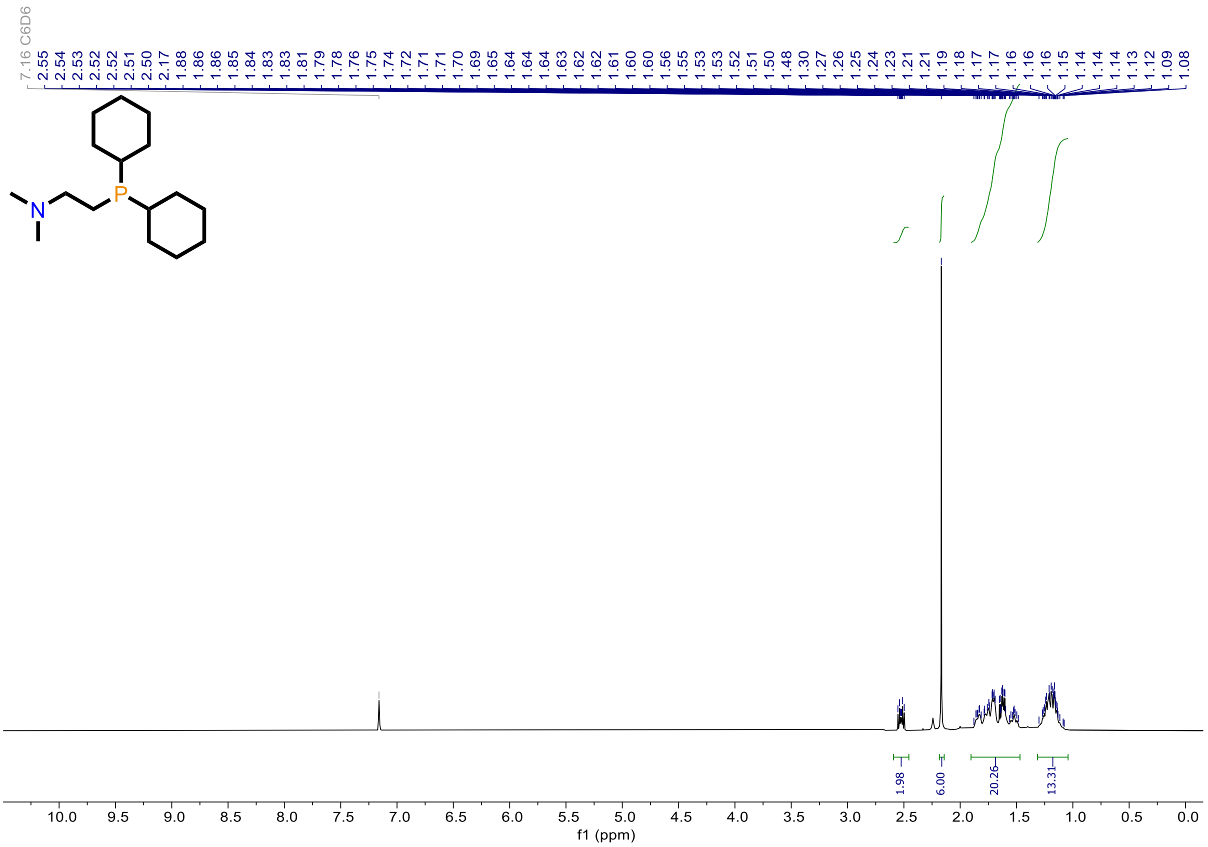

Figure S 19. ^{1}H NMR (400 MHz, C_6D_6) of 2-(Dicyclohexylphosphino)-*N, N*-dimethylethanamine (**L7**)

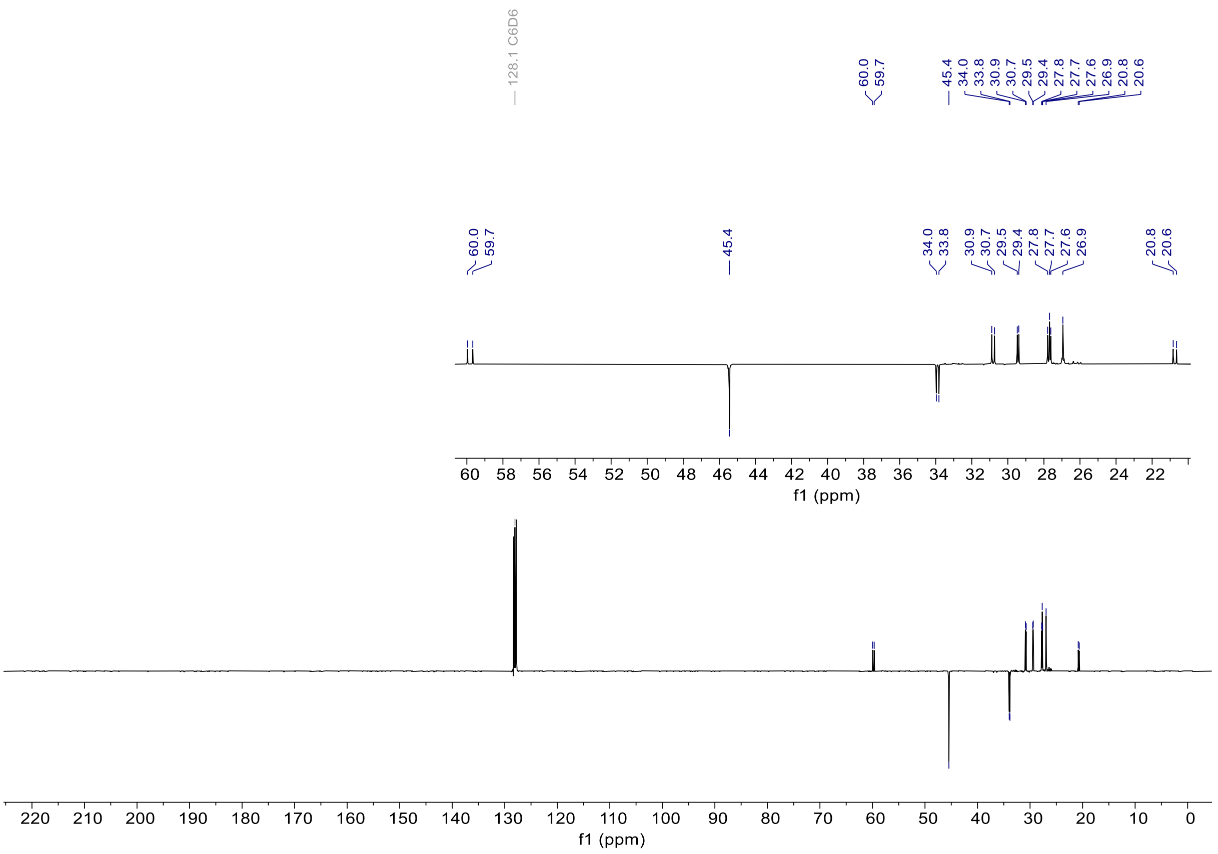

Figure S20. $^{13}C\{^{1}H\}$ NMR (101 MHz, $C^{6}D^{6}$) of 2-(Dicyclohexylphosphino)-N, N-dimethylethanamine (**L7**)

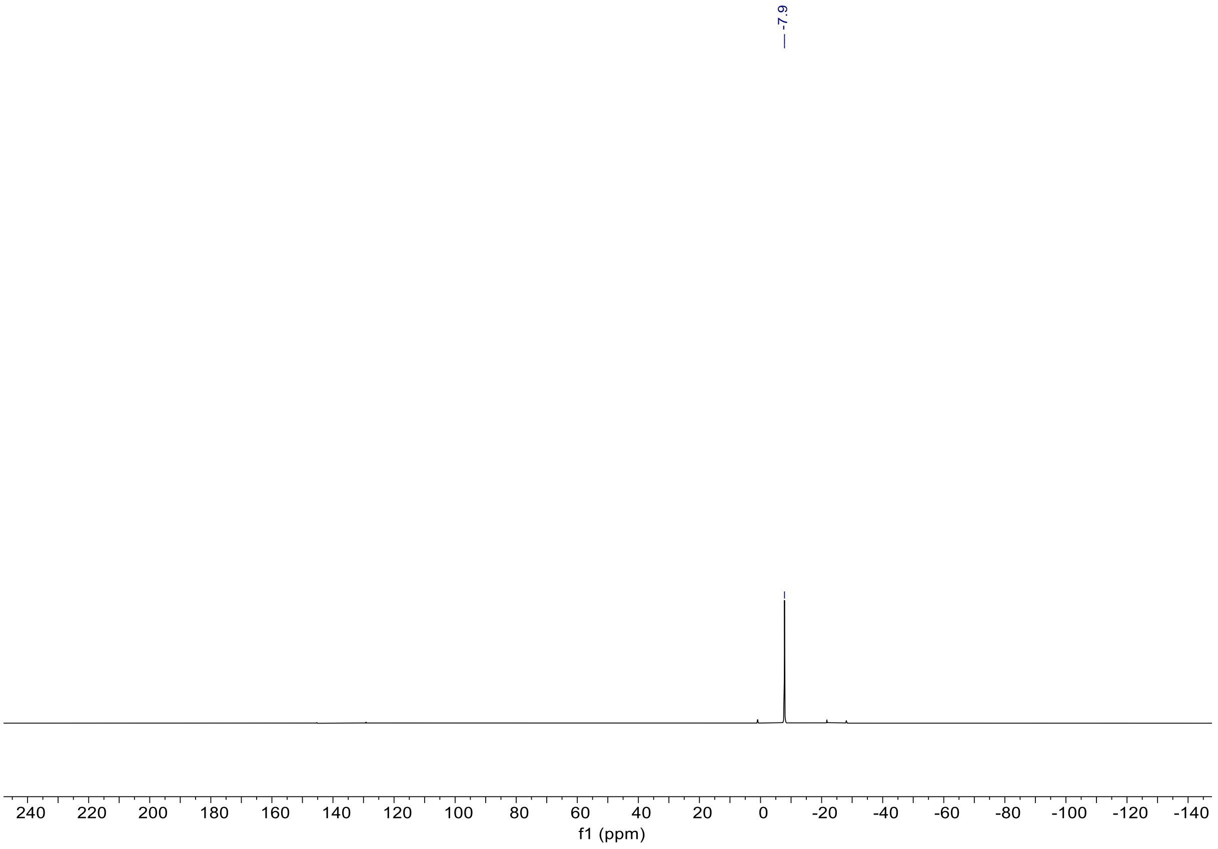

Figure S21. $^{31}P\{1H\}$ NMR (162 MHz, C_6D_6) of 2-(Dicyclohexylphosphino)-*N, N*-dimethylethanamine (**L7**)

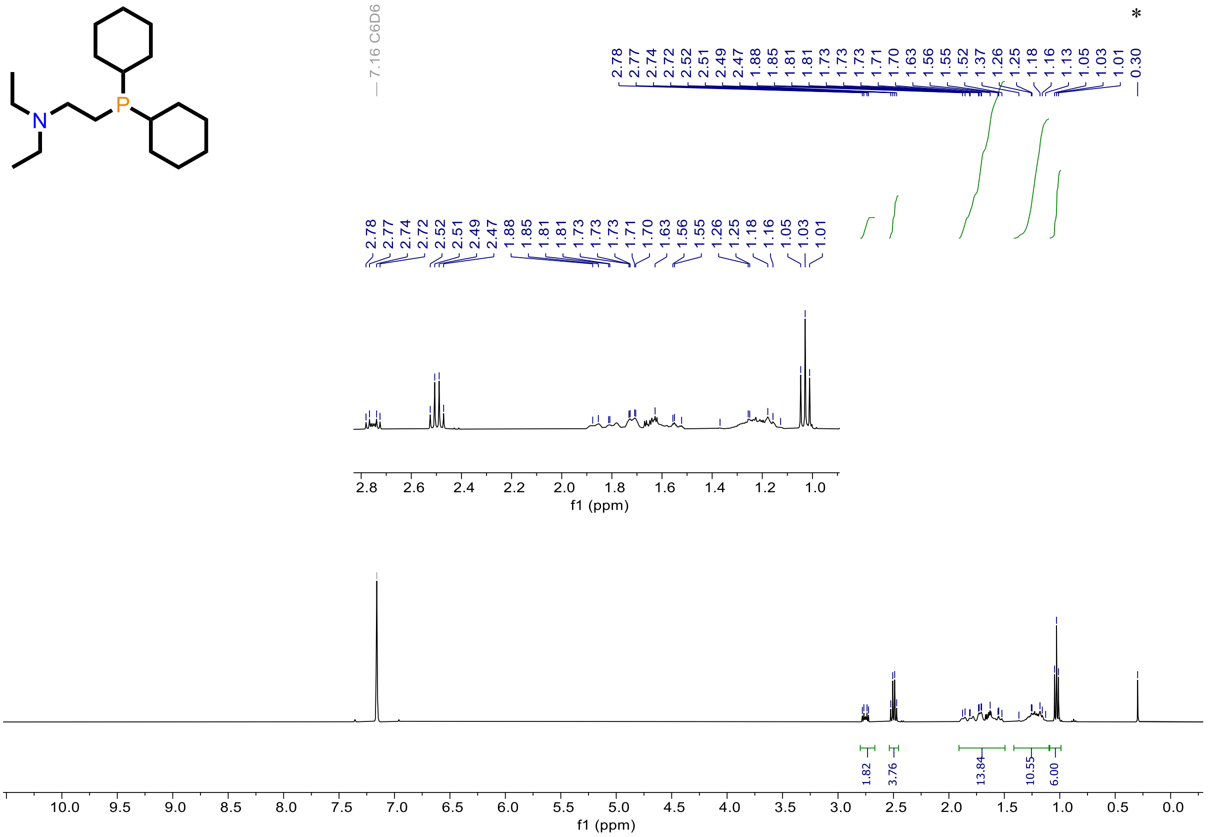

* = grease

Figure S 22. ^{1}H NMR (400 MHz, C_6D_6) of 2-(Dicyclohexylphosphino)-*N,N*-diethylethanamine (**L8**)

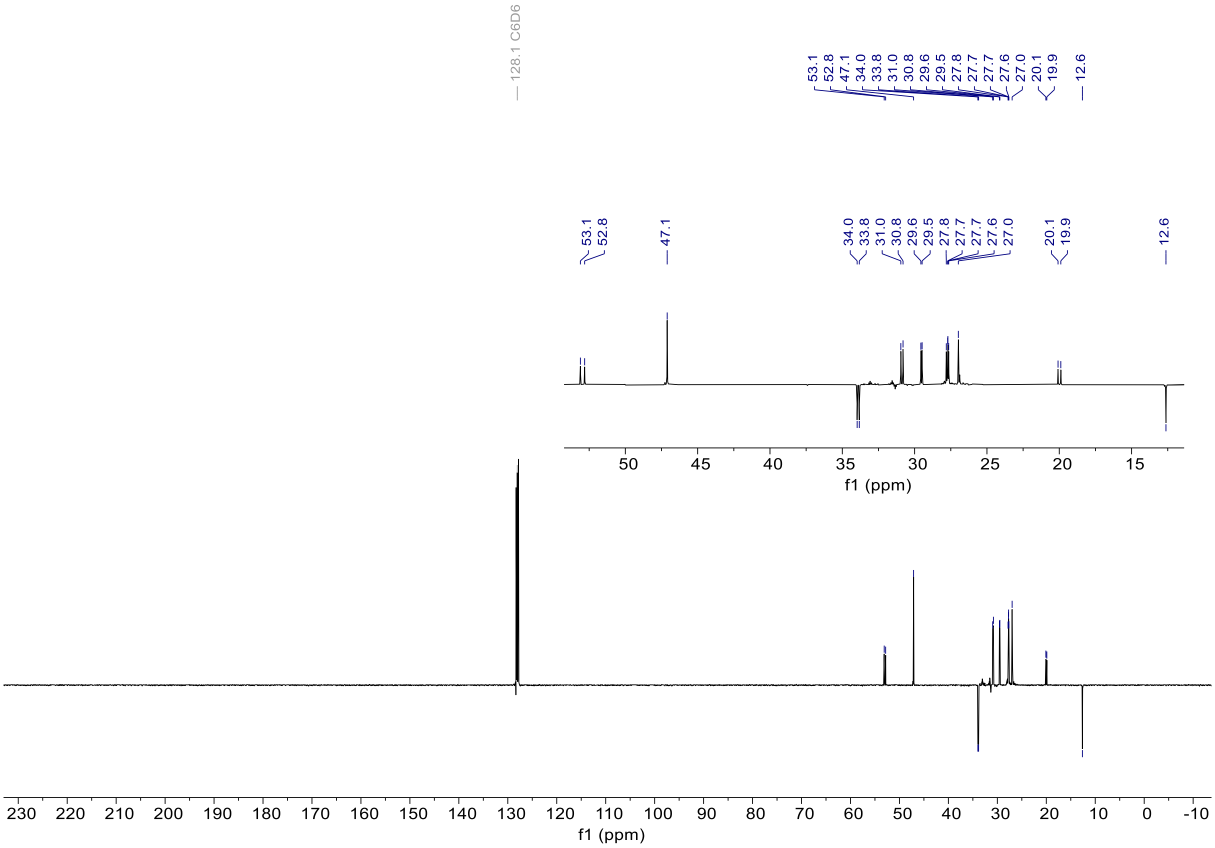

Figure S23. ^{13}C{^{1}H} NMR (101 MHz, C_6D_6) of 2-(Dicyclohexylphosphino)-*N,N*-diethylethanamine (**L8**)

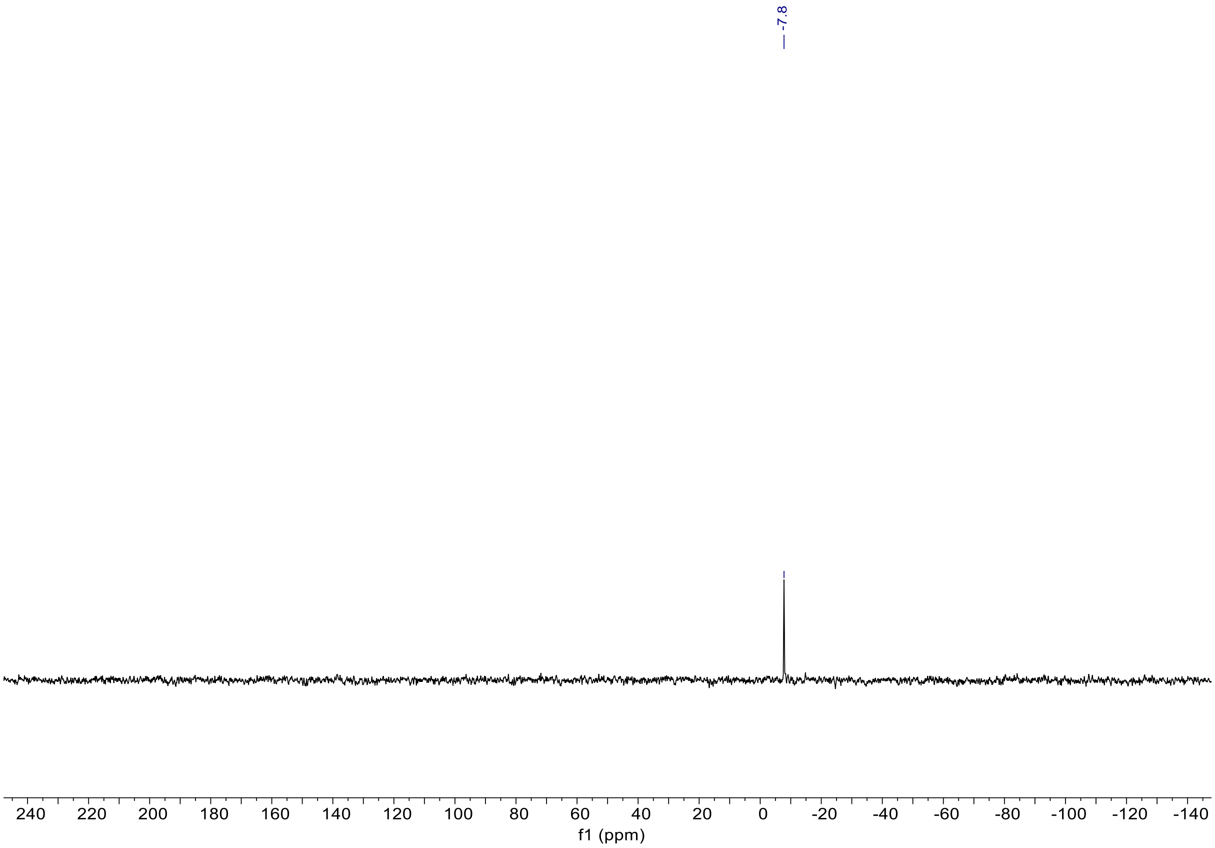

Figure S24. $^{31}P\{^{1}H\}$ NMR (162 MHz, C_6D_6) of 2-(Dicyclohexylphosphino)-*N,N*-diethylethanamine (**L8**)

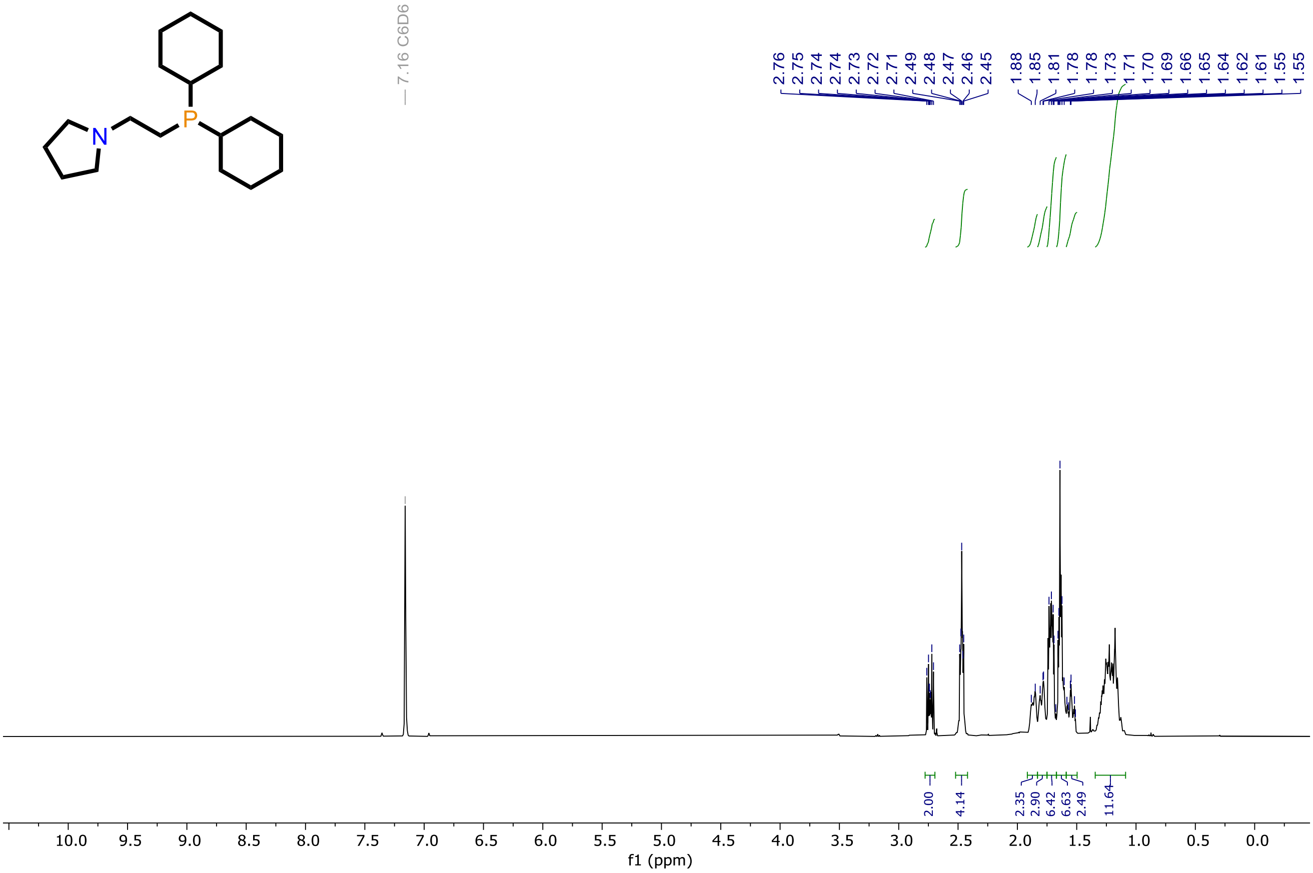

Figure S25. ^{1}H NMR (400 MHz, C_6D_6) of 1-[2-(Dipcyclohexylsphino)ethyl]pyrrolidine (**L9**)

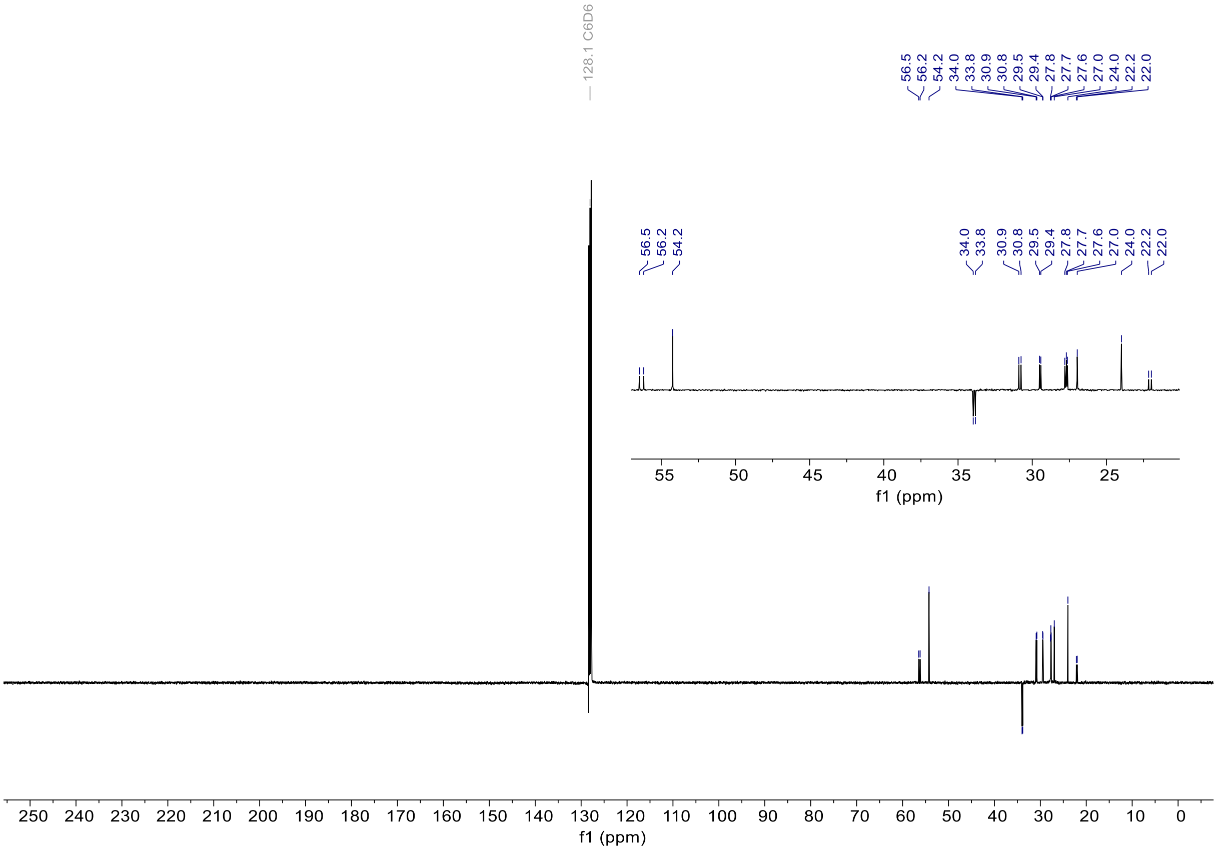

Figure S26. ^{13}C NMR (101 MHz, C_6D_6) of 1-[2-(Dipcyclohexylsphino)ethyl]pyrrolidine (**L9**)

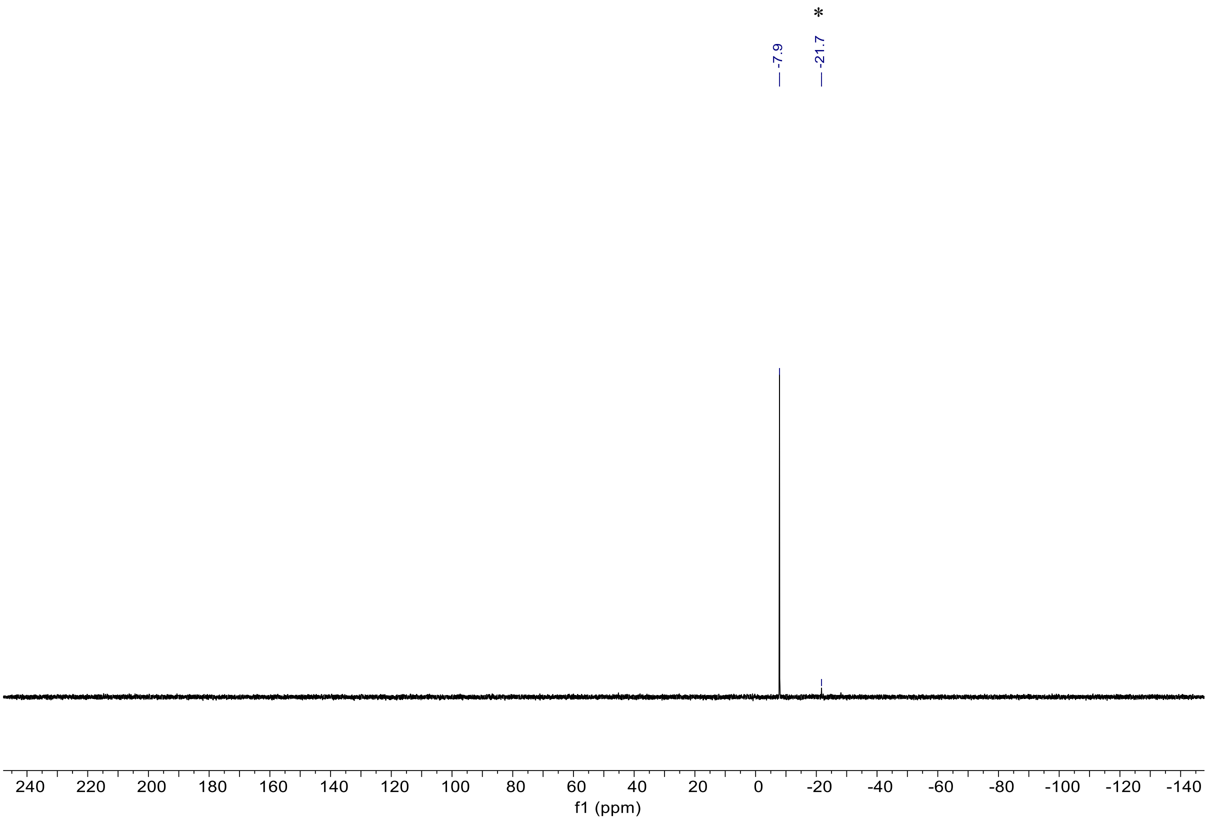

* = 1,1,2,2-Tetracyclohexyldiphosphine

Figure S27. $^{31}P\{^1H\}$ NMR (162 MHz, C_6D_6) of 1-[2-(Dipcyclohexylsphino)ethyl]pyrrolidine (**L9**)

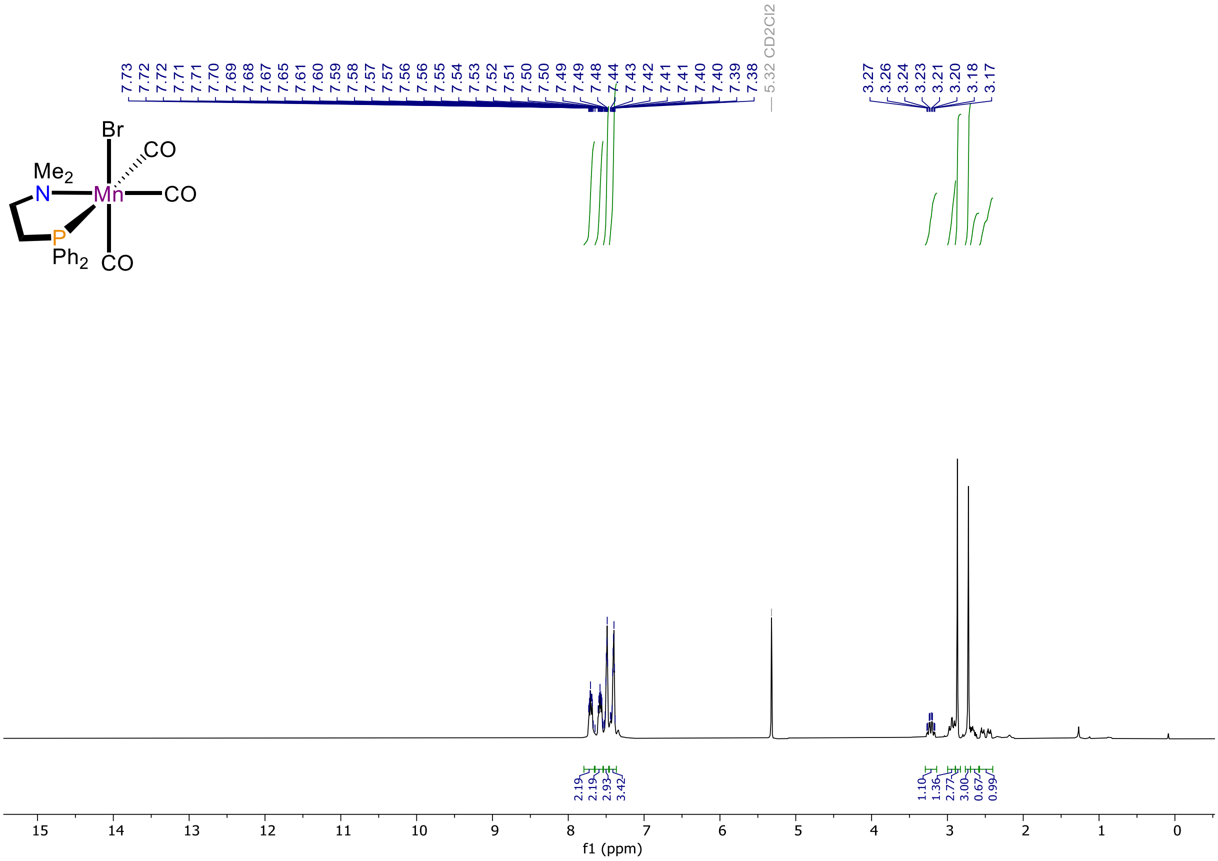

Figure S28. ^{1}H NMR (400 MHz, CD_2Cl_2) of *fac*-[Mn($P^{Ph}N^{Me}$)(CO)$_3$Br] (**1a**)

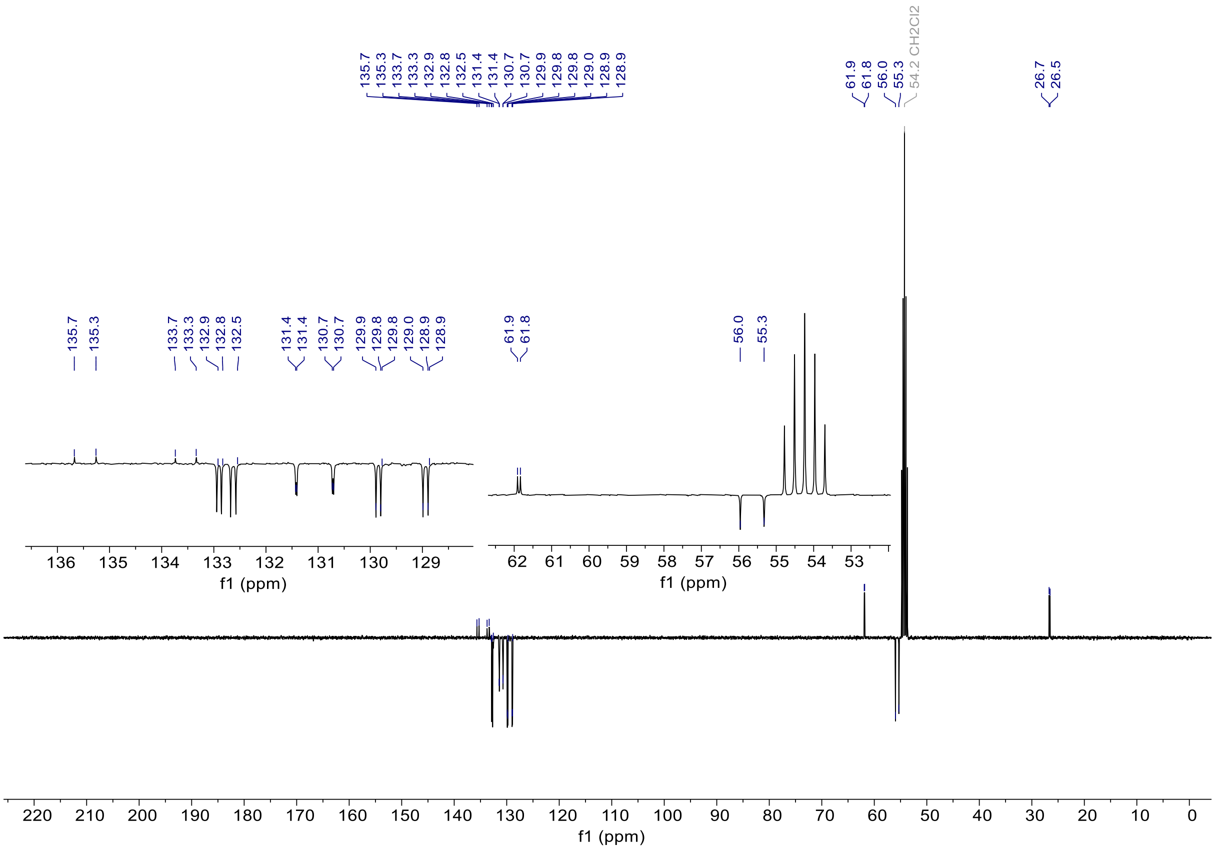

Figure S29. $^{13}C\{^{1}H\}$ NMR (101 MHz, CD_2Cl_2) of *fac*-[Mn($P^{Ph}N^{Me}$)(CO)$_3$Br] (**1a**)

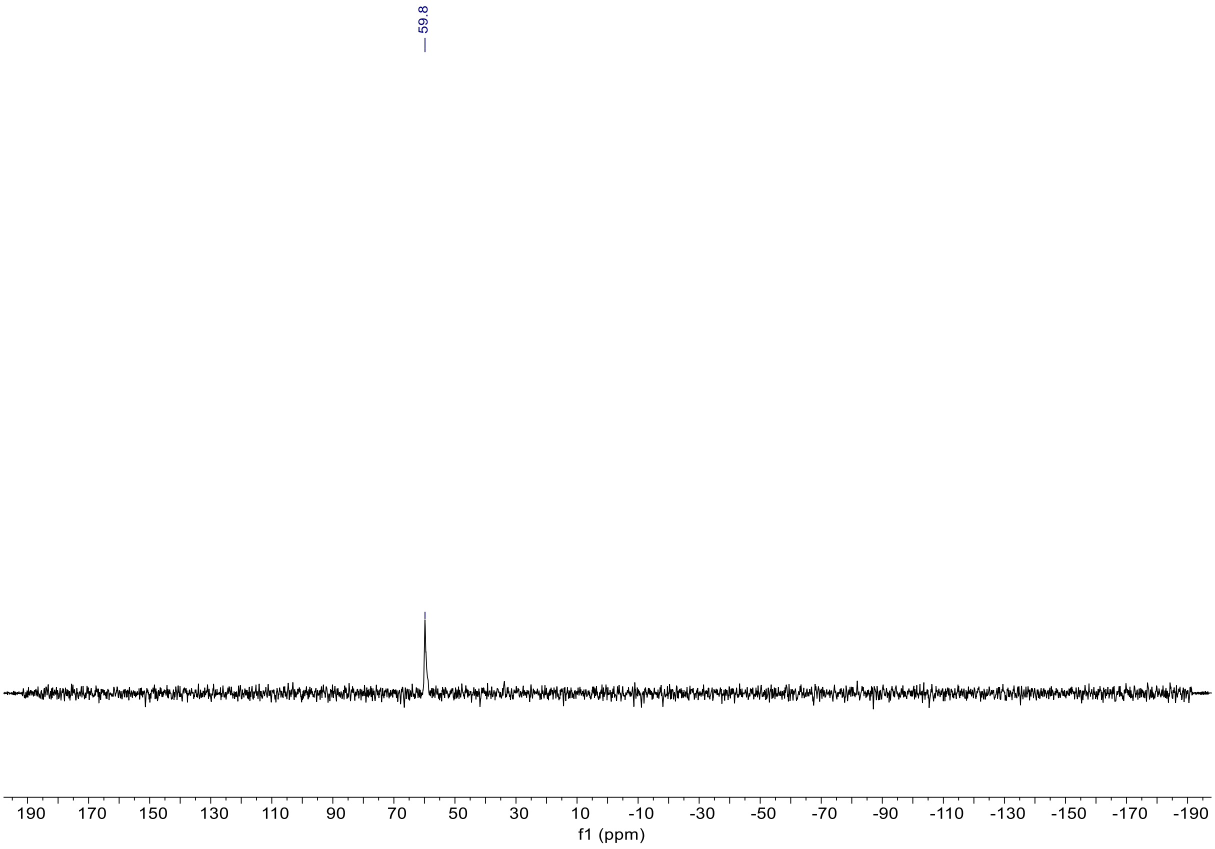

Figure S30. $^{31}P\{^{1}H\}$ NMR (162 MHz, CD_2Cl_2) of *fac*-[Mn($P^{Ph}N^{Me}$)$(CO)_3$Br] (**1a**)

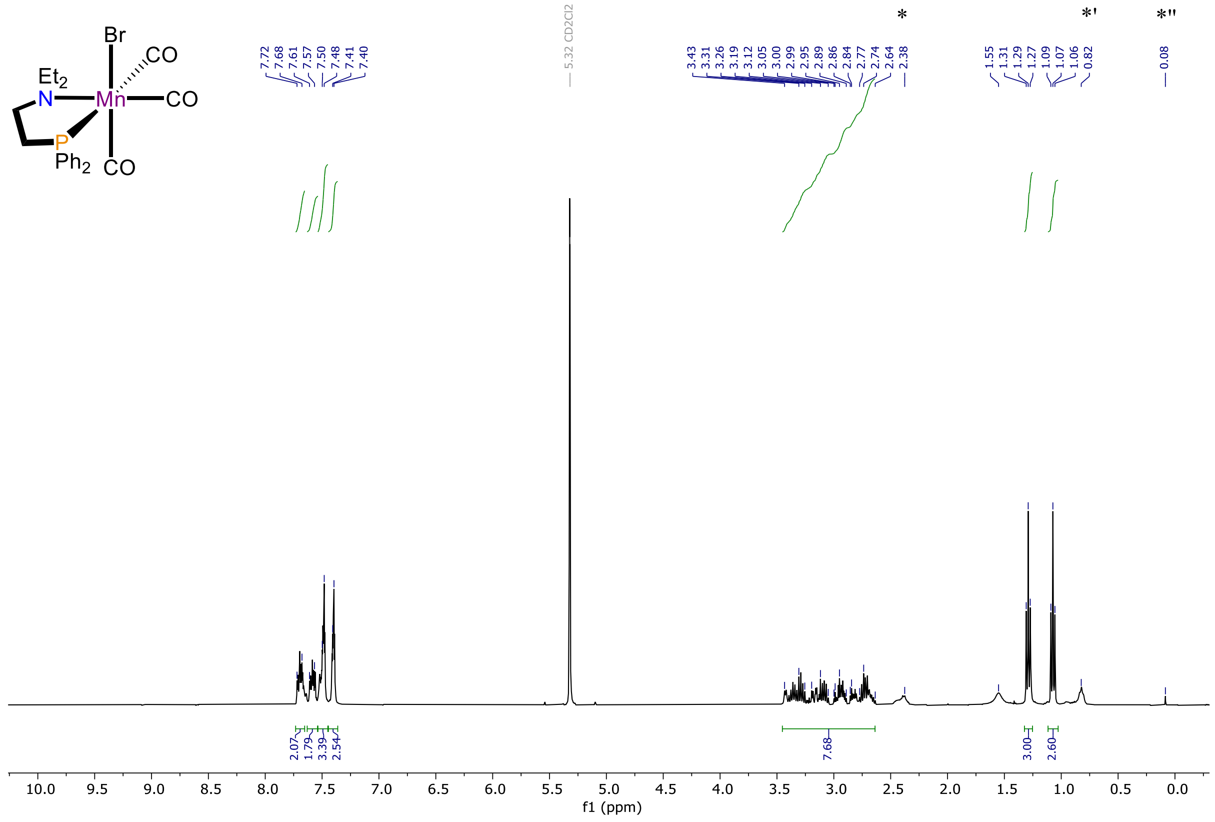

* = toluene, *' = *n*-pentane, *'' = grease

Figure S31. ^{1}H NMR (400 MHz, CD_2Cl_2) of *fac*-[Mn($P^{Ph}N^{Et}$)$(CO)_3$Br] (**1b**)

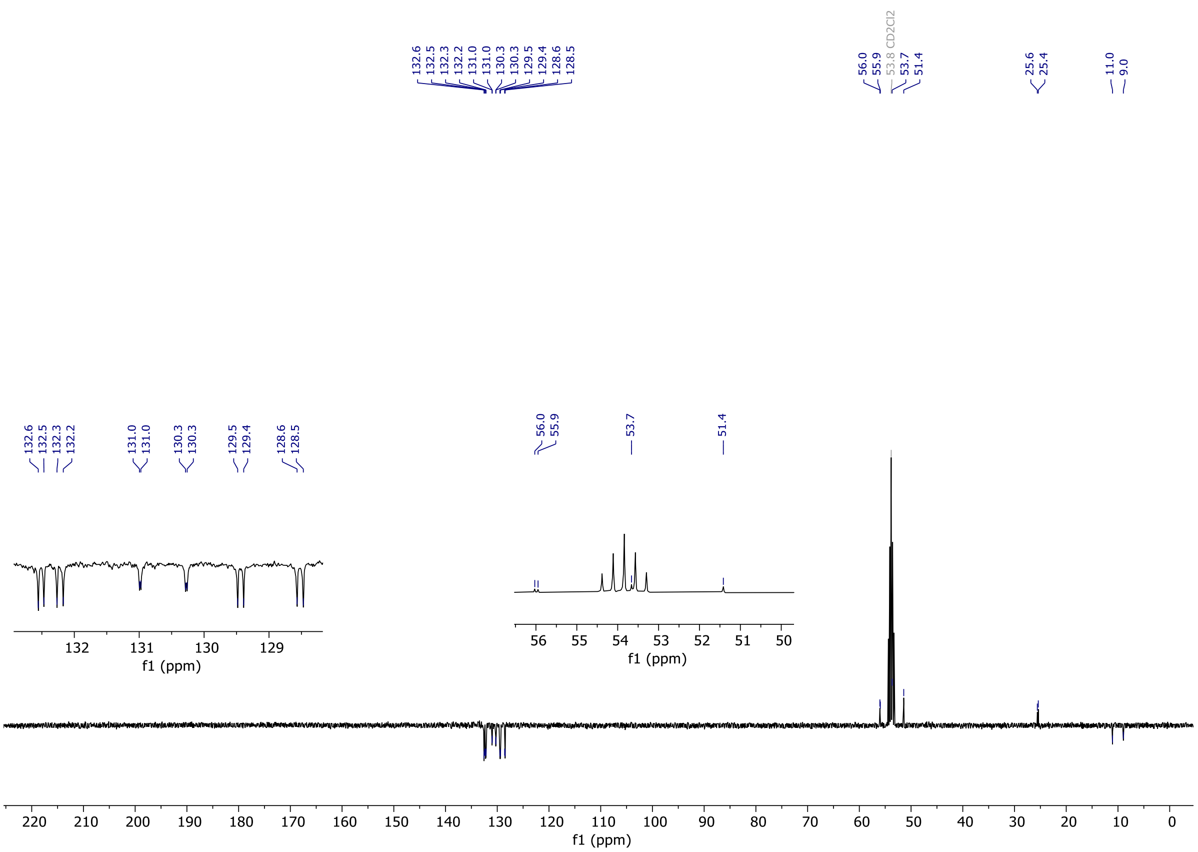

Figure S32. $^{13}C\{^{1}H\}$ NMR (101 MHz, CD_2Cl_2) of *fac*-[Mn($P^{Ph}N^{Et}$)(CO)$_3$Br] (**1b**)

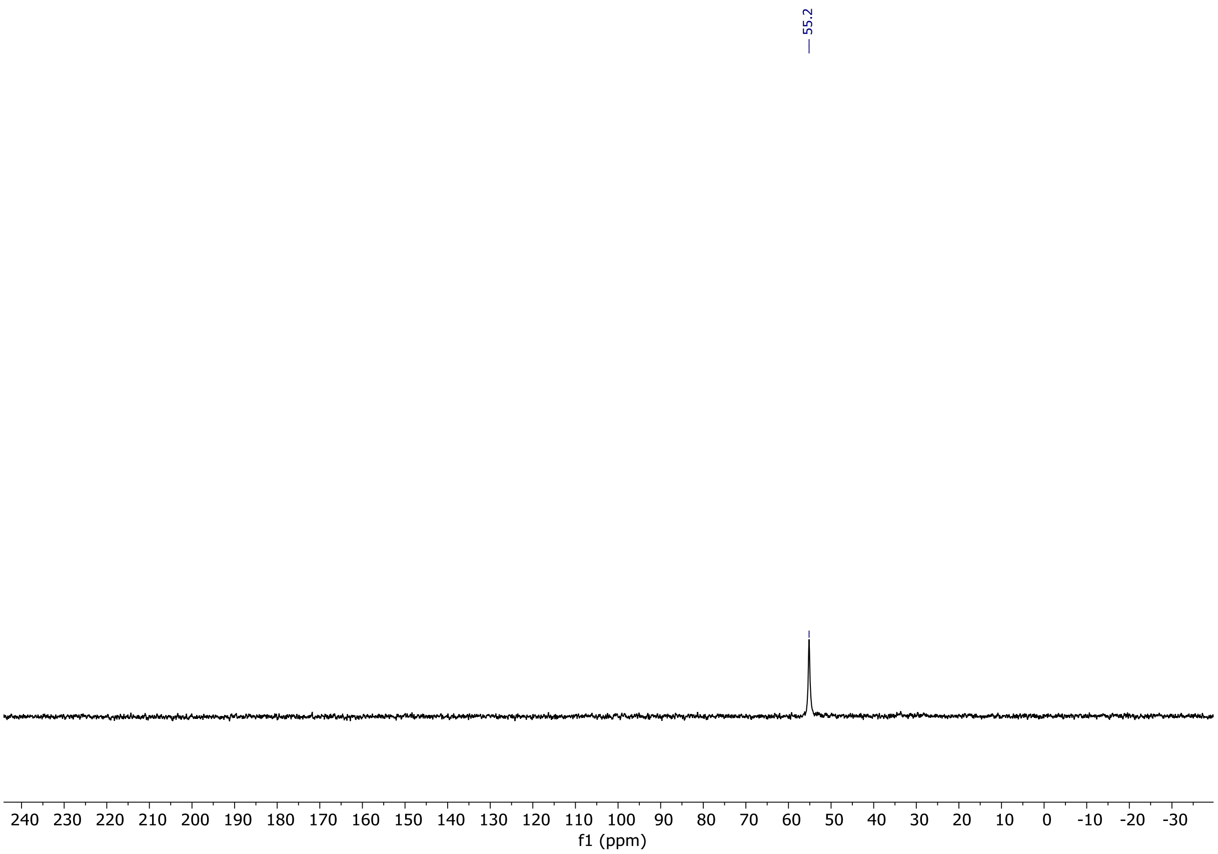

Figure S33. $^{31}P\{^{1}H\}$ NMR (162 MHz, CD_2Cl_2) of *fac*-[Mn($P^{Ph}N^{Et}$)(CO)$_3$Br] (**1b**)

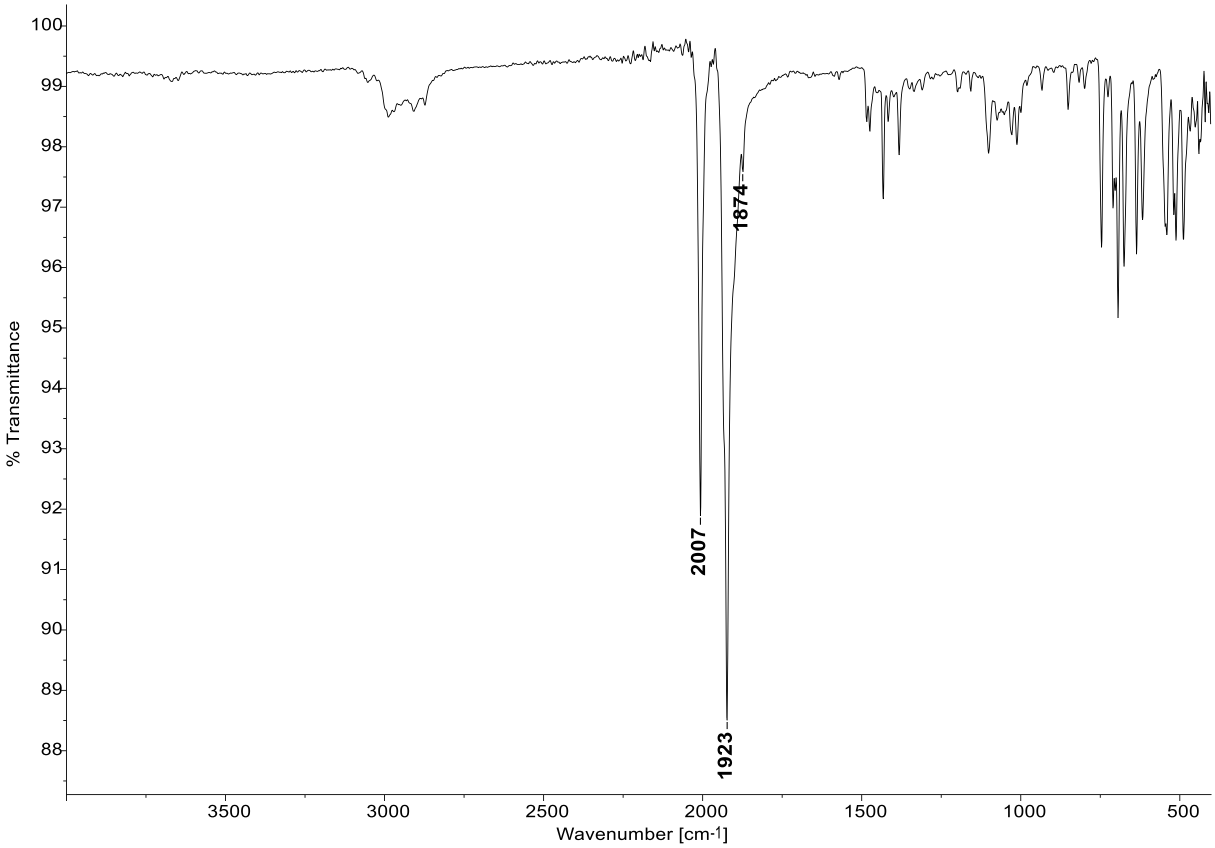

Figure S34. IR (ATR, cm^{-1}) of *fac*-[Mn($P^{Ph}N^{Et}$)$(CO)_3$Br] (**1b**)

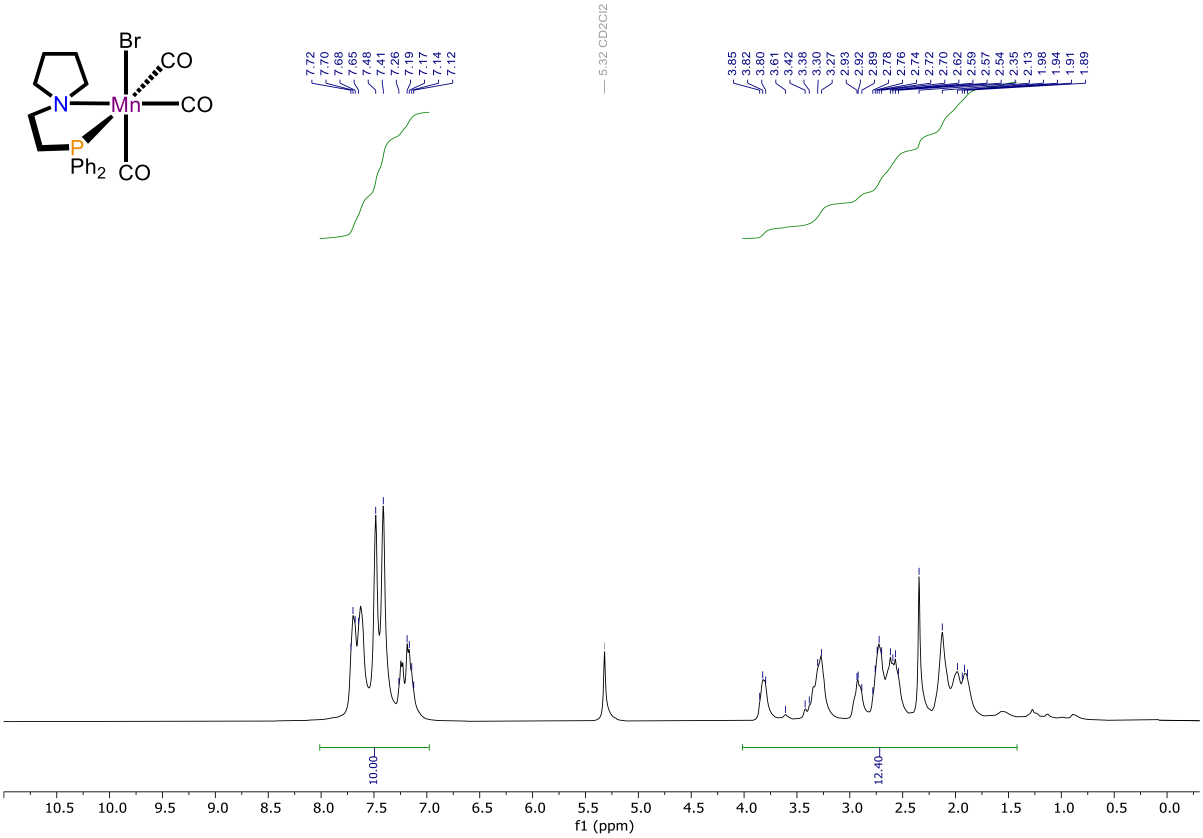

Figure S35. ^{1}H NMR (400 MHz, CD_2Cl_2) of *fac*-[Mn($P^{Ph}N^{Pyrr}$)$(CO)_3$Br] (**1c**)

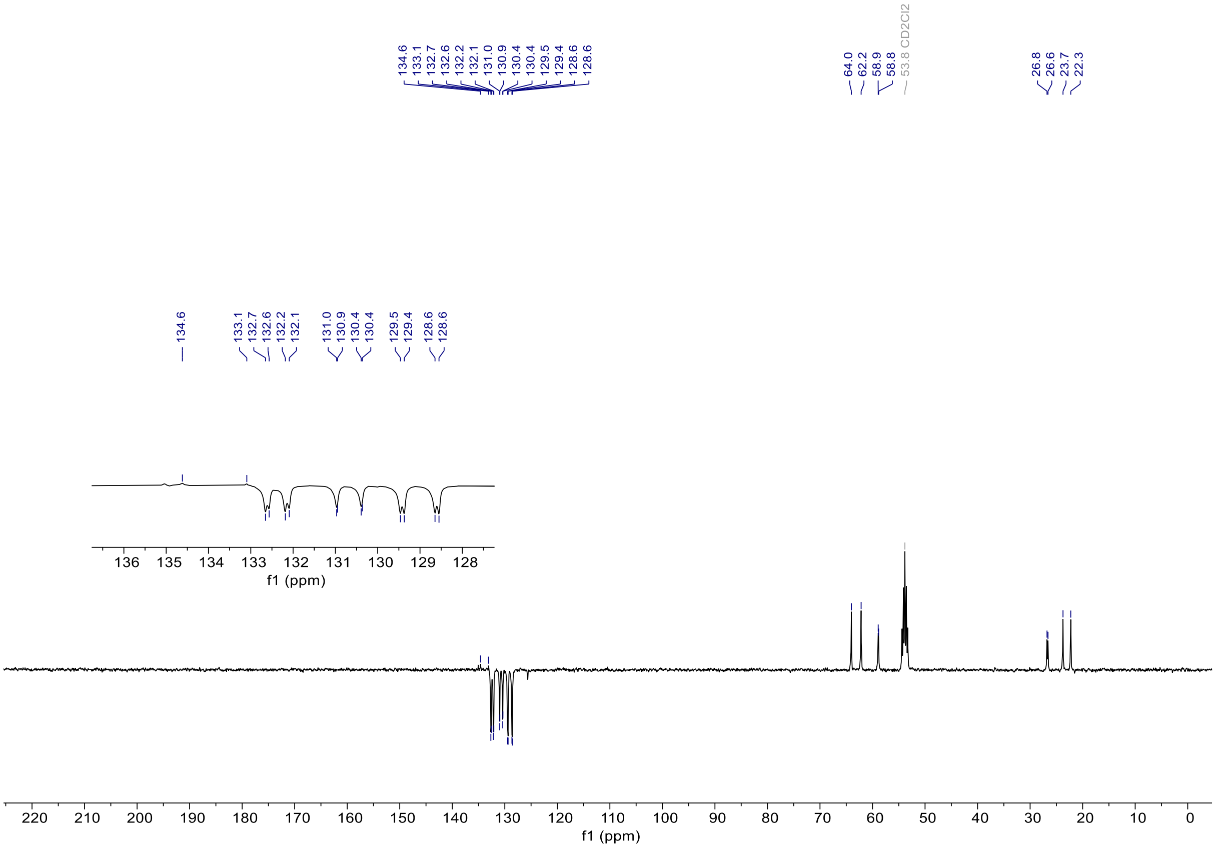

Figure S36. $^{13}C\{^1H\}$ NMR (101 MHz, CD_2Cl_2) of *fac*-[Mn($P^{Ph}N^{Pyrr}$)$(CO)_3$Br] (**1c**)

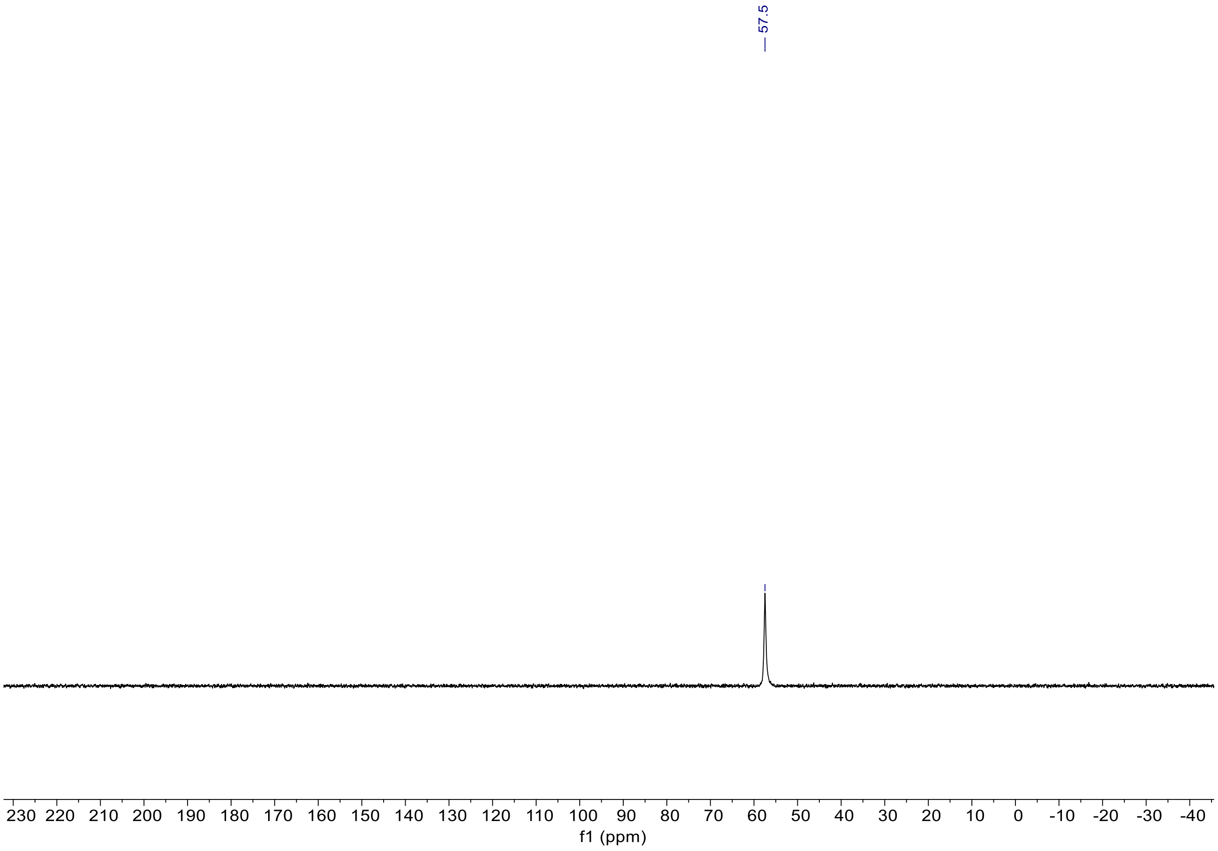

Figure S37. $^{31}P\{^1H\}$ NMR (162 MHz, CD_2Cl_2) of *fac*-[Mn($P^{Ph}N^{Pyrr}$)$(CO)_3$Br] (**1c**)

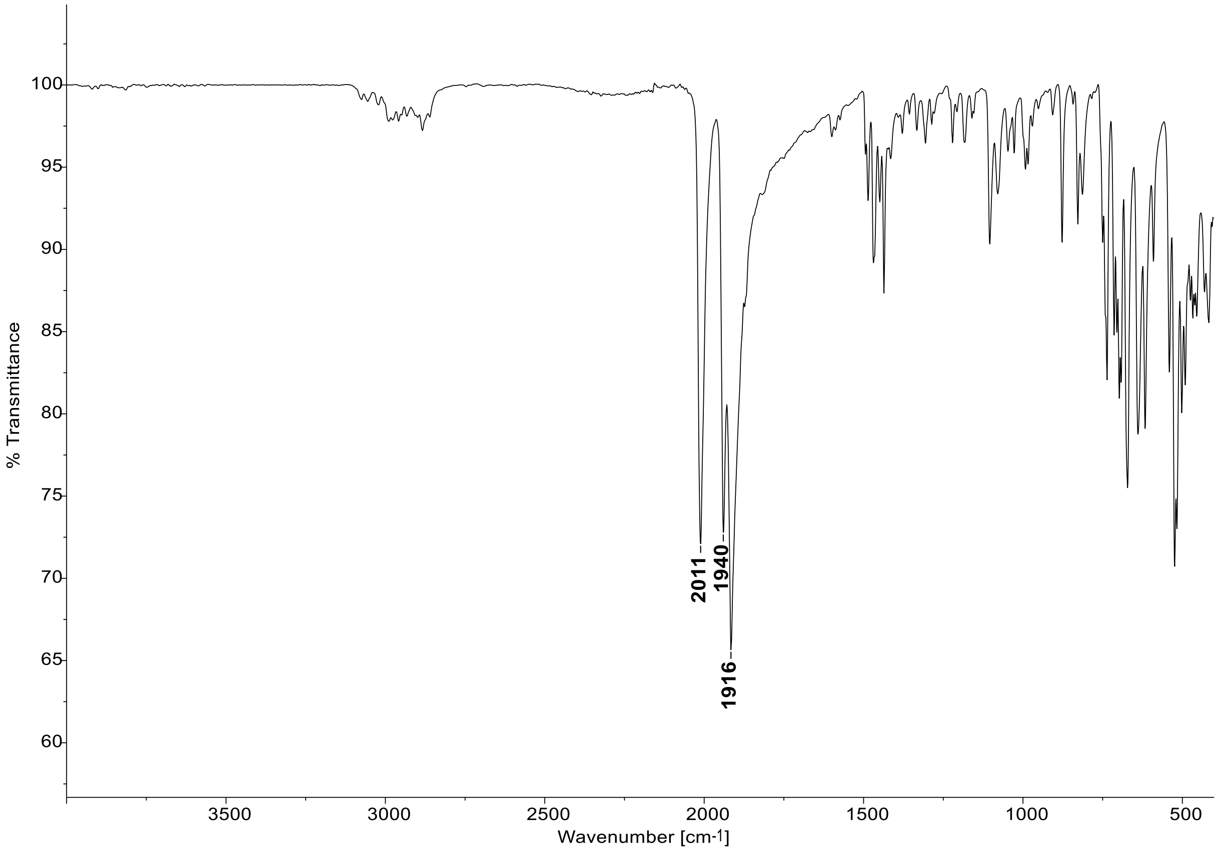

Figure S38. IR (ATR, cm^{-1}) of *fac*-[Mn($P^{Ph}N^{Pyrr}$)$(CO)_3$Br] (**1c**)

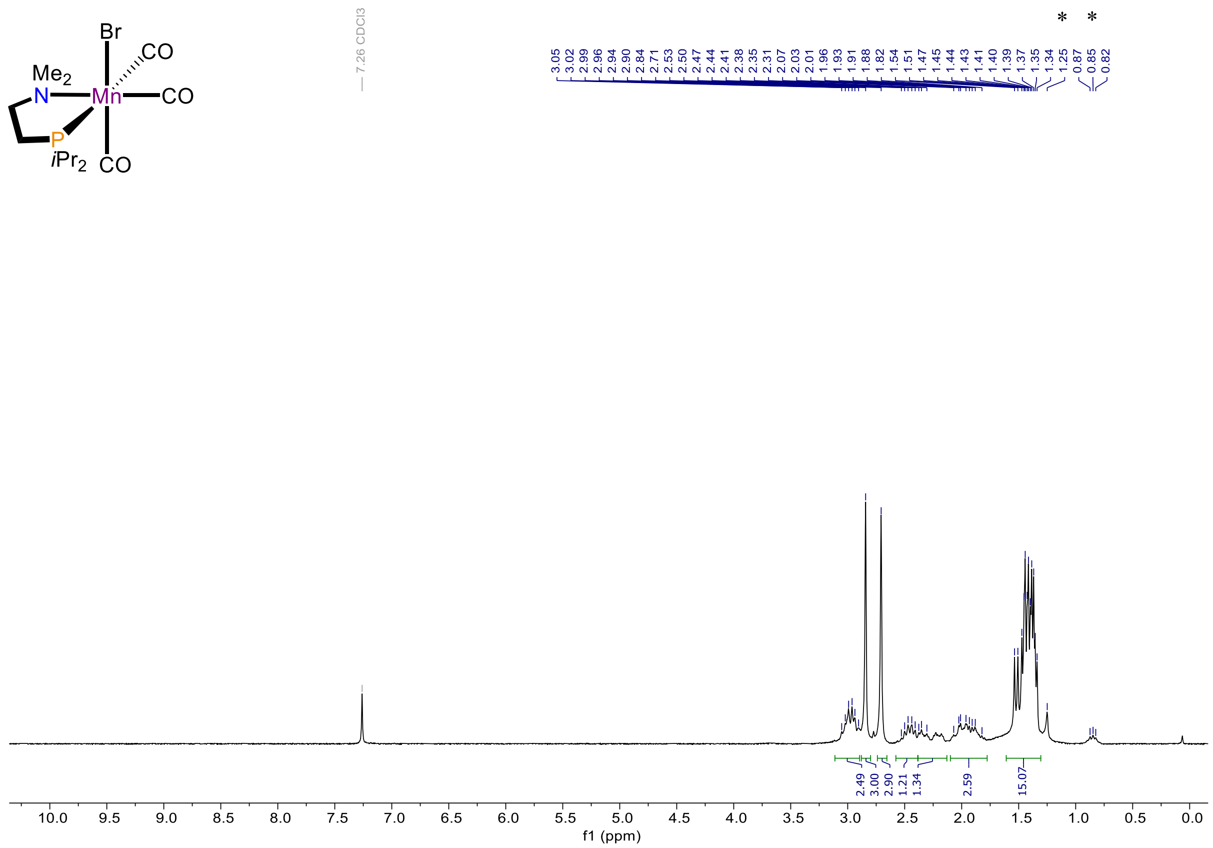

* = *n*-pentane

Figure S39. ^{1}H NMR (400 MHz, CD_2Cl_2) of *fac*-[Mn($P^{iPr}N^{Me}$)$(CO)_3$Br] (**1d**)

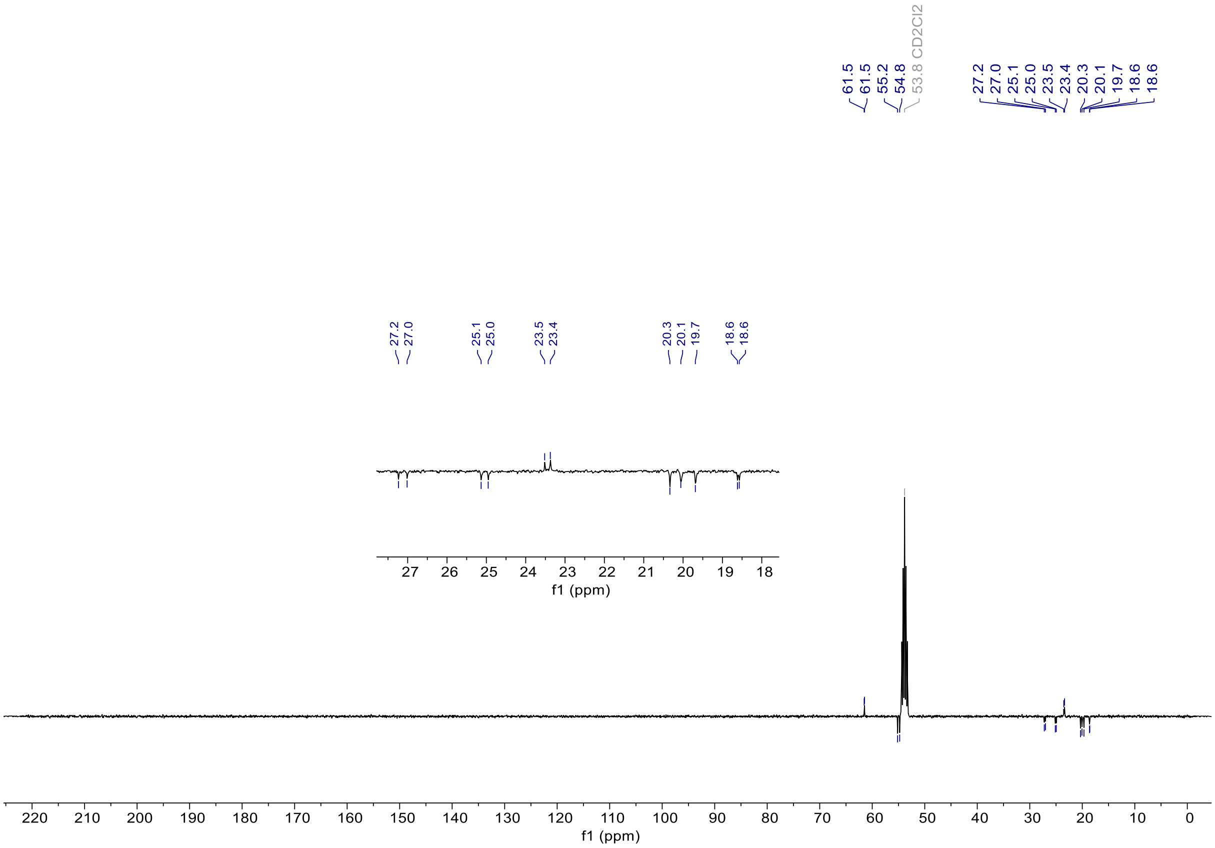

Figure S40. $^{13}C\{^1H\}$ NMR (101 MHz, CD_2Cl_2) of *fac*-[Mn($P^{iPr}N^{Me}$)(CO)$_3$Br] (**1d**)

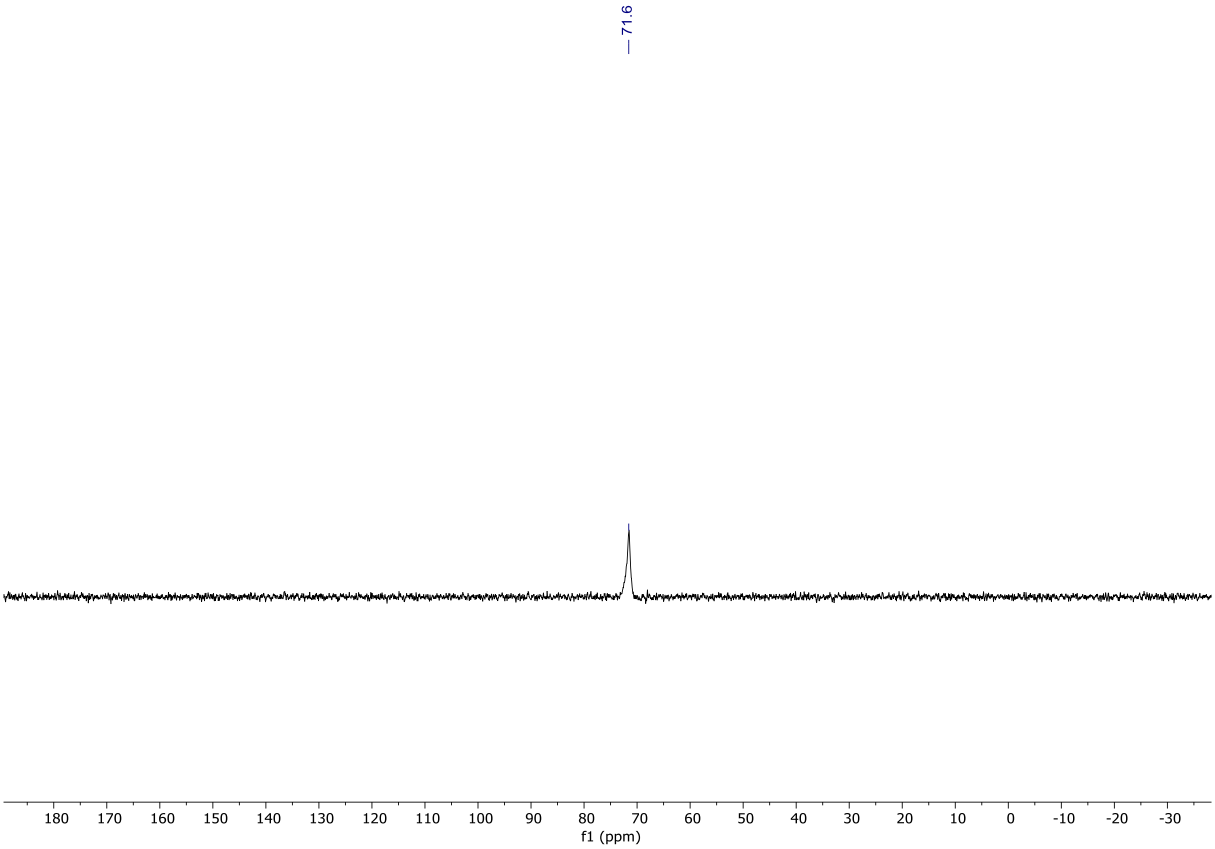

Figure S41. $^{31}P\{^1H\}$ NMR (162 MHz, CD_2Cl_2) of *fac*-[Mn($P^{iPr}N^{Me}$)(CO)$_3$Br] (**1d**)

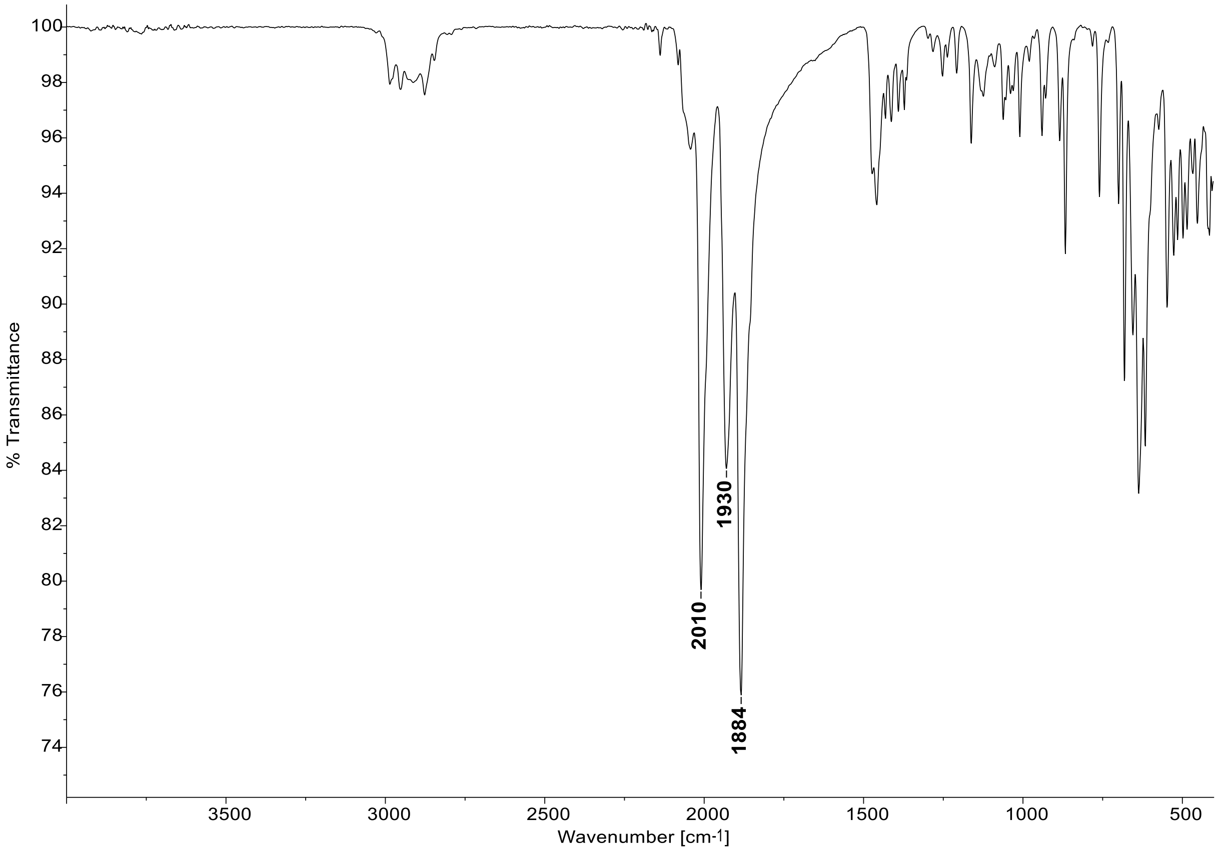

Figure S42. IR (ATR, cm^{-1}) of *fac*-[Mn(P^{iPr}N^{Me})(CO)$_3$Br] (**1d**)

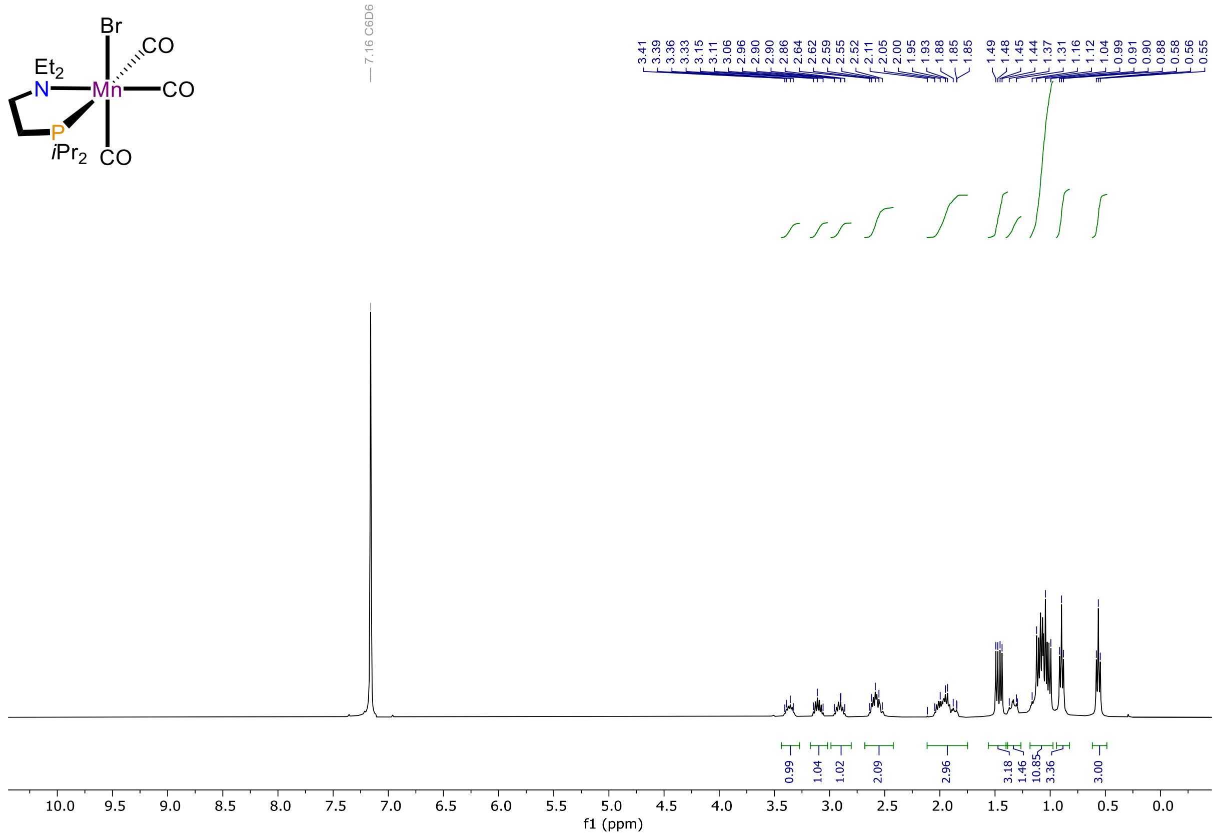

Figure S43. ^{1}H NMR (400 MHz, C_6D_6) of *fac*-[Mn(P^{iPr}N^{Et})(CO)$_3$Br] (**1e**)

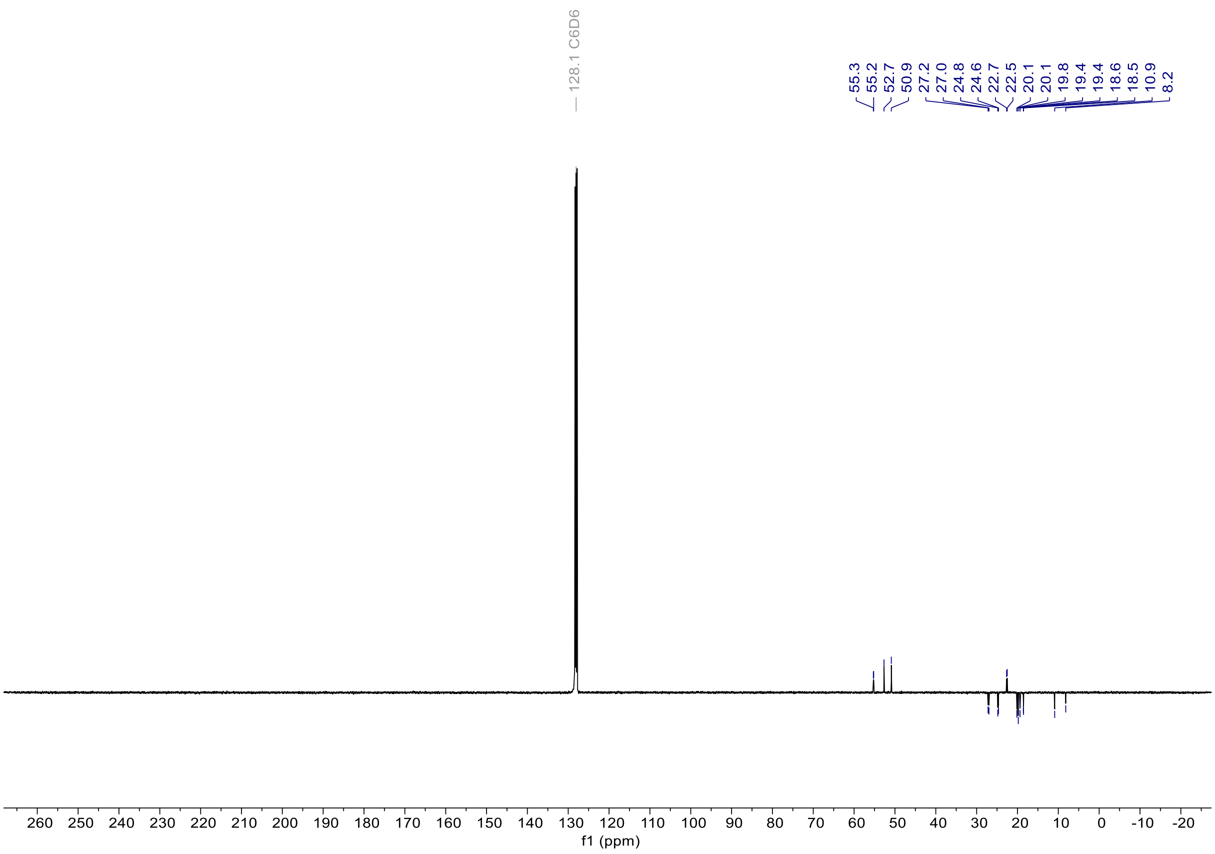

Figure S44. $^{13}C\{^{1}H\}$ NMR (101 MHz, C_6D_6) of *fac*-[Mn($P^{iPr}N^{Et}$)$(CO)_3$Br] (**1e**)

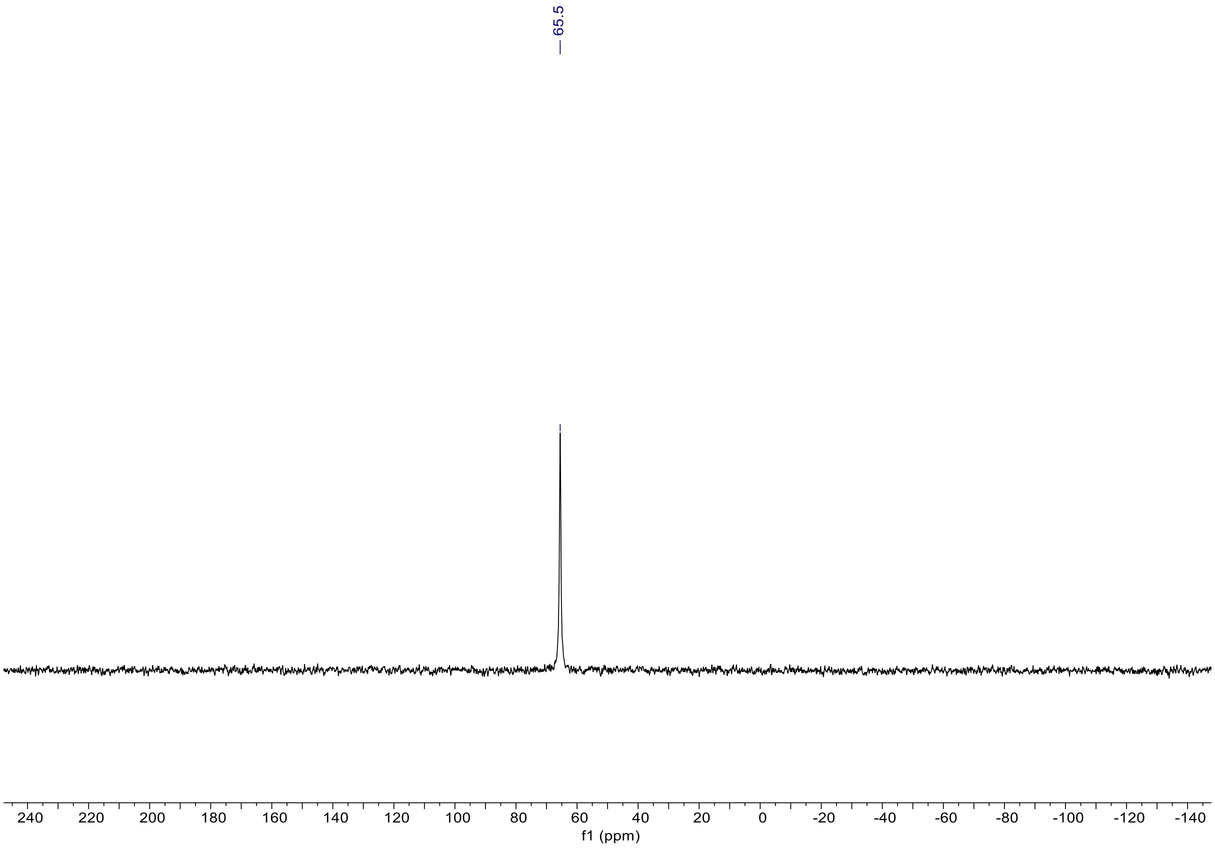

Figure S45. $^{31}P\{^{1}H\}$ NMR (162 MHz, CD_2Cl_2) of *fac*-[Mn($P^{iPr}N^{Et}$)$(CO)_3$Br] (**1e**)

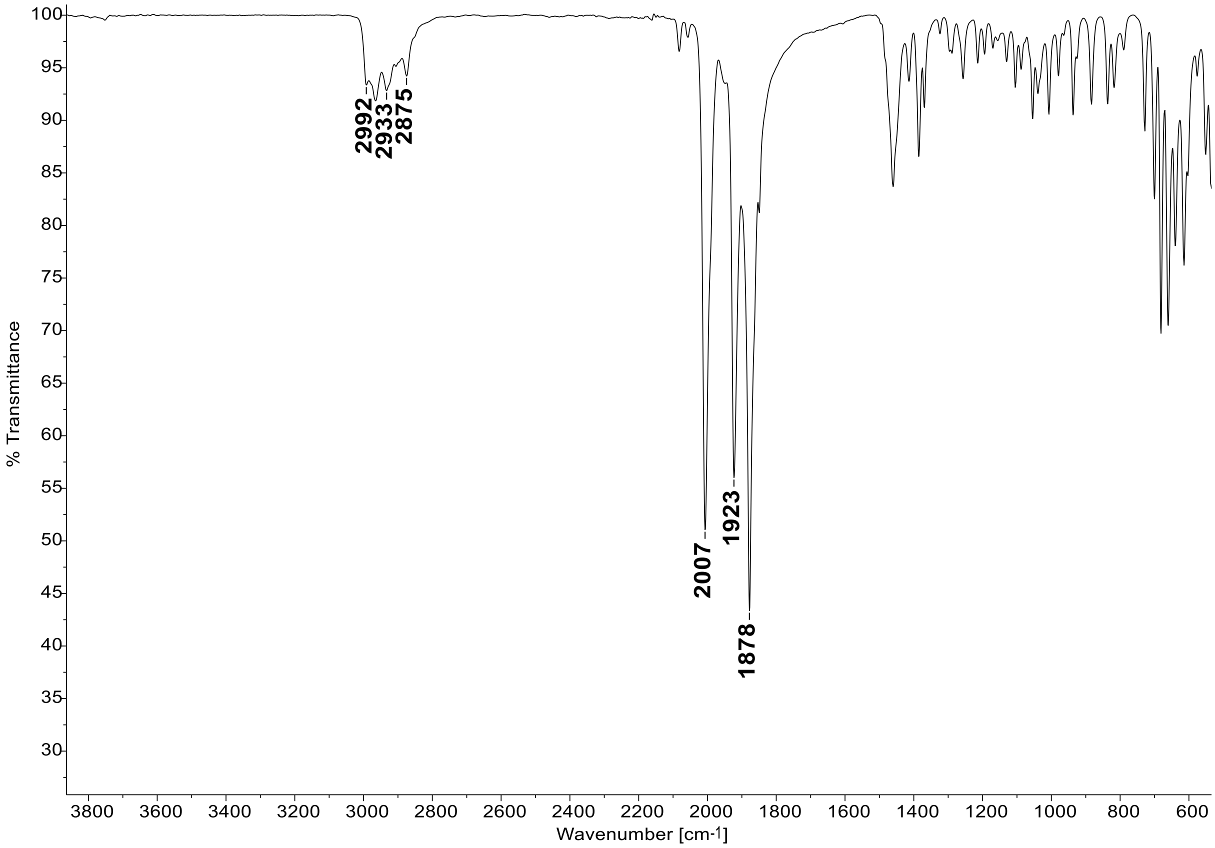

Figure S46. IR (ATR, cm^{-1}) of *fac*-[Mn($P^{iPr}N^{Et}$)$(CO)_3$Br] (**1e**)

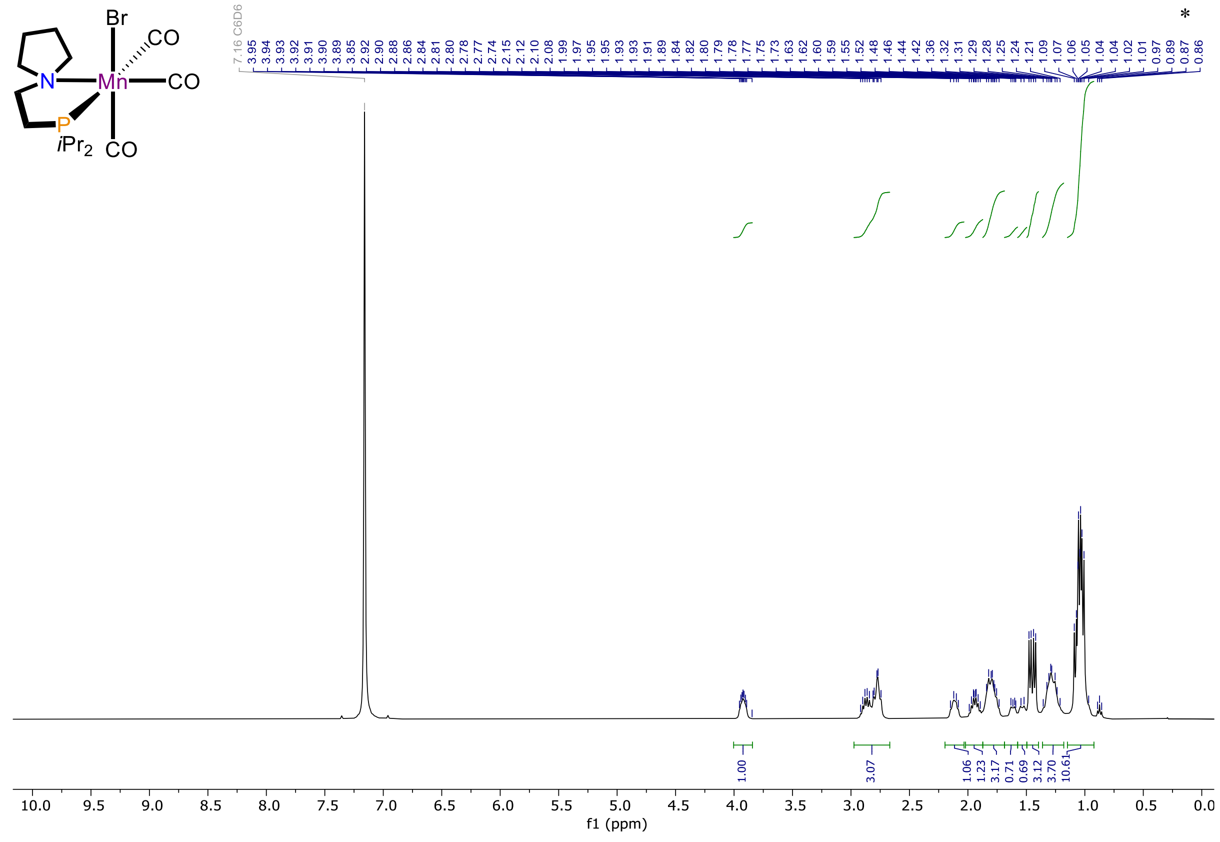

* = *n*-pentane

Figure S47. ^{1}H NMR (400 MHz, C_6D_6) of *fac*-[Mn($P^{iPr}N^{Pyrr}$)$(CO)_3$Br] (**1f**)

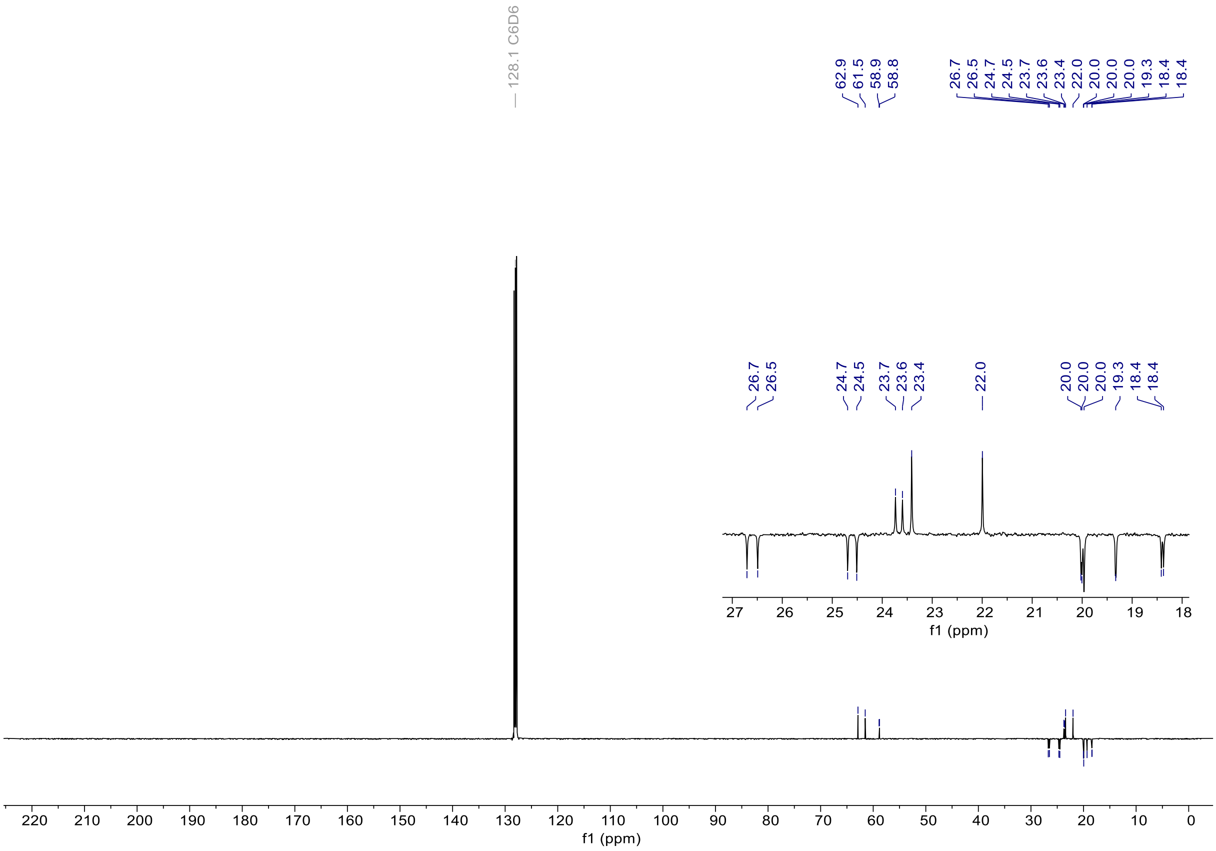

Figure S48. $^{13}C\{^{1}H\}$ NMR (101 MHz, C_6D_6) of *fac*-[Mn($P^{iPr}N^{Pyrr}$)$(CO)_3$Br] (**1f**)

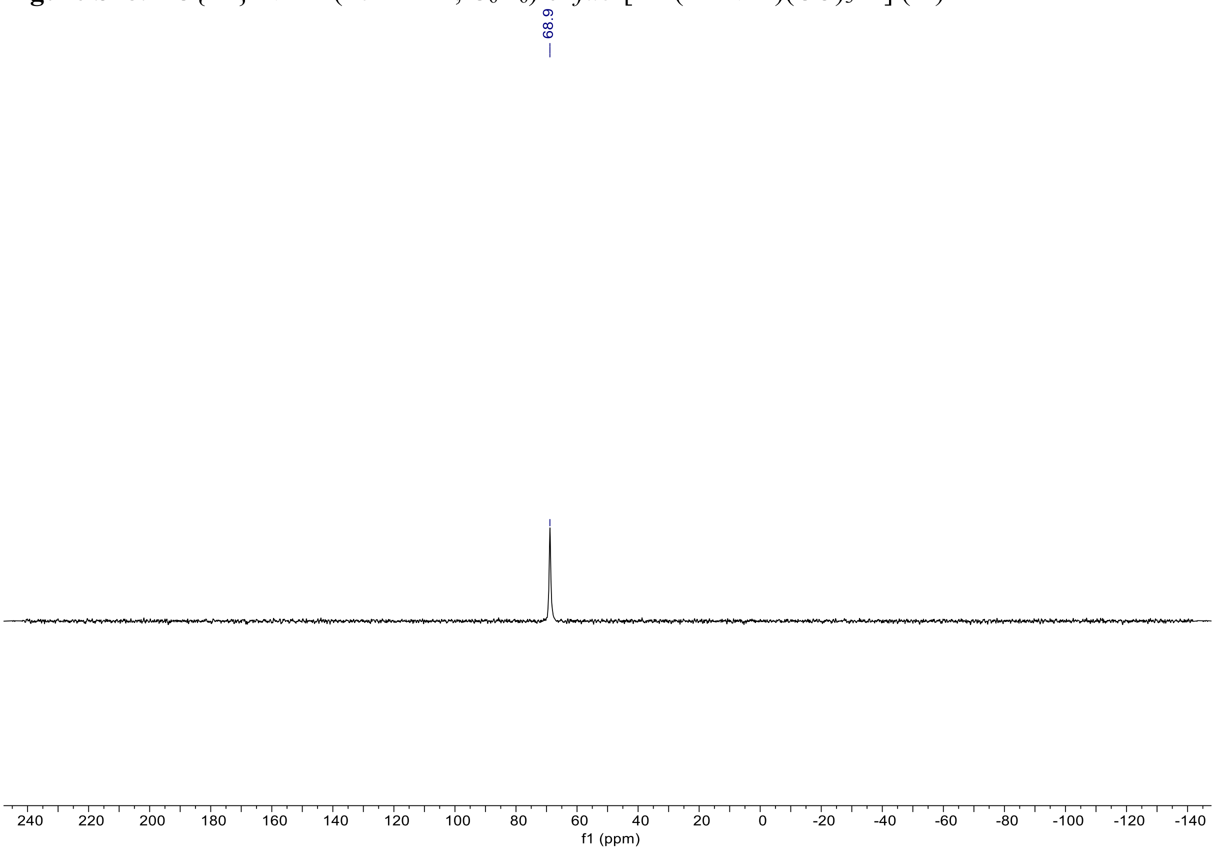

Figure S49. $^{31}P\{^{1}H\}$ NMR (162 MHz, C_6D_6) of *fac*-[Mn($P^{iPr}N^{Pyrr}$)$(CO)_3$Br] (**1f**)

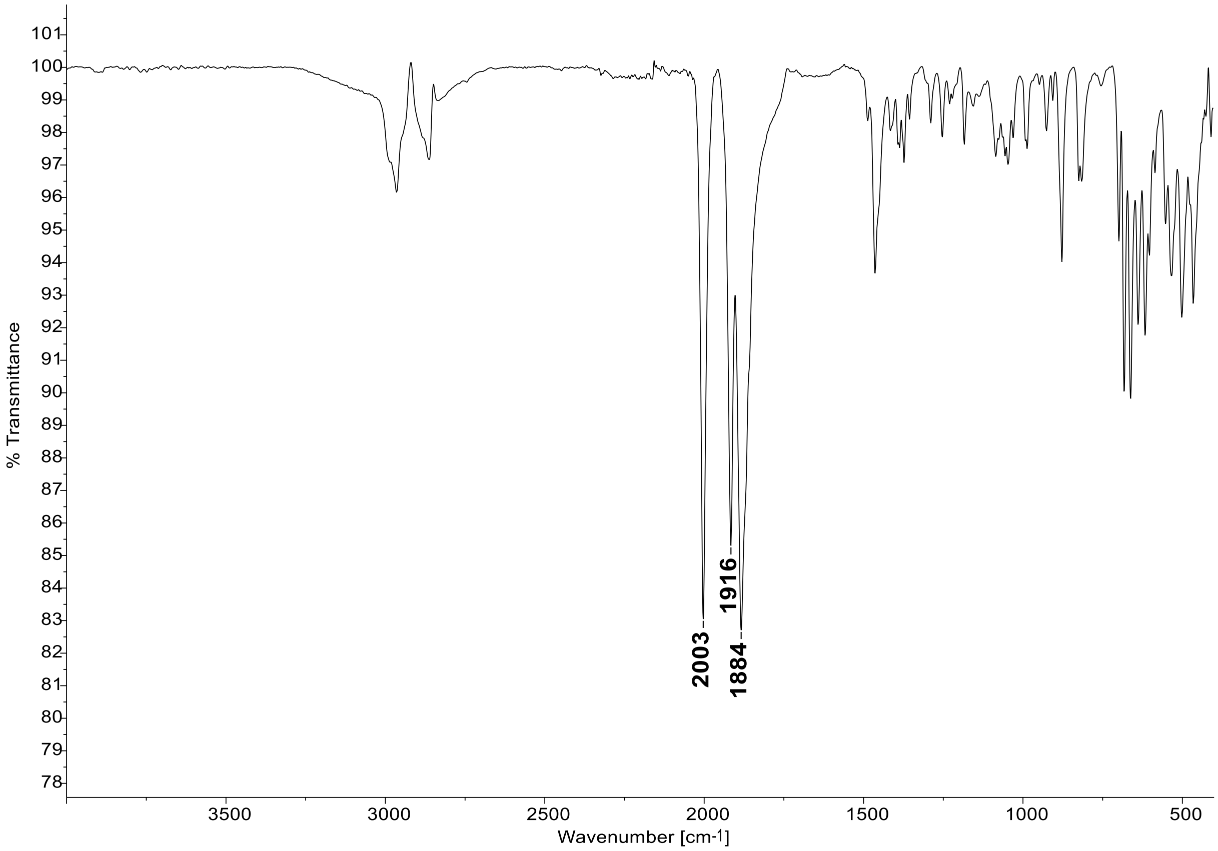

Figure S50. IR (ATR, cm^{-1}) of *fac*-[Mn($P^{iPr}N^{Pyrr}$)$(CO)_3$Br] (**1f**)

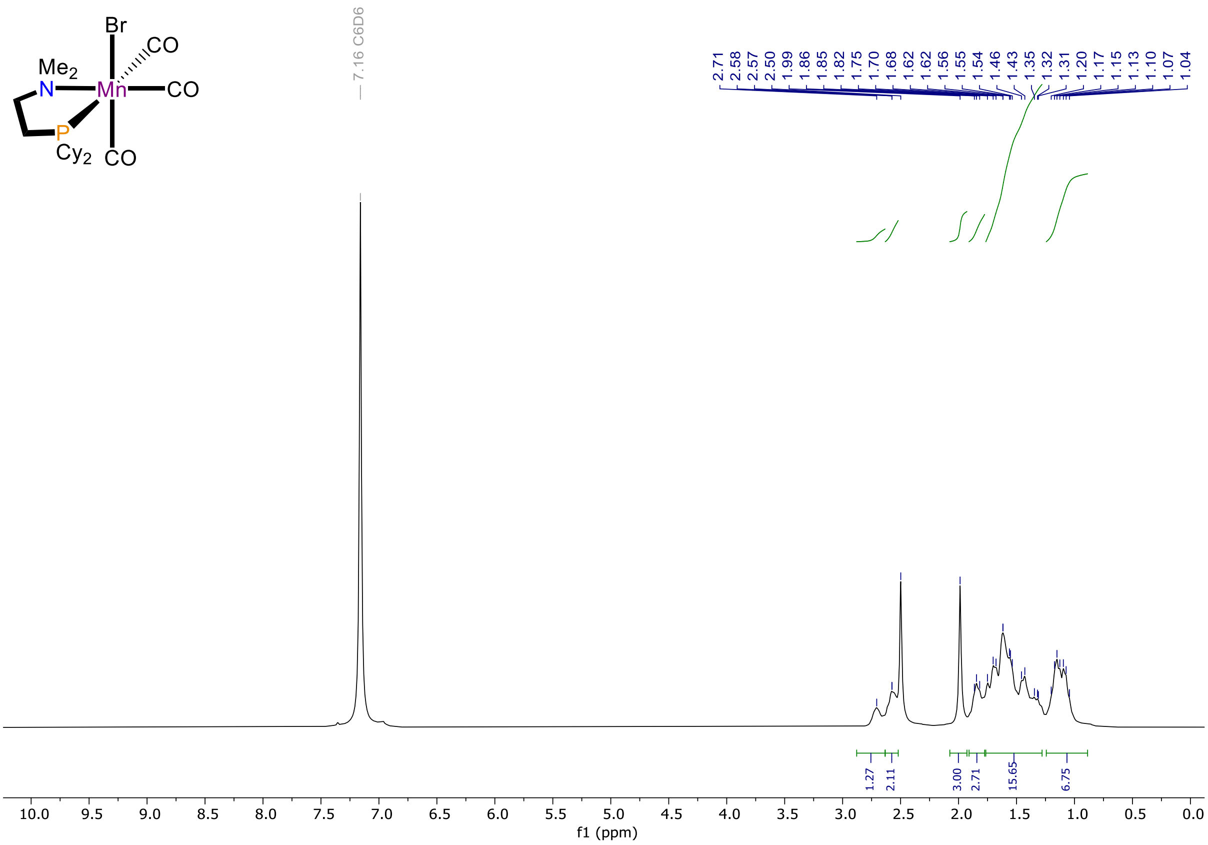

Figure S51. ^{1}H NMR (400 MHz, C_6D_6) of *fac*-[Mn($P^{Cy}N^{Me}$)$(CO)_3$Br] (**1g**)

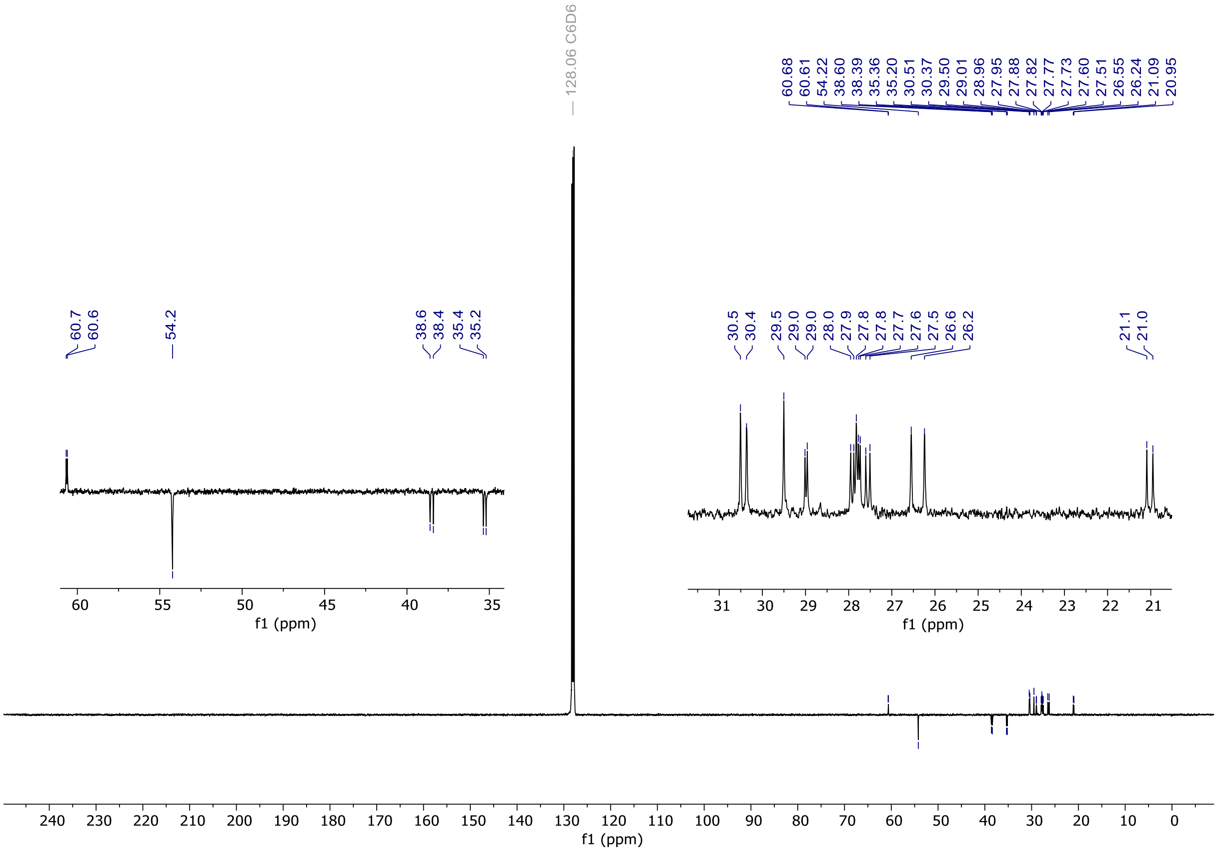

Figure S52. $^{13}C\{^{1}H\}$ NMR (101 MHz, C_6D_6) of *fac*-[Mn($P^{Cy}N^{Me}$)$(CO)_3$Br] (**1g**)

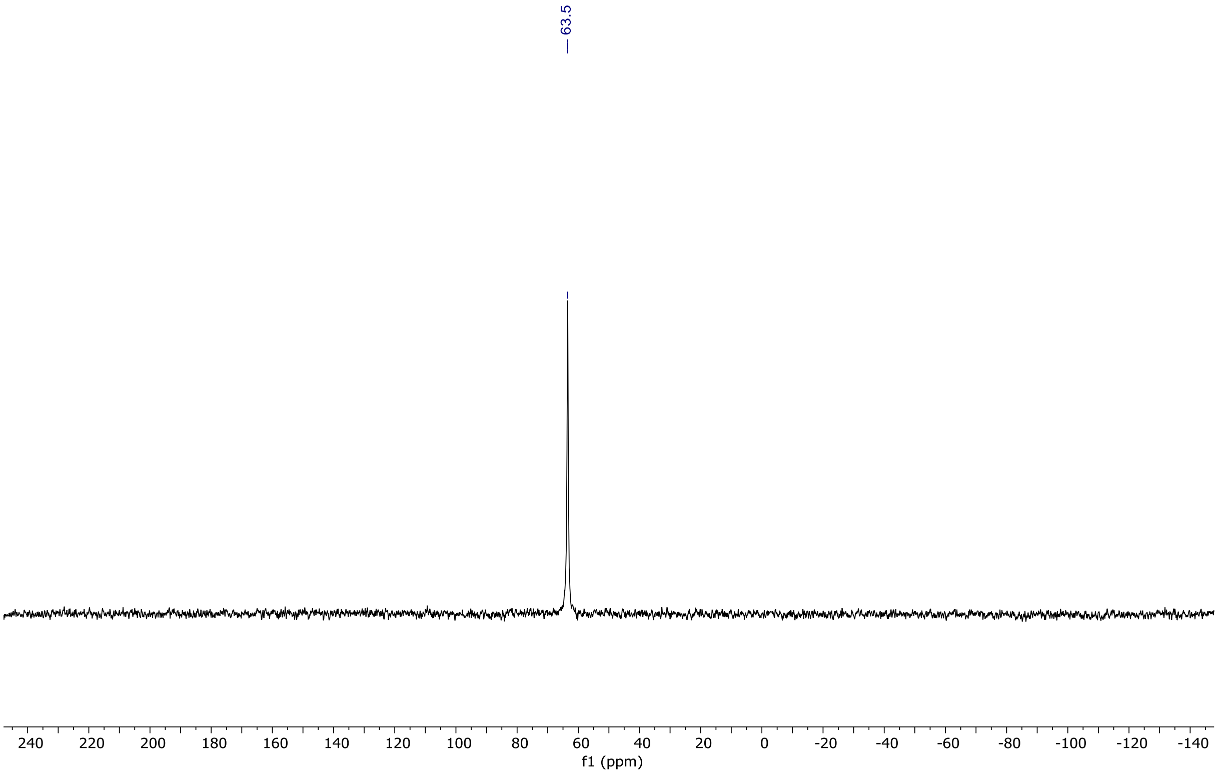

Figure S53. $^{31}P\{^{1}H\}$ NMR (162 MHz, C_6D_6) of *fac*-[Mn($P^{Cy}N^{Me}$)$(CO)_3$Br] (**1g**)

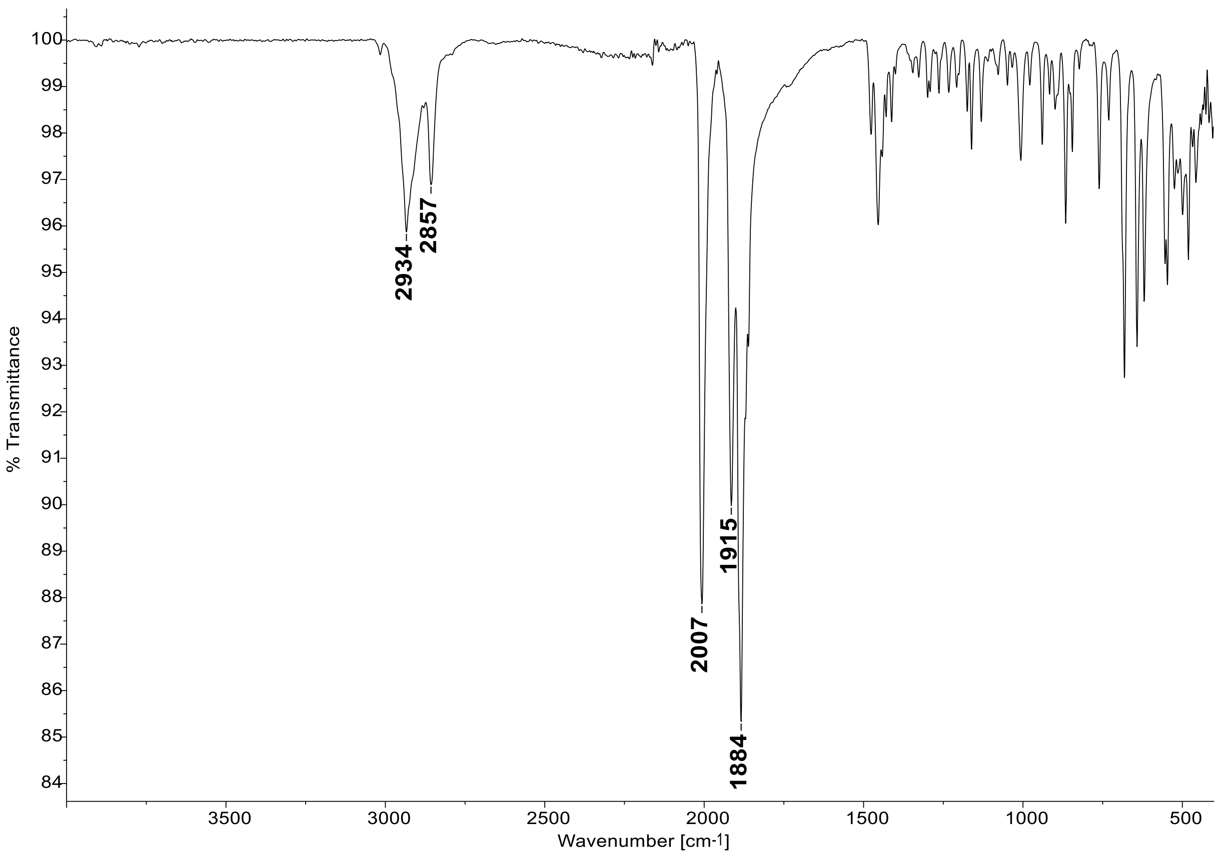

Figure S54. IR (ATR, cm^{-1}) of *fac*-[Mn($P^{Cy}N^{Me}$)$(CO)_3$Br] (**1g**)

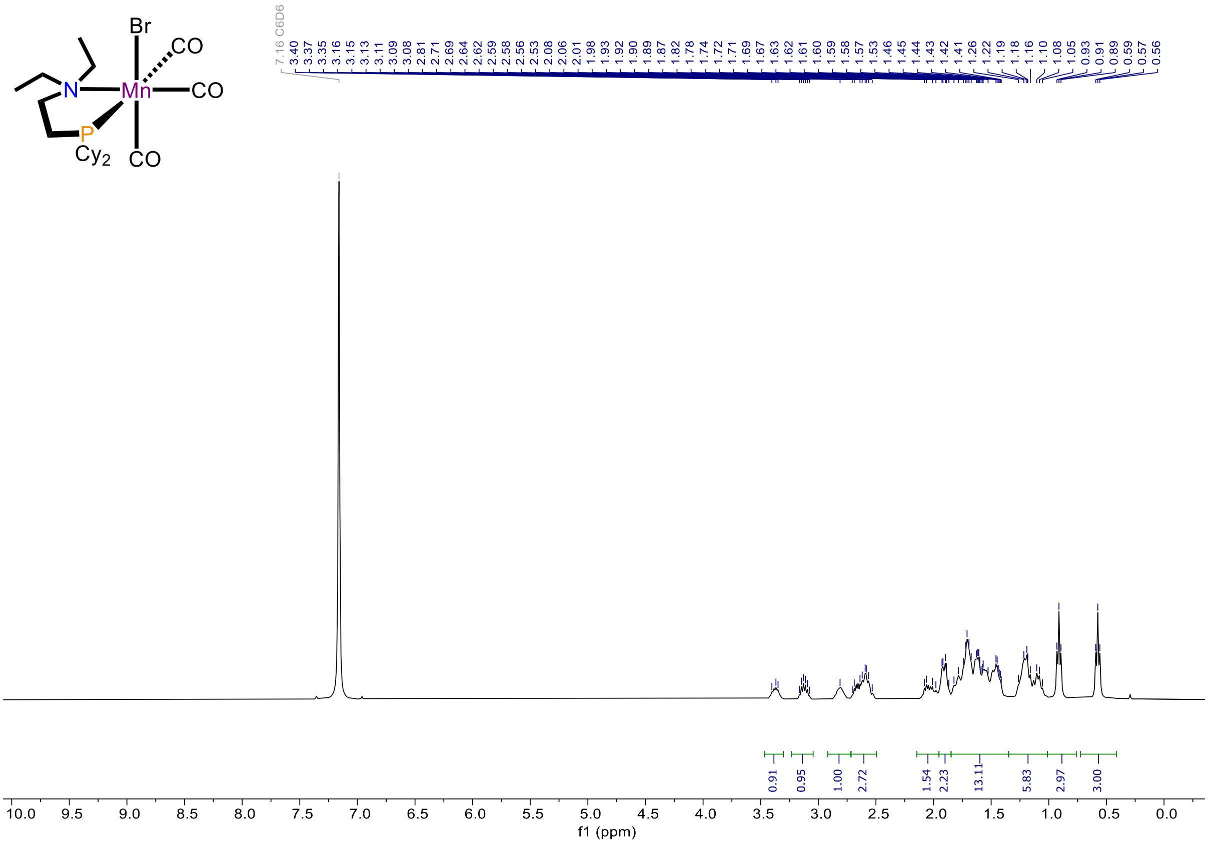

Figure S55. ^{1}H NMR (400 MHz, C_6D_6) of *fac*-[Mn($P^{Cy}N^{Et}$)$(CO)_3$Br] (**1h**)

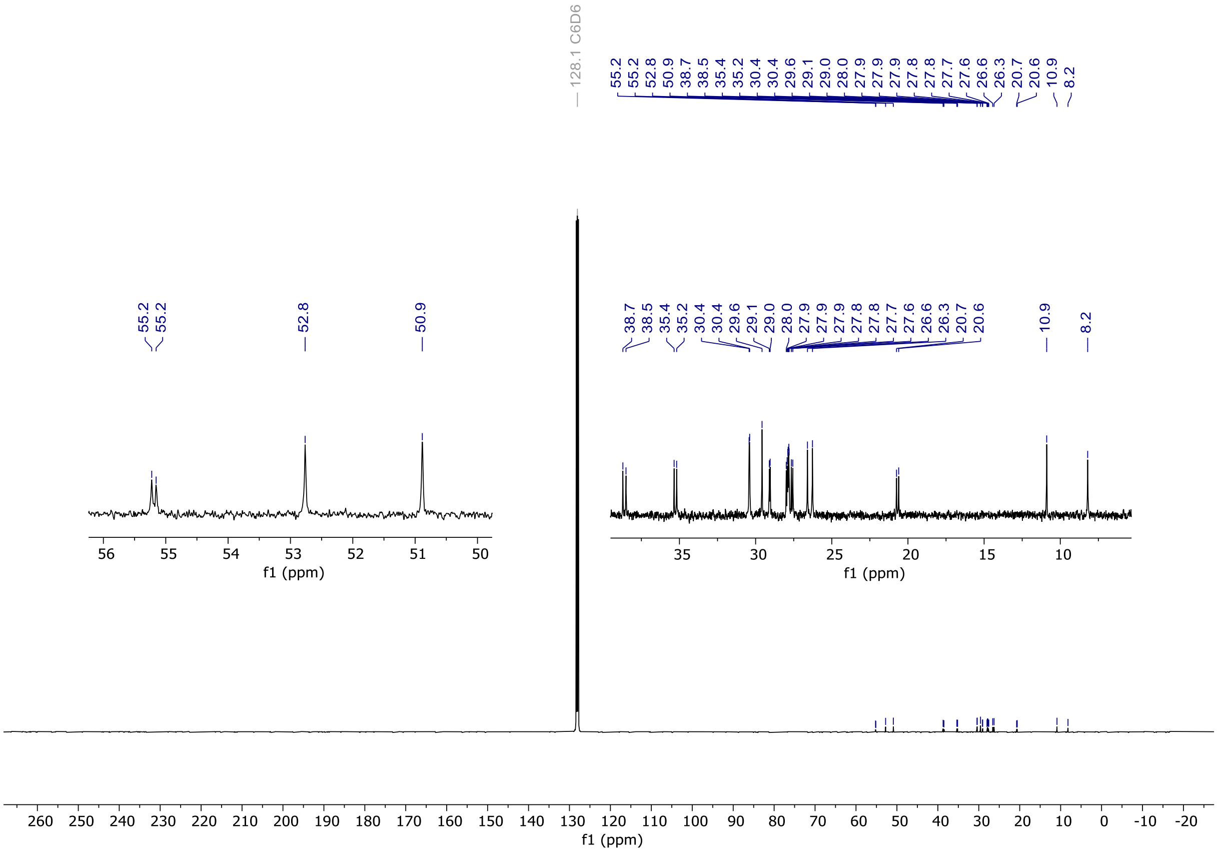

Figure S56. $^{13}C\{^{1}H\}$ NMR (101 MHz, C_6D_6) of *fac*-[Mn($P^{Cy}N^{Et}$)$(CO)_3$Br] (**1h**)

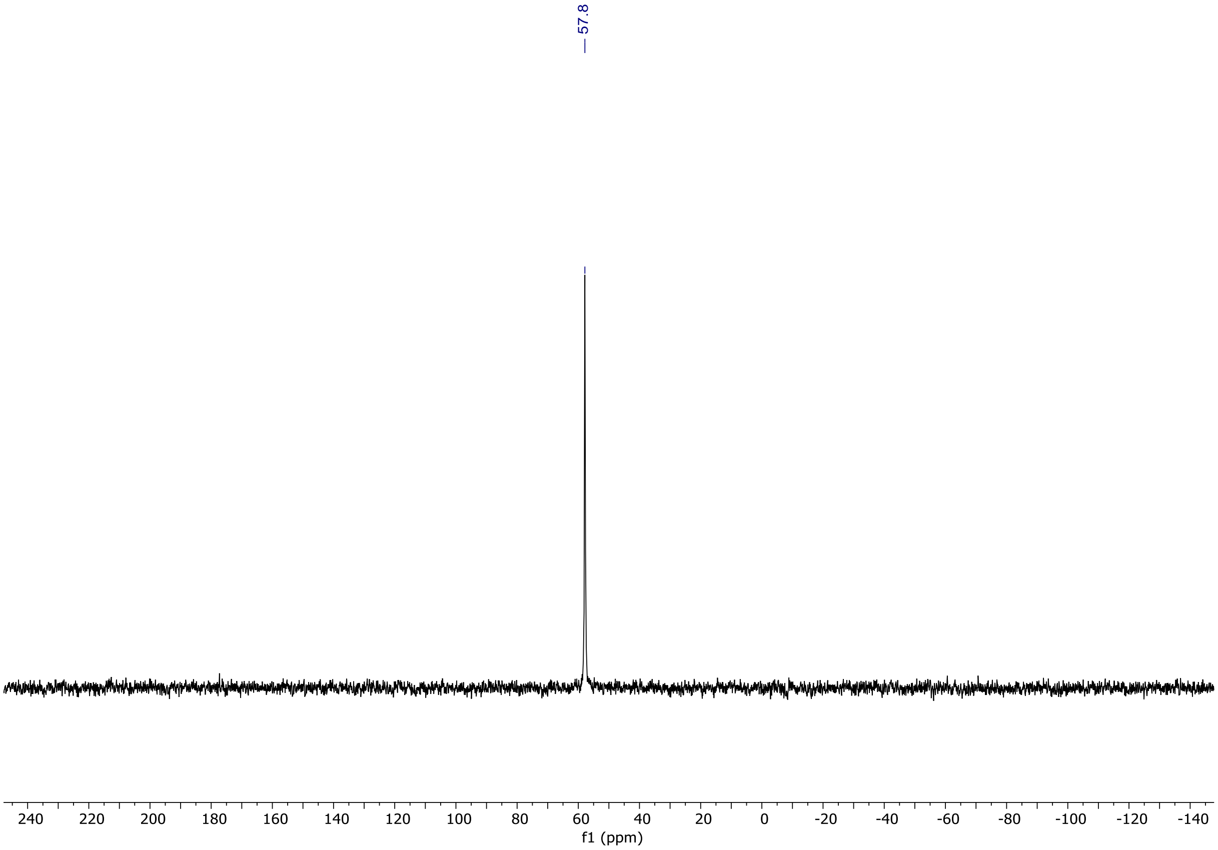

Figure S57. $^{31}P\{^{1}H\}$ NMR (162 MHz, C_6D_6) of *fac*-[Mn($P^{Cy}N^{Et}$)$(CO)_3$Br] (**1h**)

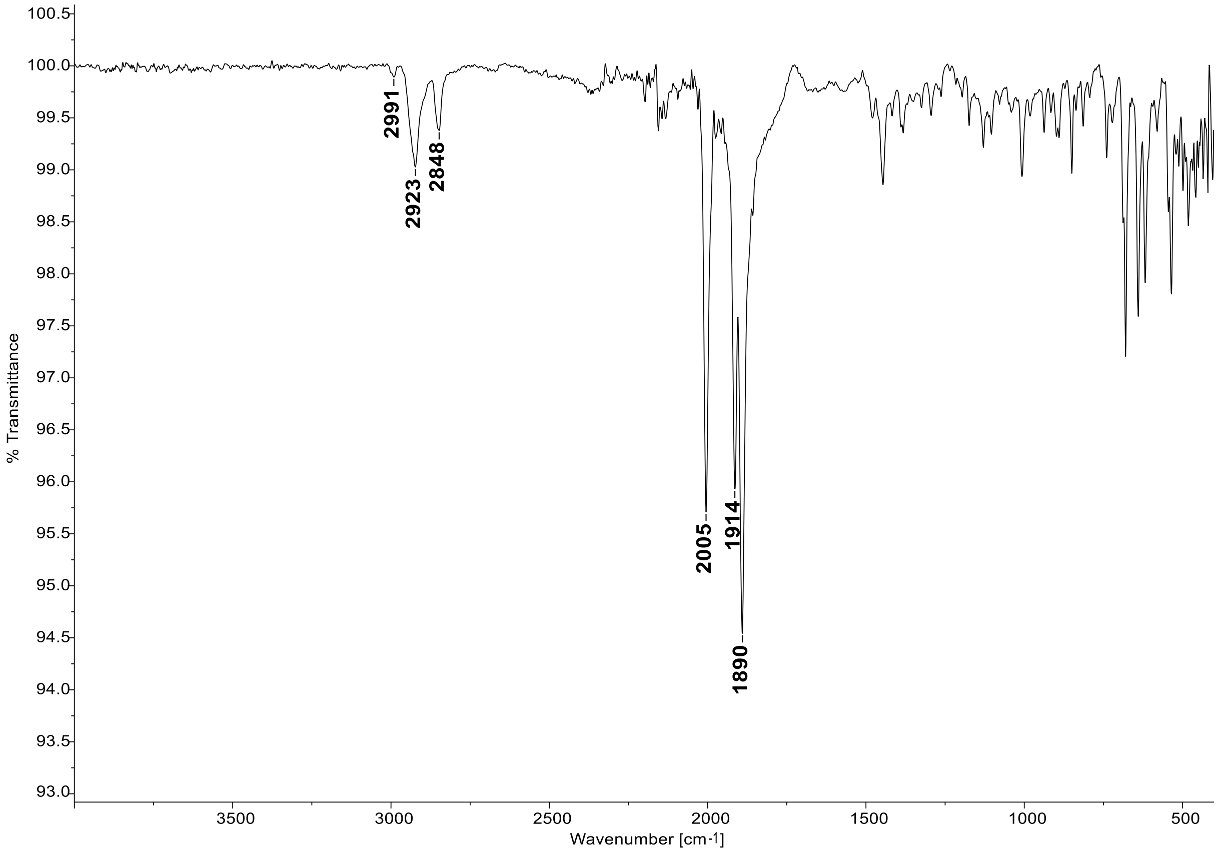

Figure S58. IR (ATR, cm^{-1}) of *fac*-[Mn($P^{Cy}N^{Et}$)$(CO)_3$Br] (**1h**)

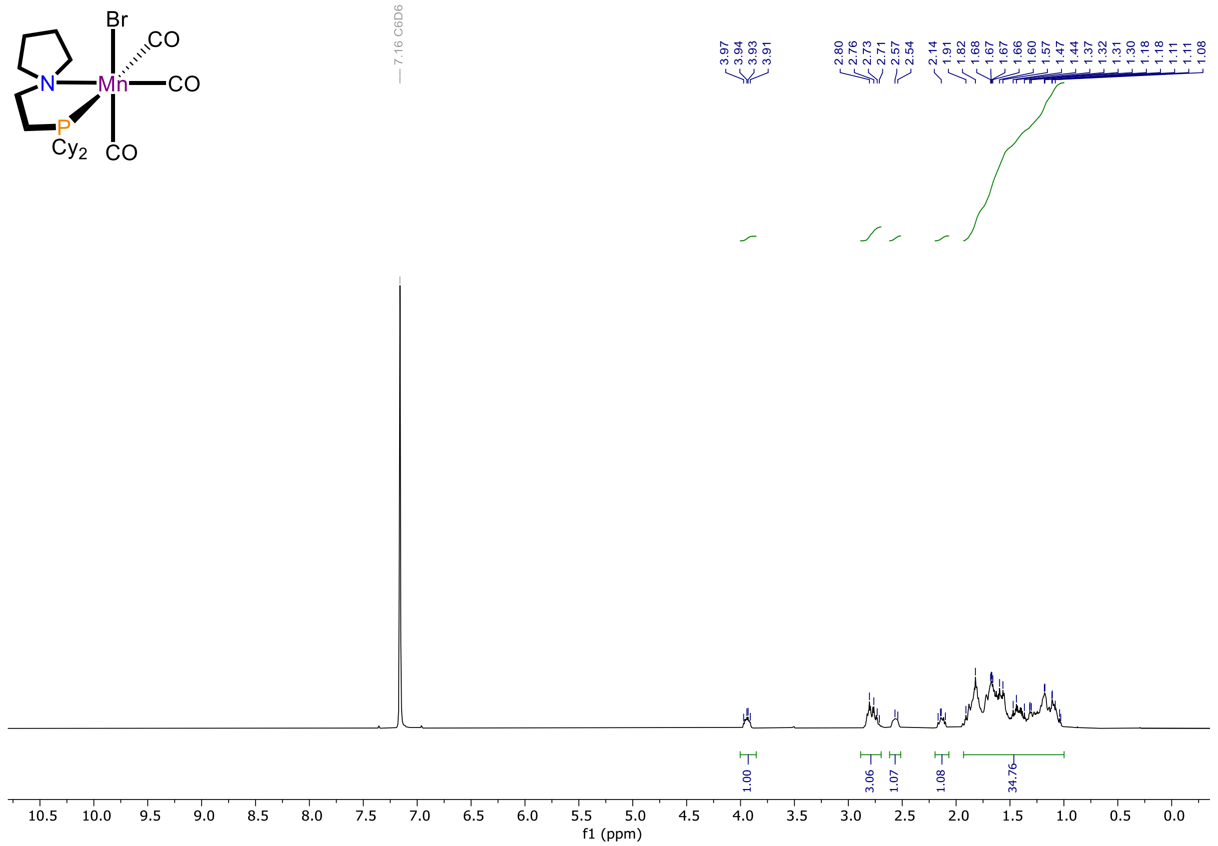

Figure S59. ^{1}H NMR (400 MHz, C_6D_6) of *fac*-[Mn($P^{Cy}N^{Pyr}$)$(CO)_3$Br] (**1i**)

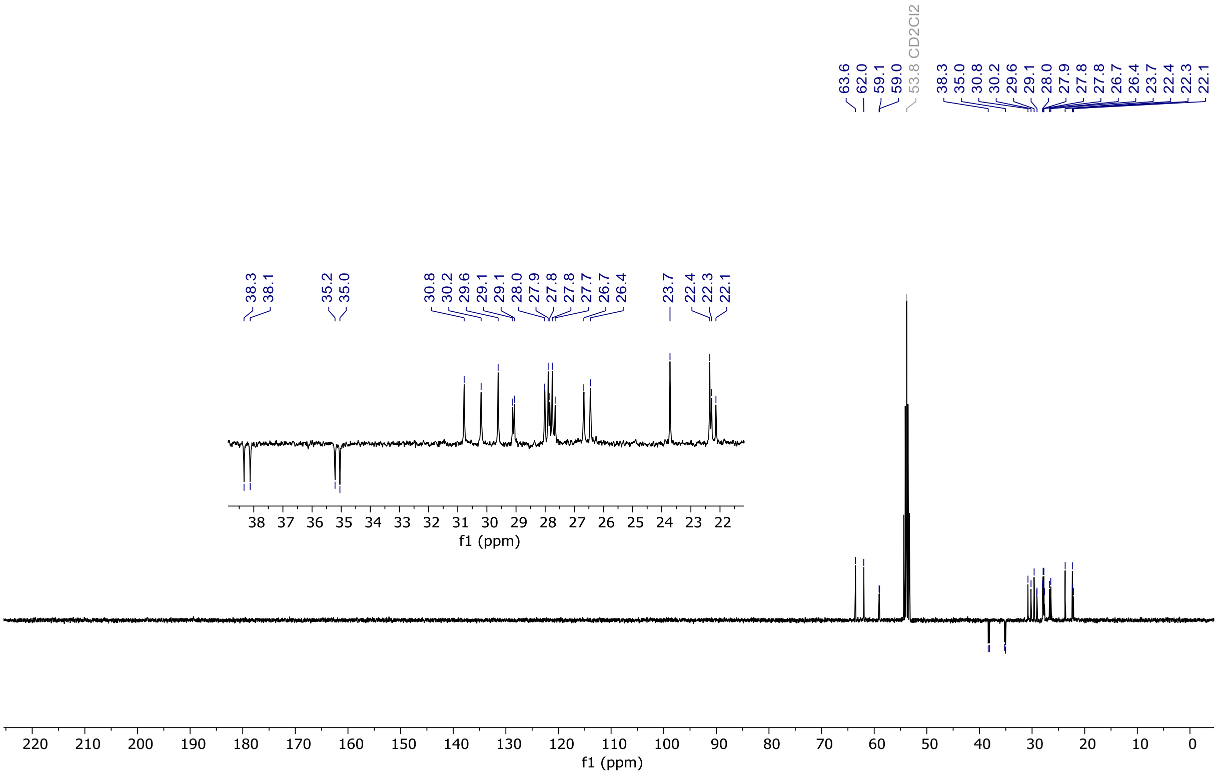

Figure S60. $^{13}C\{^1H\}$ NMR (101 MHz, CD_2Cl_2) of *fac*-[Mn($P^{Cy}N^{Pyr}$)(CO)$_3$Br] (**1i**)

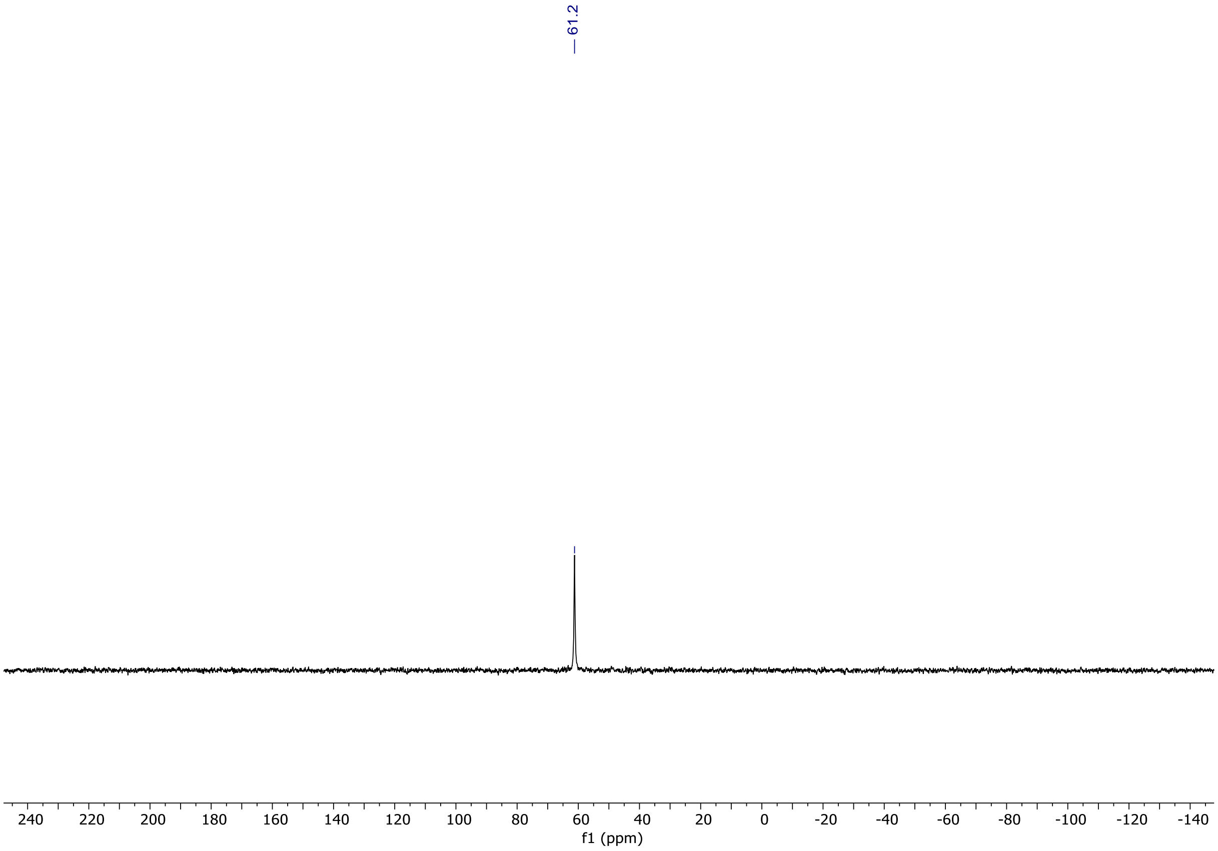

Figure S61. $^{31}P\{^1H\}$ NMR (162 MHz, C_6D_6) of *fac*-[Mn($P^{Cy}N^{Pyr}$)(CO)$_3$Br] (**1i**)

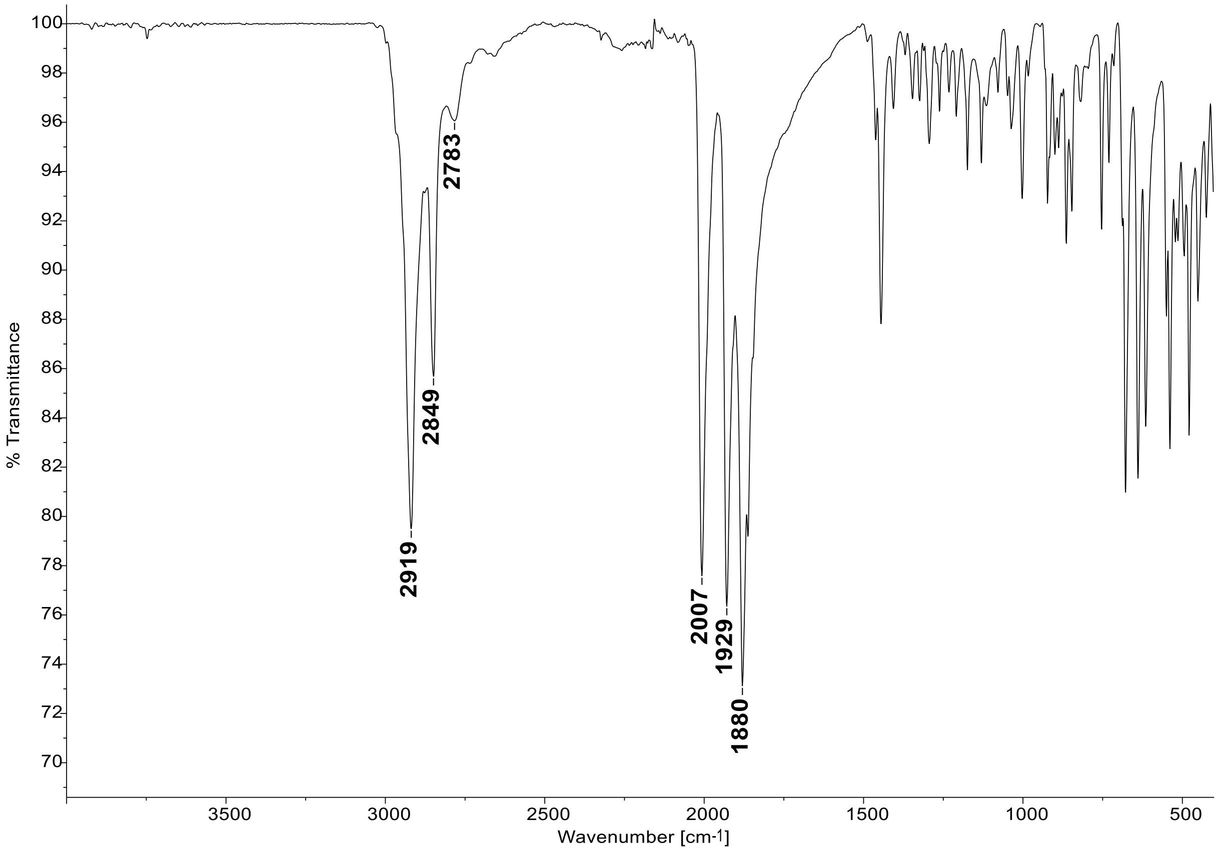

Figure S62. IR (ATR, cm^{-1}) of *fac*-[Mn(P^{Cy}N^{Pyr})(CO)$_3$Br] (**1i**)

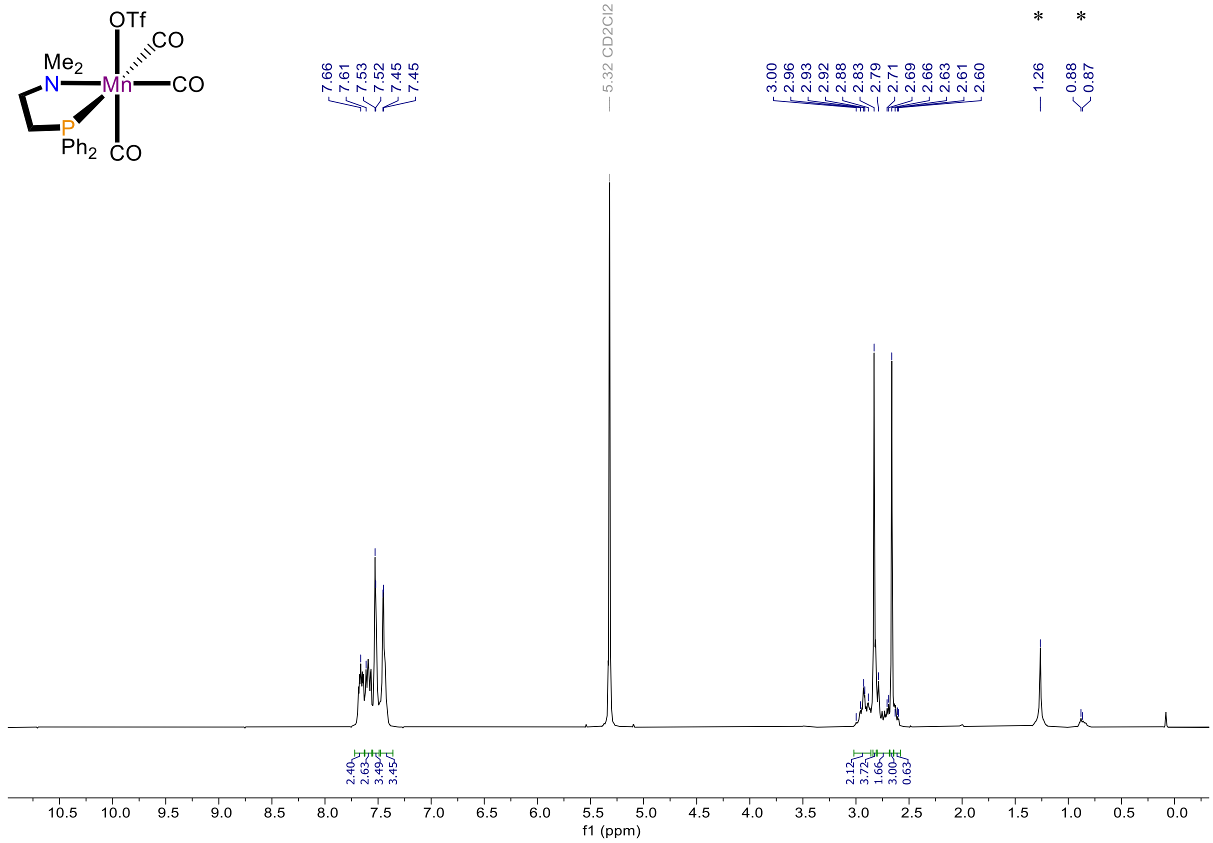

* = grease

Figure S63. ^{1}H NMR (400 MHz, CD_2Cl_2) of *fac*-[Mn(P^{Ph}N^{Me})(CO)$_3$OTf] (**2a**)

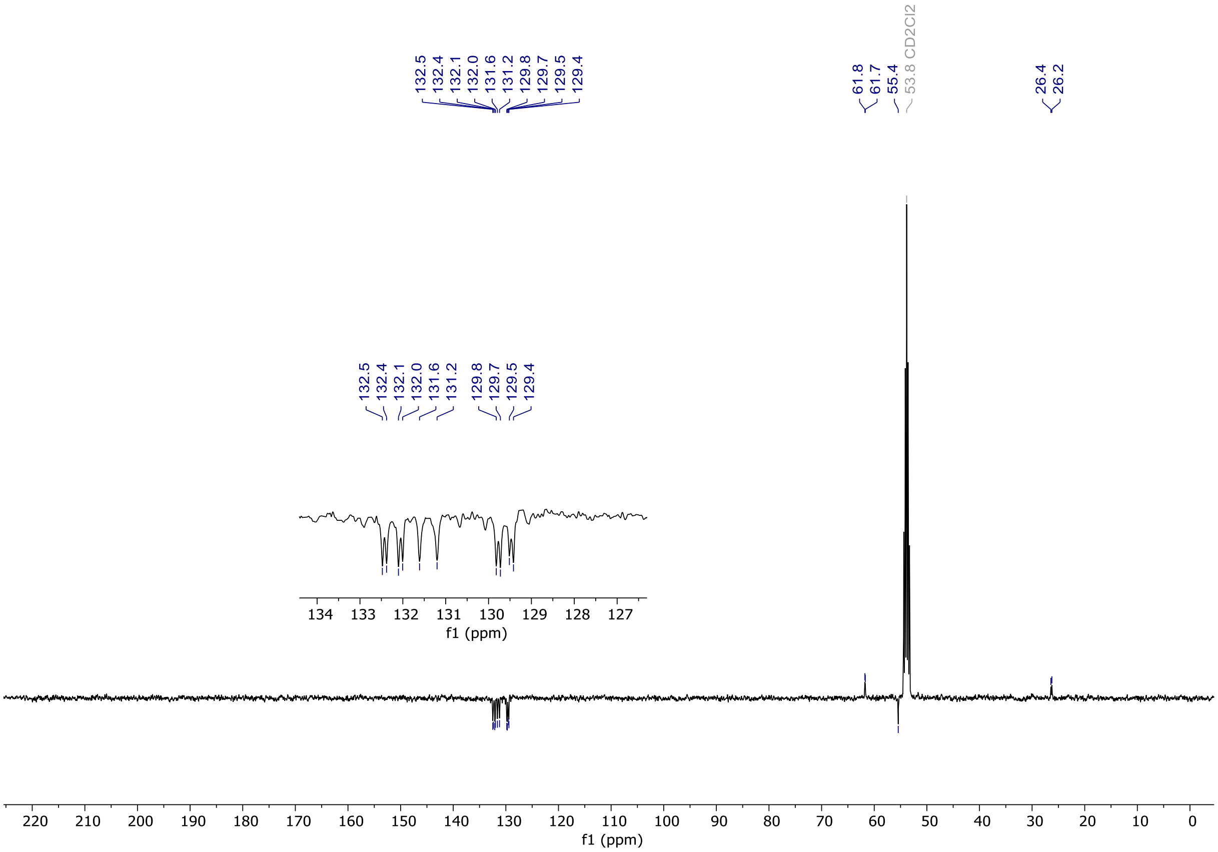

Figure S64. $^{13}C\{^1H\}$ NMR (101 MHz, CD_2Cl_2) of *fac*-[Mn($P^{Ph}N^{Me}$)$(CO)_3$OTf] (**2a**)

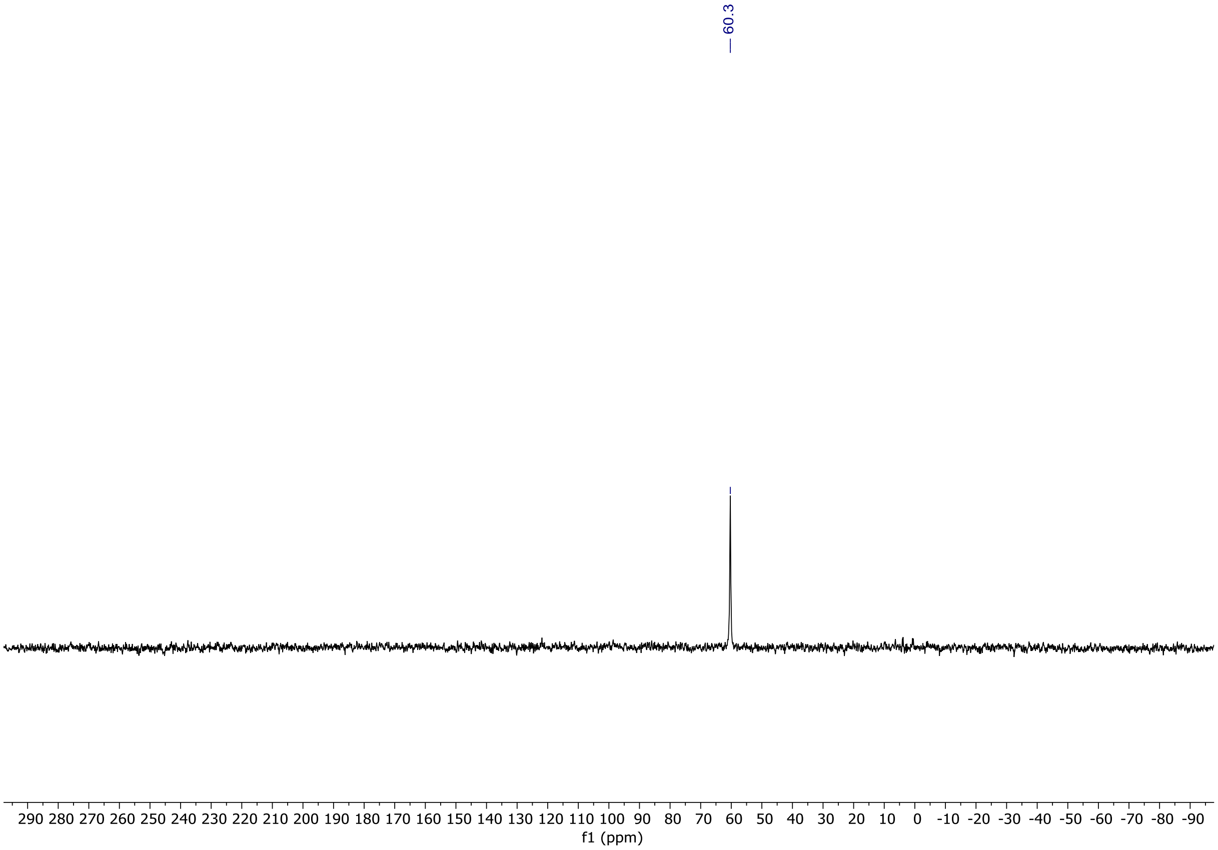

Figure S65. $^{31}P\{^1H\}$ NMR (162 MHz, CD_2Cl_2) of *fac*-[Mn($P^{Ph}N^{Me}$)$(CO)_3$OTf] (**2a**)

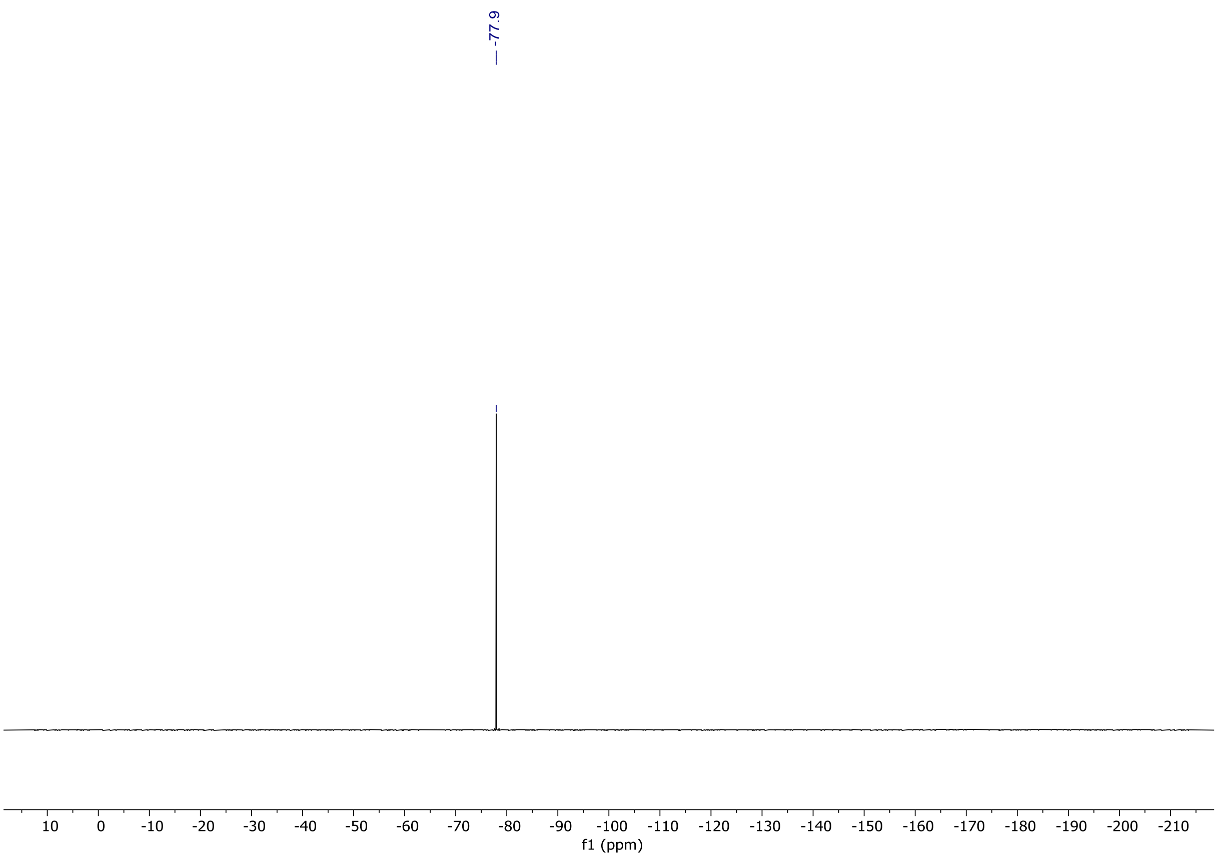

Figure S66. $^{19}F\{^{1}H\}$ NMR (376 MHz, CD_2Cl_2) of *fac*-[Mn($P^{Ph}N^{Me}$)$(CO)_3$OTf] (**2a**)

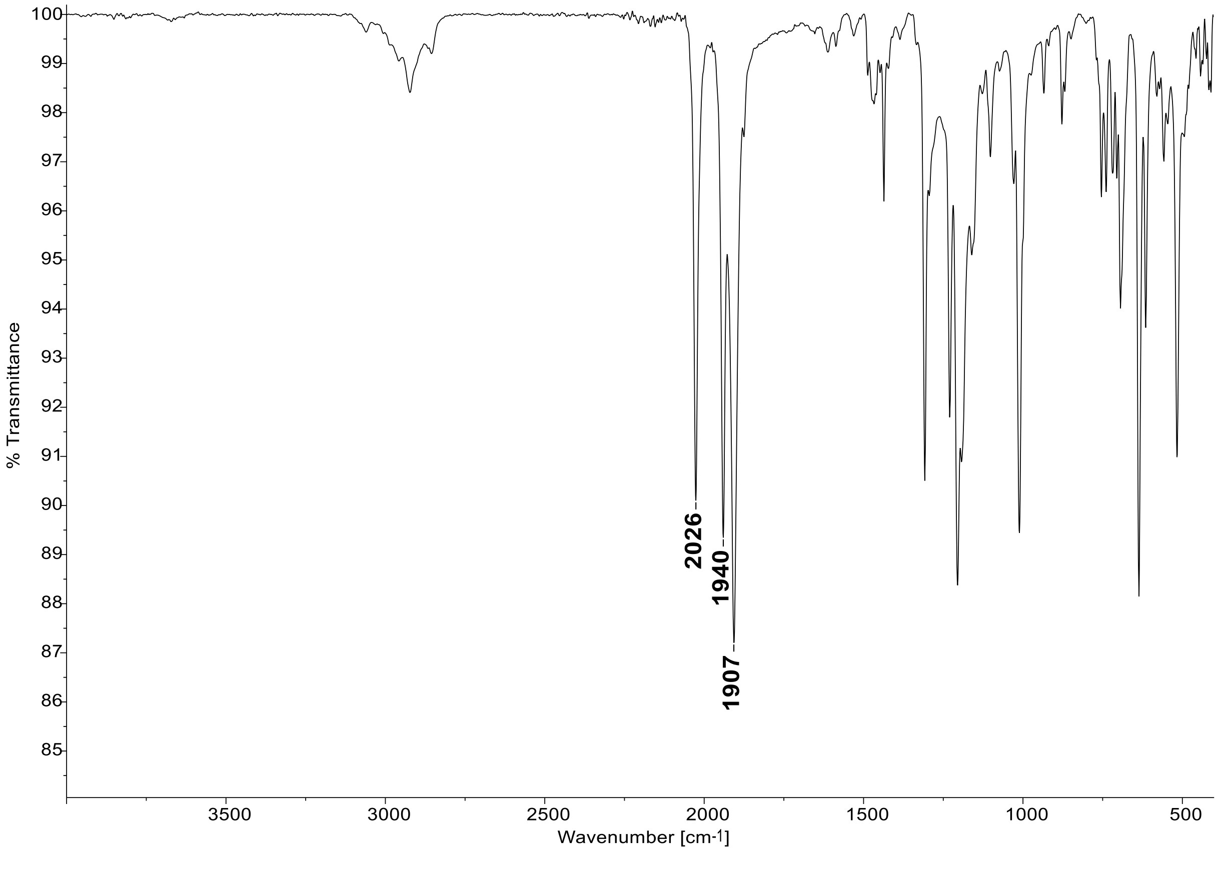

Figure S67. IR (ATR, cm^{-1}) of *fac*-[Mn($P^{Ph}N^{Me}$)$(CO)_3$OTf] (**2a**)

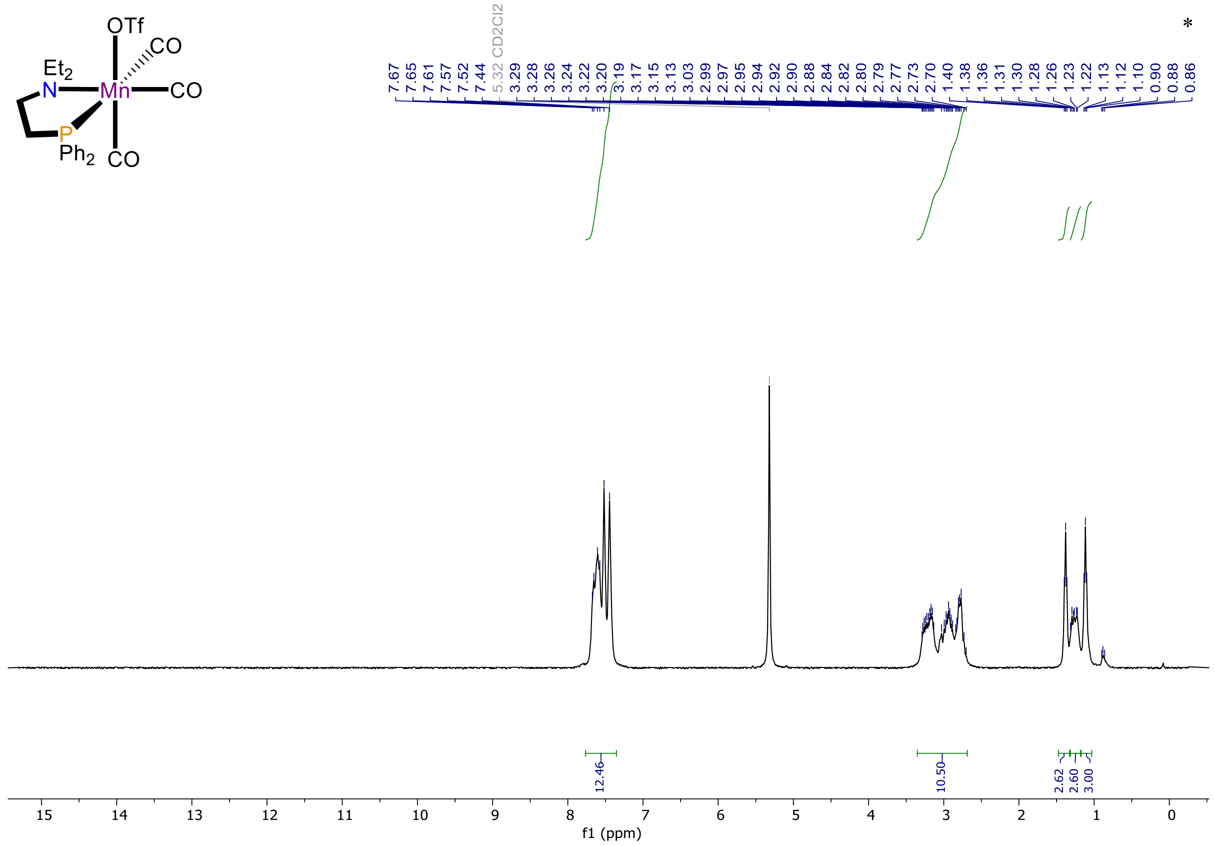

* = grease

Figure S68. ^{1}H NMR (400 MHz, CD_2Cl_2) of *fac*-[Mn($P^{Ph}N^{Et}$)(CO)$_3$OTf] (**2b**)

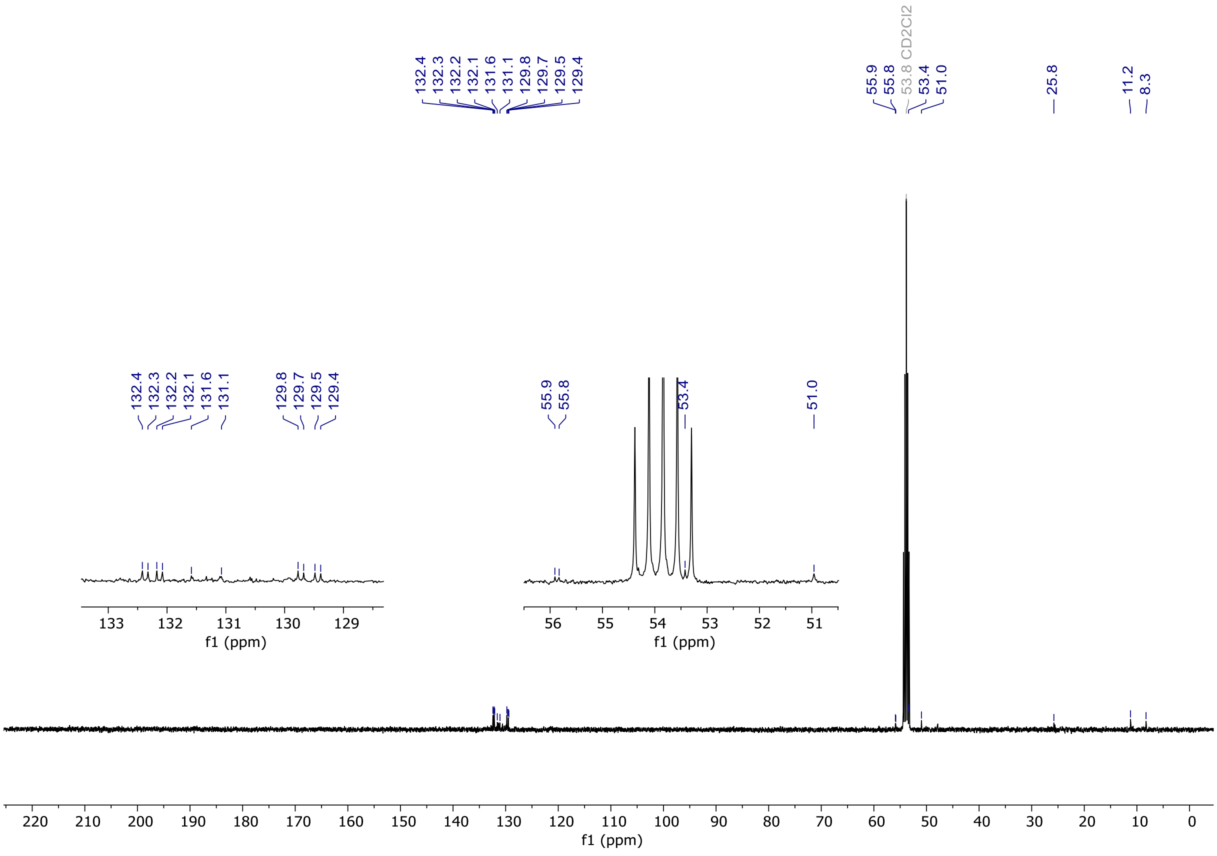

Figure S69. ^{13}C{^{1}H} NMR (101 MHz, CD_2Cl_2) of *fac*-[Mn($P^{Ph}N^{Et}$)(CO)$_3$OTf] (**2b**)

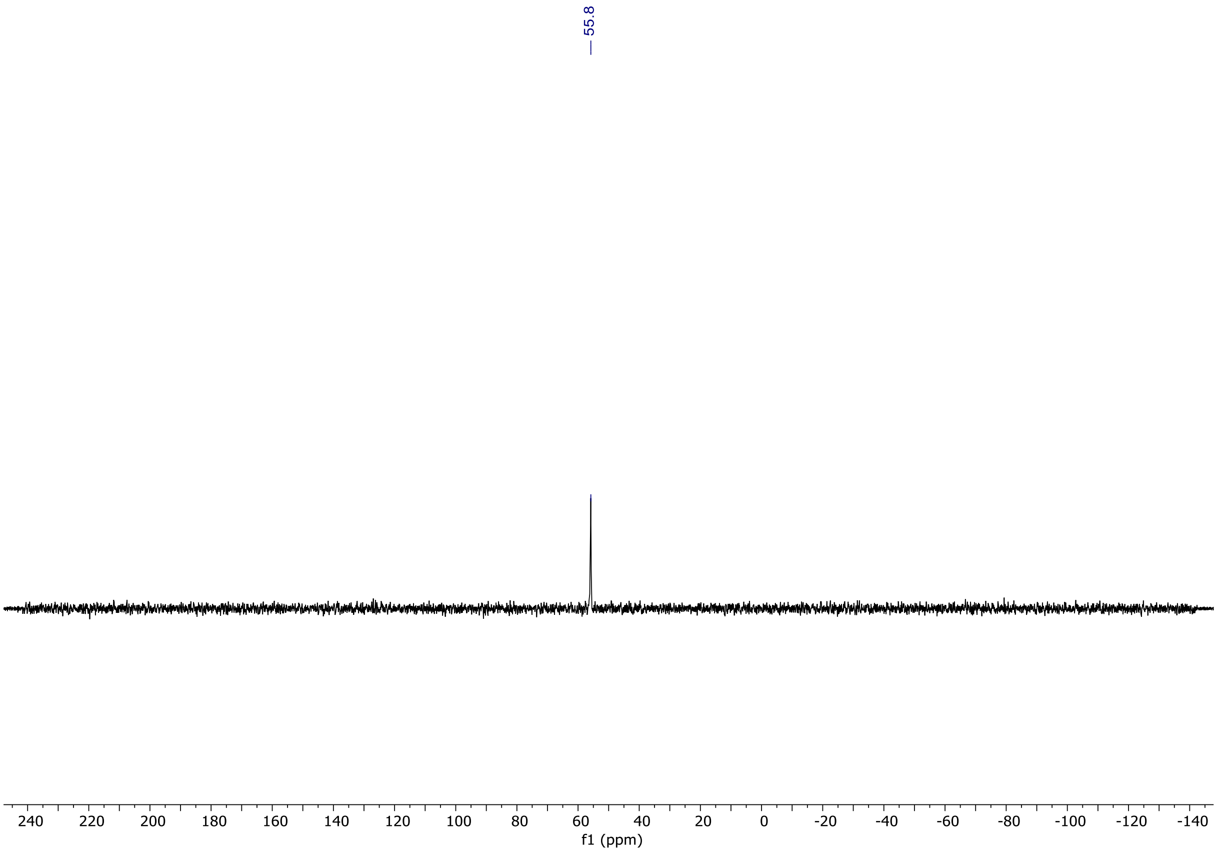

Figure S70. $^{31}P\{^{1}H\}$ NMR (162 MHz, CD_2Cl_2) of *fac*-[Mn($P^{Ph}N^{Et}$)$(CO)_3$OTf] (**2b**.

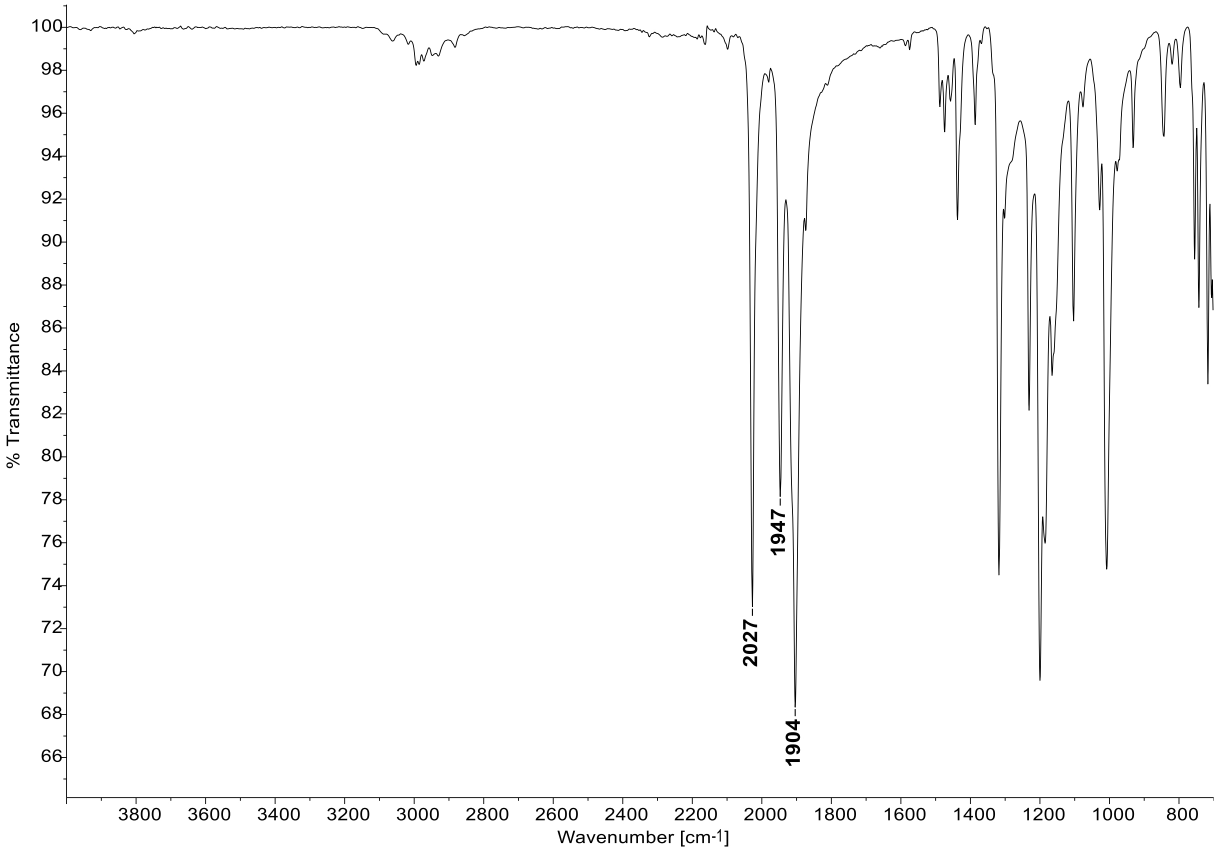

Figure S71. IR (ATR, cm^{-1}) of *fac*-[Mn($P^{Ph}N^{Et}$)$(CO)_3$OTf] (**2b**)

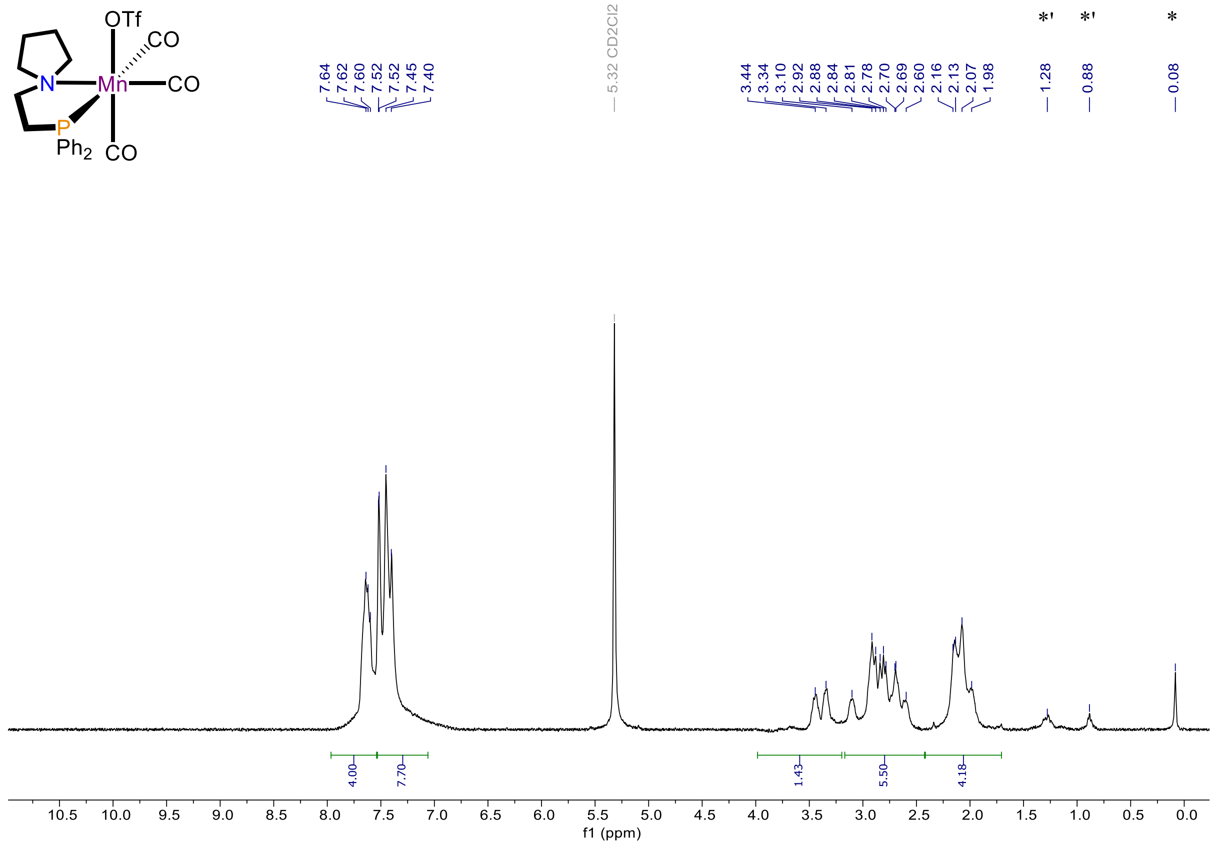

* = grease, *' = *n*-pentane

Figure S72. ^{1}H NMR (400 MHz, CD_2Cl_2) of *fac*-[Mn($P^{Ph}N^{Pyrr}$)$(CO)_3$OTf] (**2c**)

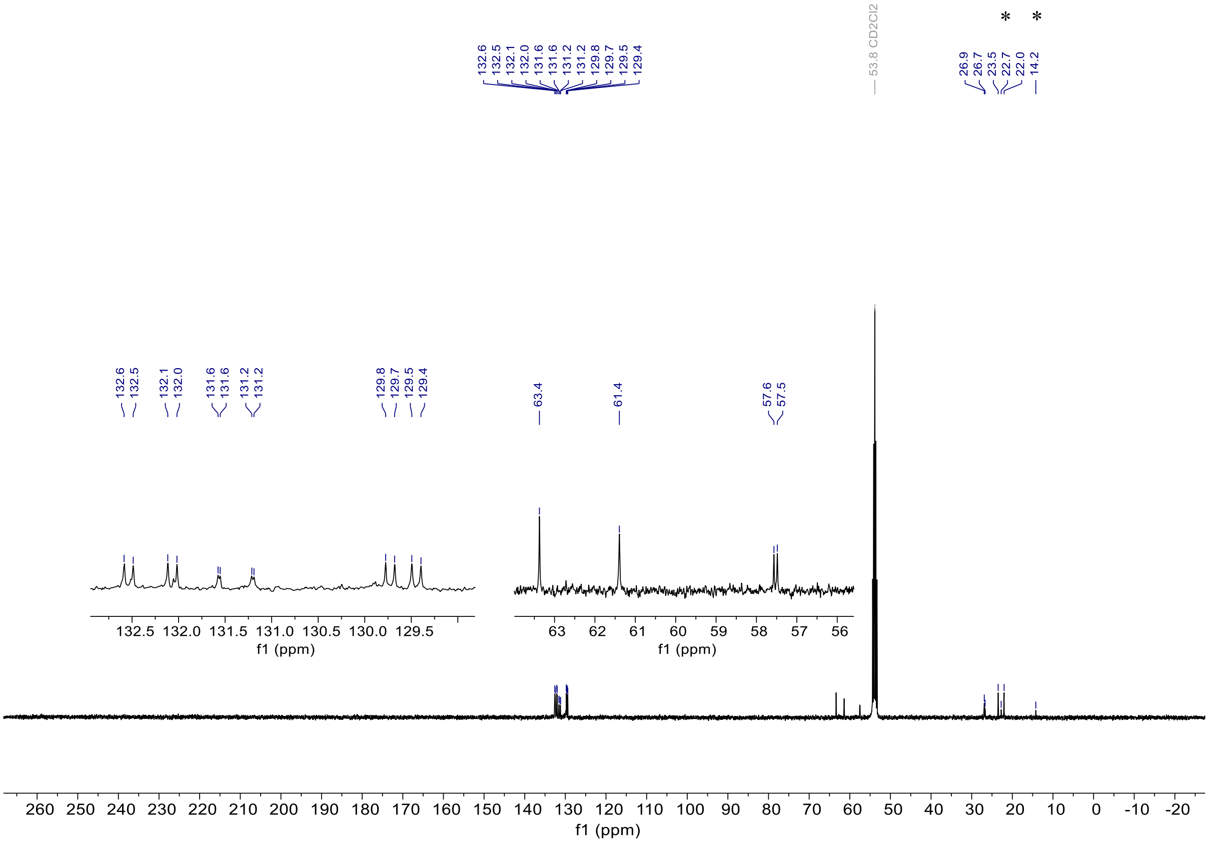

* = *n*-pentane

Figure S73. $^{13}C\{^1H\}$ NMR (101 MHz, CD_2Cl_2) of *fac*-[Mn($P^{Ph}N^{Pyrr}$)(CO)$_3$OTf] (**2c**)

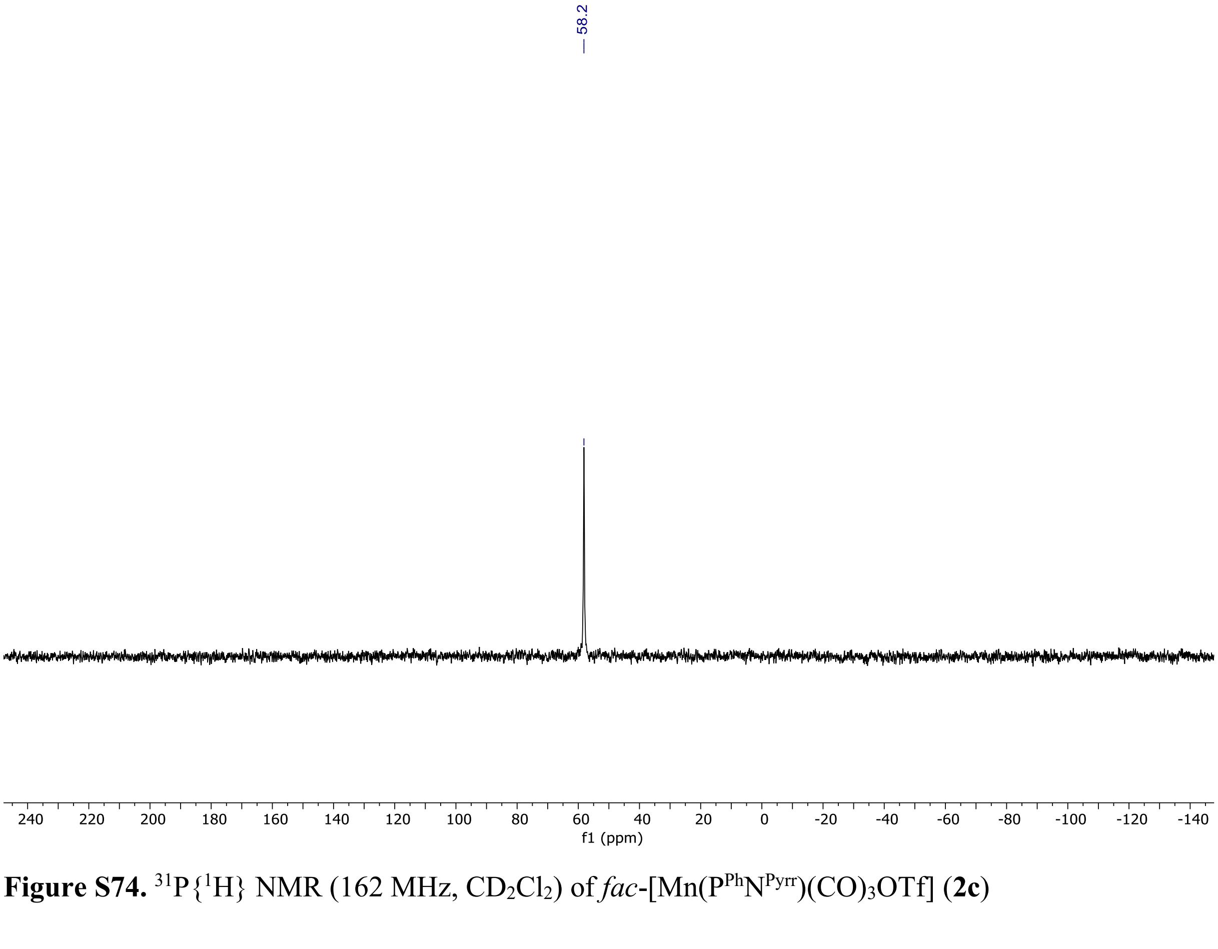

Figure S74. $^{31}P\{^1H\}$ NMR (162 MHz, CD_2Cl_2) of *fac*-[Mn($P^{Ph}N^{Pyrr}$)(CO)$_3$OTf] (**2c**)

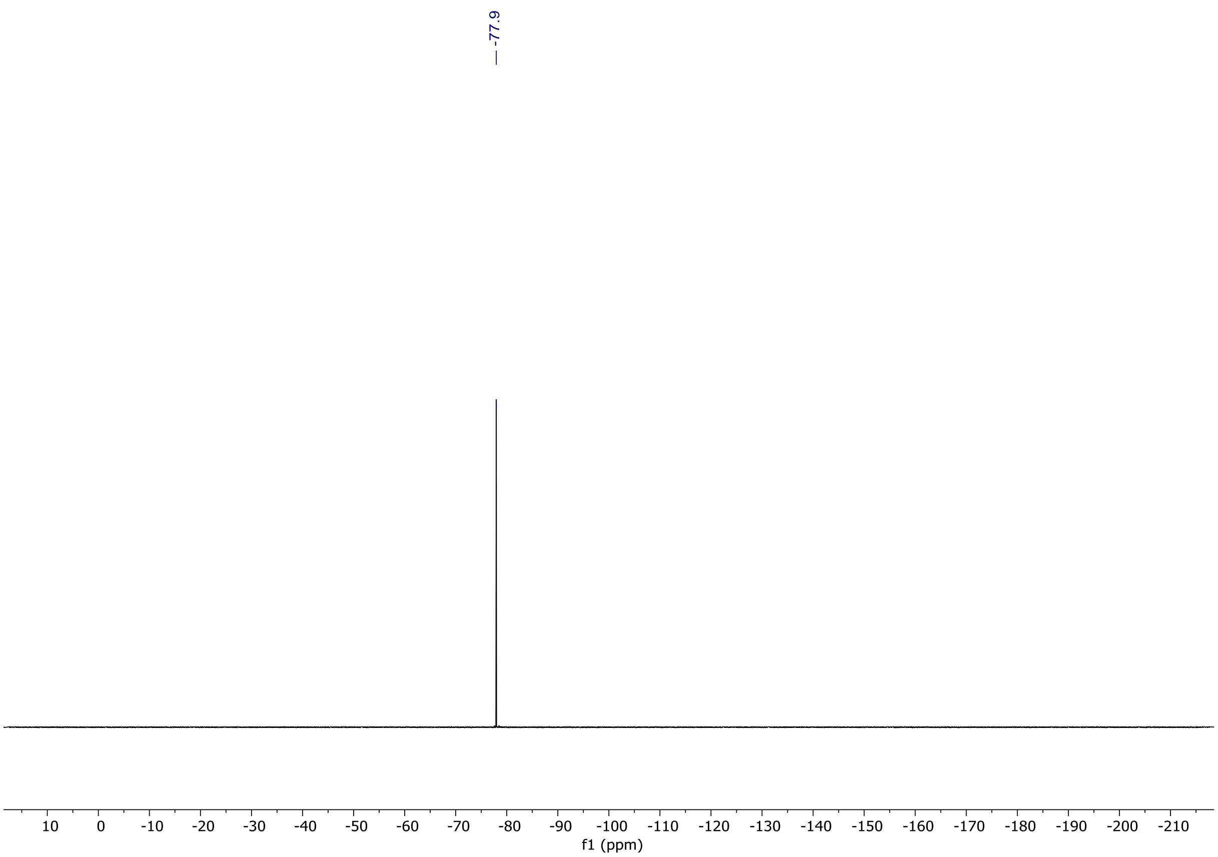

Figure S75. $^{19}F\{^1H\}$ NMR (376 MHz, CD_2Cl_2) of *fac*-[Mn($P^{Ph}N^{Pyrr}$)$(CO)_3$OTf] (**2c**)

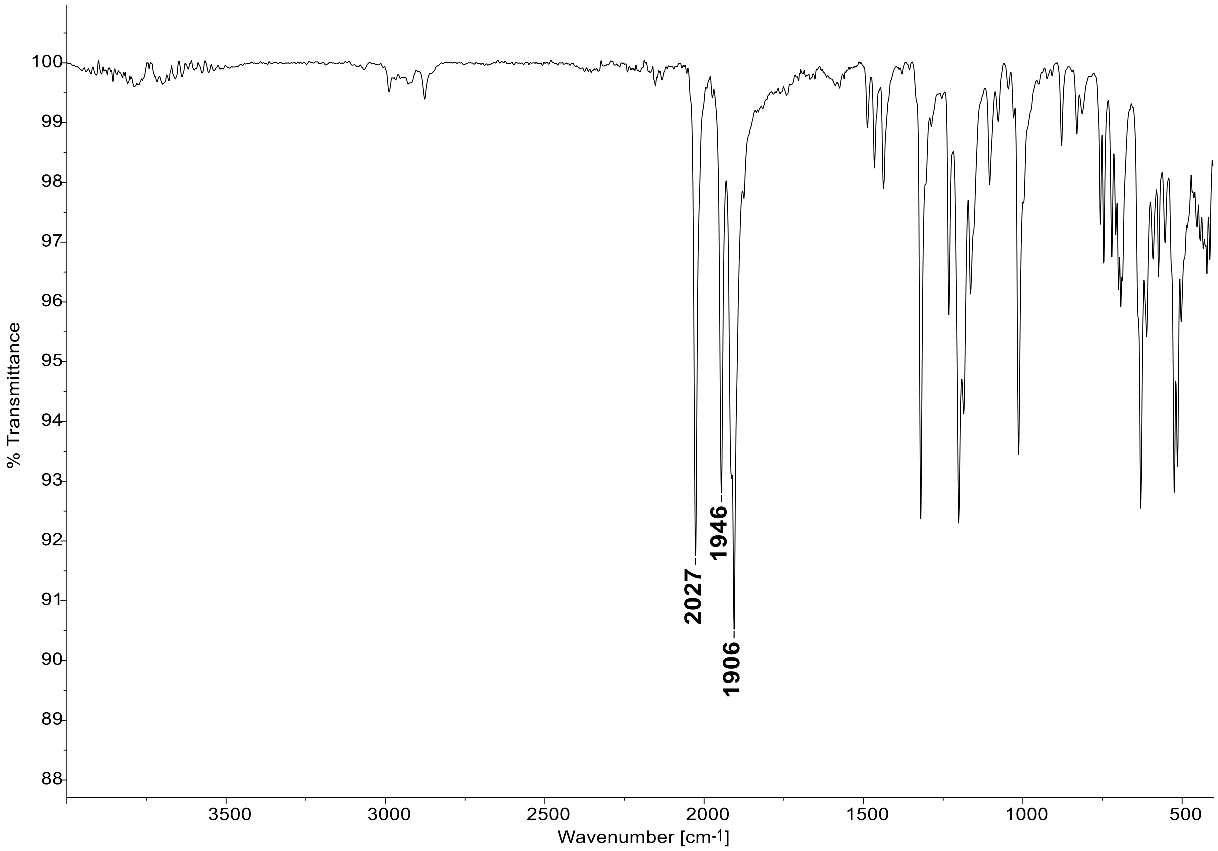

Figure S76. IR (ATR, cm^{-1}) of *fac*-[Mn($P^{Ph}N^{Pyrr}$)$(CO)_3$OTf] (**2c**)

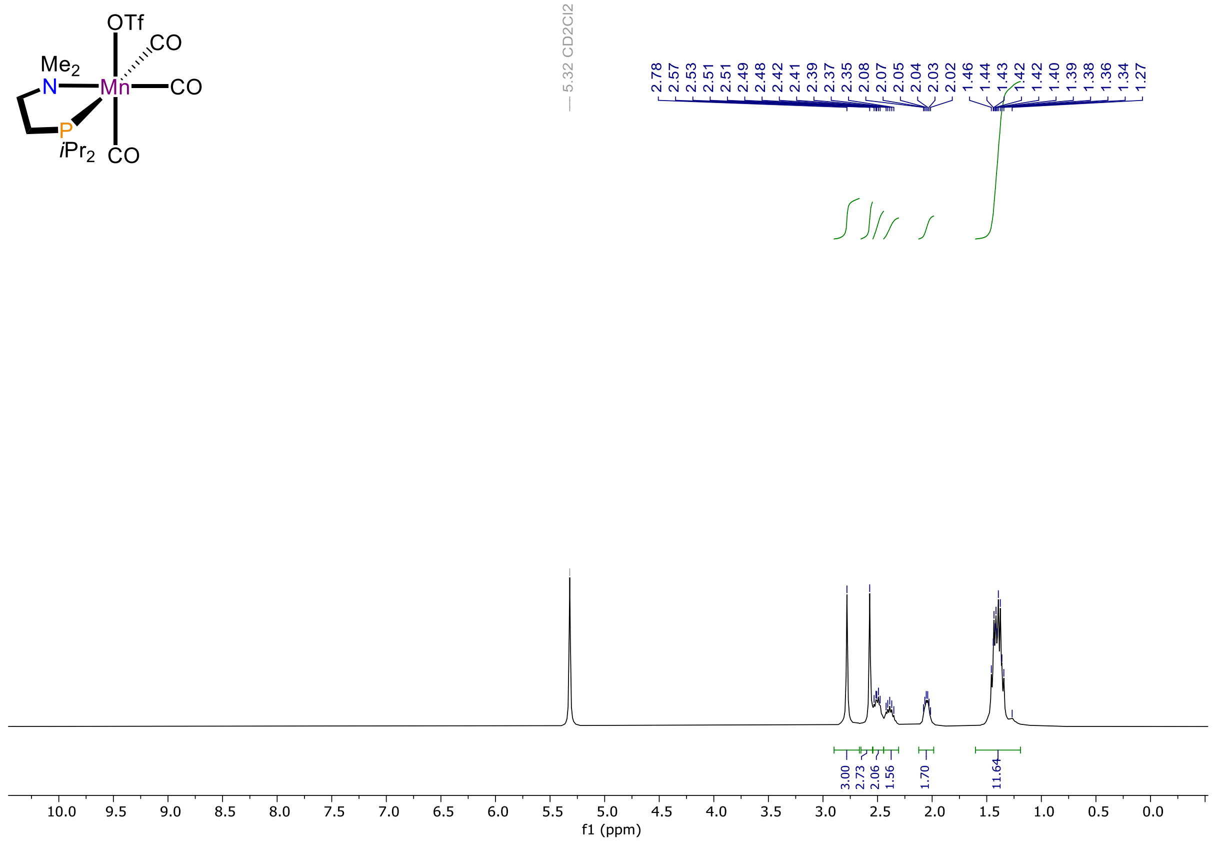

Figure S77. ^{1}H NMR (400 MHz, CD_2Cl_2) of *fac*-[Mn($P^{iPr}N^{Me}$)(CO)$_3$OTf] (**2d**)

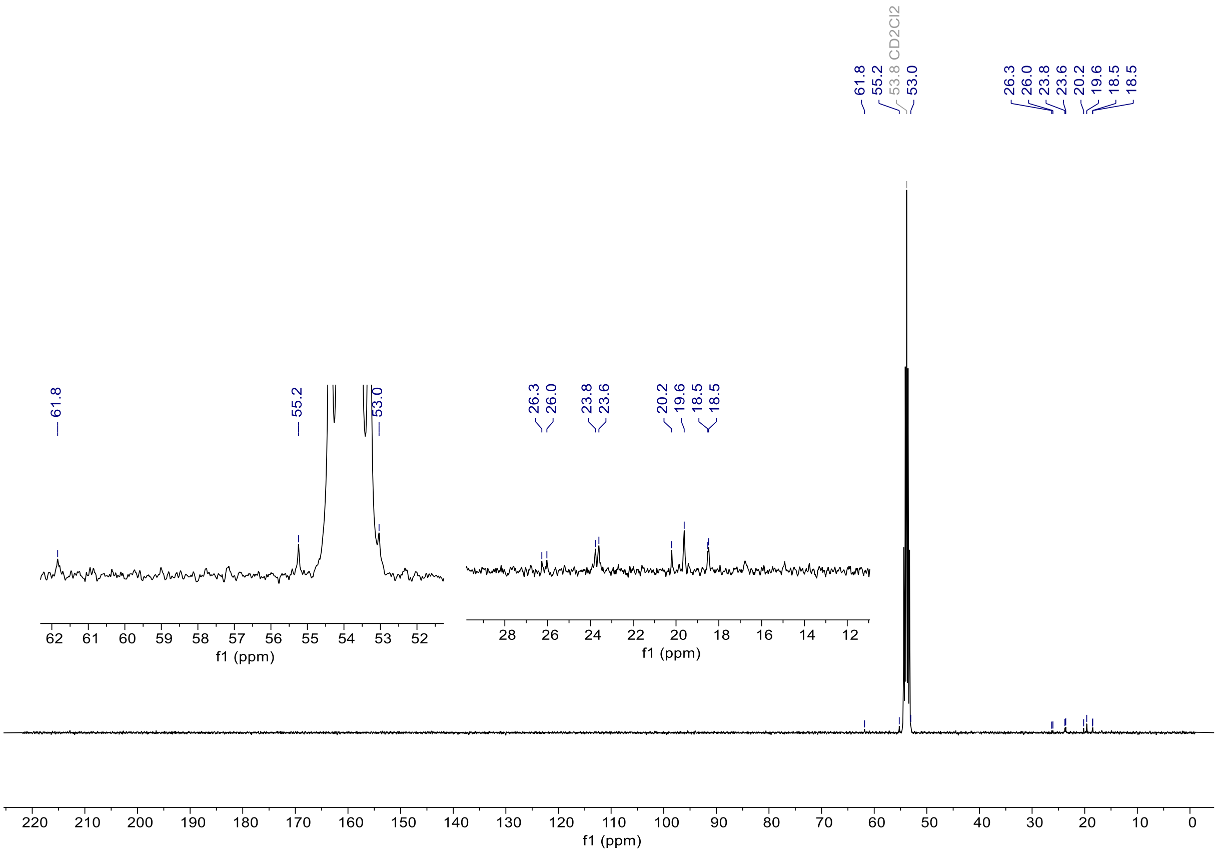

Figure S78. $^{13}C\{^{1}H\}$ NMR (101 MHz, CD_2Cl_2) of *fac*-[Mn($P^{iPr}N^{Me}$)(CO)$_3$OTf] (**2d**)

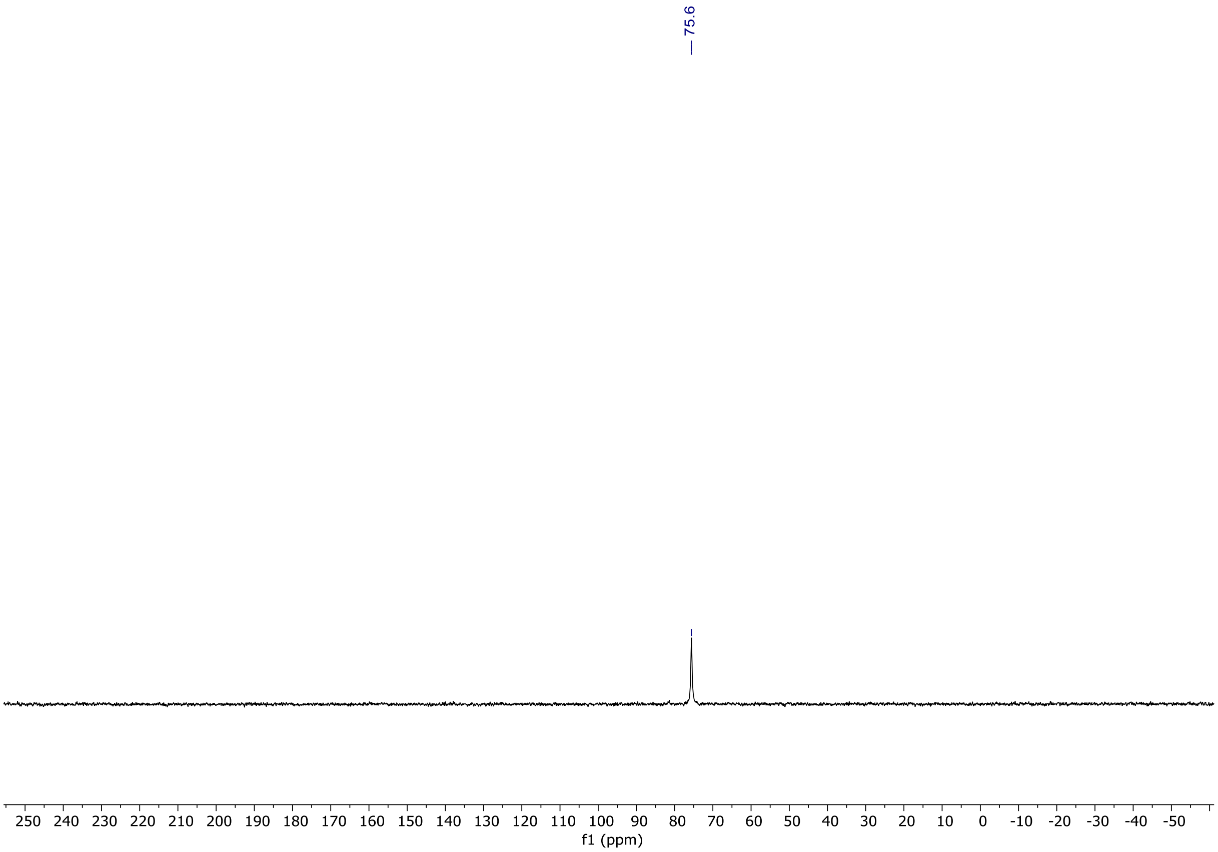

Figure S79. $^{31}P\{^{1}H\}$ NMR (162 MHz, CD_2Cl_2) of *fac*-[Mn($P^{iPr}N^{Me}$)$(CO)_3$OTf] (**2d**)

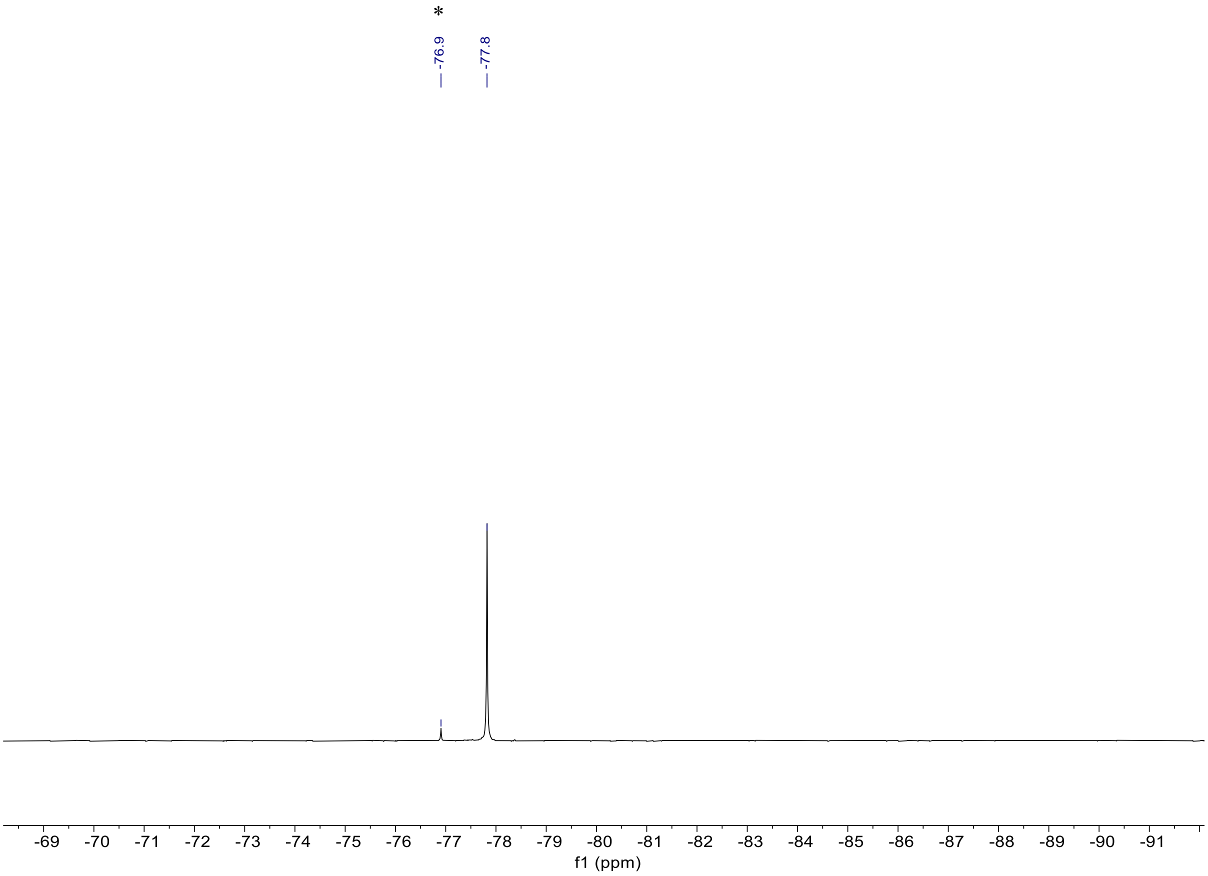

* = AgOTf

Figure S80. $^{19}F\{^{1}H\}$ NMR (376 MHz, CD_2Cl_2) of *fac*-[Mn($P^{iPr}N^{Me}$)$(CO)_3$OTf] (**2d**)

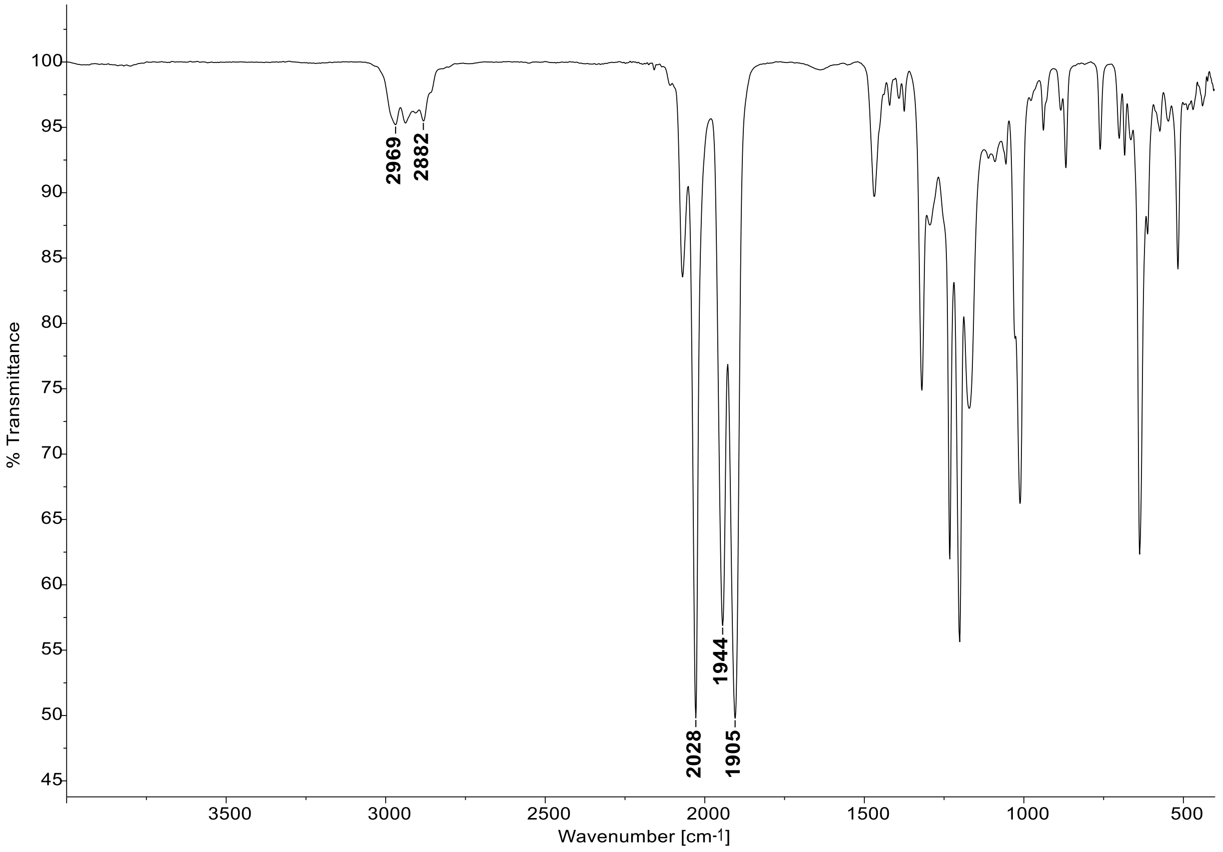

Figure S81. IR (ATR, cm^{-1}) of *fac*-[Mn(P^{iPr}N^{Me})(CO)$_3$OTf] (**2d**)

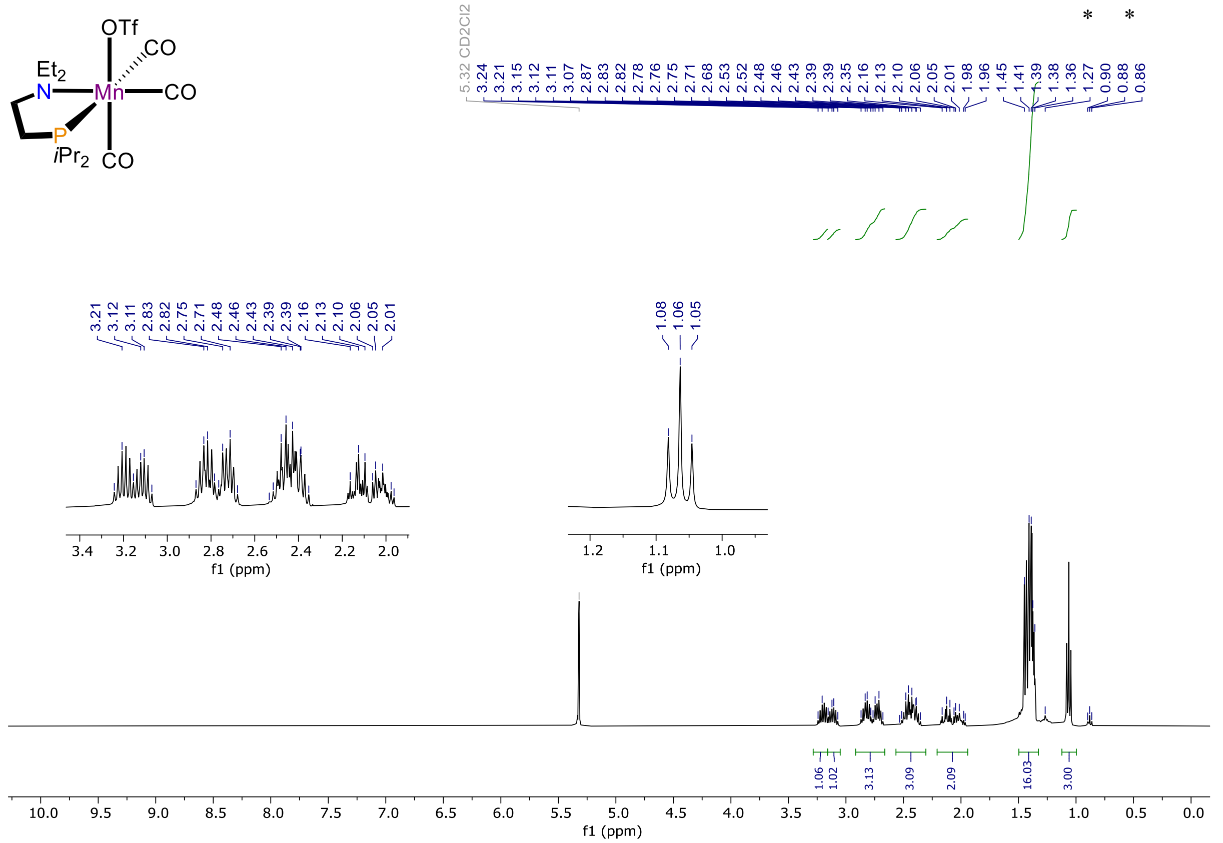

* = *n*-pentane

Figure S82. ^{1}H NMR (400 MHz, CD_2Cl_2) of *fac*-[Mn(P^{iPr}N^{Et})(CO)$_3$OTf] (**2e**)

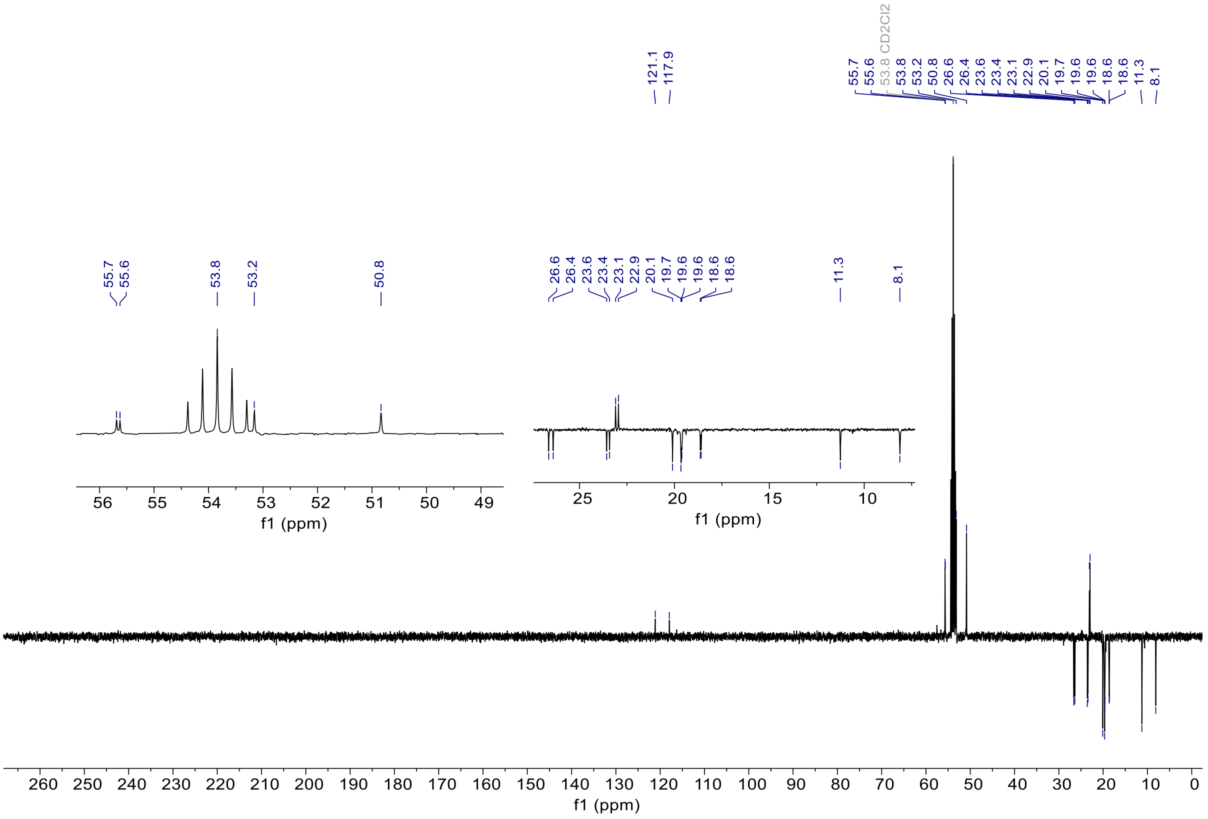

Figure S83. $^{13}C\{^{1}H\}$ NMR (101 MHz, CD_2Cl_2) of *fac*-[Mn($P^{iPr}N^{Et}$)$(CO)_3$OTf] (**2e**)

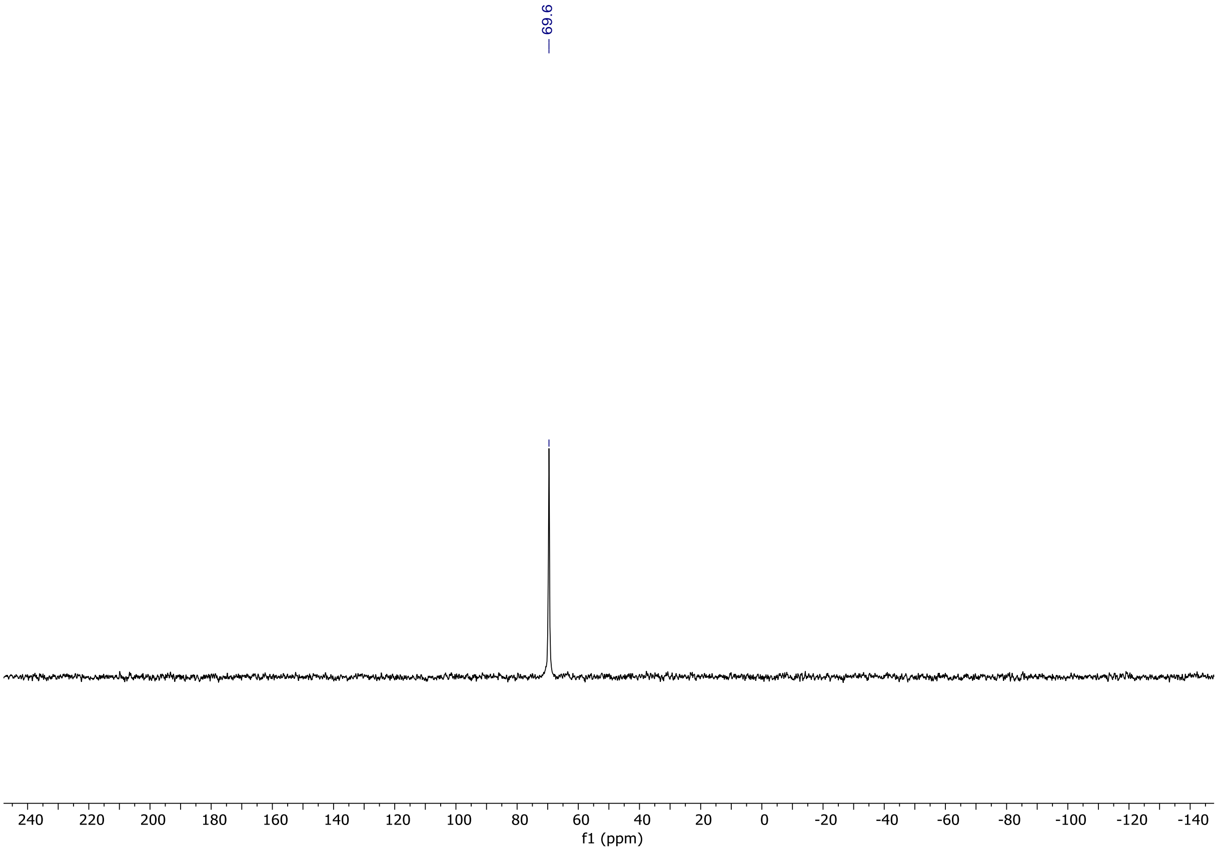

Figure S84. $^{31}P\{^{1}H\}$ NMR (162 MHz, CD_2Cl_2) of *fac*-[Mn($P^{iPr}N^{Et}$)$(CO)_3$OTf] (**2e**)

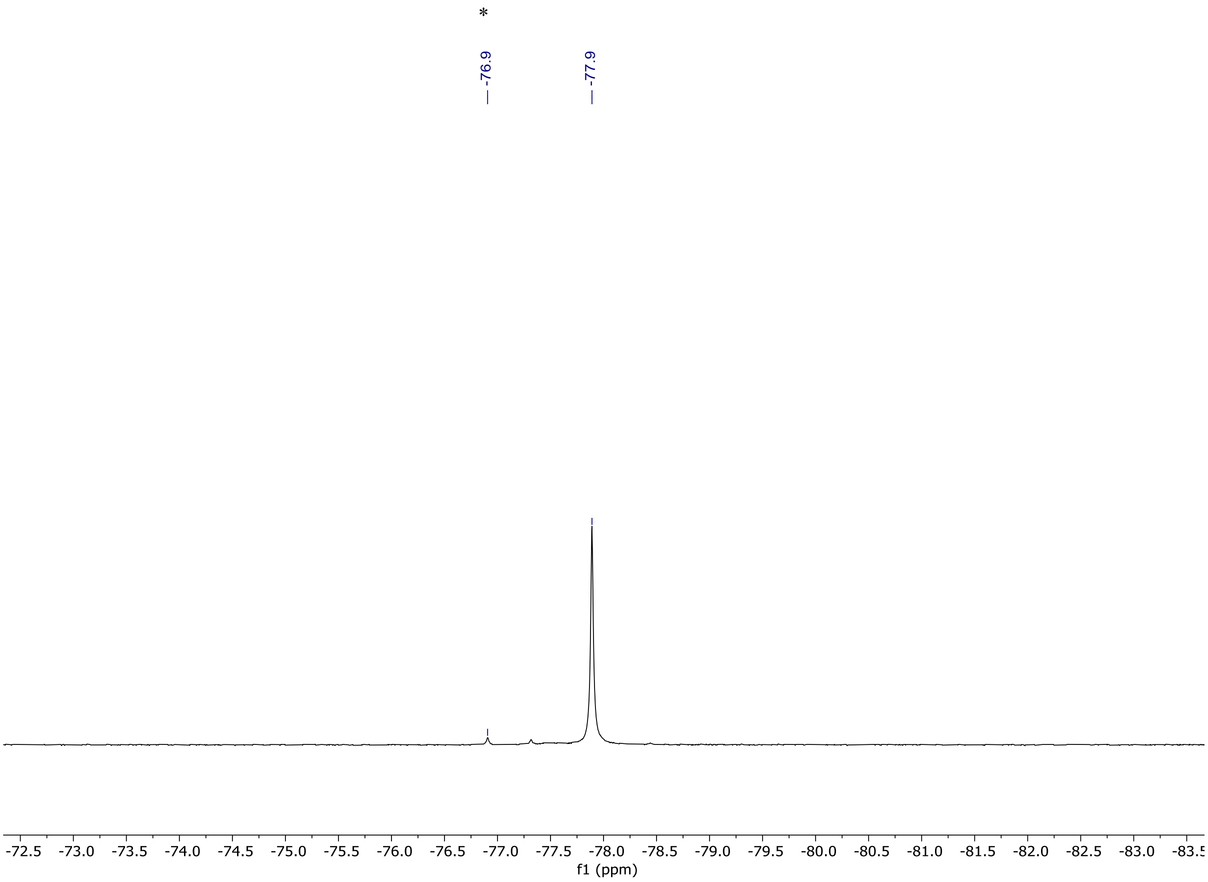

* = AgOTf

Figure S85. $^{19}F\{^{1}H\}$ NMR (376 MHz, CD_2Cl_2) of *fac*-[Mn($P^{iPr}N^{Et}$)$(CO)_3$OTf] (**2e**)

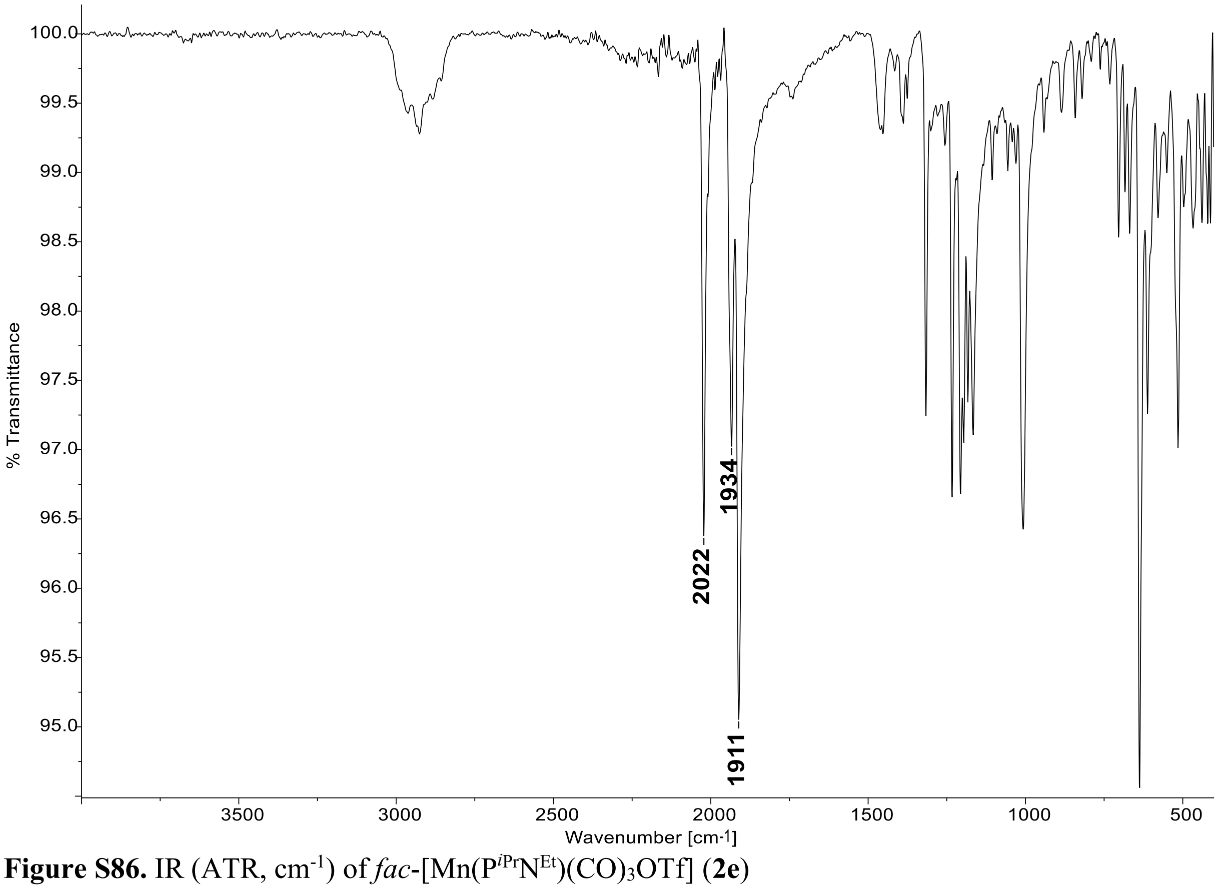

Figure S86. IR (ATR, cm^{-1}) of *fac*-[Mn($P^{iPr}N^{Et}$)$(CO)_3$OTf] (**2e**)

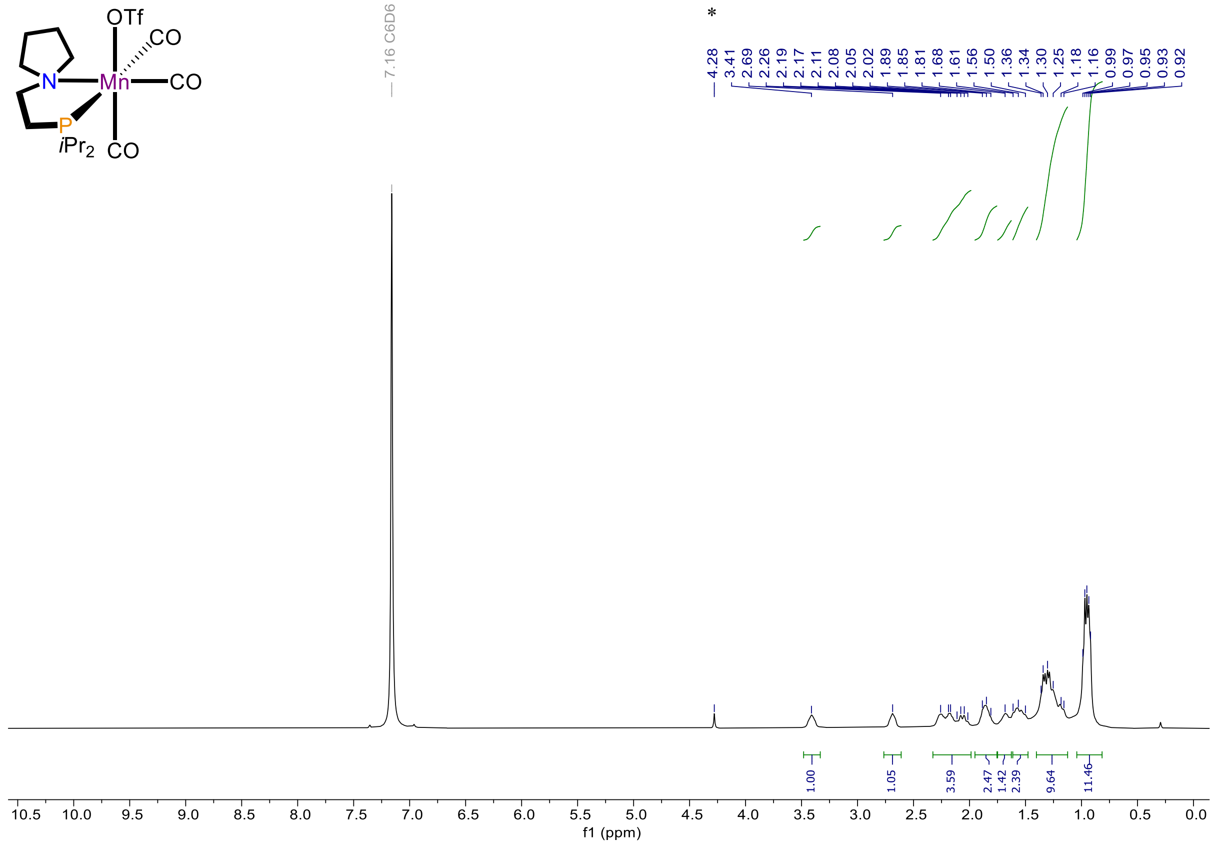

* = dichloromethane

Figure S87. ^{1}H NMR (400 MHz, C_6D_6) of *fac*-[Mn(P^{iPr}N^{Pyrr})(CO)$_3$OTf] (**2f**)

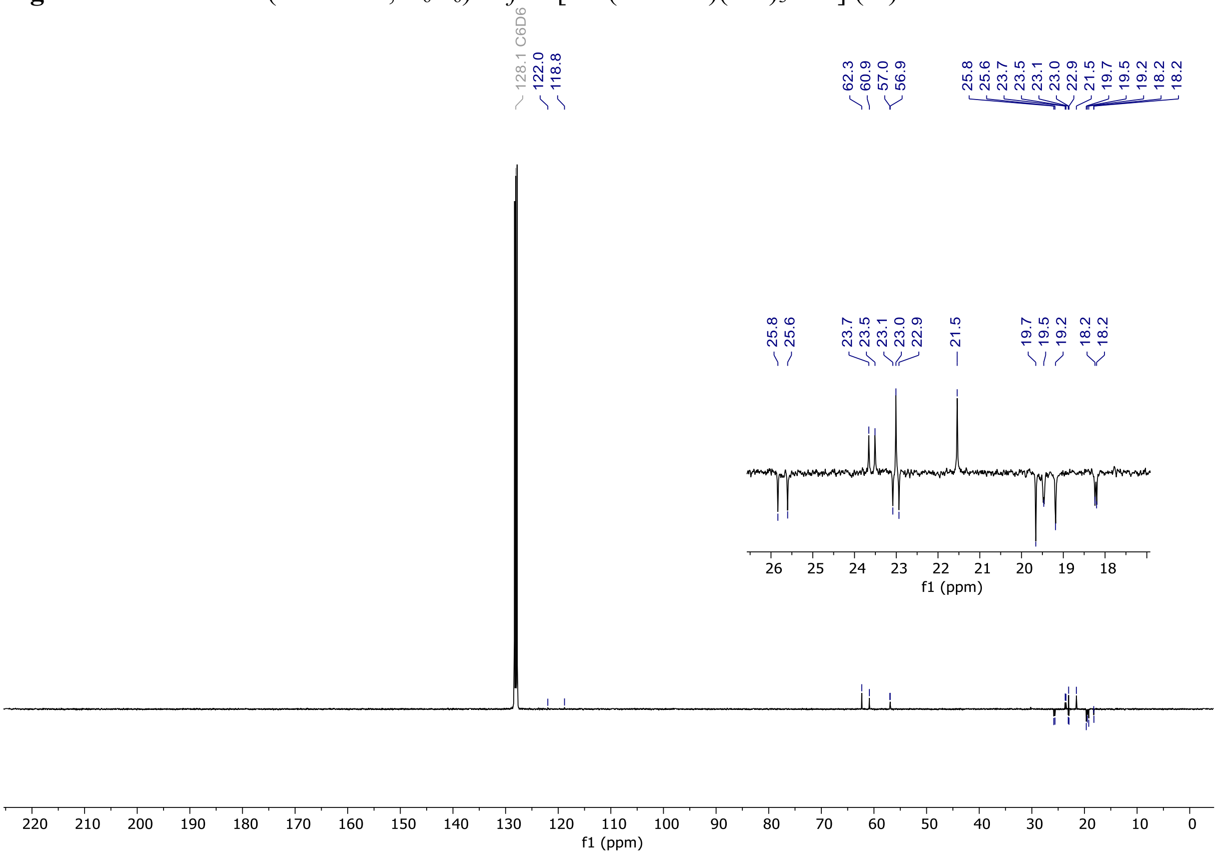

Figure S88. ^{13}C{^{1}H} NMR (101 MHz, C_6D_6) of *fac*-[Mn(P^{iPr}N^{Pyrr})(CO)$_3$OTf] (**2f**)

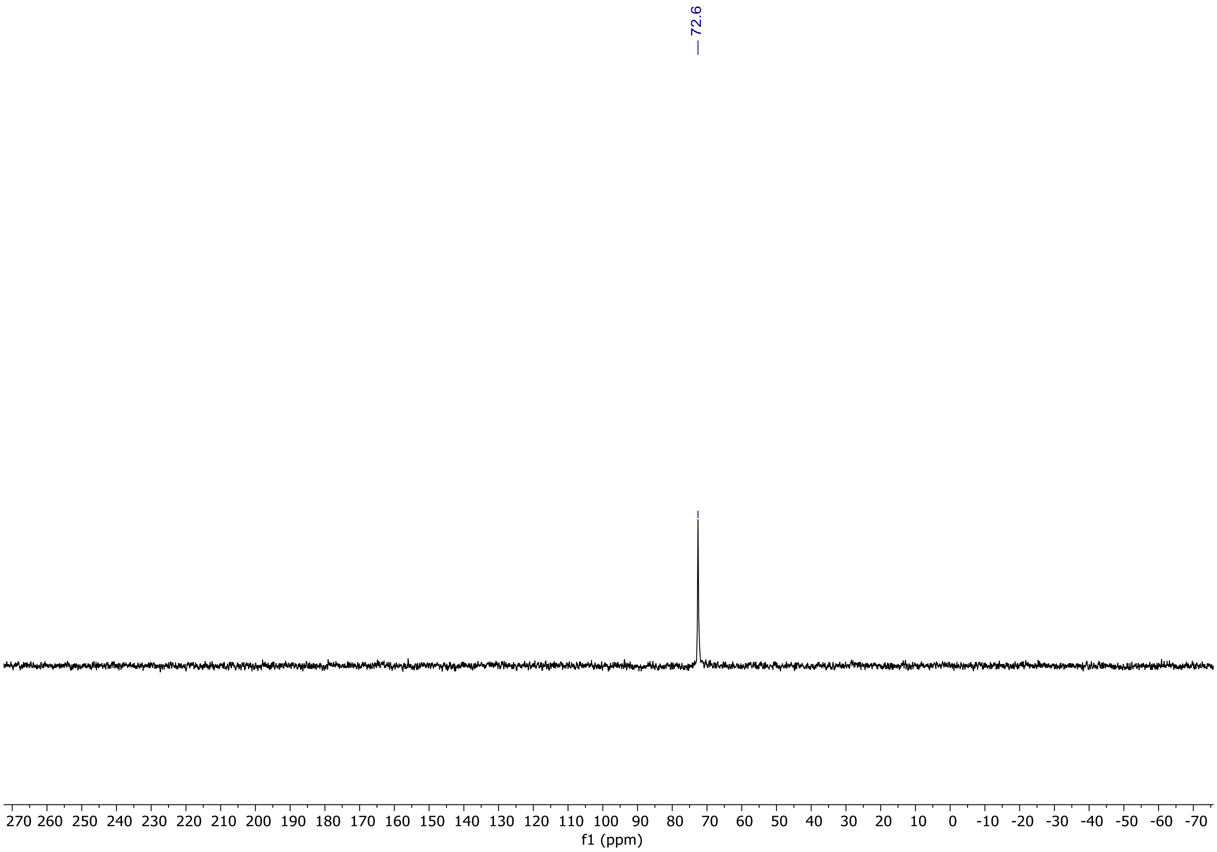

Figure S89. $^{31}P\{^{1}H\}$ NMR (162 MHz, C_6D_6) of *fac*-[Mn($P^{iPr}N^{Pyrr}$)$(CO)_3$OTf] (**2f**)

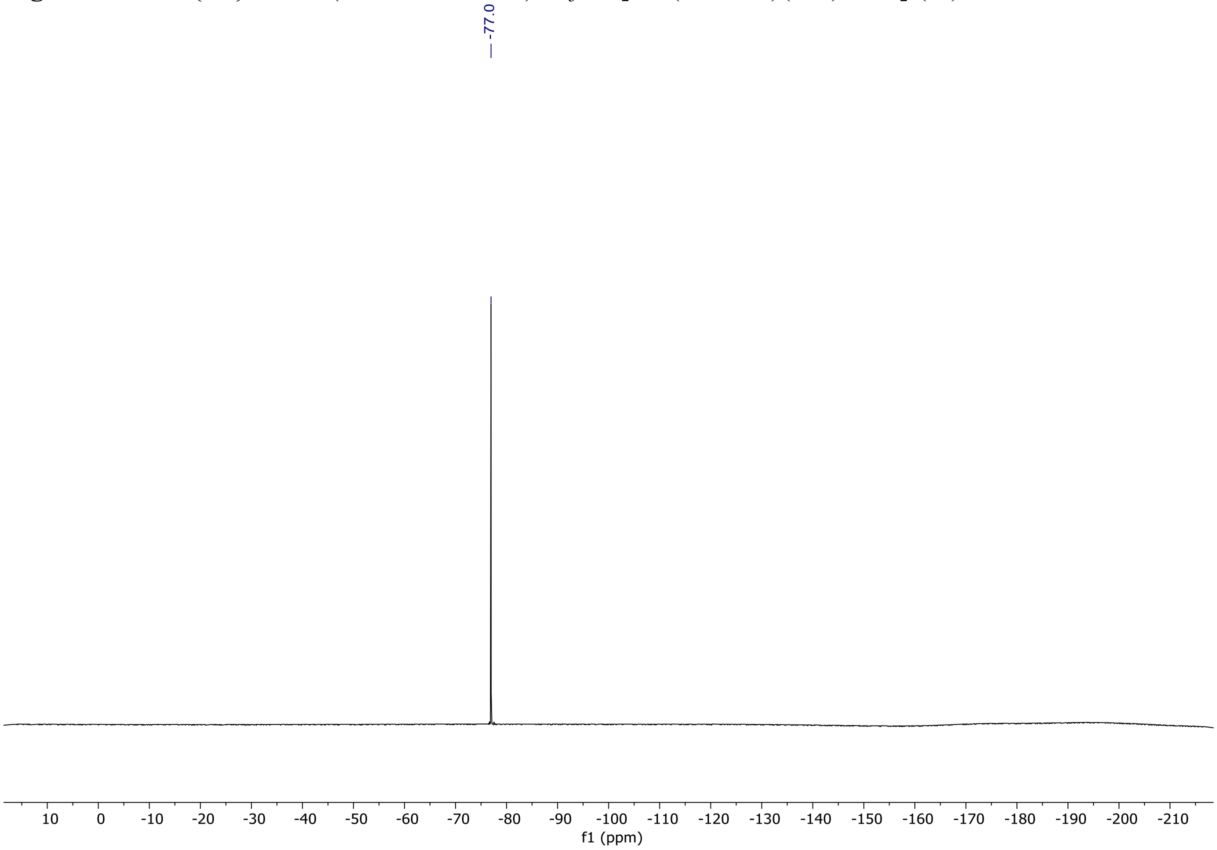

Figure S90. $^{19}F\{^{1}H\}$ NMR (376 MHz, CD_2Cl_2) of *fac*-[Mn($P^{iPr}N^{Pyrr}$)$(CO)_3$OTf] (**2f**)

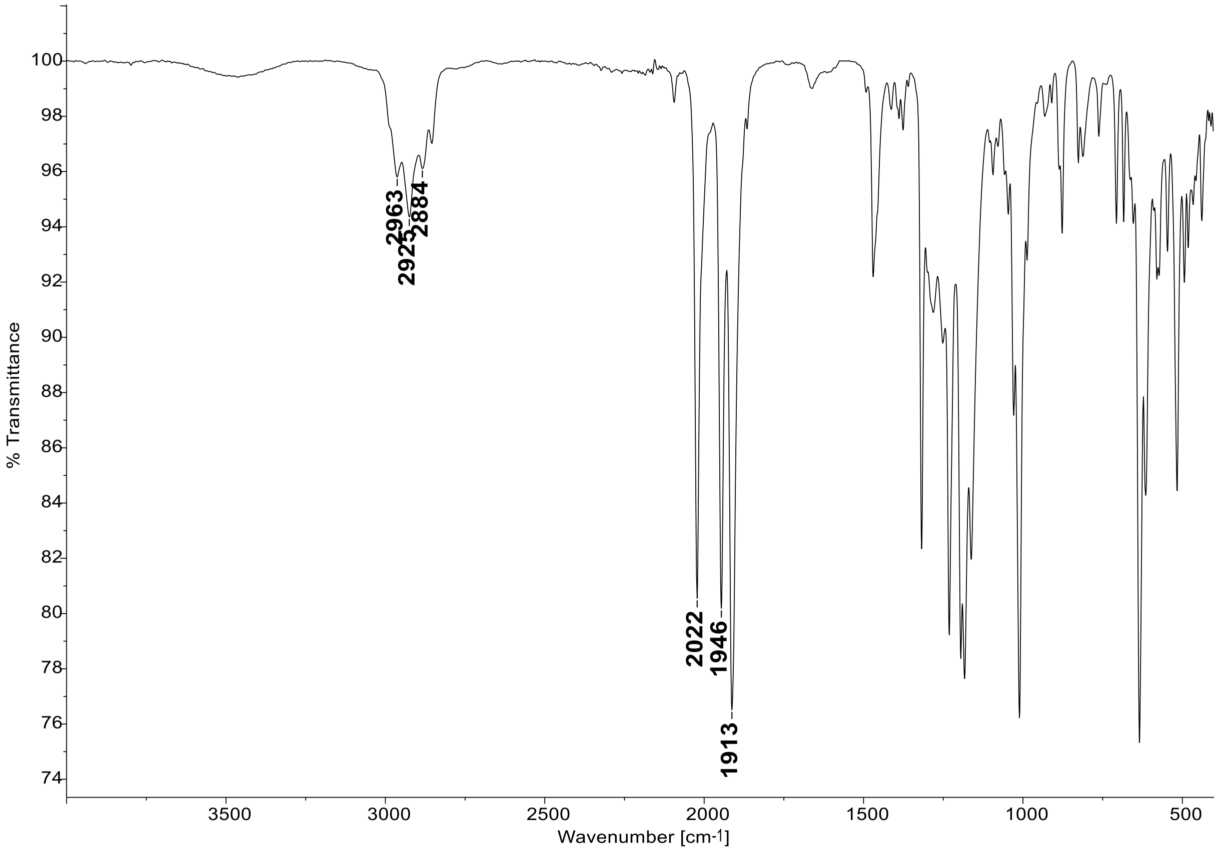

Figure S91. IR (ATR, cm^{-1}) of *fac*-[Mn($P^{iPr}N^{Pyrr}$)$(CO)_3$OTf] (**2f**)

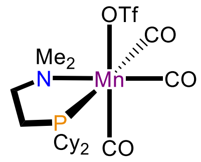

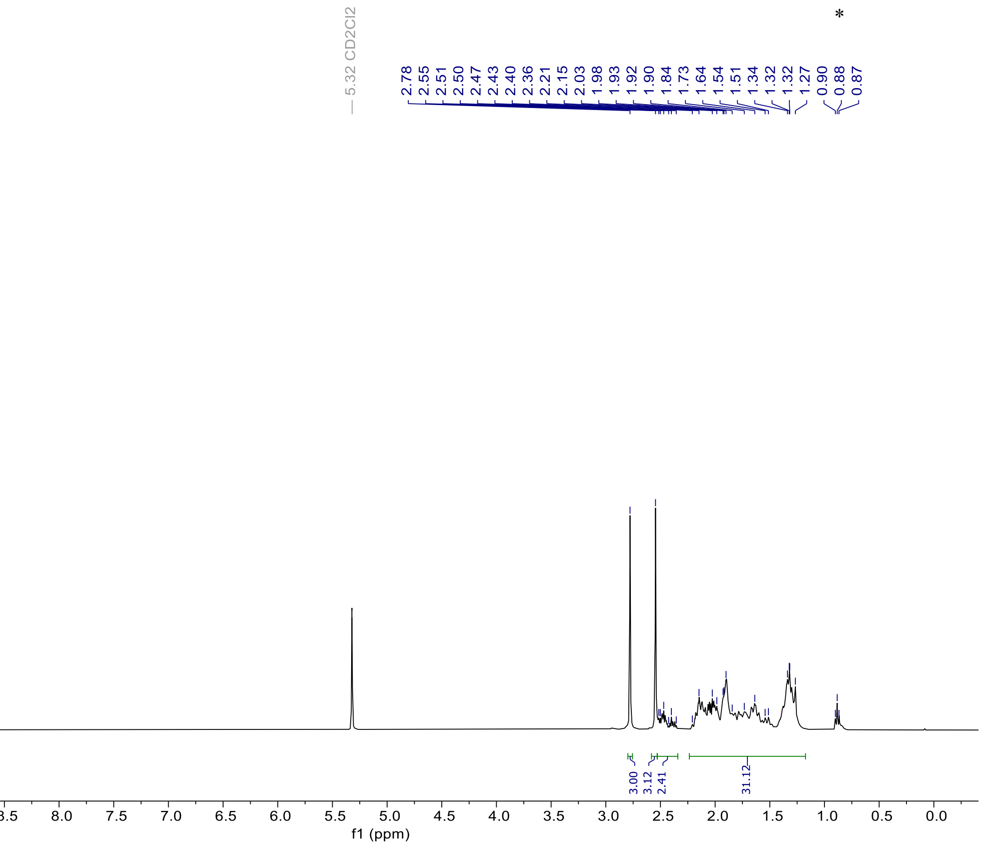

* = *n*-pentane

Figure S92. ^{1}H NMR (400 MHz, CD_2Cl_2) of *fac*-[Mn($P^{Cy}N^{Me}$)$(CO)_3$OTf] (**2g**)

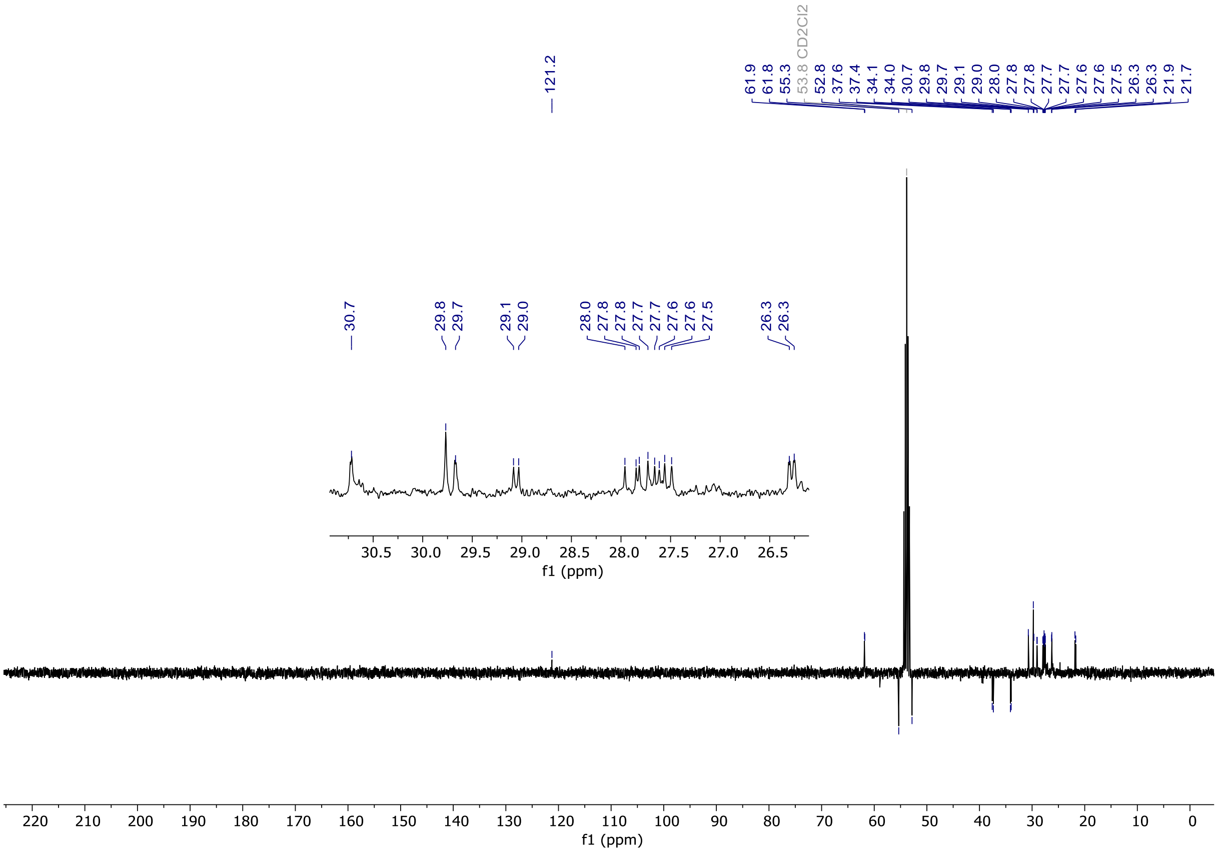

Figure S93. $^{13}C\{^{1}H\}$ NMR (101 MHz, CD_2Cl_2) of *fac*-[Mn($P^{Cy}N^{Me}$)$(CO)_3$OTf] (**2g**)

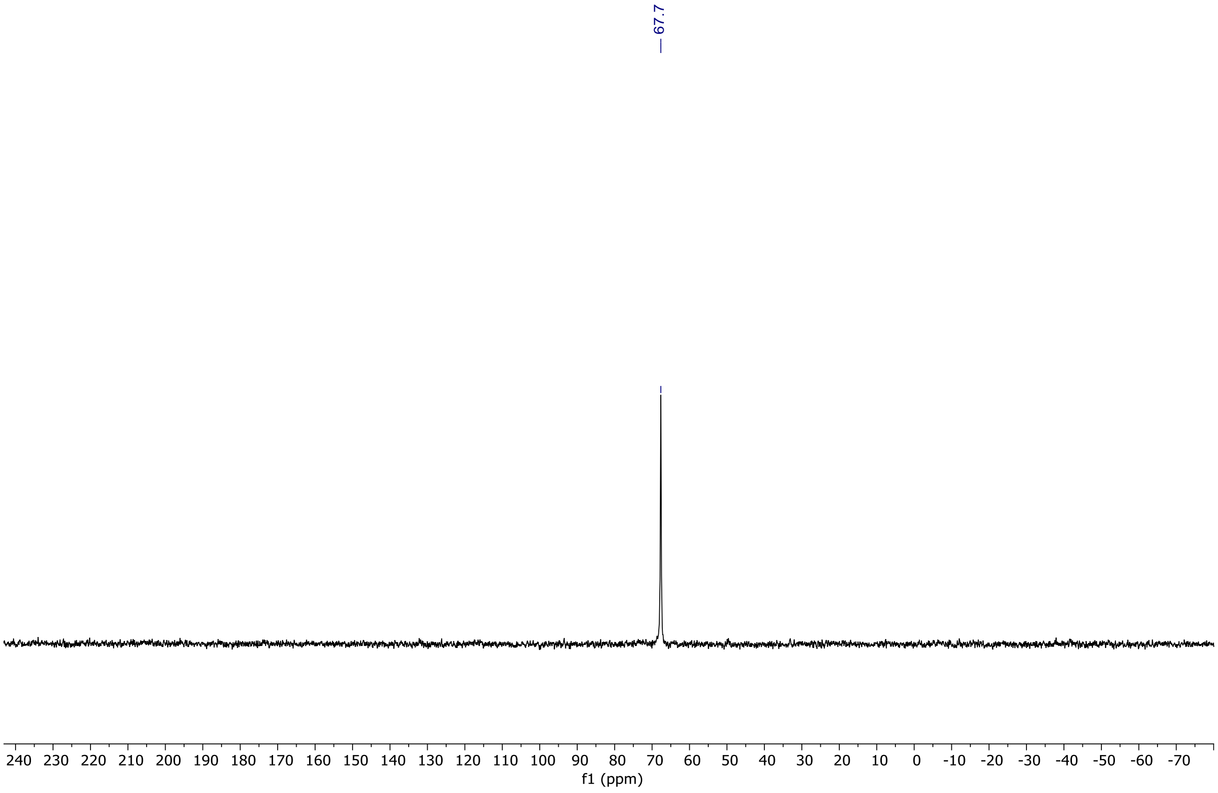

Figure S94. ^{31}P NMR (162 MHz, CD_2Cl_2) of *fac*-[Mn($P^{Cy}N^{Me}$)$(CO)_3$OTf] (**2g**)

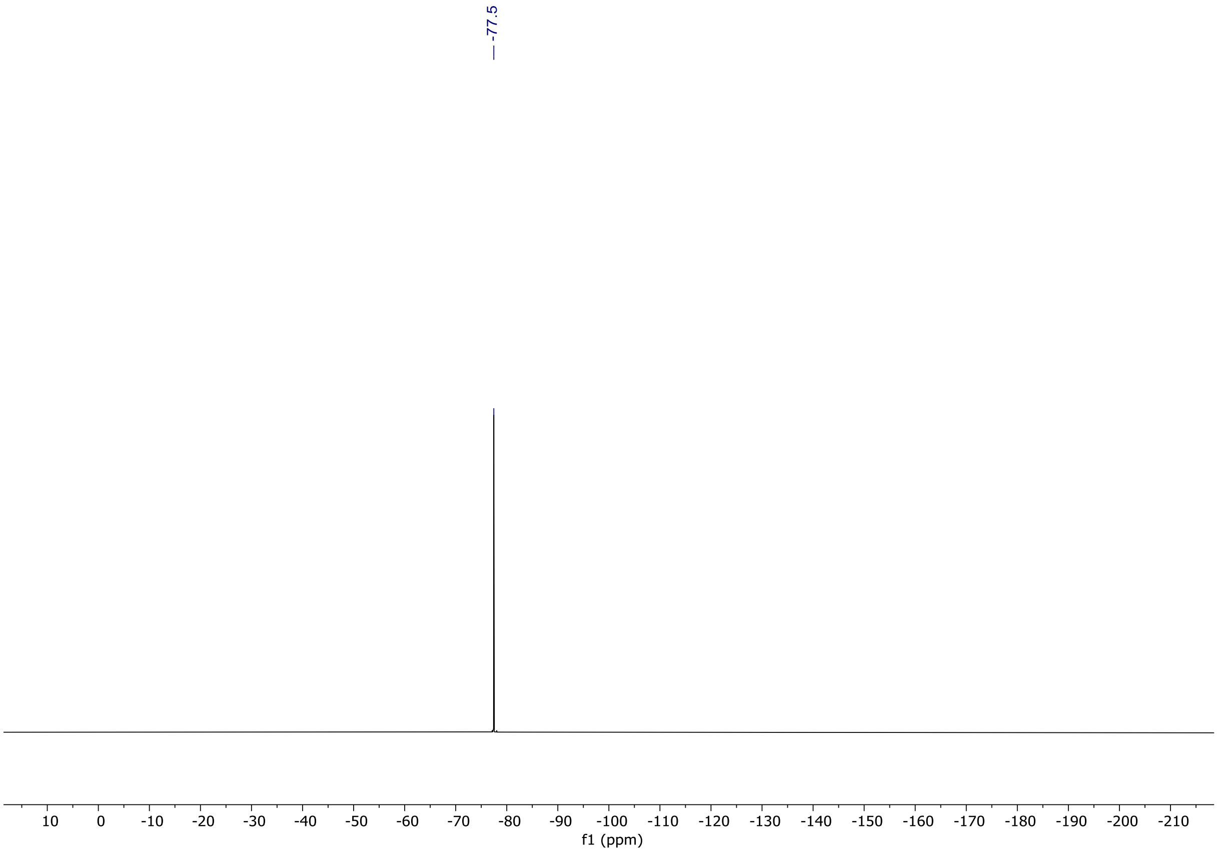

Figure S95. ^{19}F NMR (376 MHz, CD_2Cl_2) of *fac*-[Mn($P^{Cy}N^{Me}$)(CO)$_3$OTf] (**2g**)

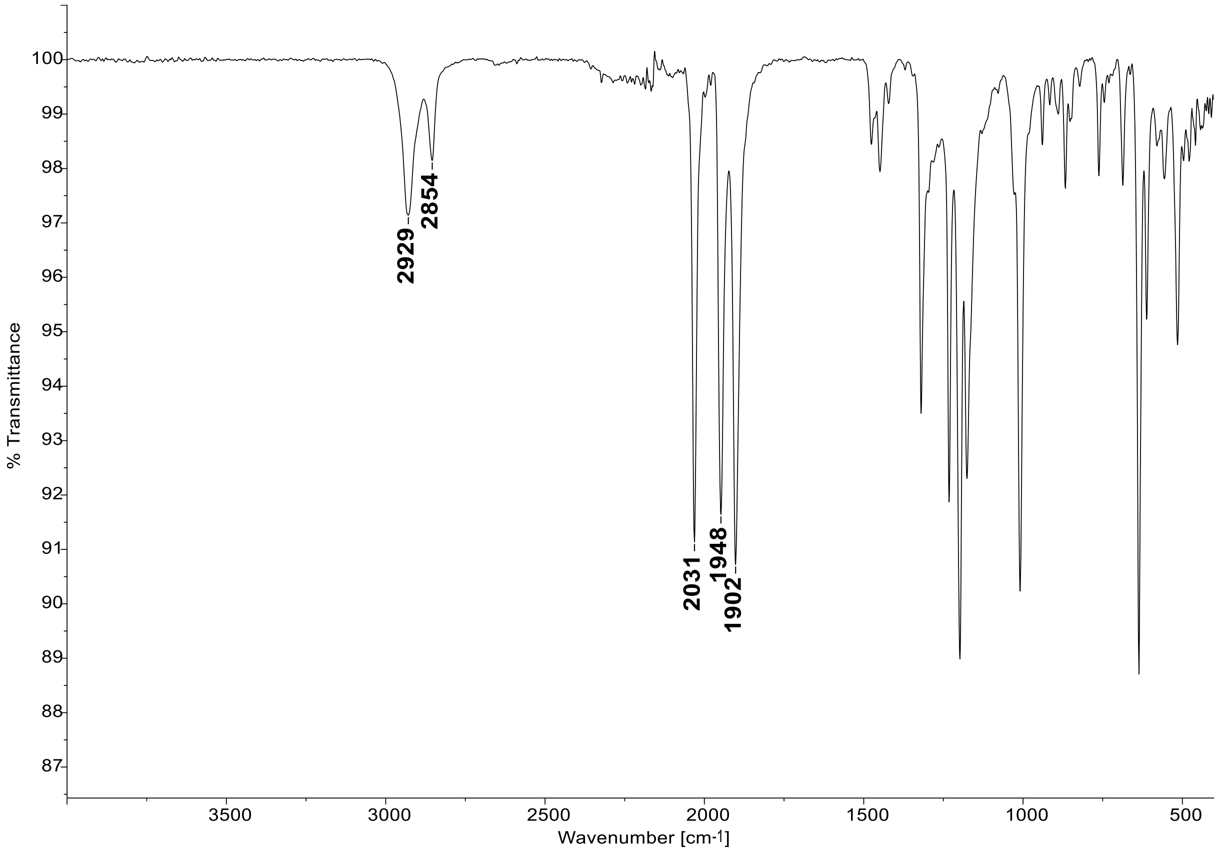

Figure S96. IR (ATR, cm^{-1}) of *fac*-[Mn($P^{Cy}N^{Me}$)(CO)$_3$OTf] (**2g**)

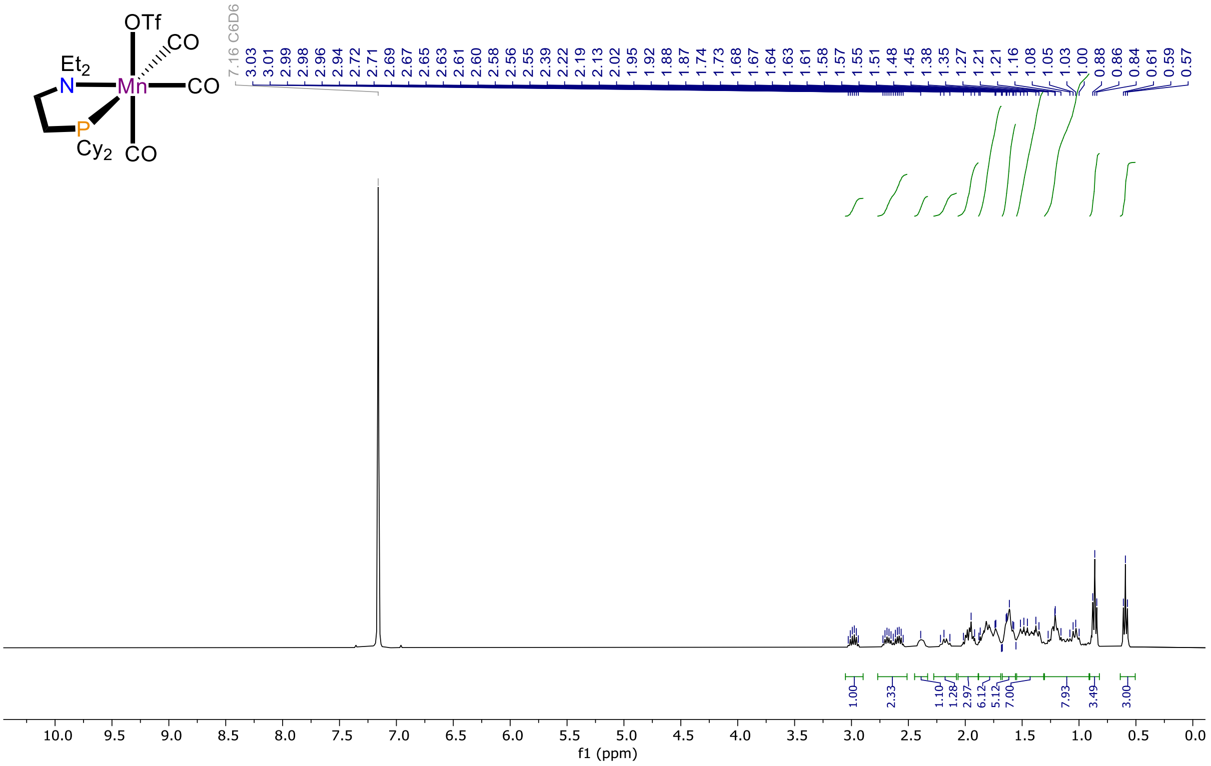

Figure S97. ^{1}H NMR (400 MHz, C_6D_6) of *fac*-[Mn($P^{Cy}N^{Et}$)$(CO)_3$OTf] (**2h**)

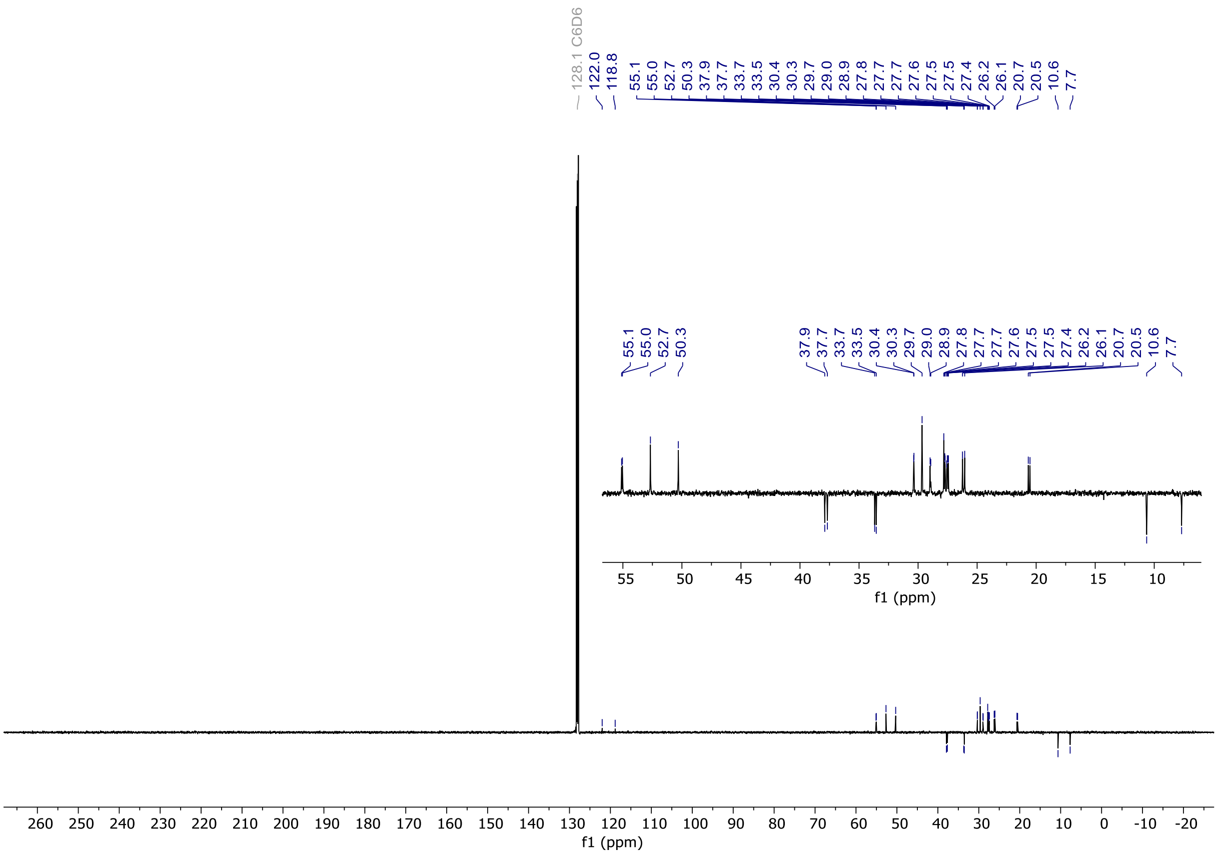

Figure S98. $^{13}C\{^{1}H\}$ NMR (101 MHz, C_6D_6) of *fac*-[Mn($P^{Cy}N^{Et}$)$(CO)_3$OTf] (**2h**)

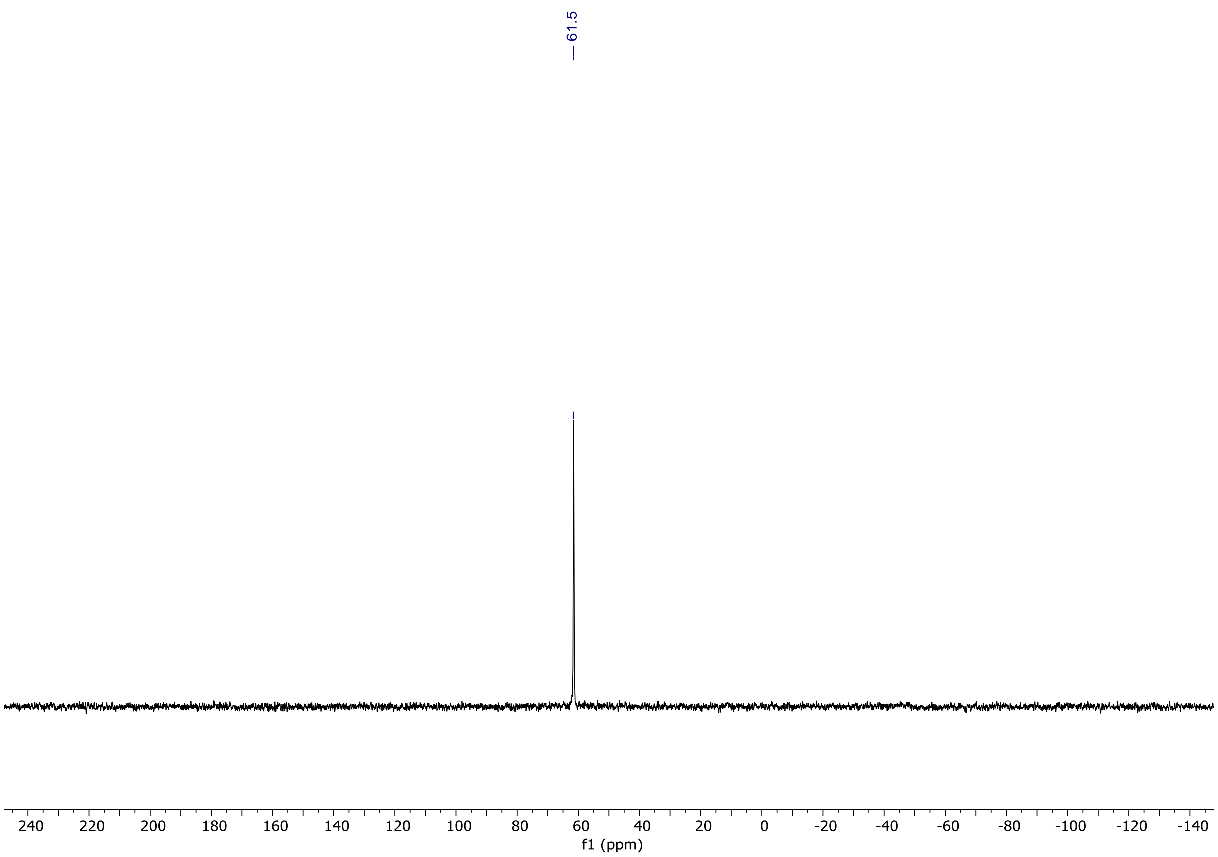

Figure S99. ^{31}P NMR (162 MHz, C_6D_6) of *fac*-[Mn($P^{Cy}N^{Et}$)$(CO)_3$OTf] (**2h**)

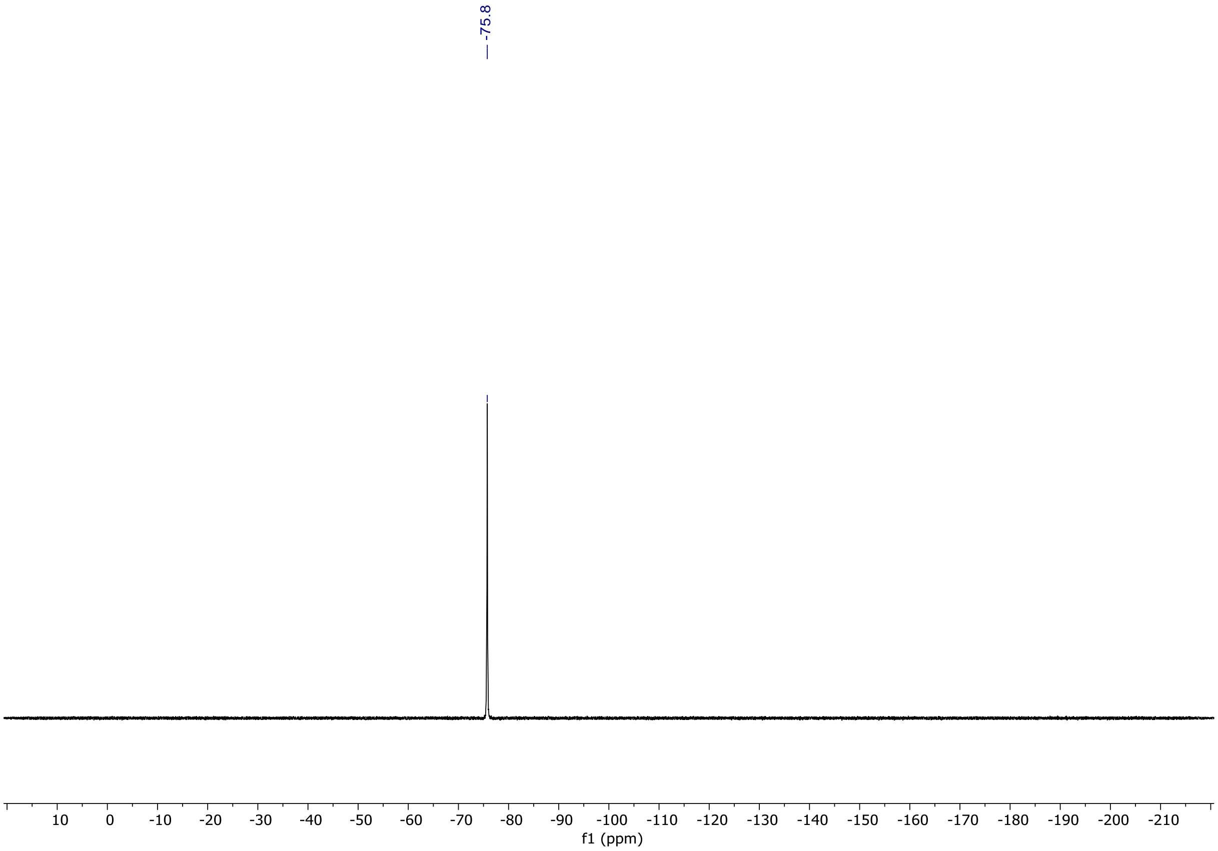

Figure S100. ^{19}F NMR (235 MHz, CD_2Cl_2) of *fac*-[Mn($P^{Cy}N^{Et}$)$(CO)_3$OTf] (**2h**)

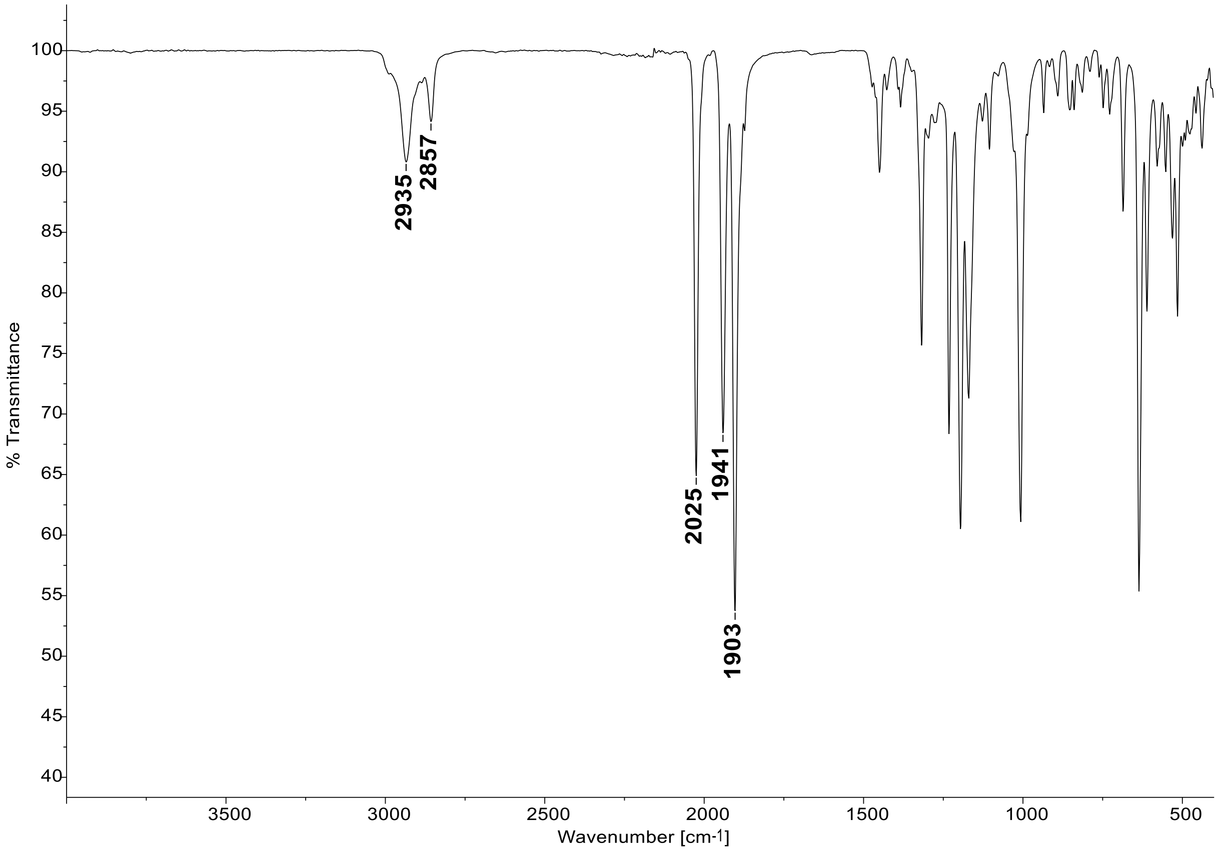

Figure S101. IR (ATR, cm^{-1}) of *fac*-[Mn($P^{Cy}N^{Et}$)$(CO)_3$OTf] (**2h**)

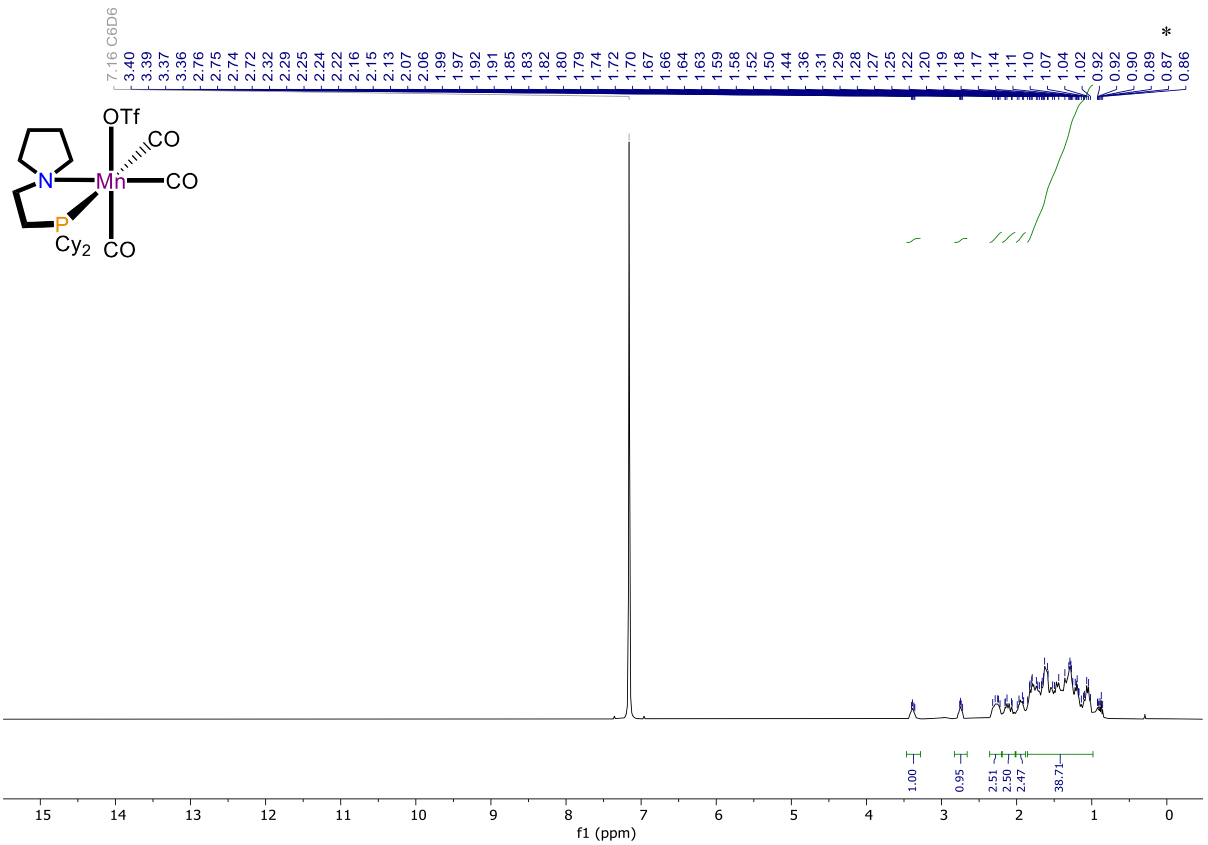

* = *n*-pentane

Figure S102. ^{1}H NMR (400 MHz, C_6D_6) of *fac*-[Mn($P^{Cy}N^{Pyrr}$)$(CO)_3$OTf] (**2i**)

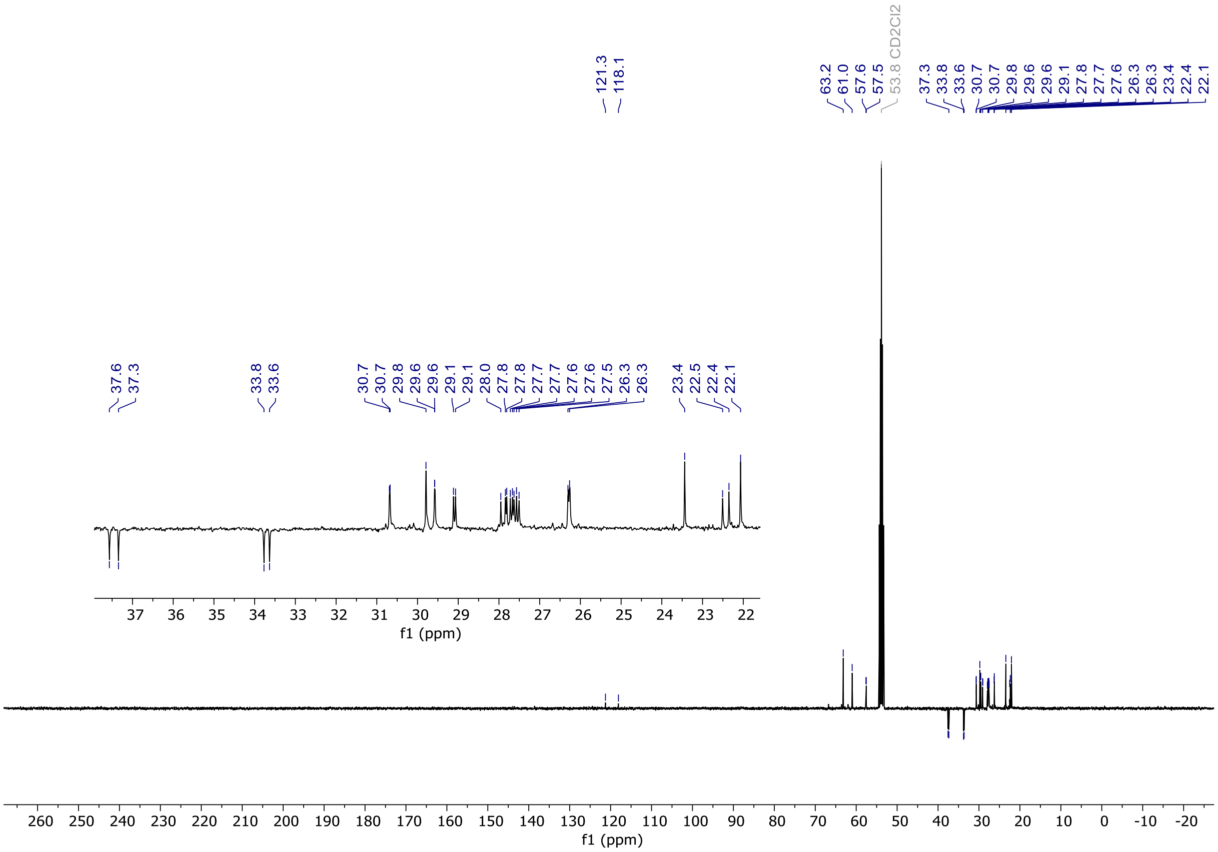

Figure S103. $^{13}C\{^{1}H\}$ NMR (101 MHz, CD_2Cl_2) of *fac*-[Mn($P^{Cy}N^{Pyrr}$)$(CO)_3$OTf] (**2i**)

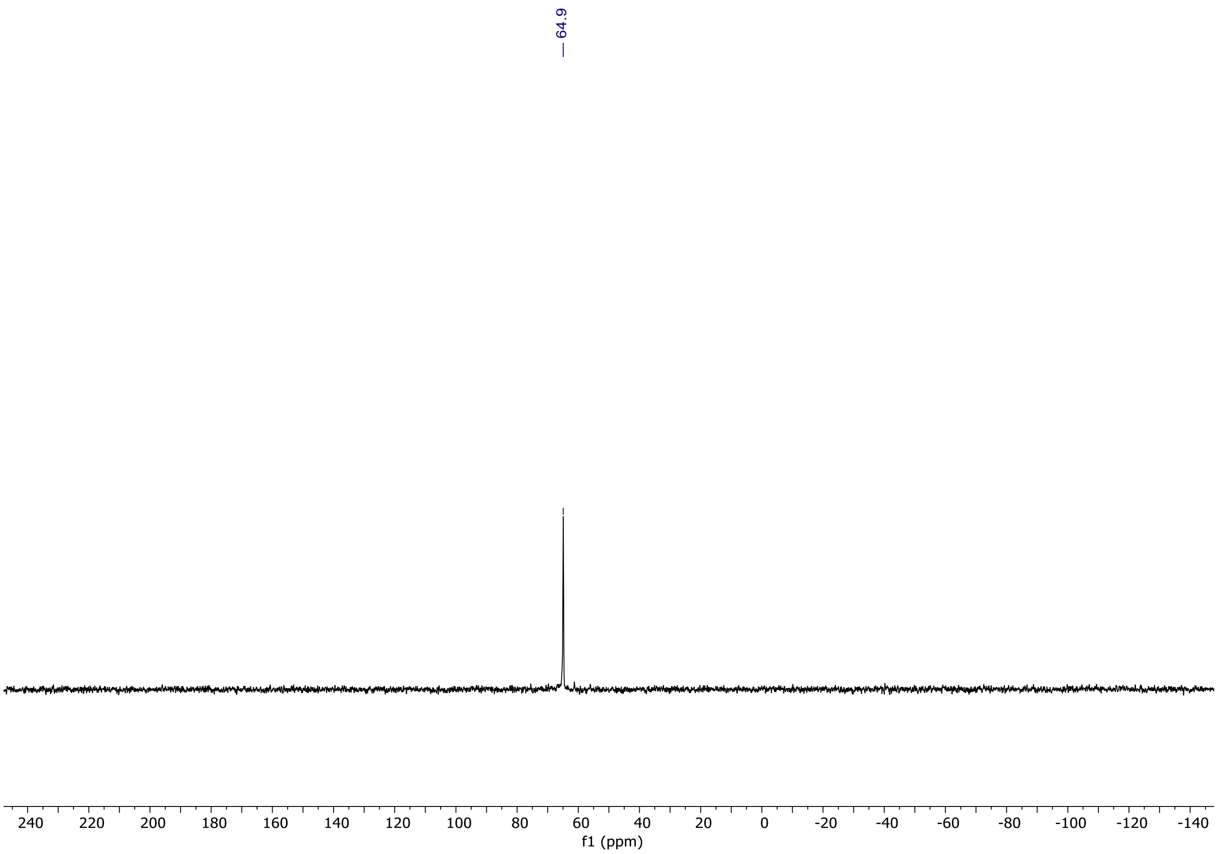

Figure S104. ^{31}P NMR (162 MHz, CD_2Cl_2) of *fac*-[Mn($P^{Cy}N^{Pyrr}$)$(CO)_3$OTf] (**2i**)

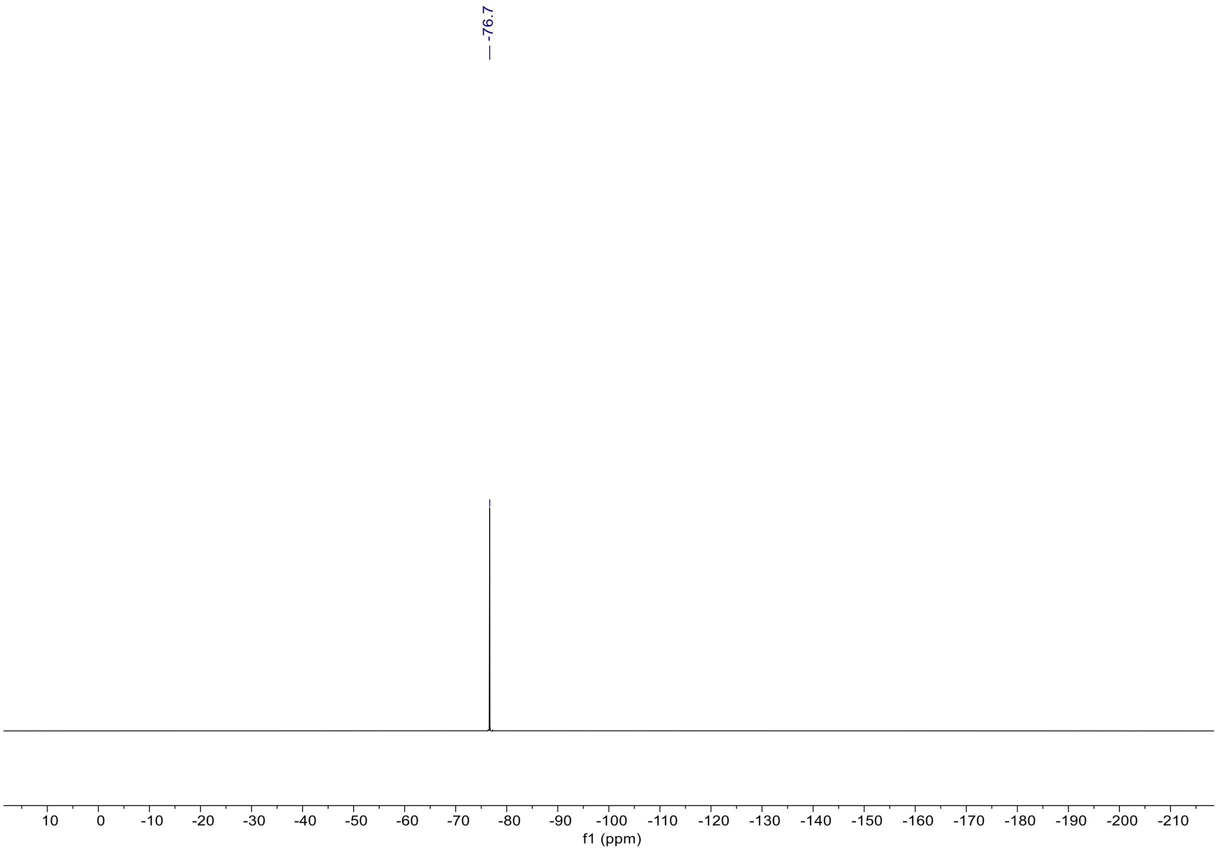

Figure S105. $^{19}F\{^{1}H\}$ NMR (376 MHz, C_6D_6) of *fac*-[Mn($P^{Cy}N^{Pyrr}$)$(CO)_3$OTf] (**2i**)

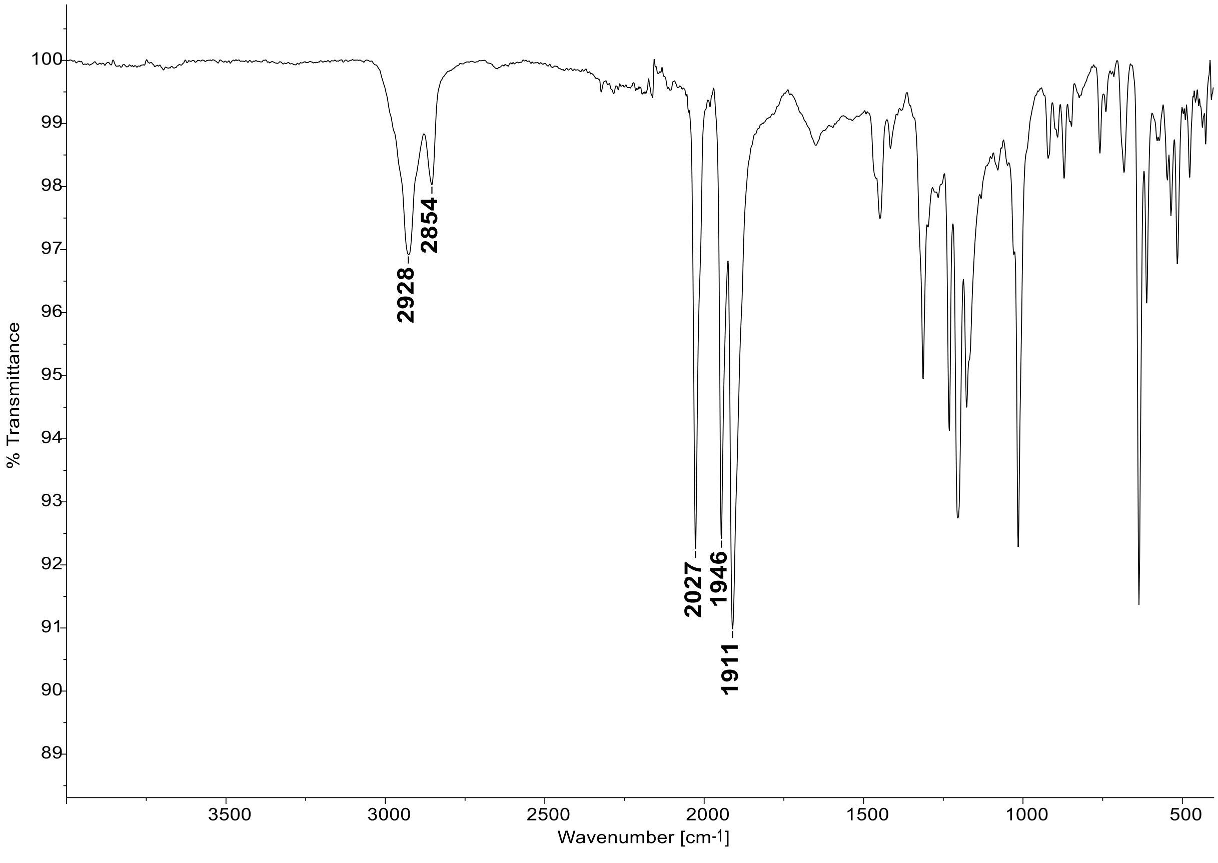

Figure S106. IR (ATR, cm^{-1}) of *fac*-[Mn($P^{Cy}N^{Pyrr}$)$(CO)_3$OTf] (**2i**)

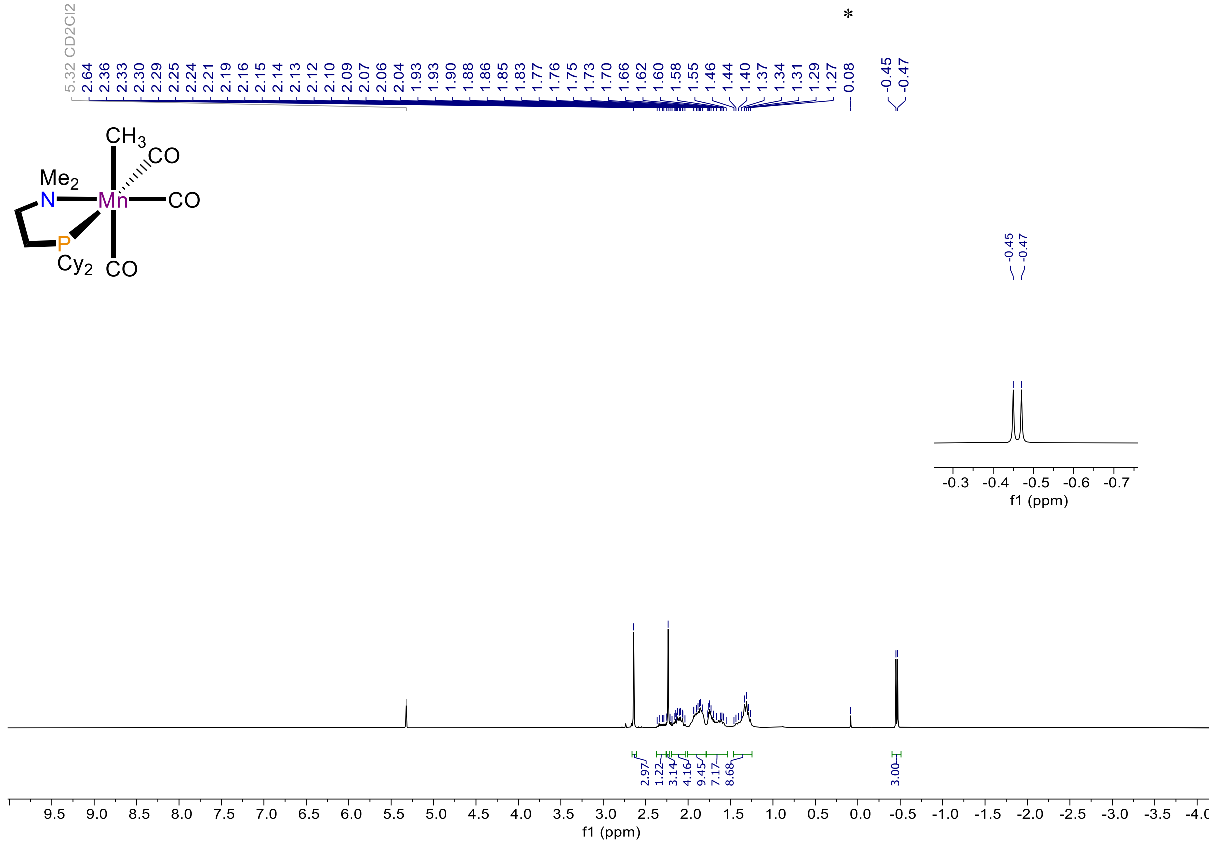

* = grease

Figure S107. ^{1}H NMR (400 MHz, CD_2Cl_2) of *fac*-[Mn($P^{Cy}N^{Me}$)$(CO)_3CH_3$] (**PN1**)

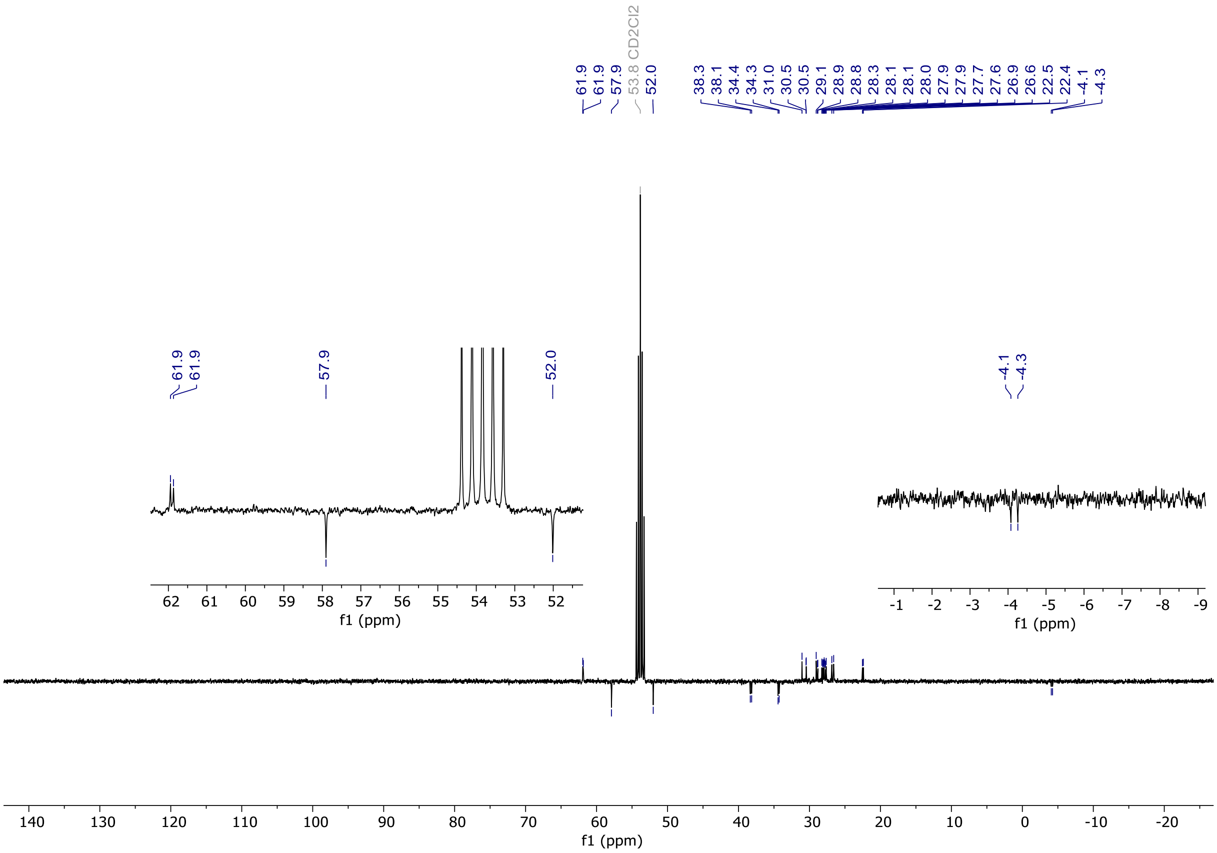

Figure S108. $^{13}C\{^{1}H\}$ NMR (101 MHz, CD_2Cl_2) of *fac*-[Mn($P^{Cy}N^{Me}$)$(CO)_3CH_3$] (**PN1**)

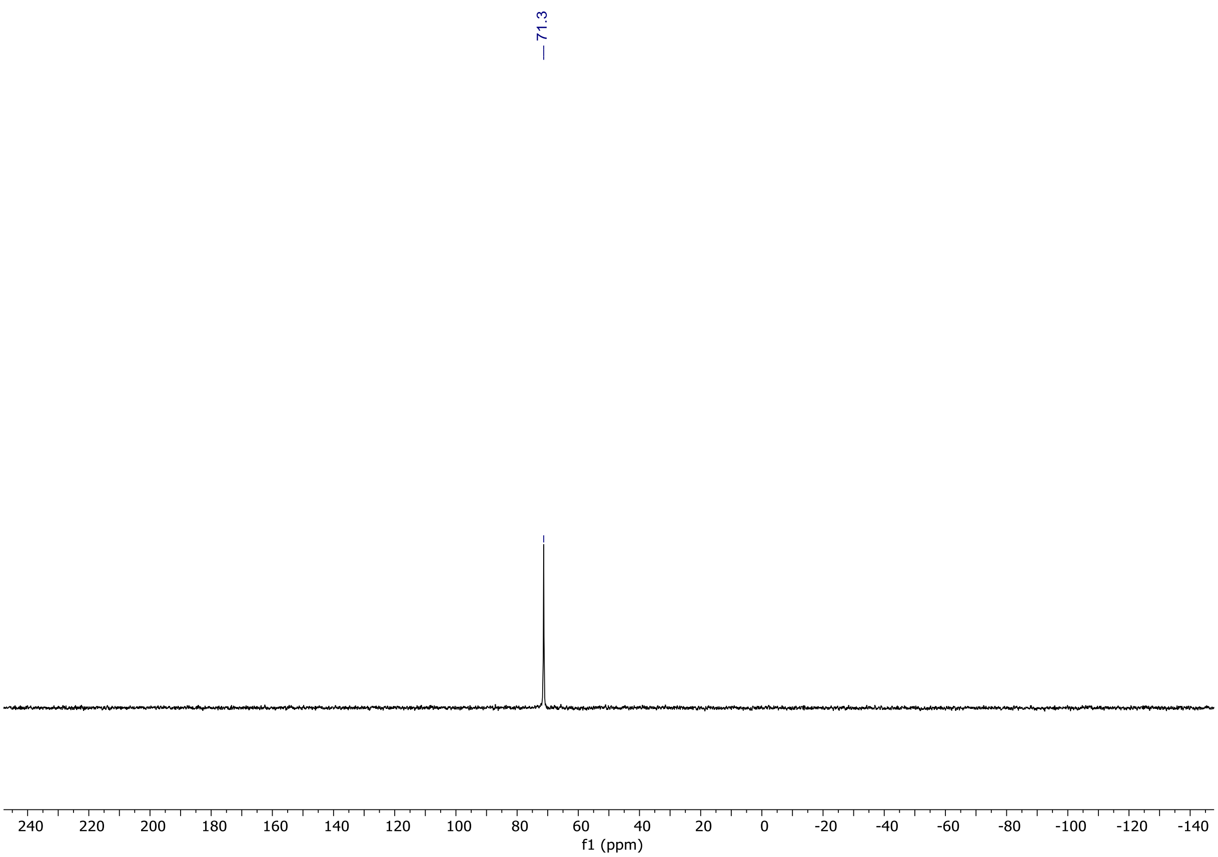

Figure S109. ^{31}P NMR (162 MHz, CD_2Cl_2) of *fac*-[Mn($P^{Cy}N^{Me}$)$(CO)_3CH_3$] (**PN1**)

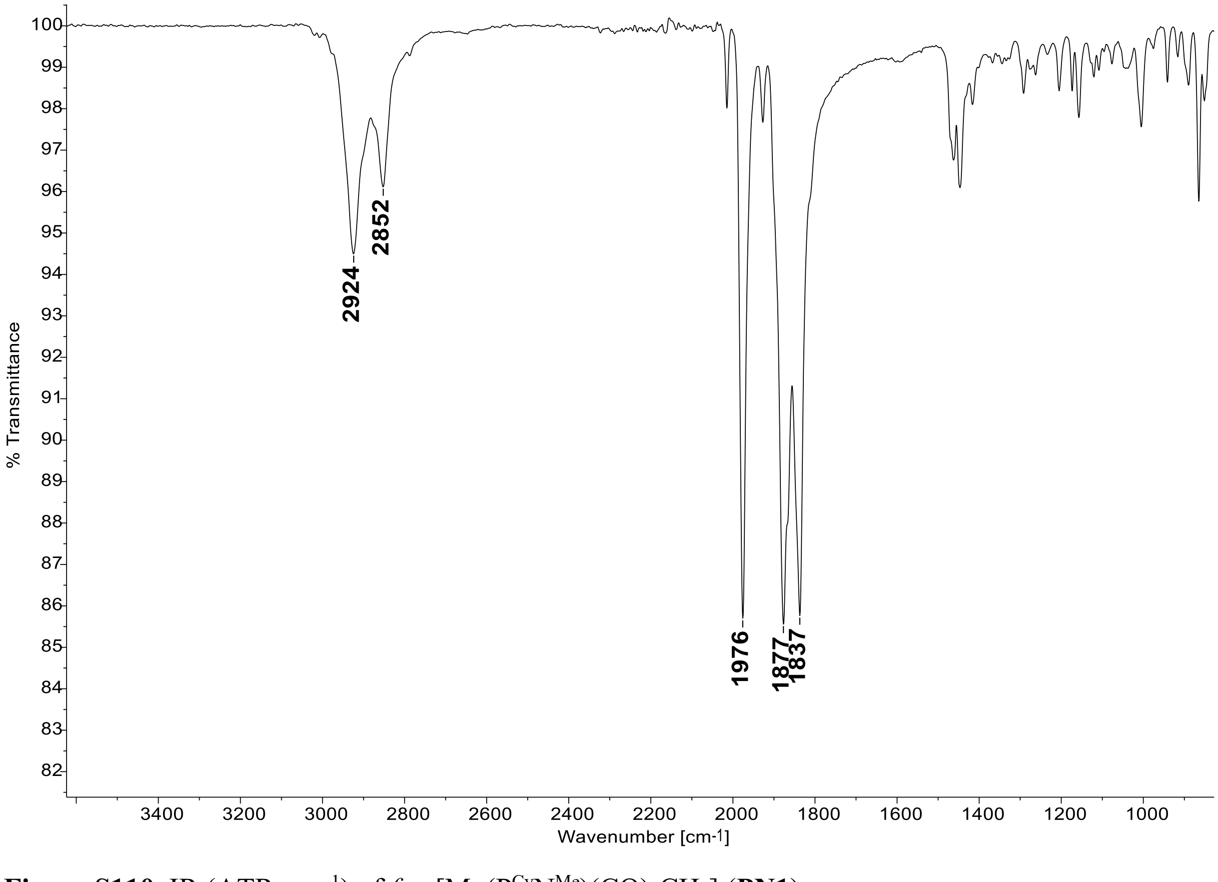

Figure S110. IR (ATR, cm^{-1}) of *fac*-[Mn($P^{Cy}N^{Me}$)$(CO)_3CH_3$] (**PN1**)

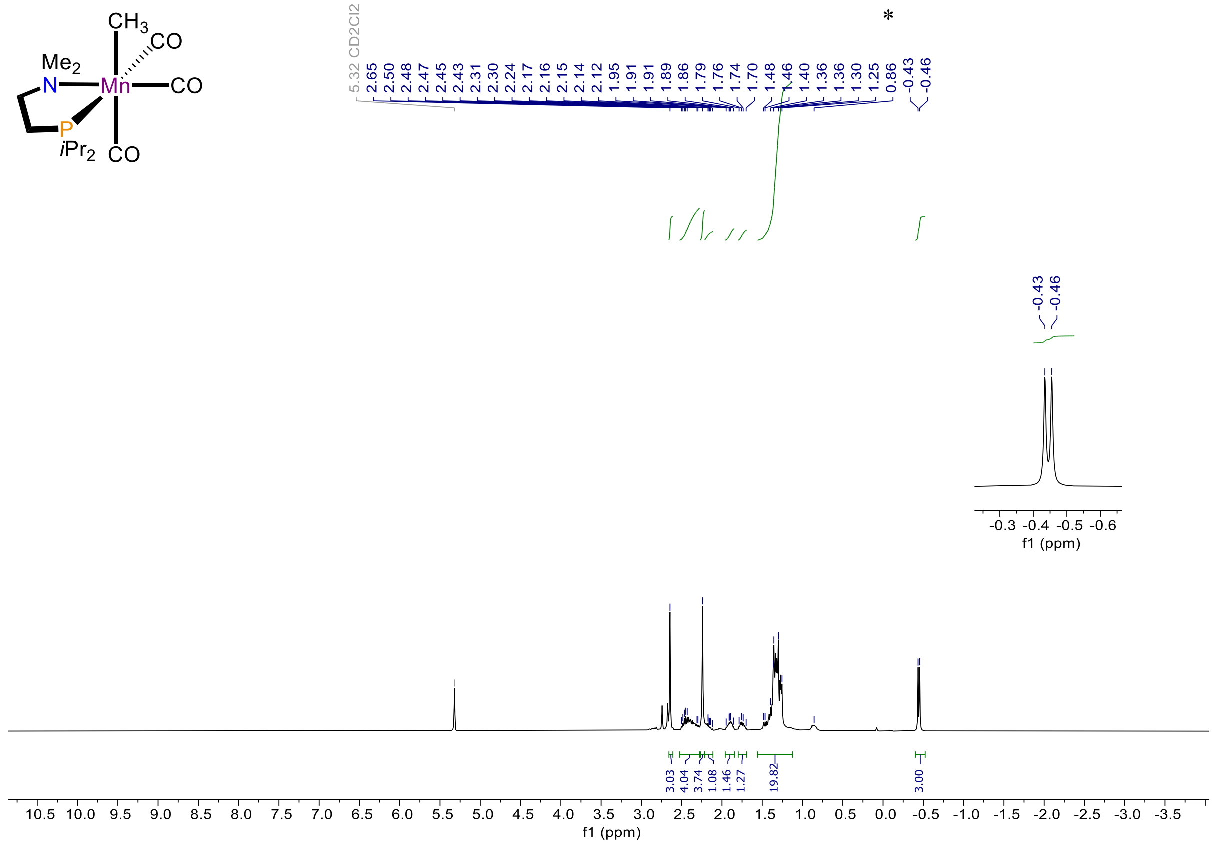

* = grease

Figure S111. ^{1}H NMR (400 MHz, CD_2Cl_2) of *fac*-[Mn($P^{iPr}N^{Me}$)$(CO)_3CH_3$] (**PN2**)

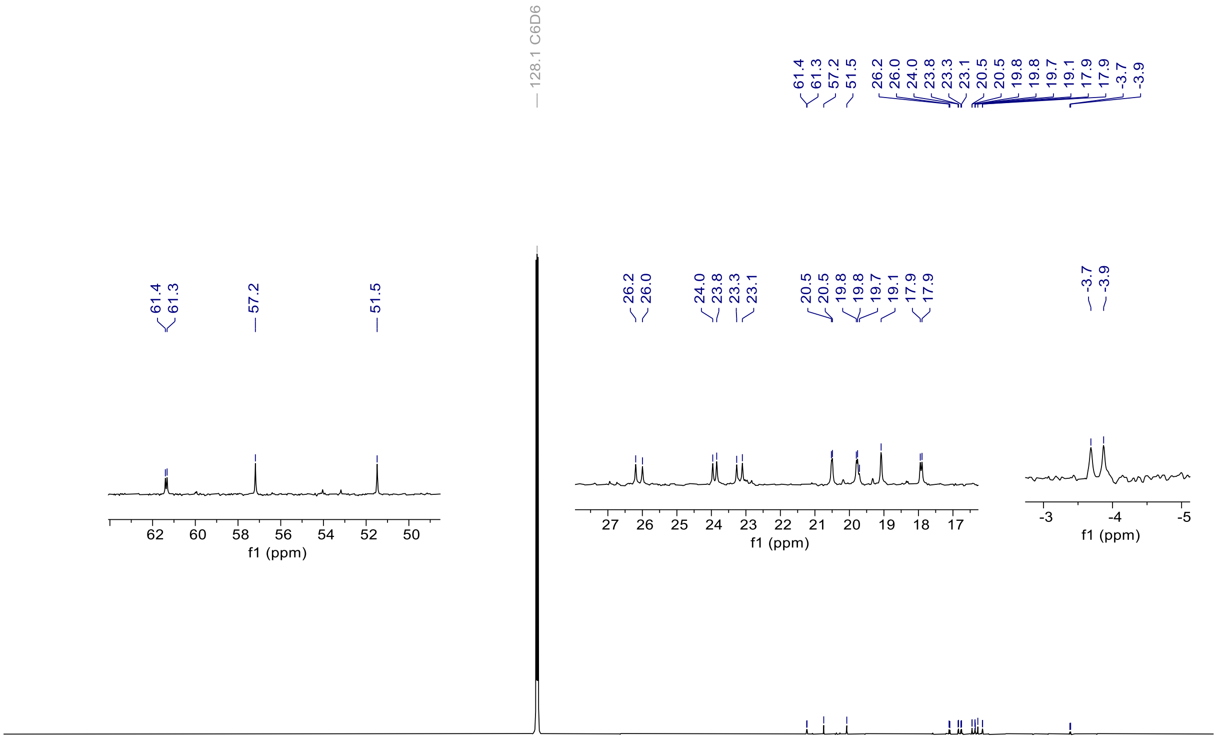

Figure S112. ^{13}C NMR (101 MHz, C_6D_6) of *fac*-[Mn($P^{iPr}N^{Me}$)$(CO)_3CH_3$] (**PN2**)

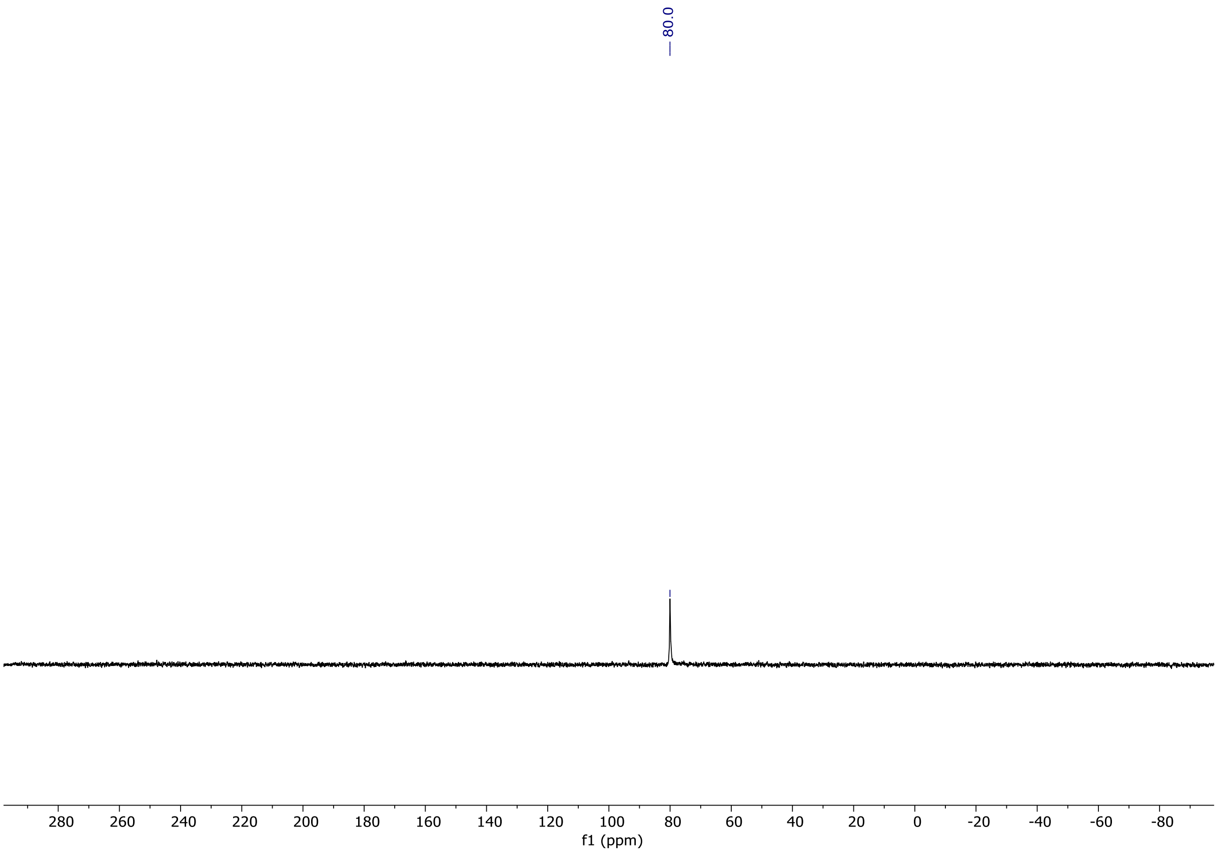

Figure S113. $^{31}P\{^{1}H\}$ NMR (162 MHz, CD_2Cl_2) of *fac*-[Mn($P^{iPr}N^{Me}$)$(CO)_3CH_3$] (**PN2**)

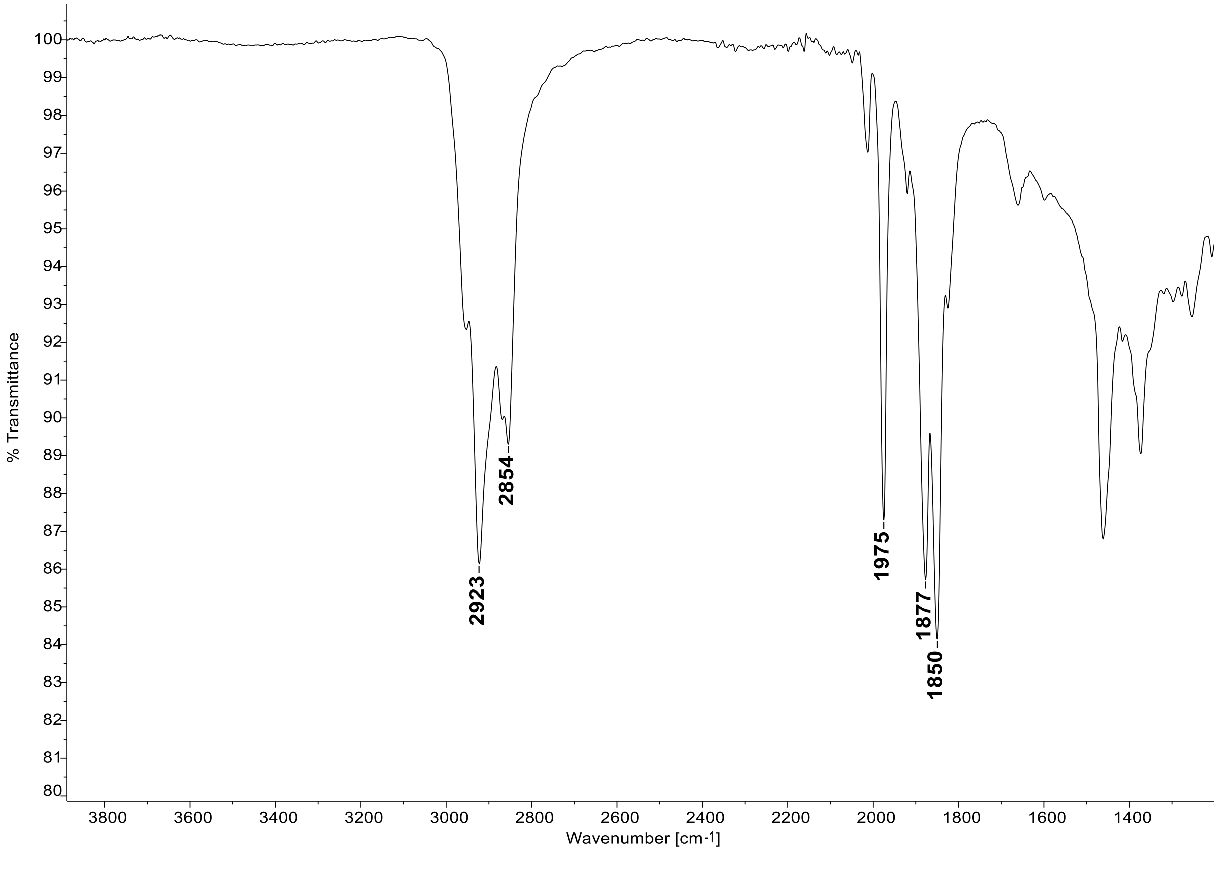

Figure S114. IR (ATR, cm^{-1}) of *fac*-[Mn($P^{iPr}N^{Me}$)$(CO)_3CH_3$] (**PN2**)

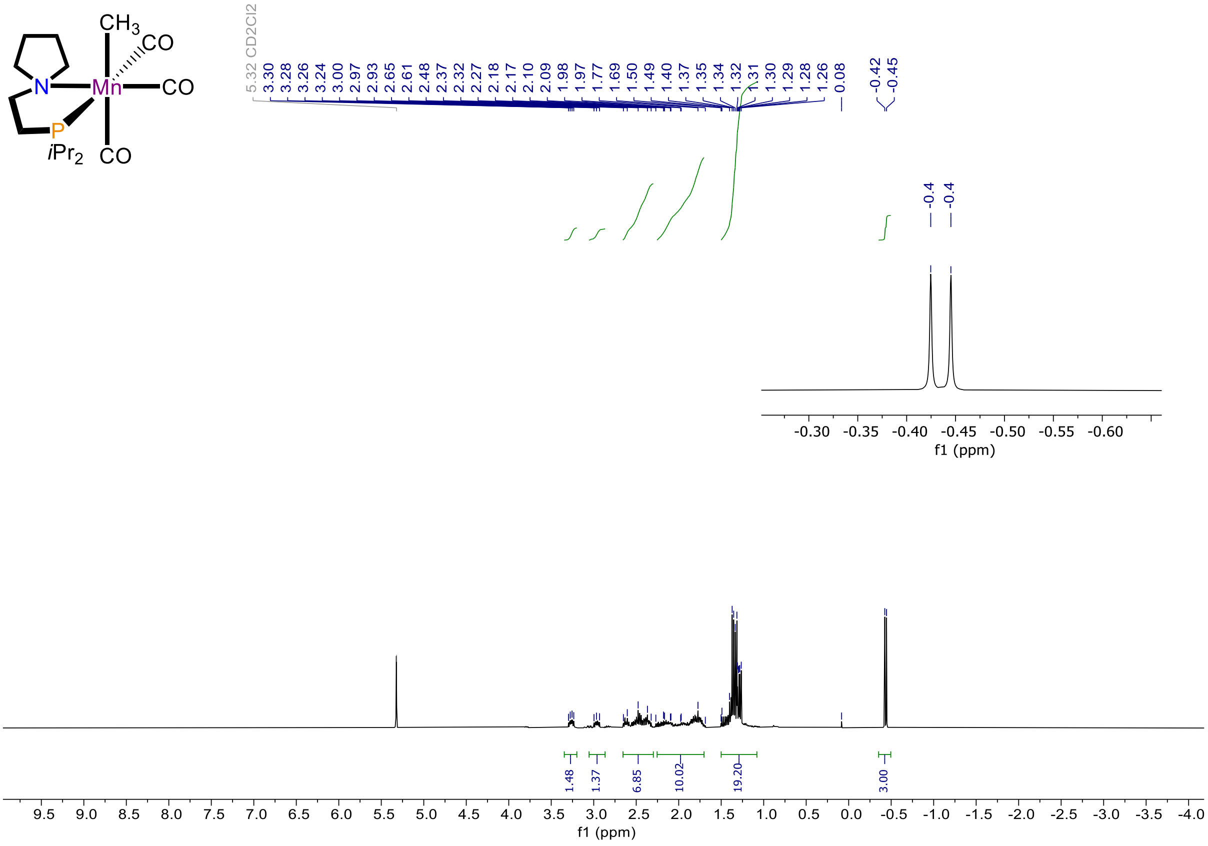

Figure S115. ^{1}H NMR (400 MHz, CD_2Cl_2) of *fac*-[Mn($P^{iPr}N^{Pyrr}$)(CO)$_3$CH$_3$] (**PN3**)

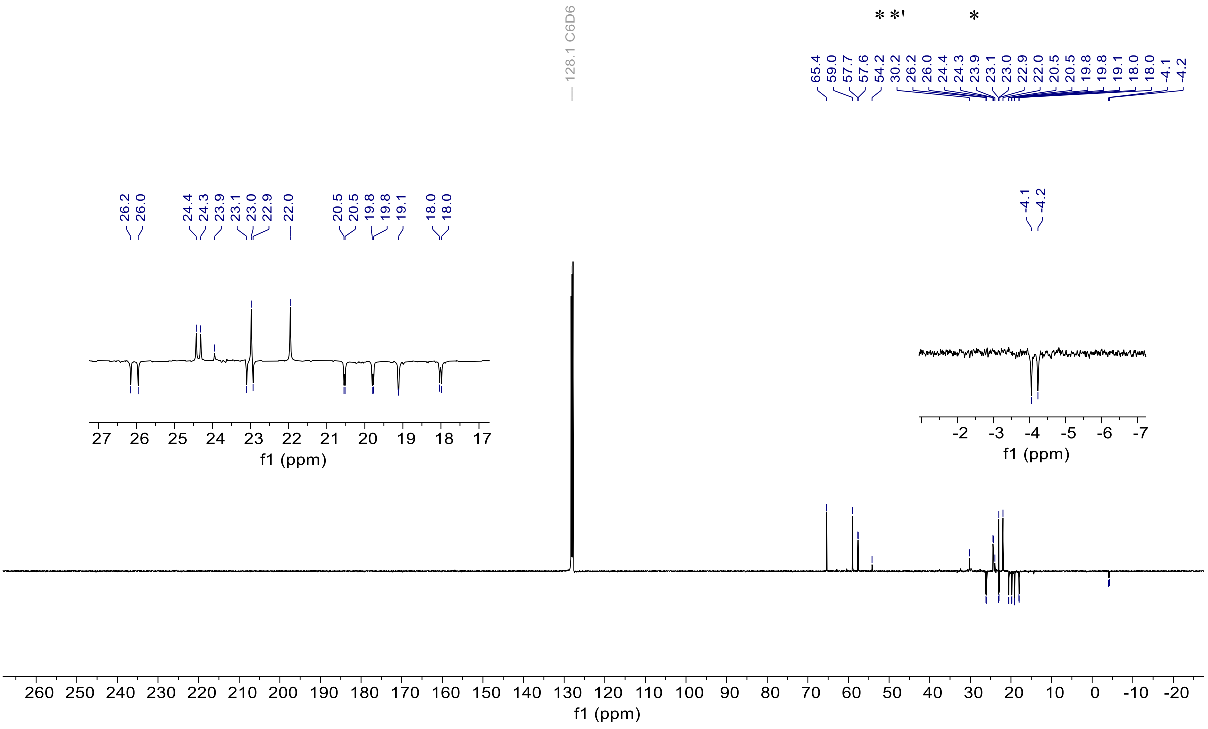

* = ligand, *' = grease

Figure S116. $^{13}C\{^{1}H\}$ NMR (101 MHz, C_6D_6) of *fac*-[Mn($P^{iPr}N^{Pyrr}$)(CO)$_3$CH$_3$] (**PN3**)

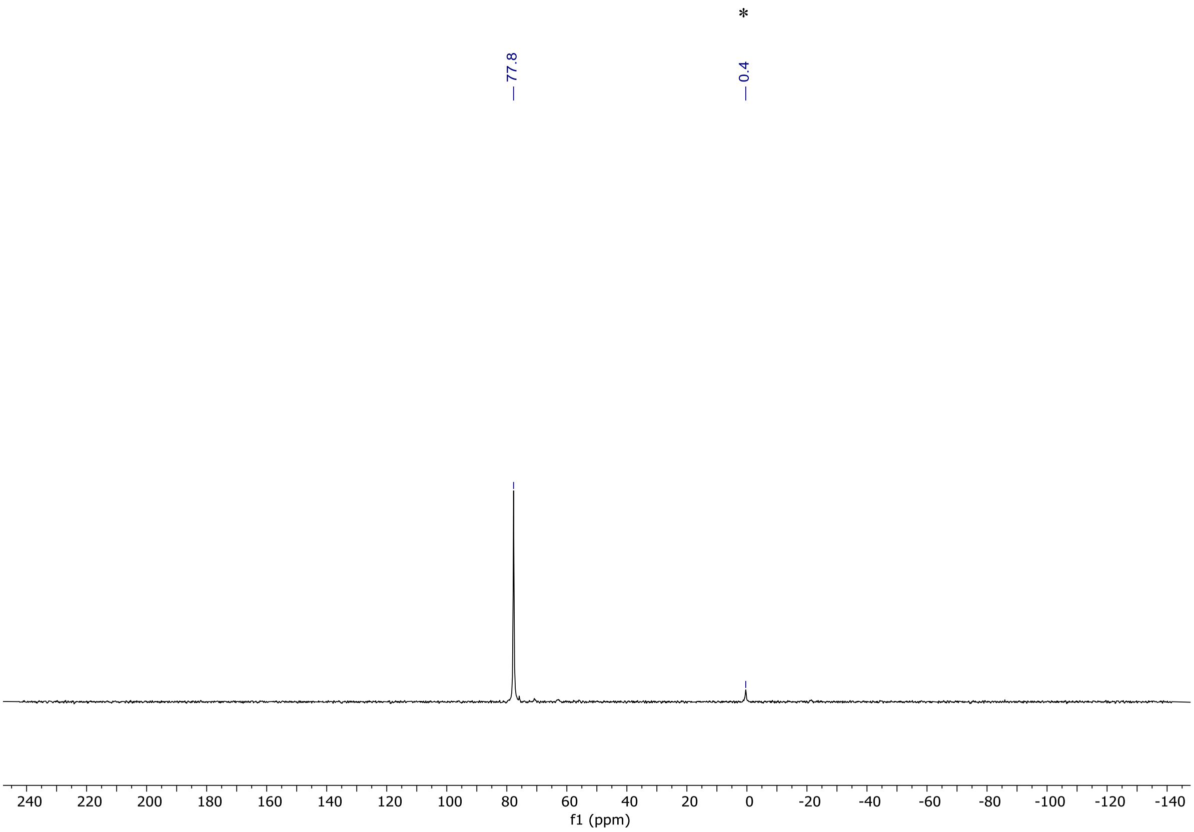

* = ligand

Figure S117. $^{31}P\{^{1}H\}$ NMR (162 MHz, C_6D_6) of *fac*-[Mn($P^{iPr}N^{Pyrr}$)$(CO)_3CH_3$] (**PN3**)

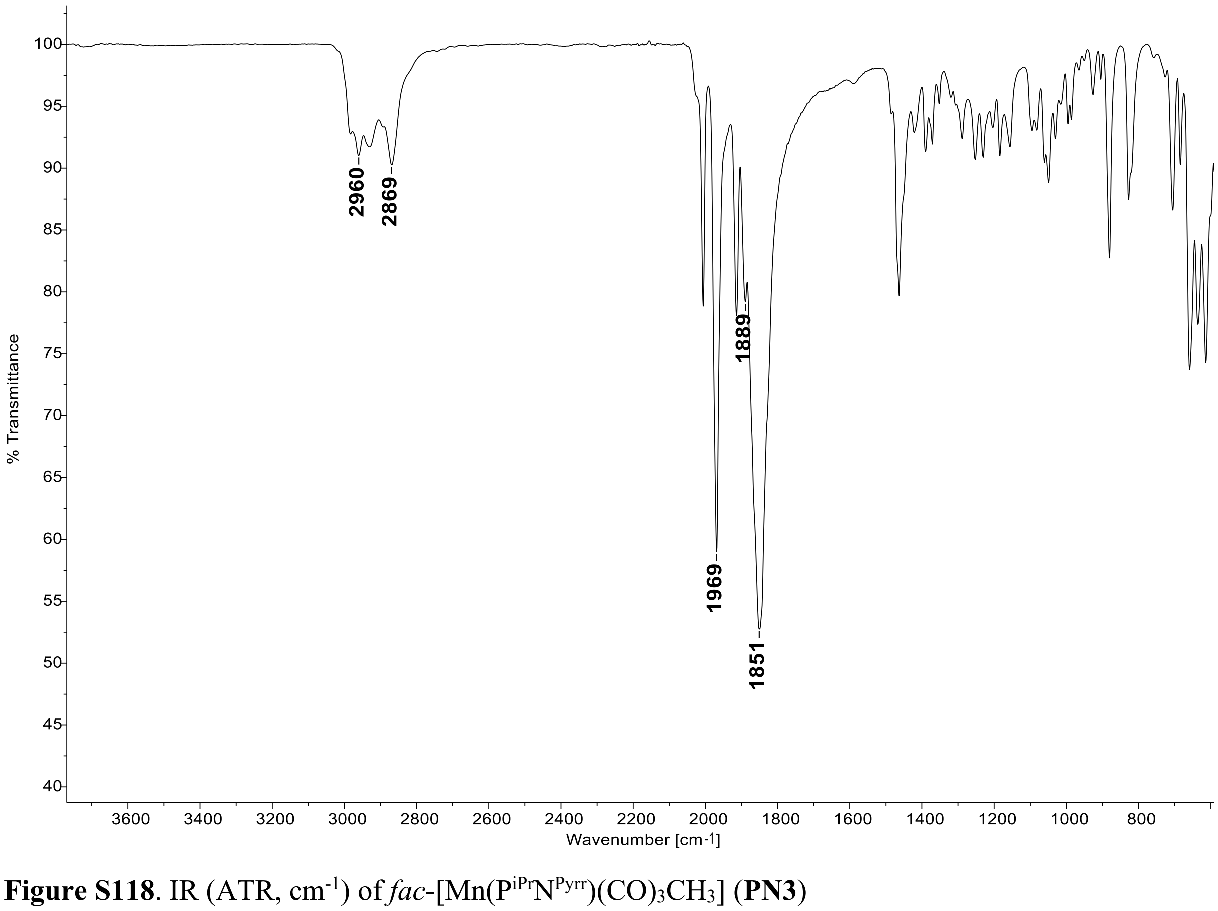

Figure S118. IR (ATR, cm^{-1}) of *fac*-[Mn($P^{iPr}N^{Pyrr}$)$(CO)_3CH_3$] (**PN3**)

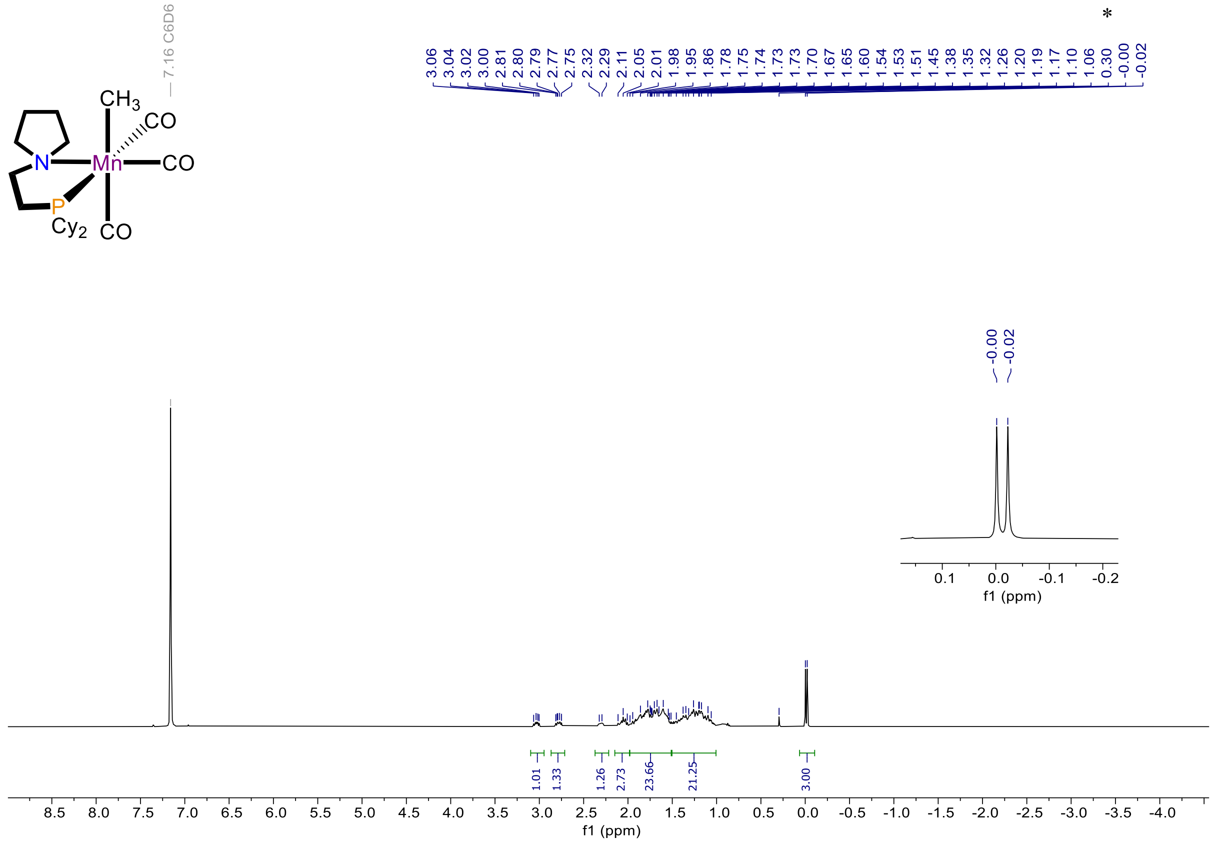

* = grease

Figure S119. ^{1}H NMR (400 MHz, C_6D_6) of *fac*-[Mn($P^{Cy}N^{Pyrr}$)$(CO)_3CH_3$] (**PN4**)

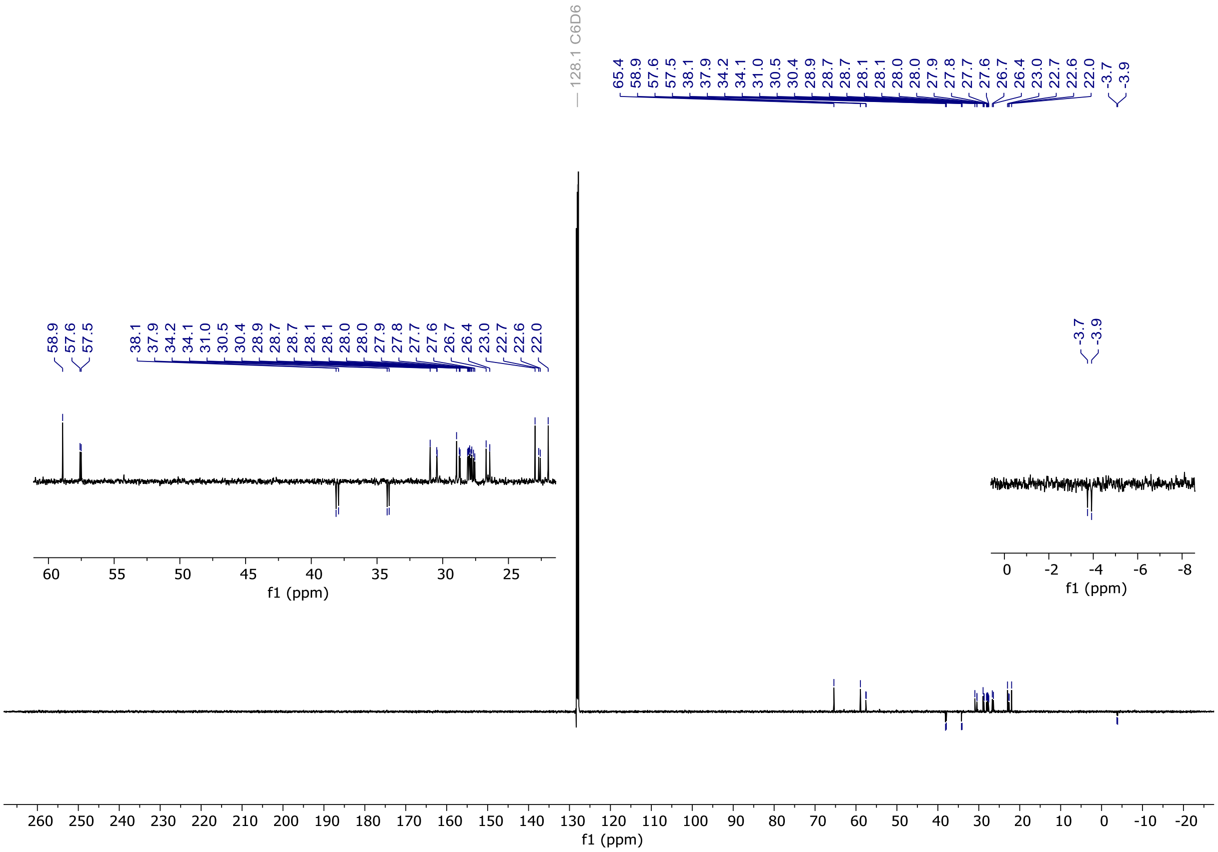

Figure S120. $^{13}C\{^{1}H\}$ NMR (101 MHz, C_6D_6) of *fac*-[Mn($P^{Cy}N^{Pyrr}$)$(CO)_3CH_3$] (**PN4**)

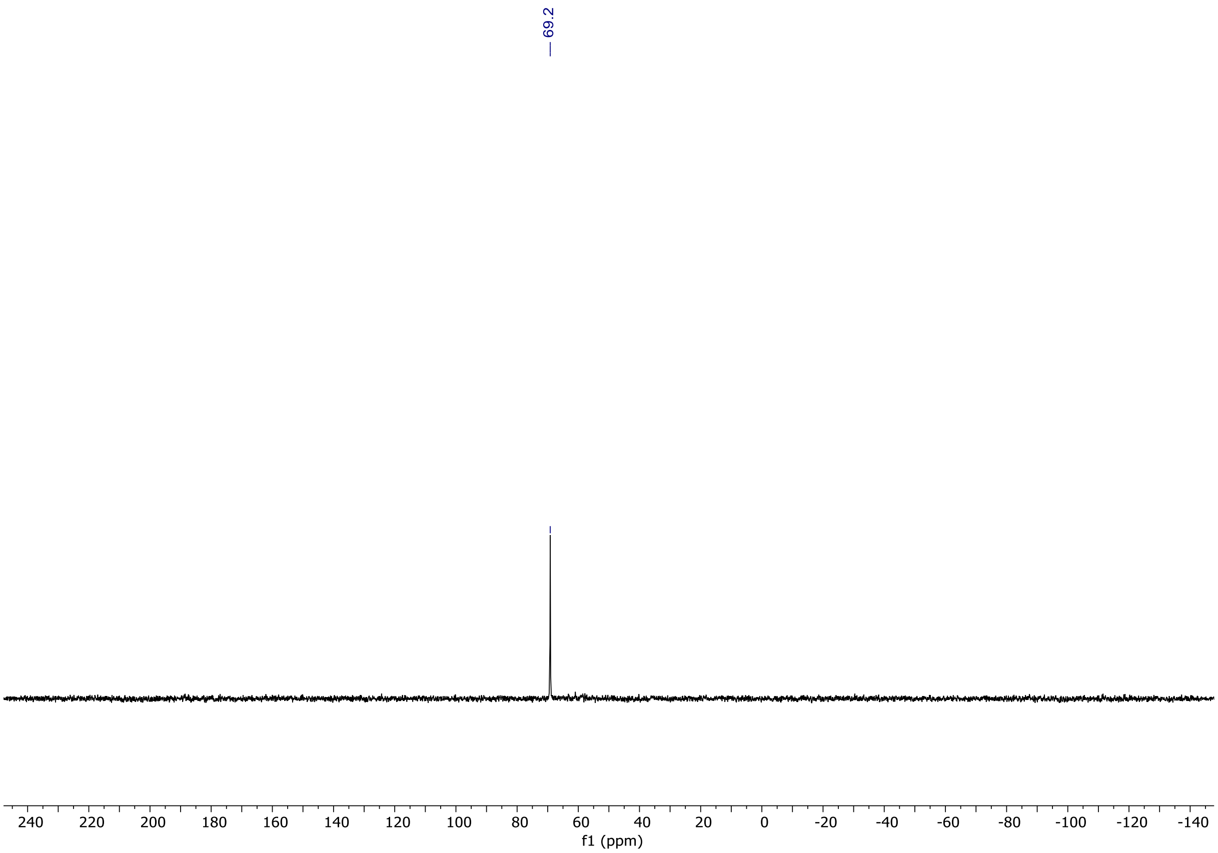

Figure S121. $^{31}P\{^{1}H\}$ NMR (162 MHz, C_6D_6) of *fac*-[Mn($P^{Cy}N^{Pyrr}$)$(CO)_3CH_3$] (**PN4**)

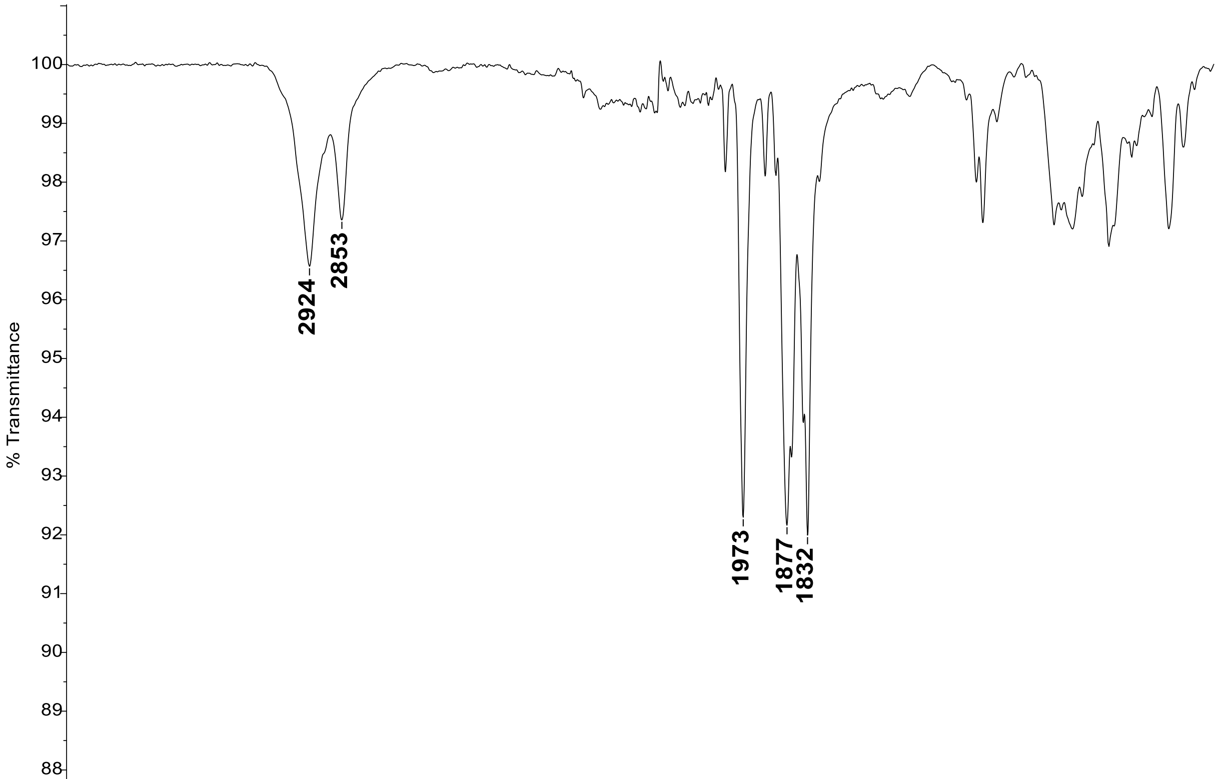

Figure S122. IR (ATR, cm^{-1}) of *fac*-[Mn($P^{Cy}N^{Pyrr}$)$(CO)_3CH_3$] (**PN4**)

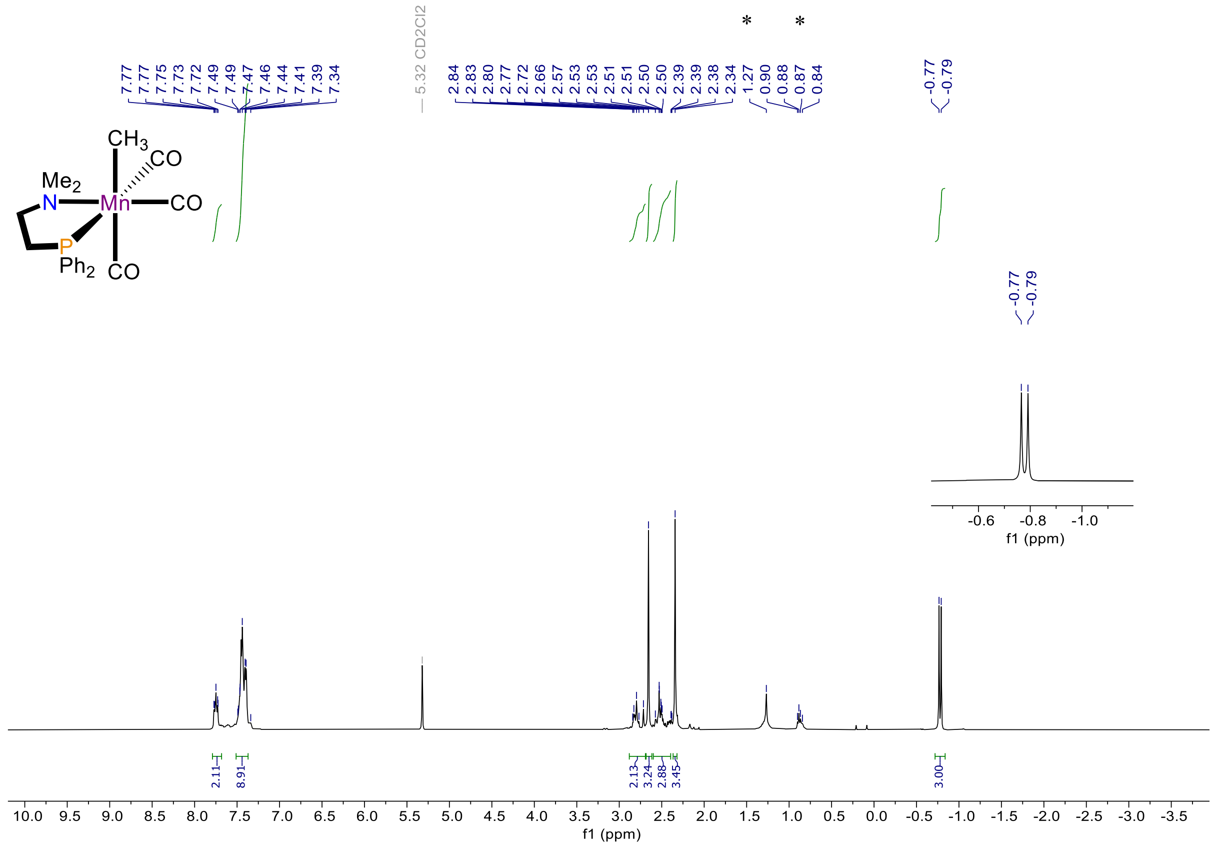

* = grease

Figure S123. ^{1}H NMR (400 MHz, CD_2Cl_2) of *fac*-[Mn($P^{Ph}N^{Me}$)$(CO)_3CH_3$] (**PN5**)

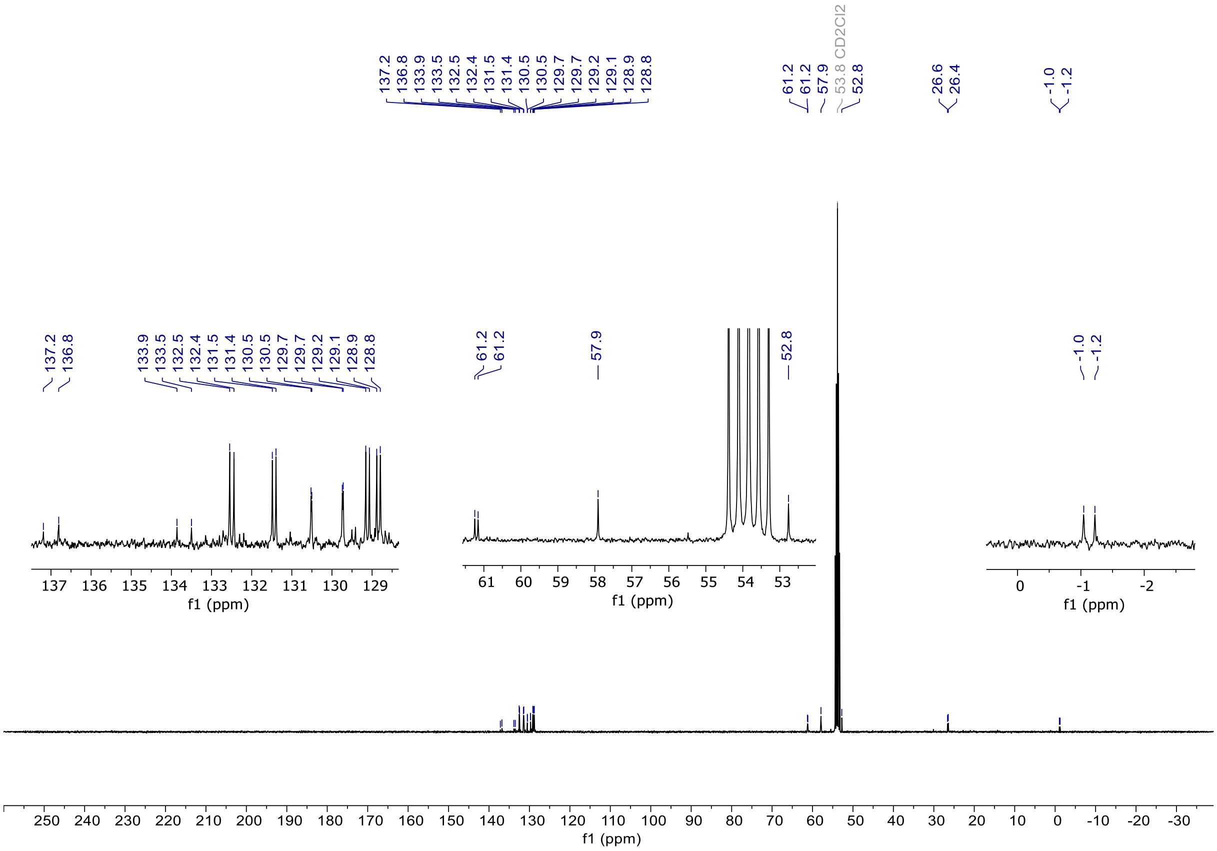

Figure S124. $^{13}C\{^{1}H\}$ NMR (101 MHz, CD_2Cl_2) of *fac*-[Mn($P^{Ph}N^{Me}$)$(CO)_3CH_3$] (**PN5**)

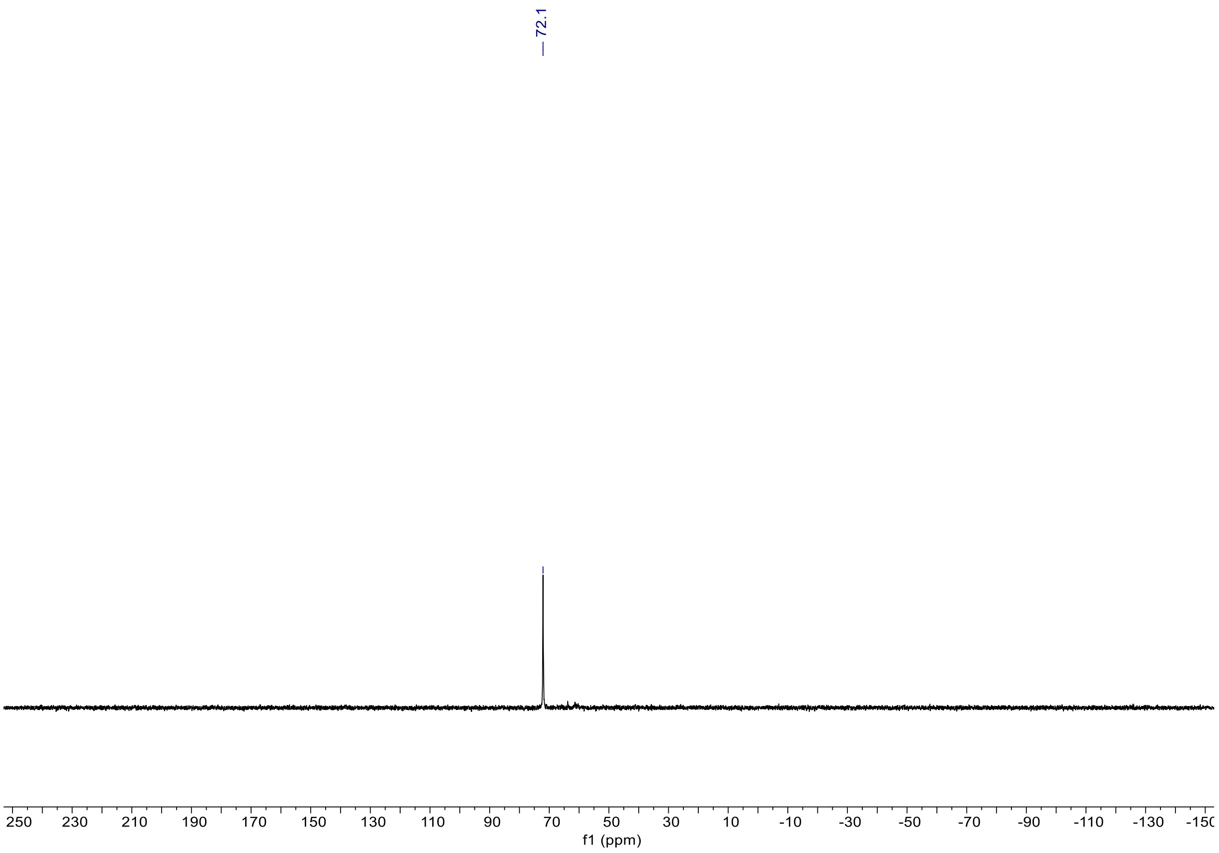

Figure S125. $^{31}P\{^{1}H\}$ NMR (162 MHz, CD_2Cl_2) of *fac*-[Mn($P^{Ph}N^{Me}$)$(CO)_3CH_3$] (**PN5**)

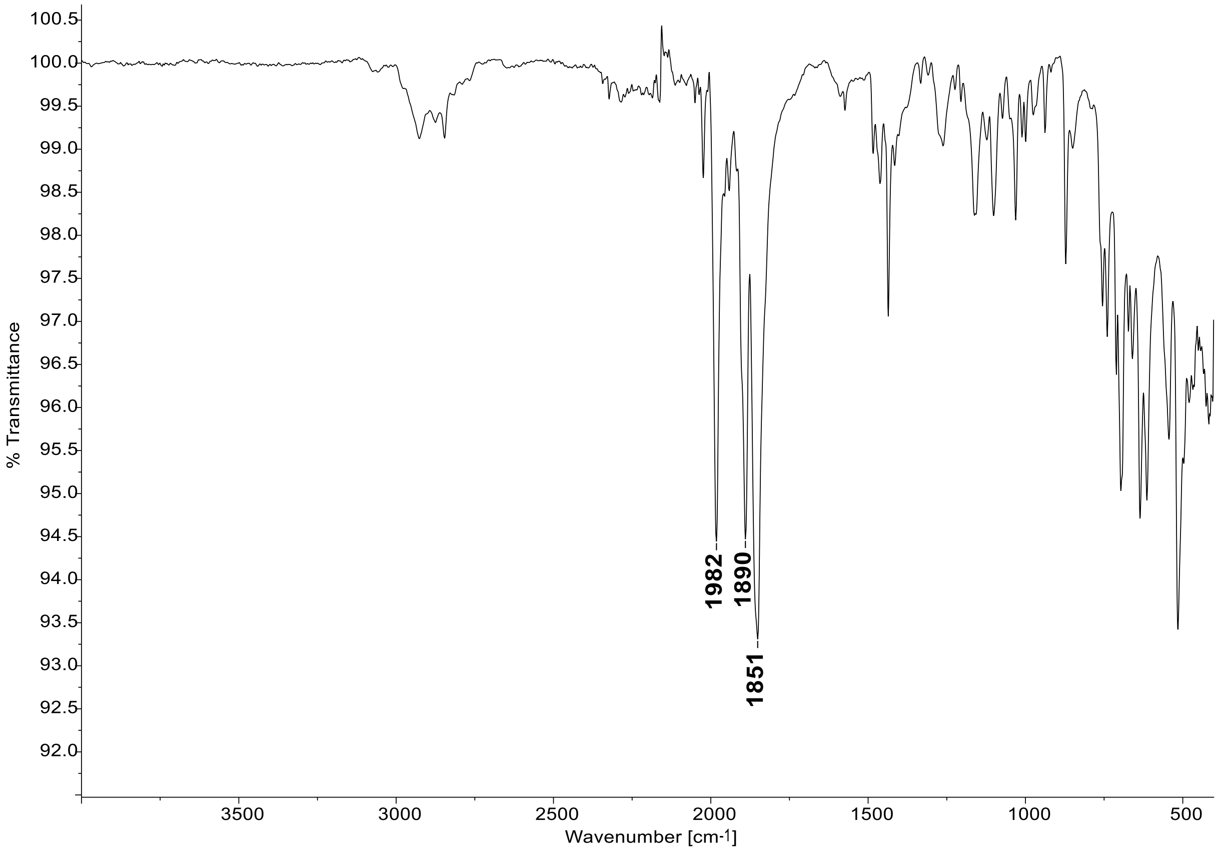

Figure S126. IR (ATR, cm^{-1}) of *fac*-[Mn($P^{Ph}N^{Me}$)$(CO)_3CH_3$] (**PN5**)

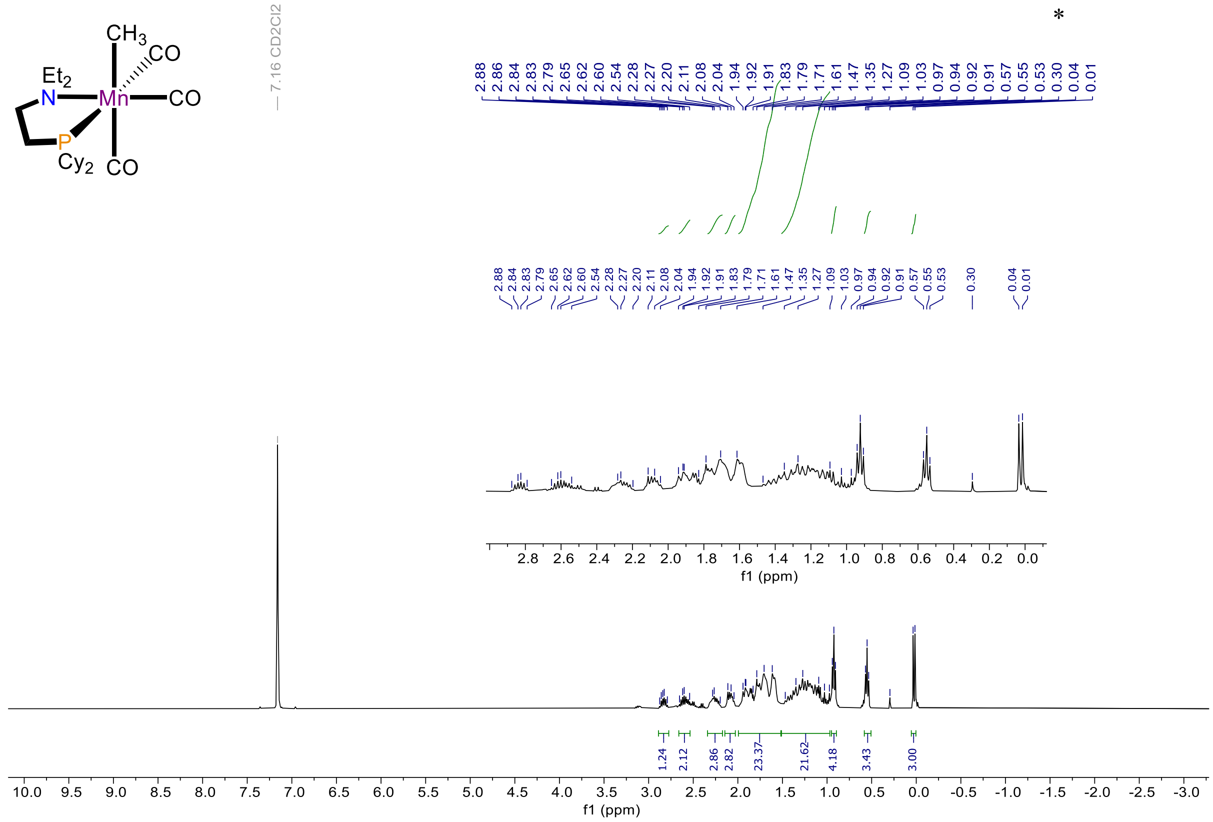

* = grease

Figure S127. ^{1}H NMR (400 MHz, C_6D_6) of *fac*-[Mn(P^{Cy}N^{Et})(CO)$_3$CH$_3$] (**PN6**)

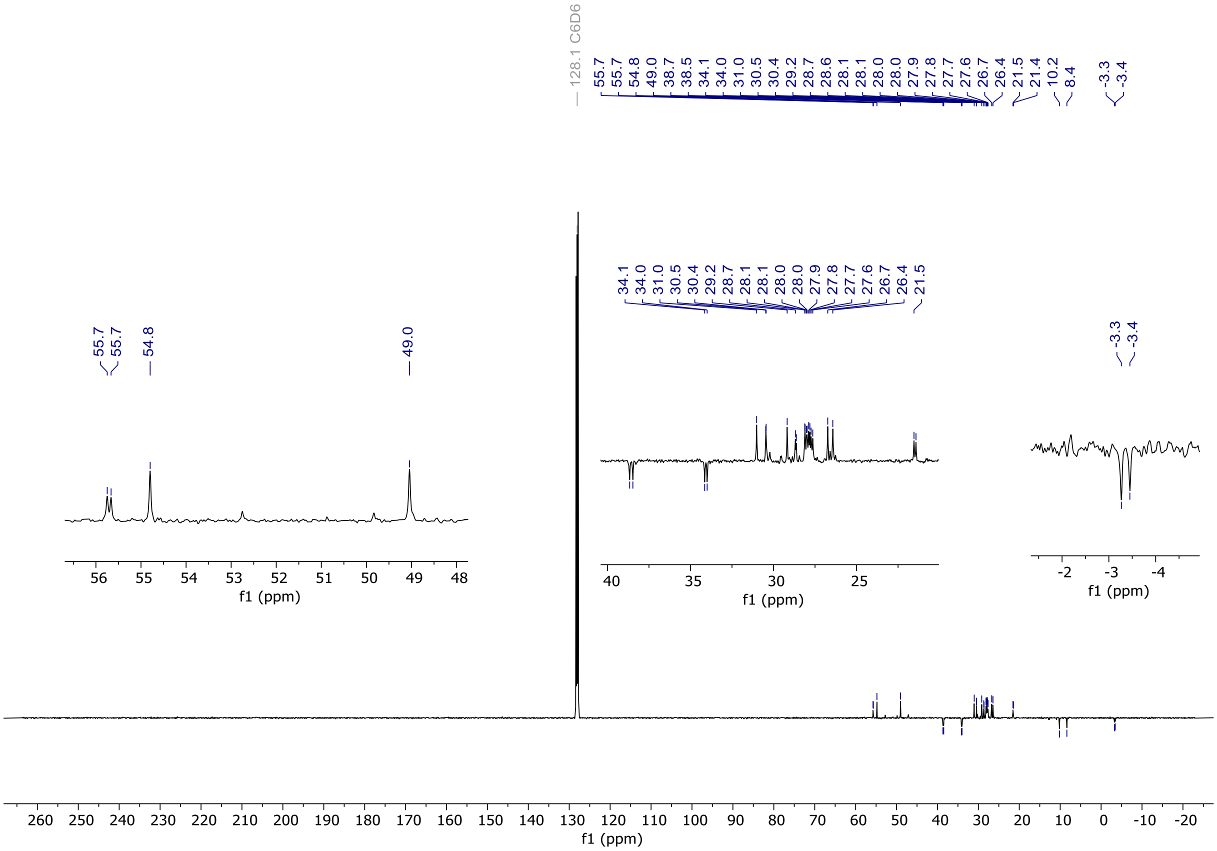

Figure S128. ^{13}C{^{1}H} NMR (101 MHz, C_6D_6) of *fac*-[Mn(P^{Cy}N^{Et})(CO)$_3$CH$_3$] (**PN6**)

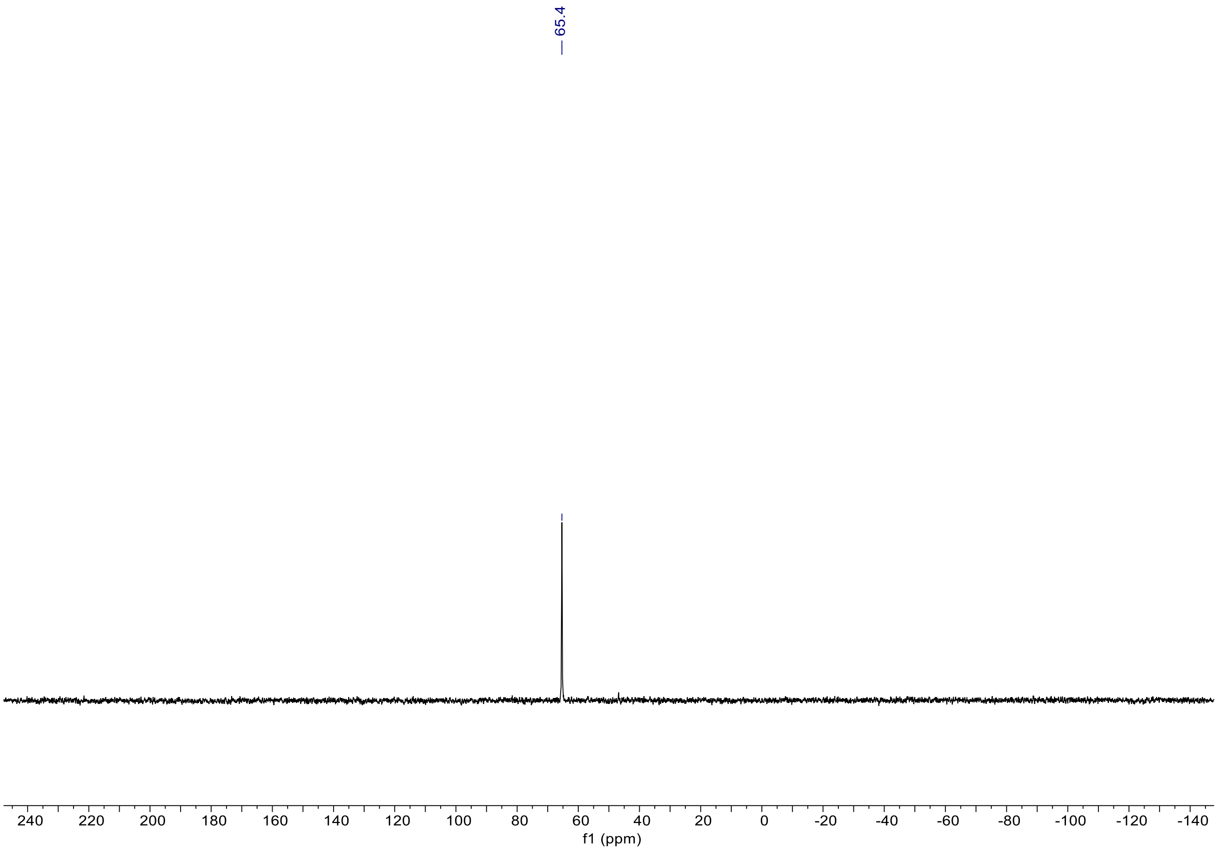

Figure S129. $^{31}P\{^{1}H\}$ NMR (162 MHz, C_6D_6) of *fac*-[Mn($P^{Cy}N^{Et}$)(CO)$_3$CH$_3$] (**PN6**)

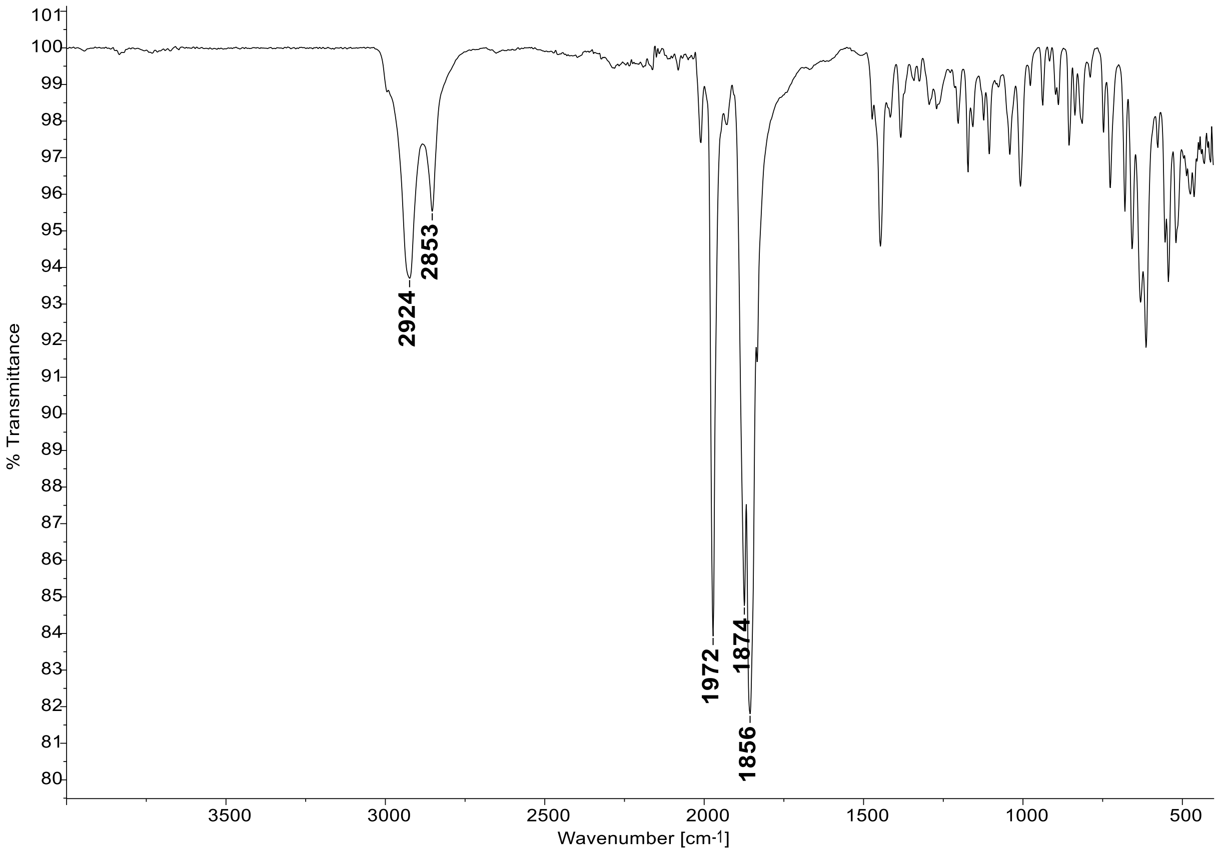

Figure S130. IR (ATR, cm^{-1}) of *fac*-[Mn($P^{Cy}N^{Et}$)(CO)$_3$CH$_3$] (**PN6**)

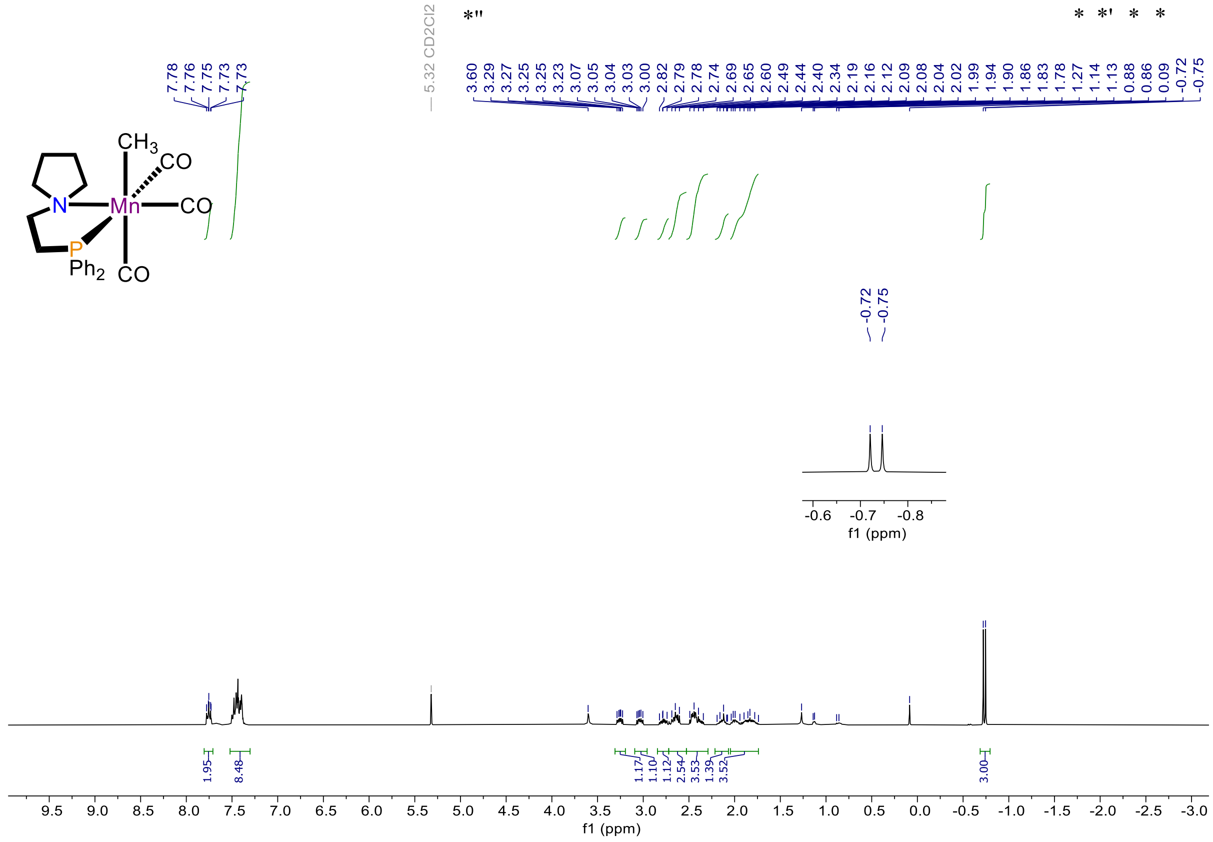

* = grease, *’ = diethyl ether, *’’ = diglyme

Figure S131. ^{1}H NMR (400 MHz, CD_2Cl_2) of *fac*-[Mn($P^{Ph}N^{Pyrr}$)$(CO)_3CH_3$] (**PN7**)

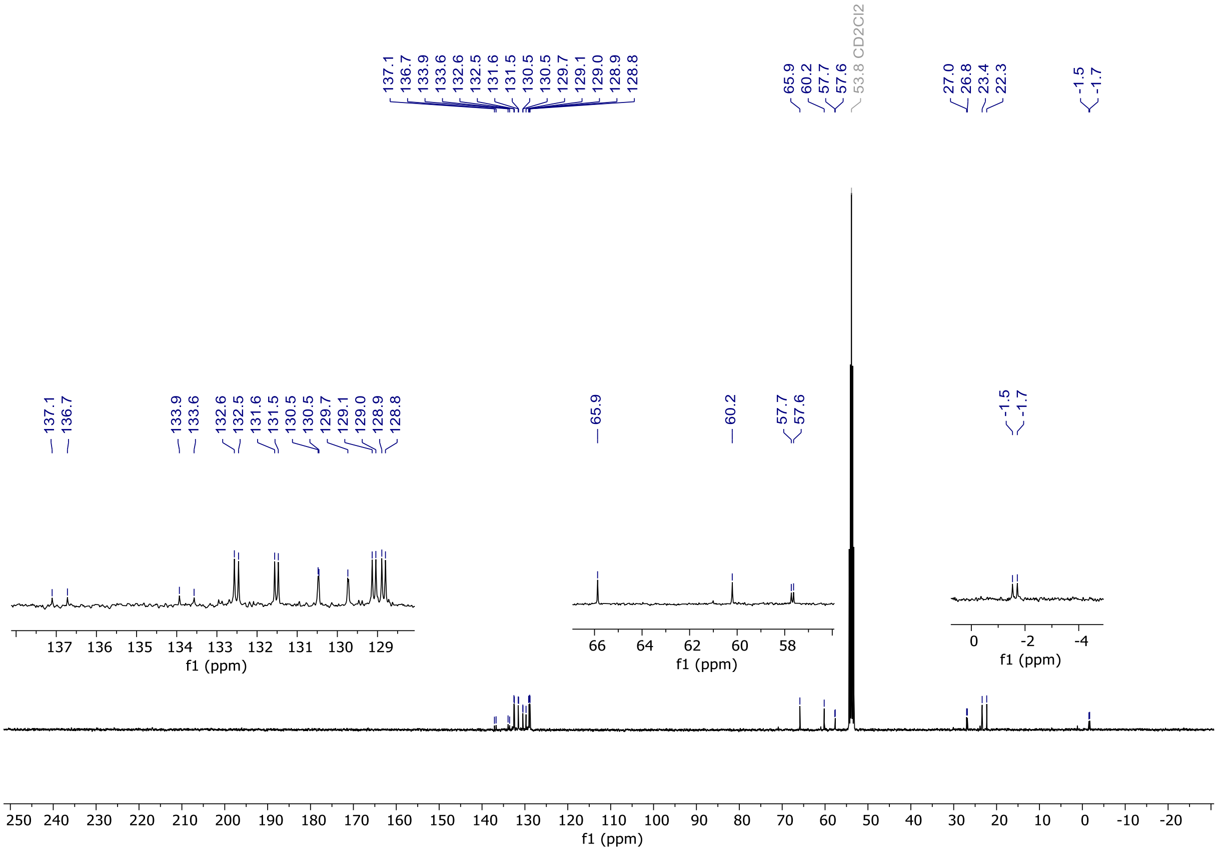

Figure S132. $^{13}C\{^{1}H\}$ NMR (101 MHz, CD_2Cl_2) of *fac*-[Mn($P^{Ph}N^{Pyrr}$)$(CO)_3CH_3$] (**PN7**)

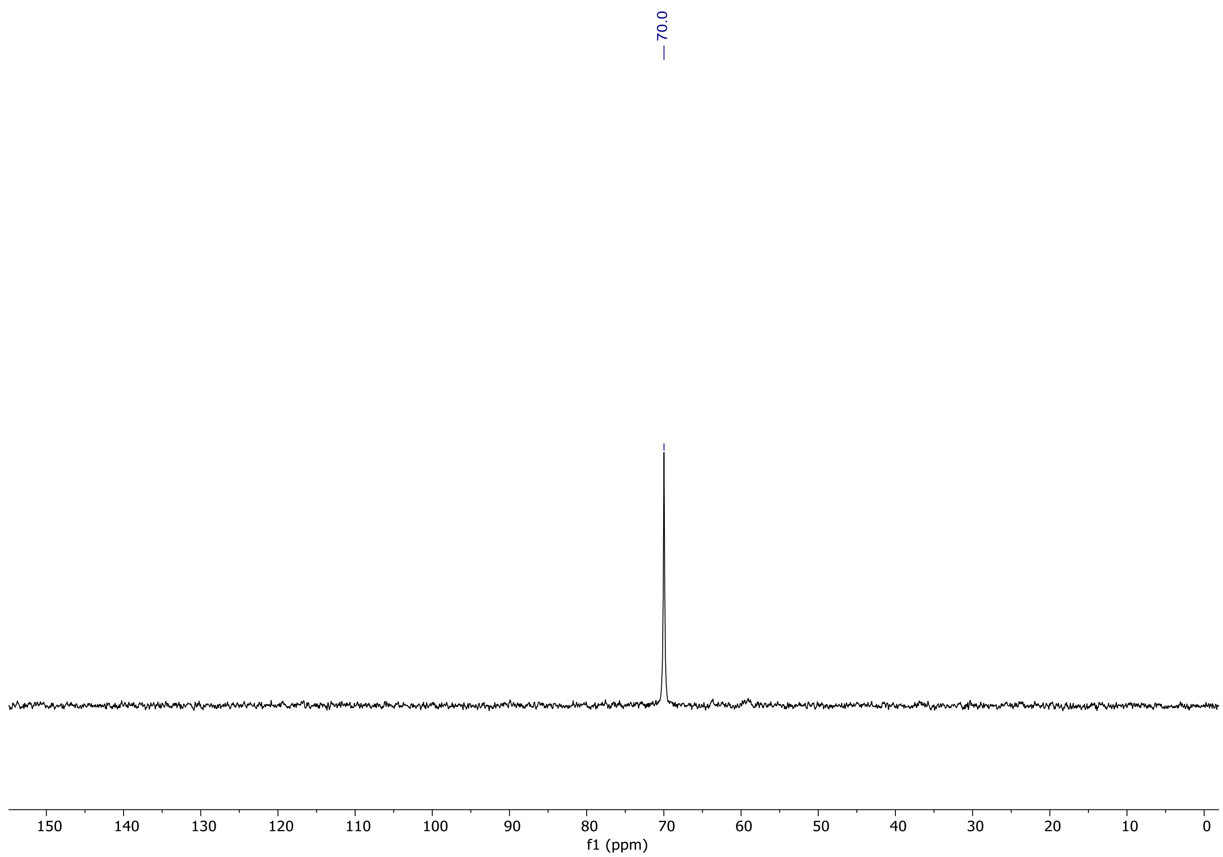

Figure S133. $^{31}P\{^1H\}$ NMR (162 MHz, CD_2Cl_2) of *fac*-[Mn($P^{Ph}N^{Pyrr}$)$(CO)_3CH_3$] (**PN7**)

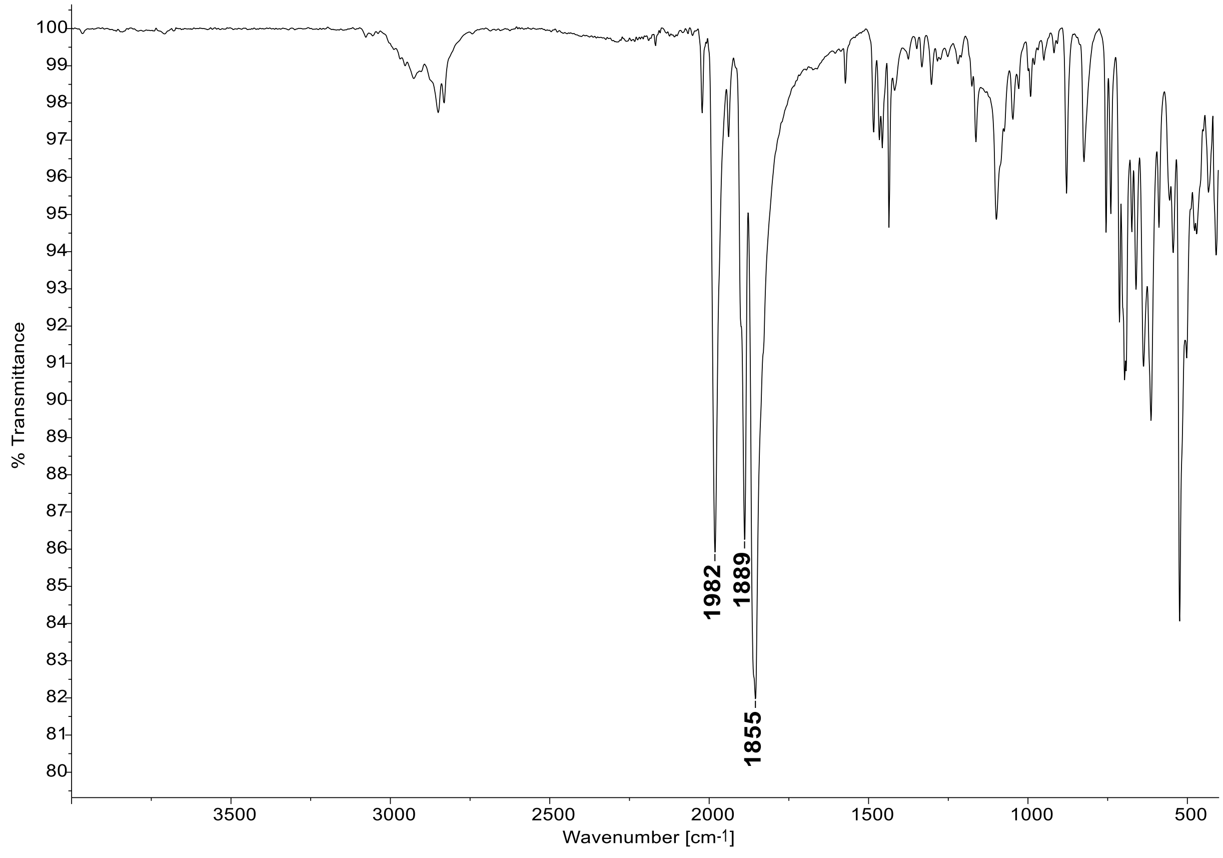

Figure S134. IR (ATR, cm^{-1}) of *fac*-[Mn($P^{Ph}N^{Pyrr}$)$(CO)_3CH_3$] (**PN7**)

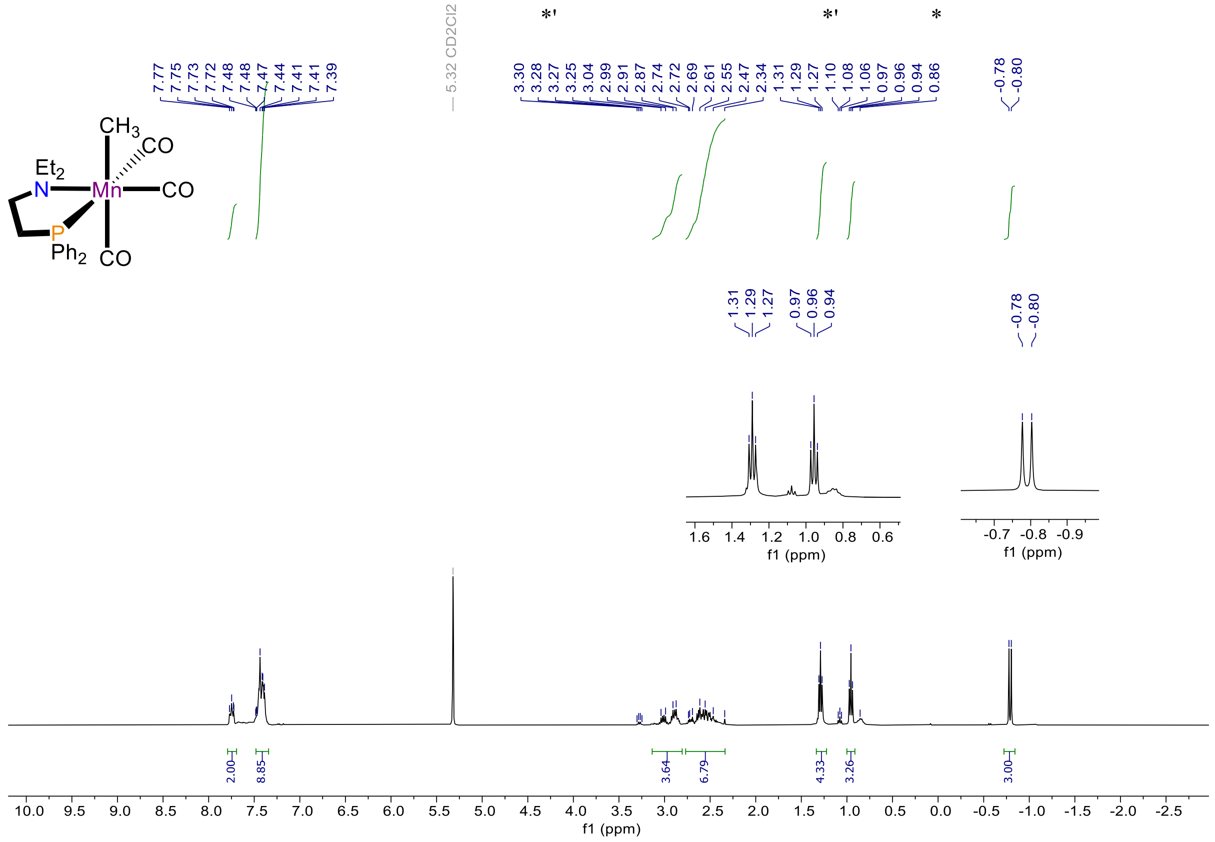

* = grease, *' = diethyl ether

Figure S135. ^{1}H NMR (400 MHz, CD_2Cl_2) of *fac*-[Mn(P^{Ph}N^{Et})(CO)$_3$CH$_3$] (**PN8**)

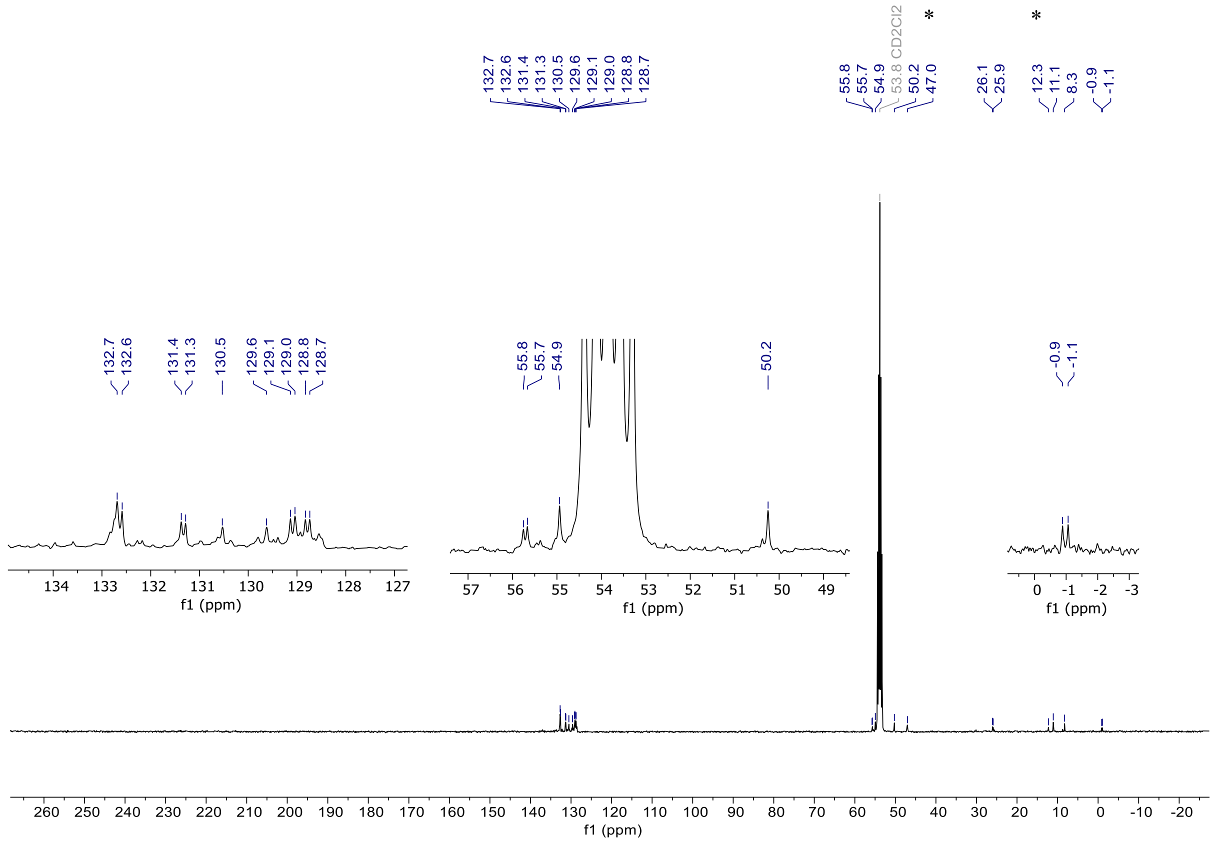

* = ligand

Figure S136. ^{13}C{^{1}H} NMR (101 MHz, CD_2Cl_2) of *fac*-[Mn(P^{Ph}N^{Et})(CO)$_3$CH$_3$] (**PN8**)

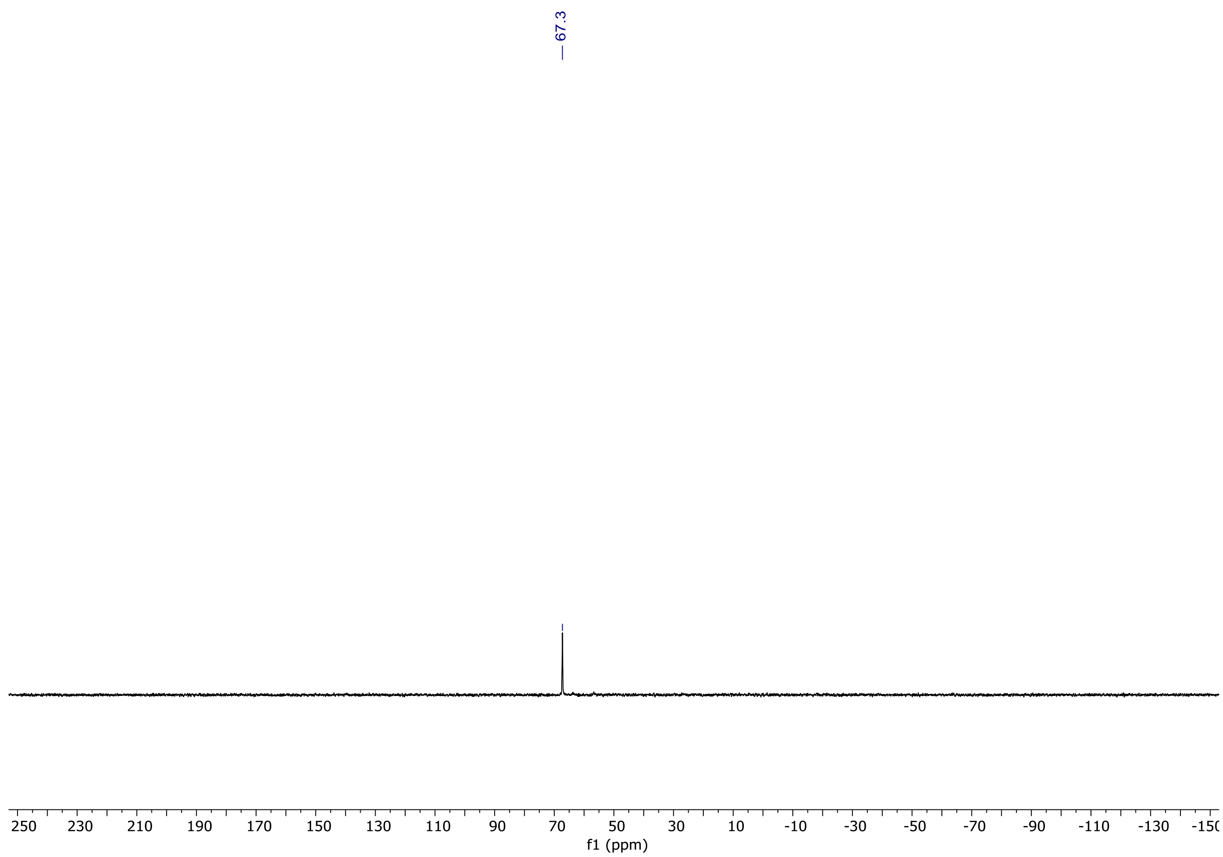

Figure S137. $^{31}P\{^{1}H\}$ NMR (101 MHz, CD_2Cl_2) of *fac*-[Mn($P^{Ph}N^{Et}$)$(CO)_3CH_3$] (**PN8**)

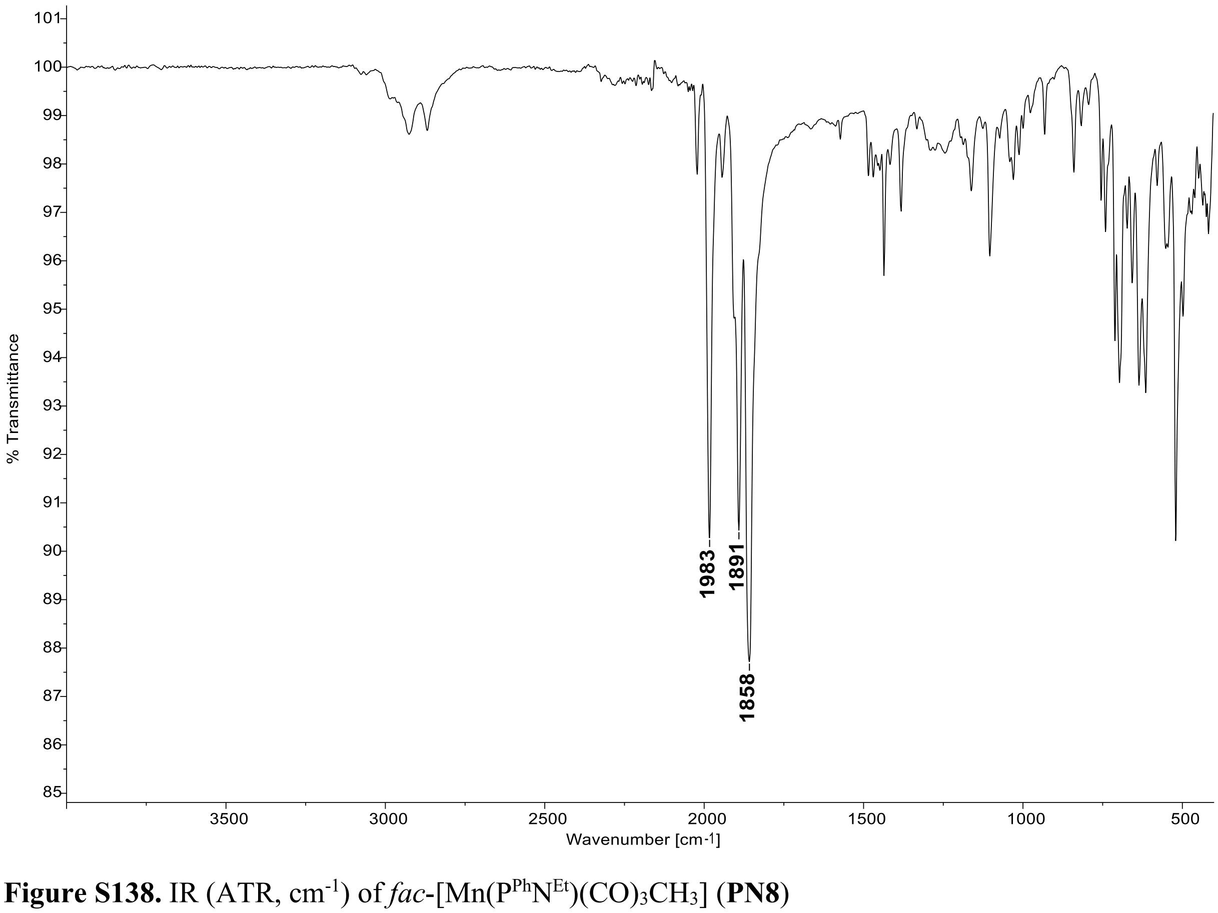

Figure S138. IR (ATR, cm^{-1}) of *fac*-[Mn($P^{Ph}N^{Et}$)$(CO)_3CH_3$] (**PN8**)

10.2 PP-based Mn(I) Carbonyl Complexes

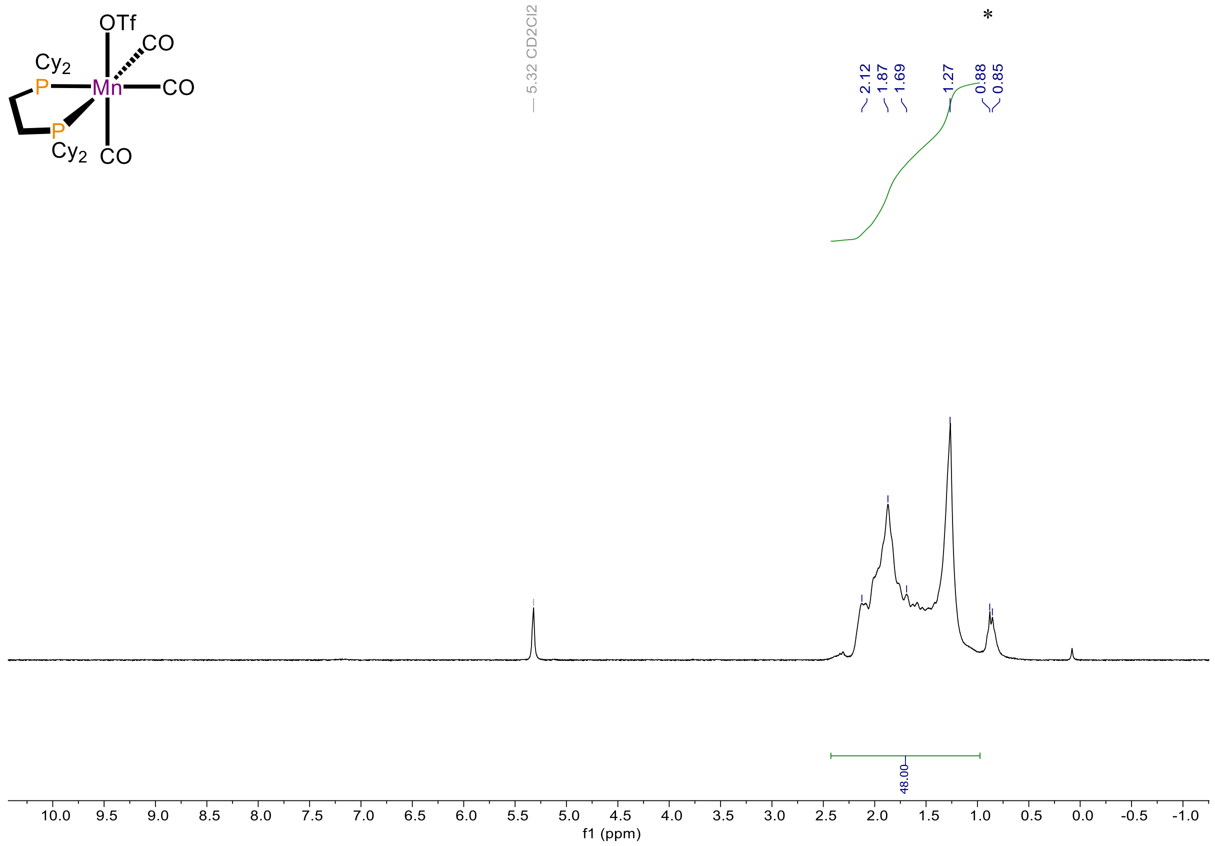

* = grease

Figure S139. ^{1}H NMR (250 MHz, CD_2Cl_2) of *fac*-[Mn($P^{Cy}P^{Cy}$)$(CO)_3$OTf] (**2j**)

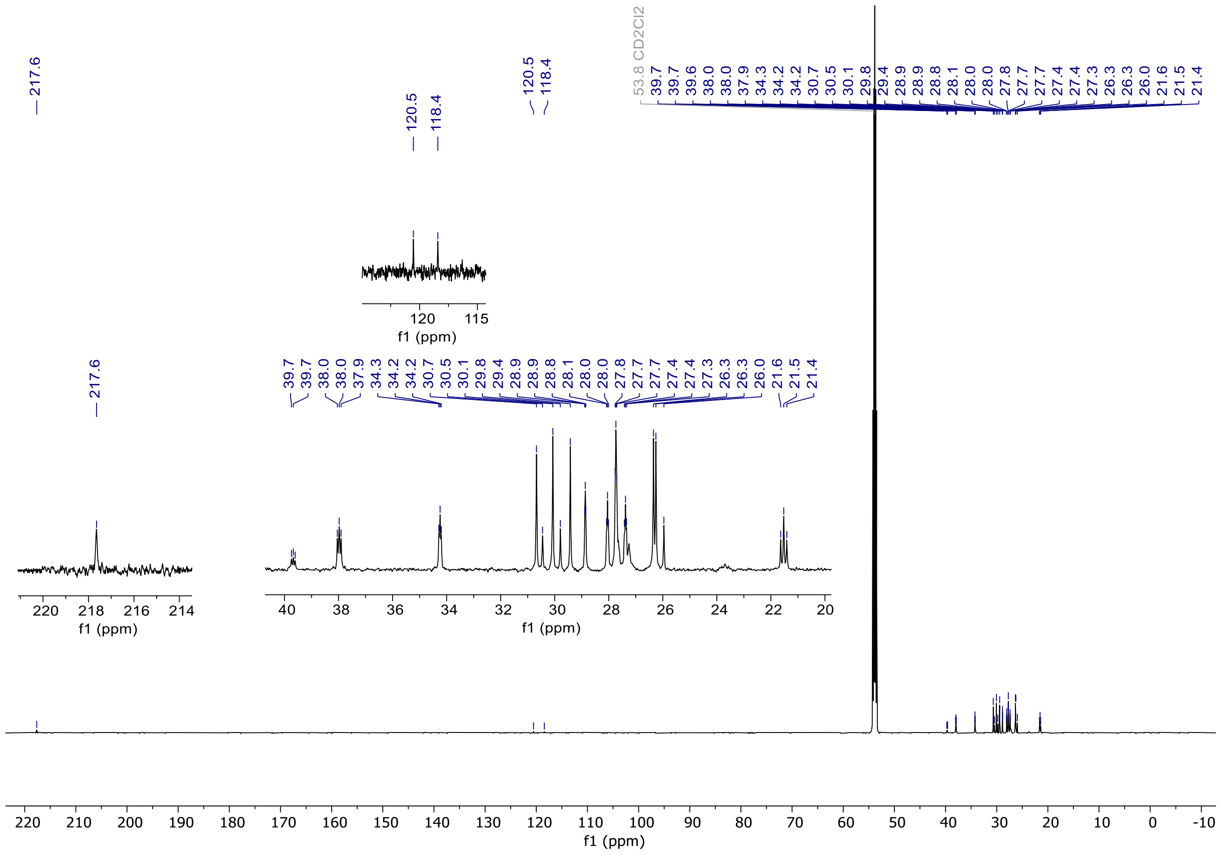

Figure S140. ^{13}C NMR (151 MHz, CD_2Cl_2) of *fac*-[Mn($P^{Cy}P^{Cy}$)$(CO)_3$OTf] (**2j**)

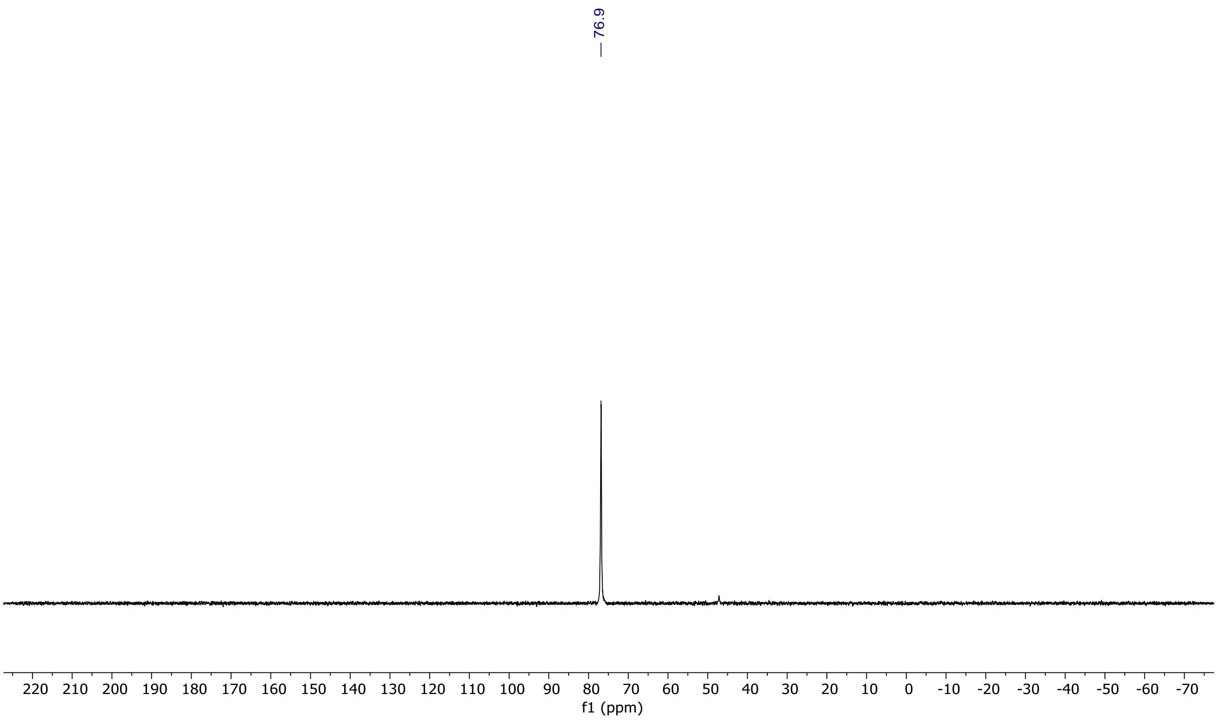

Figure S141. $^{31}P\{^{1}H\}$ NMR (101 MHz, CD_2Cl_2) of *fac*-[Mn($P^{Cy}P^{Cy}$)$(CO)_3$OTf] (**2j**)

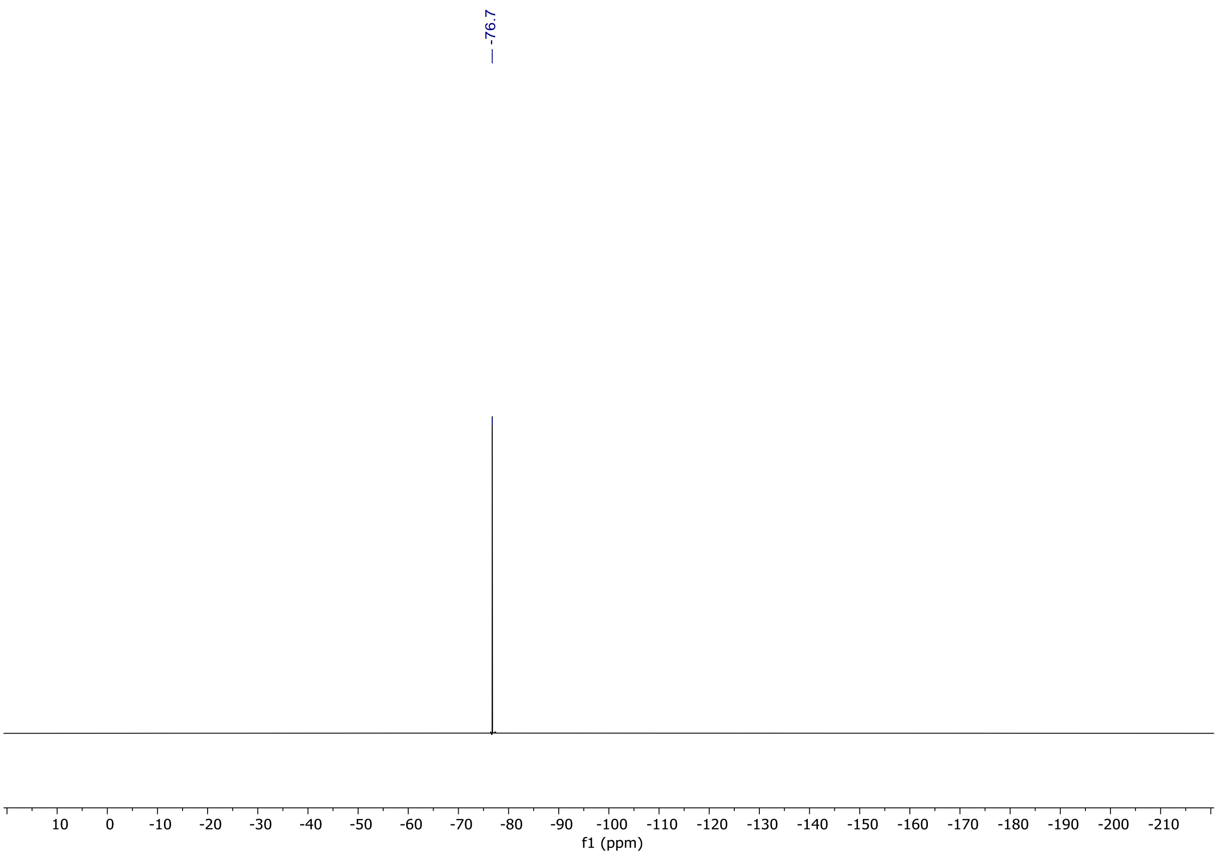

Figure S142. ^{19}F NMR (235 MHz, CD_2Cl_2) of *fac*-[Mn($P^{Cy}P^{Cy}$)$(CO)_3$OTf] (**2j**)

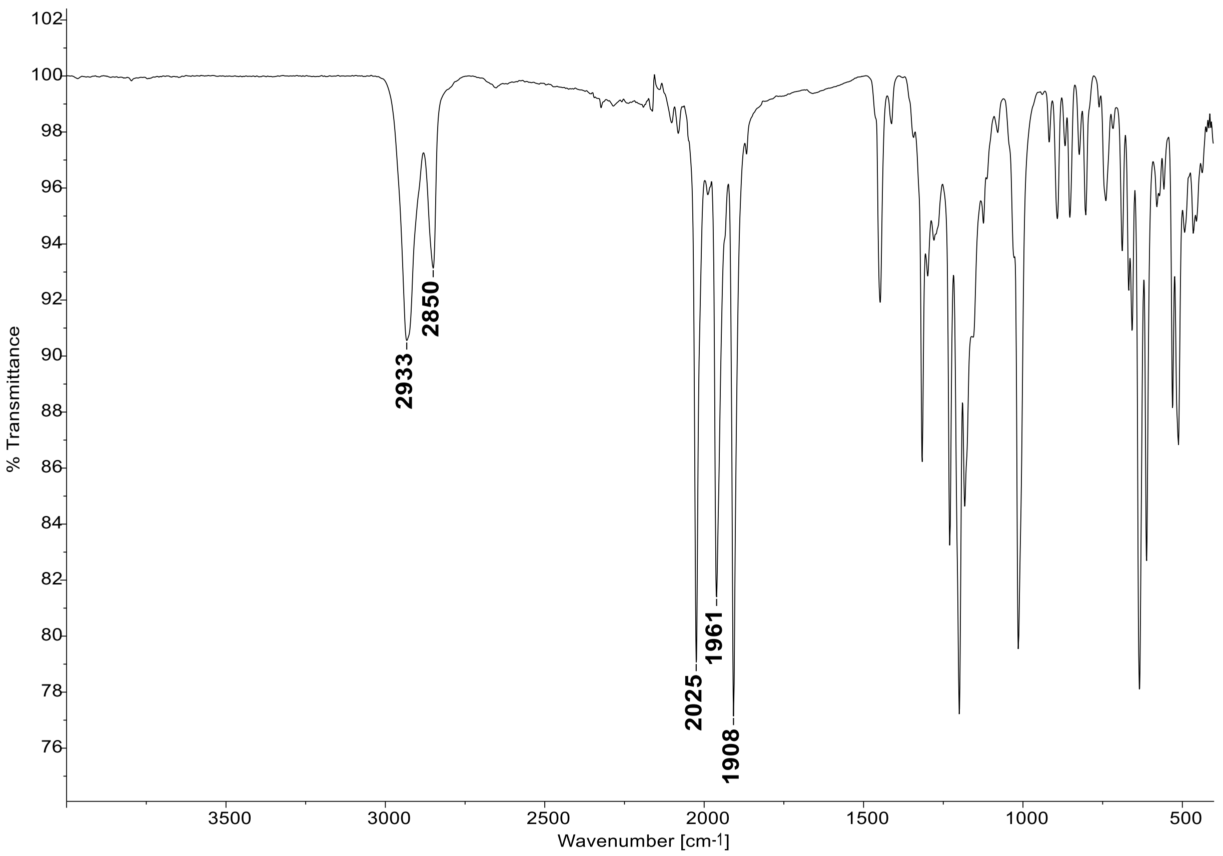

Figure S143. IR (ATR, cm^{-1}) of *fac*-[Mn($P^{Cy}P^{Cy}$)$(CO)_3$OTf] (**2j**)

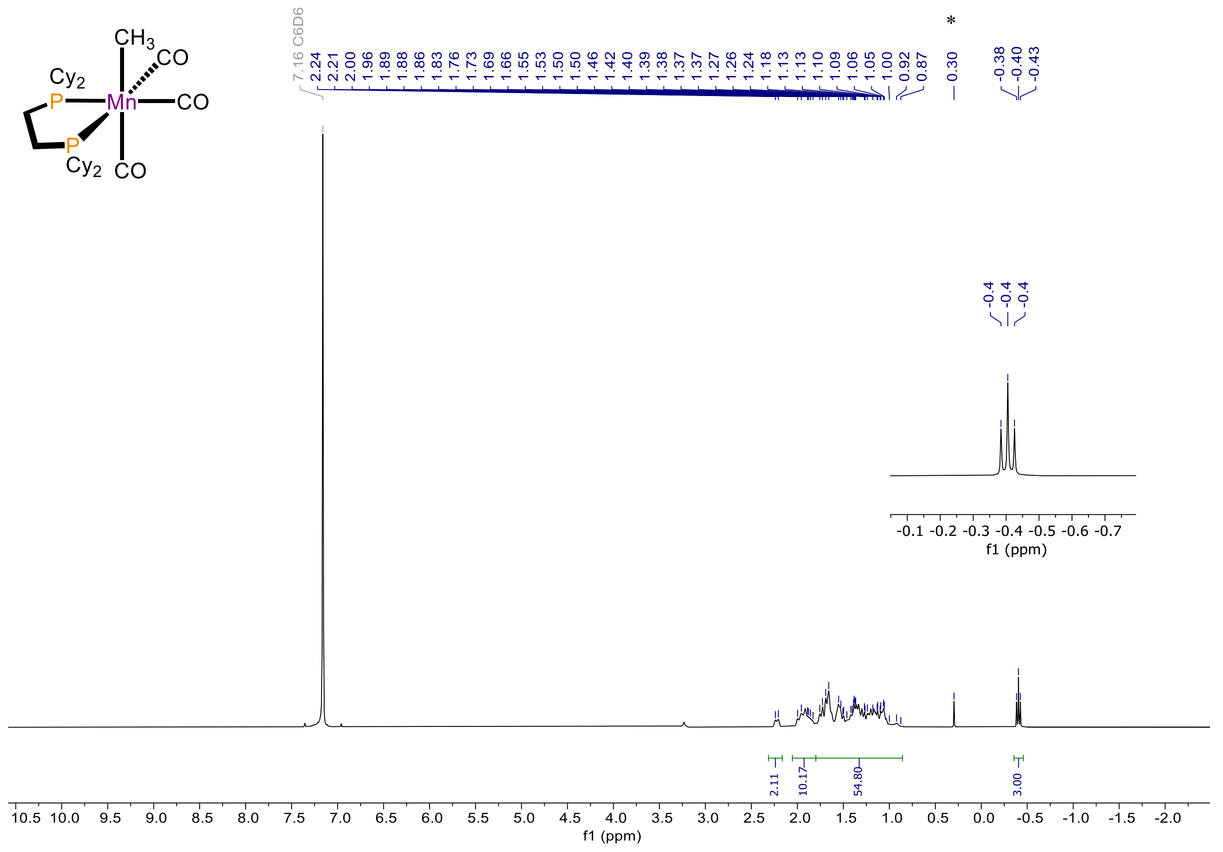

* = grease

Figure S144. ^{1}H NMR (400 MHz, C_6D_6) of *fac*-[Mn($P^{Cy}P^{Cy}$)$(CO)_3$Me] (**PP4**)

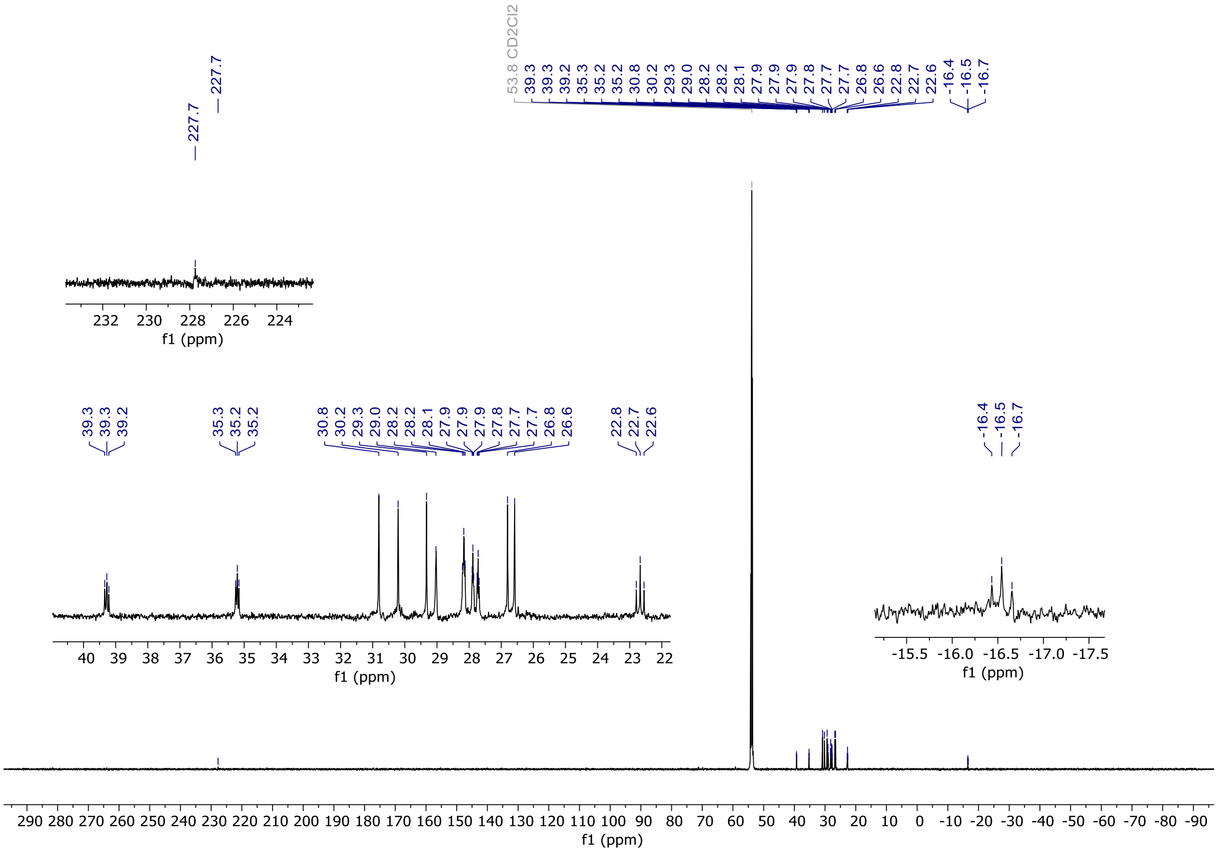

Figure S145. $^{13}C\{^{1}H\}$ NMR (151 MHz, CD_2Cl_2) of *fac*-[Mn($P^{Cy}P^{Cy}$)(CO)$_3$Me] (**PP4**)

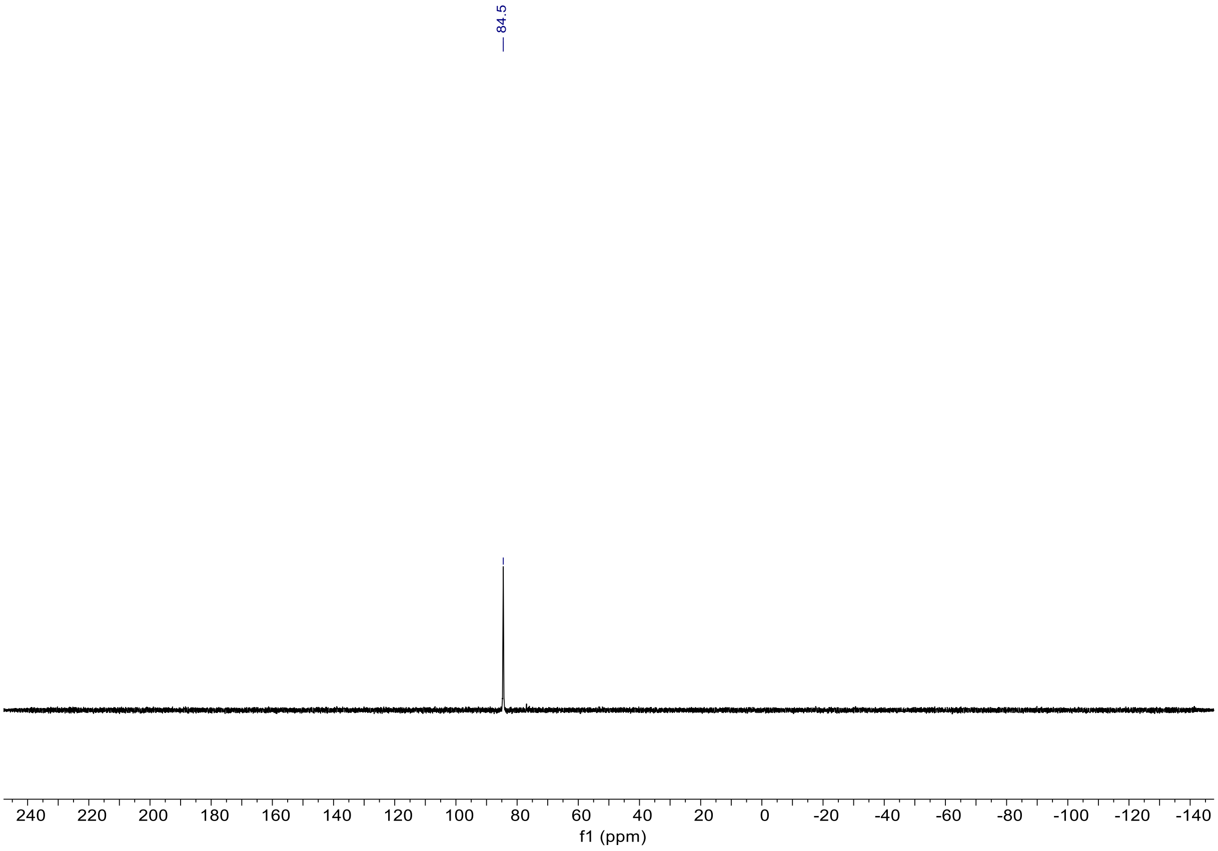

Figure S146. $^{31}P\{^{1}H\}$ NMR (162 MHz, CD_2Cl_2) of *fac*-[Mn($P^{Cy}P^{Cy}$)(CO)$_3$Me] (**PP4**)

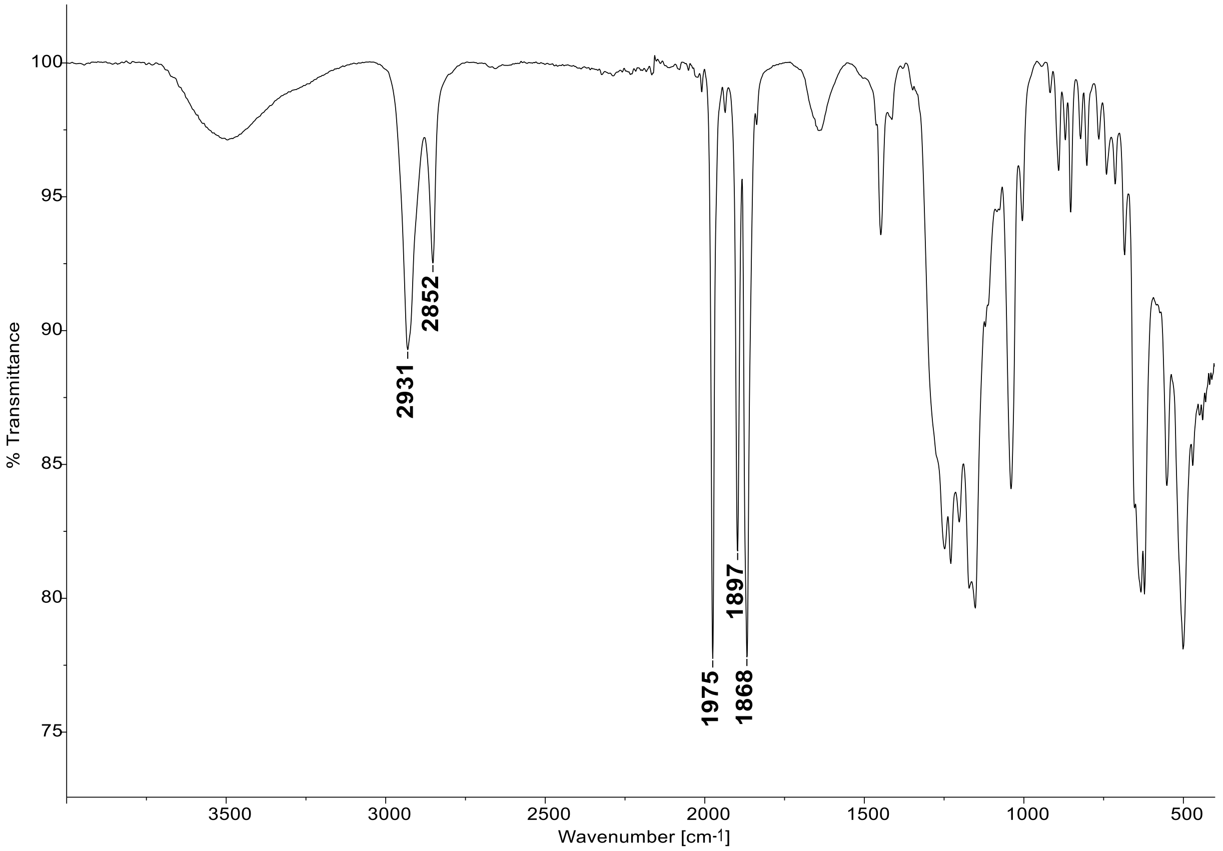

Figure S147. IR (ATR, cm^{-1}) of *fac*-[Mn($P^{Cy}P^{Cy}$)$(CO)_3$Me] (**PP4**)

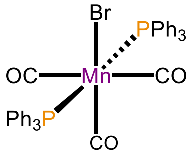

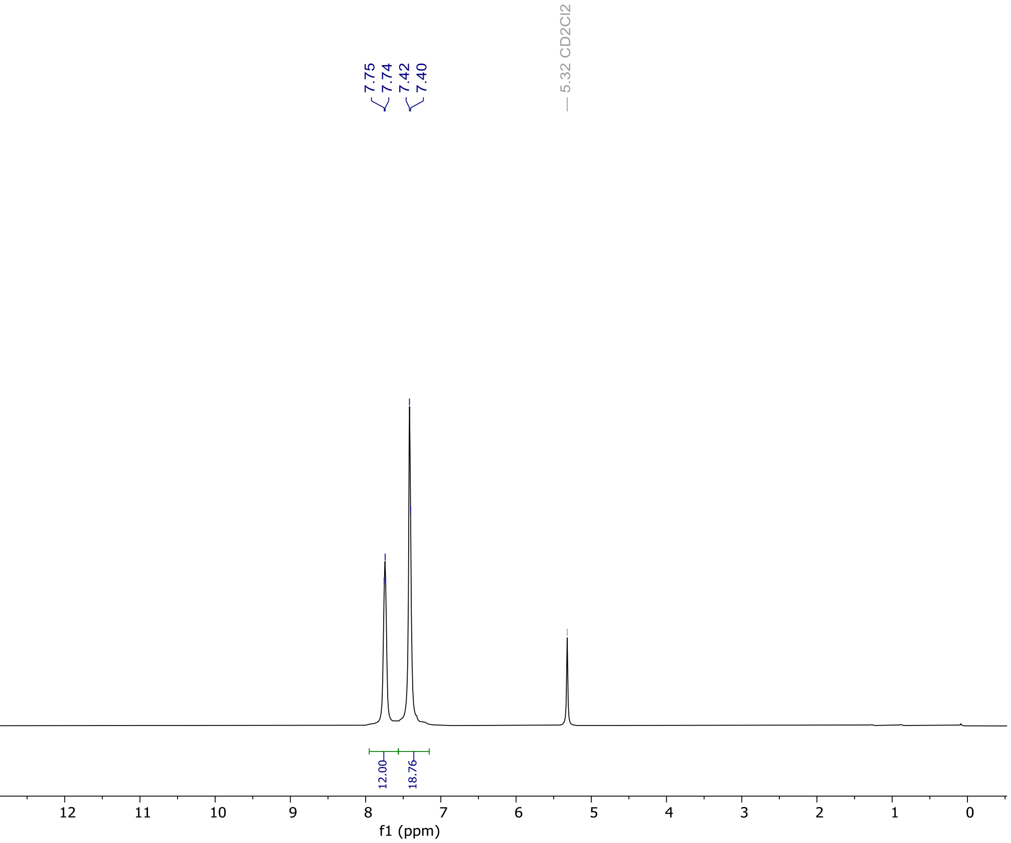

Figure S148. ^{1}H NMR (400 MHz, CD_2Cl_2) of *trans*-[Mn$(PPh_3)_2(CO)_3$Br] (**1k**)

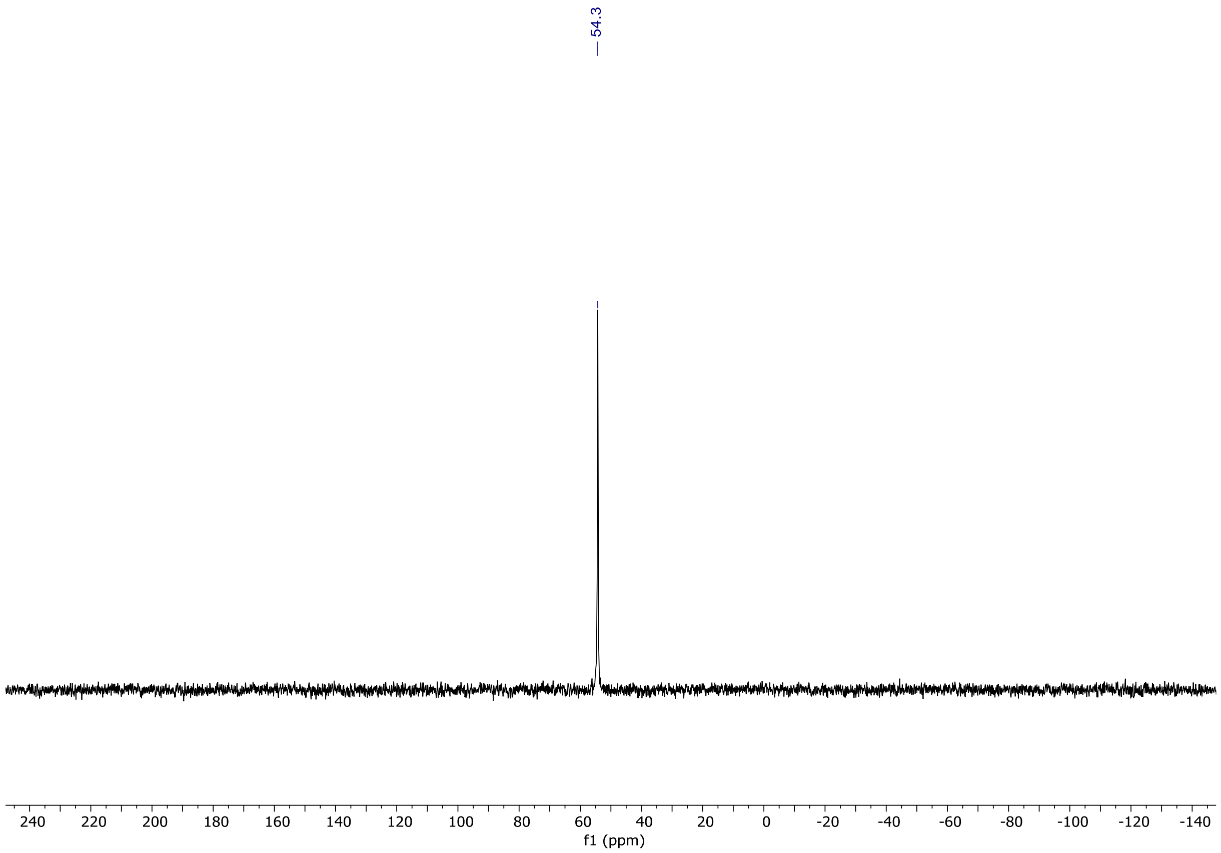

Figure S149. $^{31}P\{^1H\}$ NMR (162 MHz, CD_2Cl_2) of *trans*-[Mn(PPh_3)$_2$(CO)$_3$Br] (**1k**)

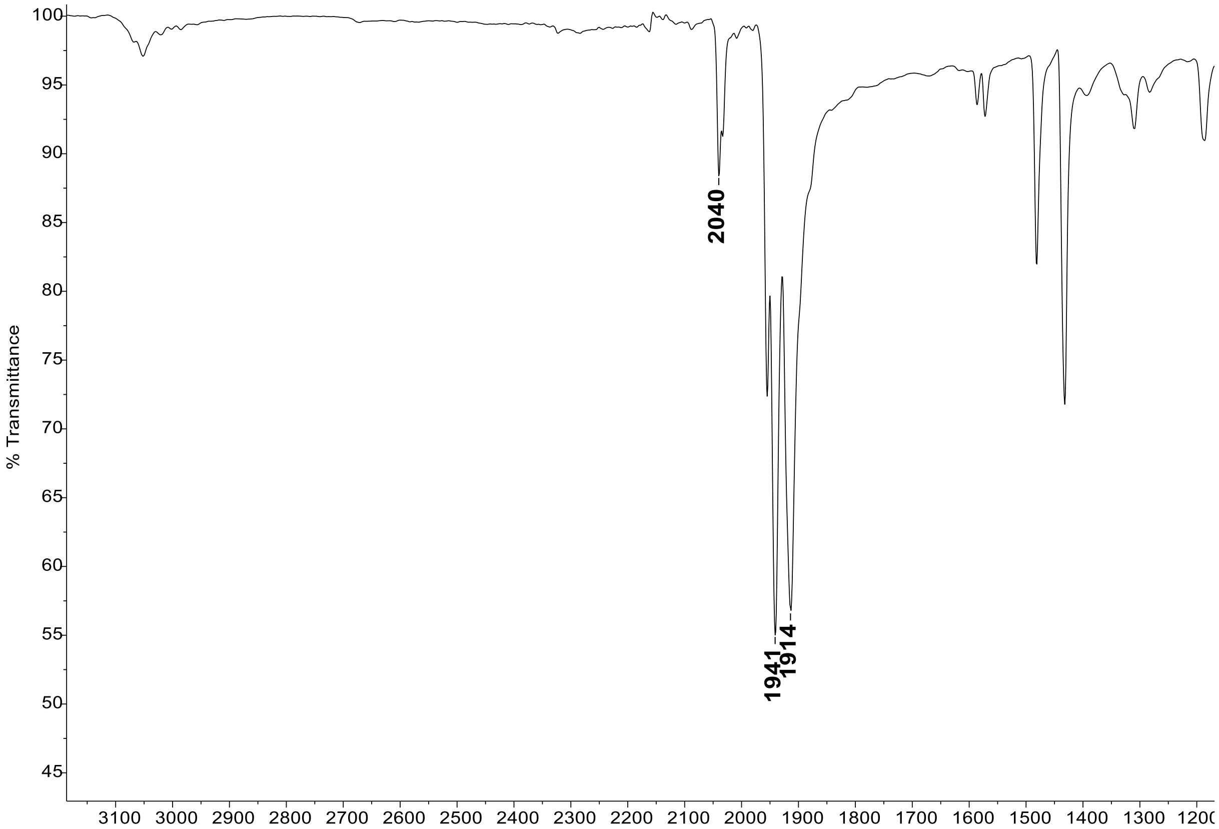

Figure S150. IR (ATR, cm^{-1}) of *trans*-[Mn(PPh_3)$_2$(CO)$_3$Br] (**1k**)

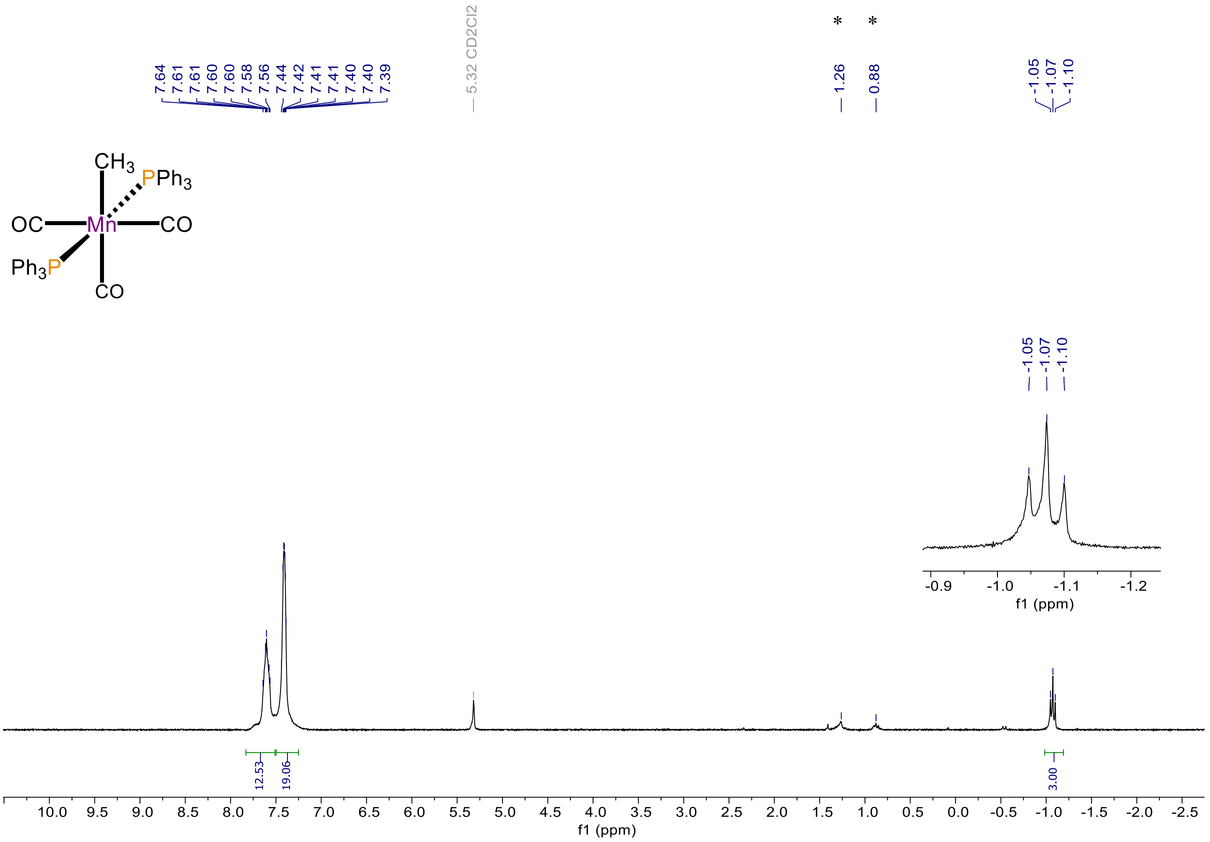

* = grease

Figure S151. ^{1}H NMR (250 MHz, CD_2Cl_2) of *trans*-[$Mn(PPh_3)_2(CO)_3Me$] (**PP5**)

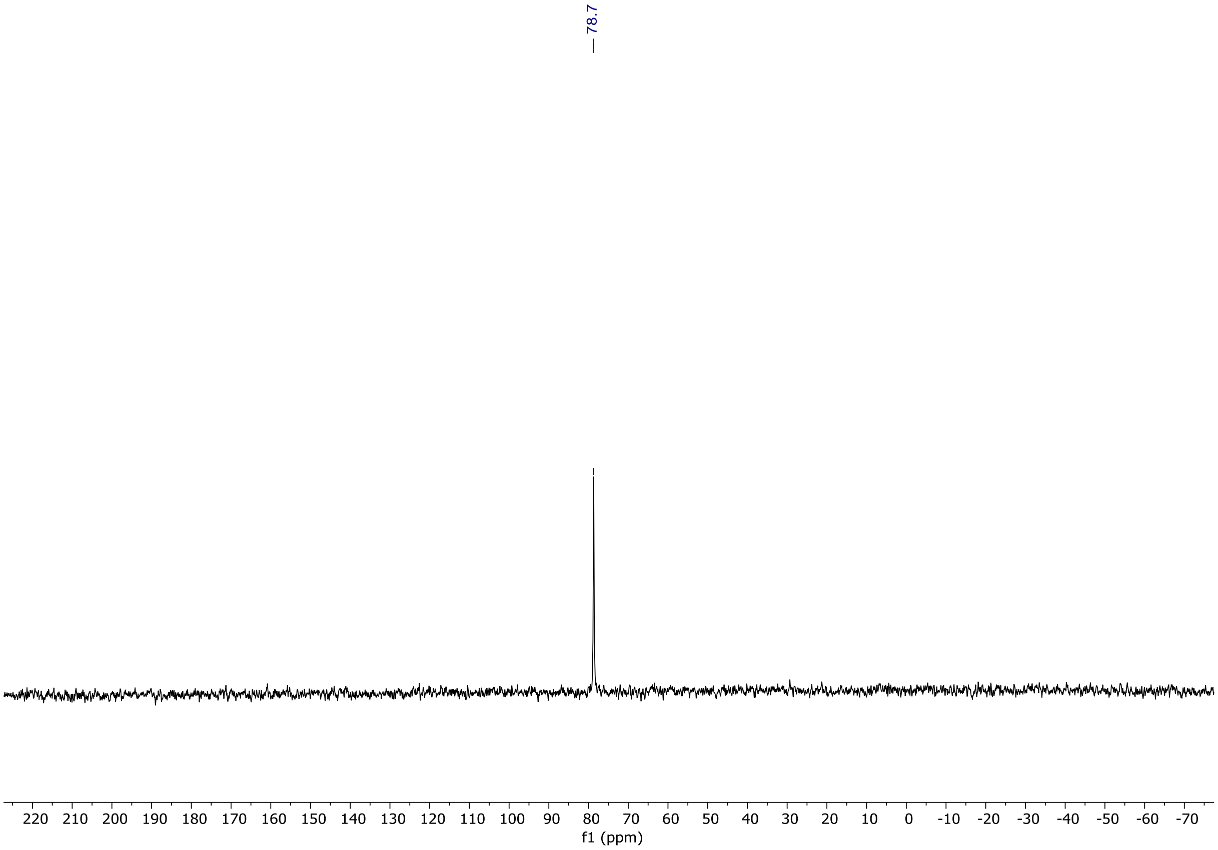

Figure S 152. ^{31}P{^{1}H} NMR (101 MHz, CD_2Cl_2) of *trans*-[$Mn(PPh_3)_2(CO)_3Me$] (**PP5**)

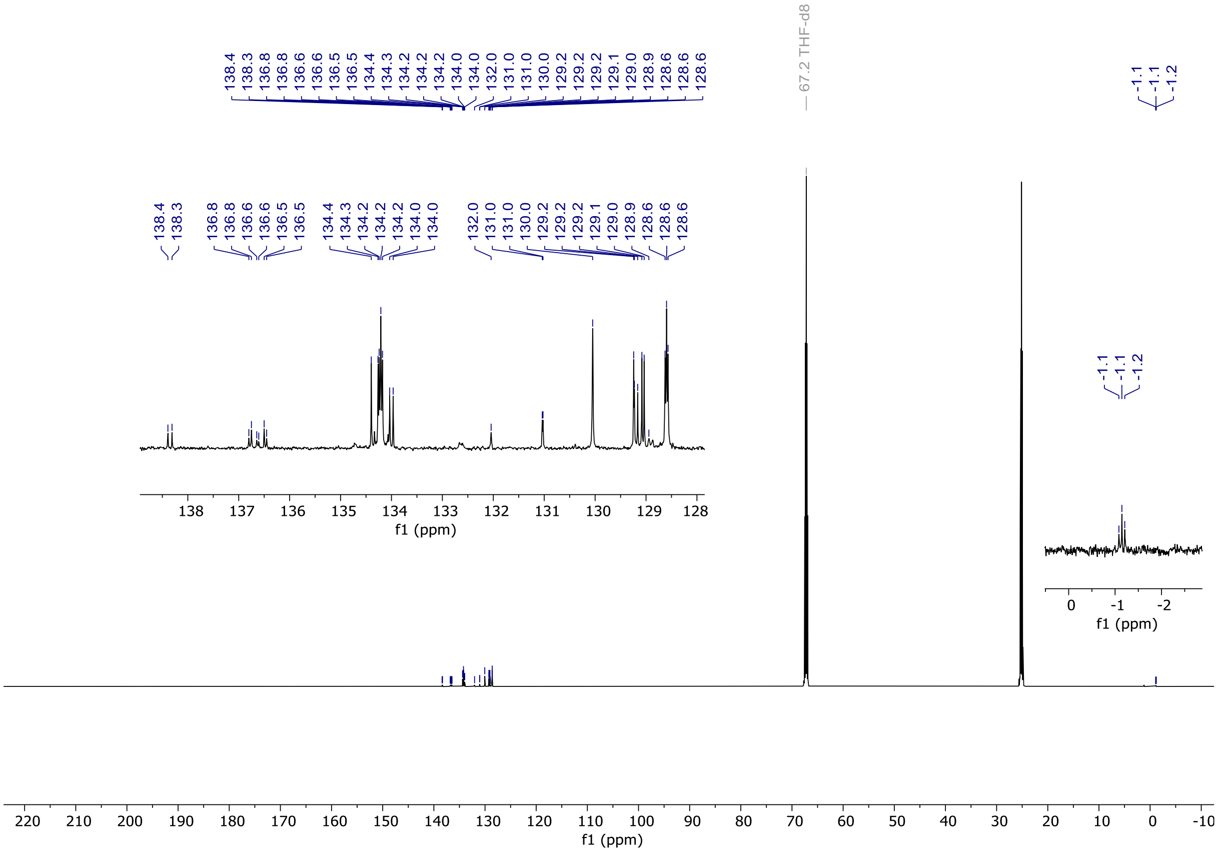

Figure S153. $^{13}C\{^{1}H\}$ NMR (151 MHz, THF-d_8) of *trans*-[Mn(PPh$_3$)$_2$(CO)$_3$Me] (**PP5**)

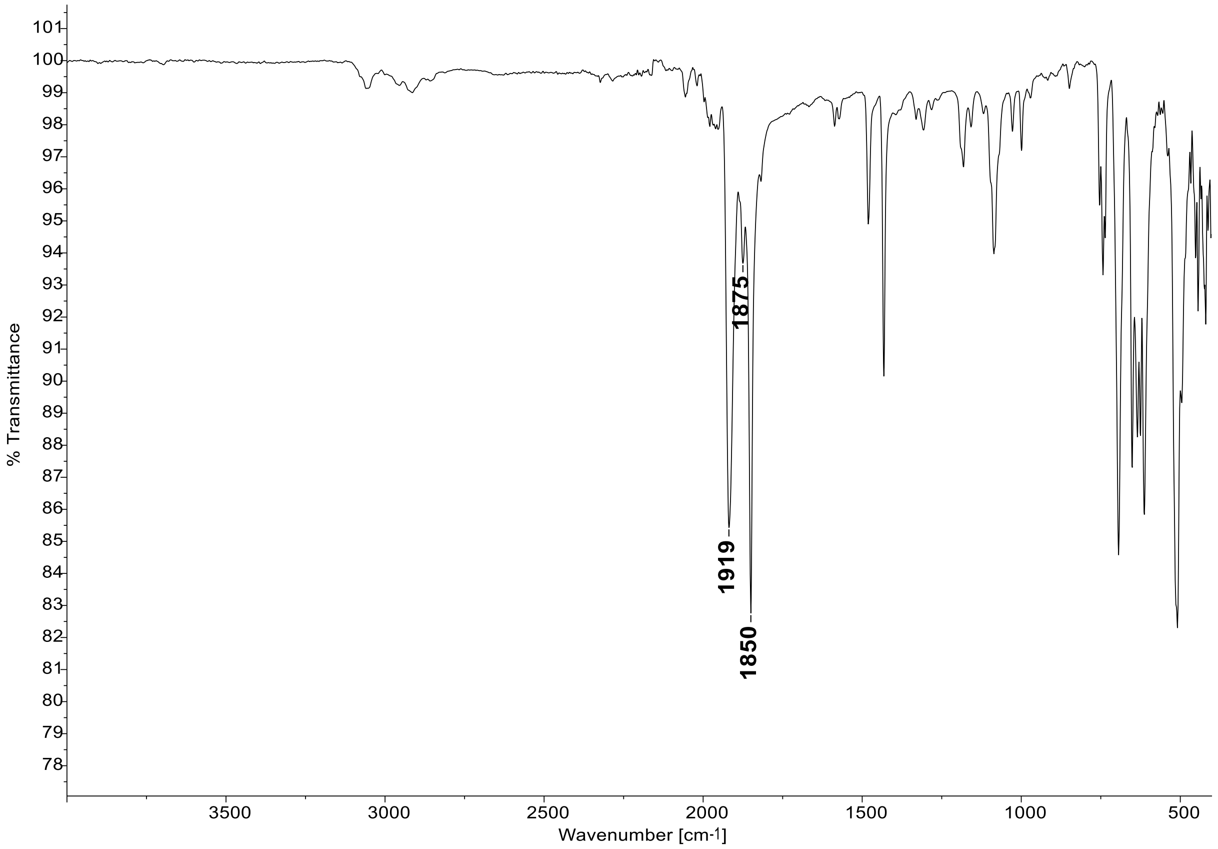

Figure S154. IR (ATR, cm^{-1}) of *trans*-[Mn(PPh$_3$)$_2$(CO)$_3$Me] (**PP5**)

11 Crystallographic Data

Crystals **1a**[12], **1b**, **1i** and **2g** suitable for X-Ray analysis were obtained by storing a saturated *n*-pentane solution at 5 °C. **PN1** was crystalized by storing a saturated *n*-pentane solution inside the glove box at -30 °C.

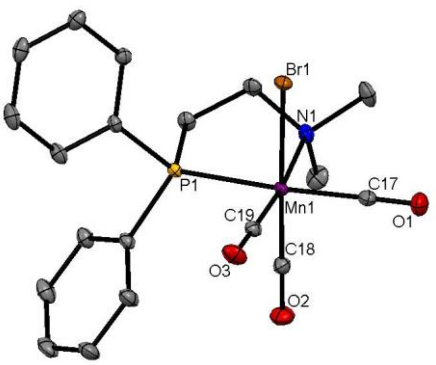

Figure S155. *fac*-[Mn($P^{Ph}N^{Me}$)$(CO)_3$Br] **(1a)**

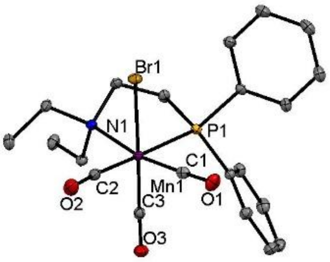

Figure S156. *fac*-[Mn($P^{Ph}N^{Et}$)$(CO)_3$Br] **(1b)**

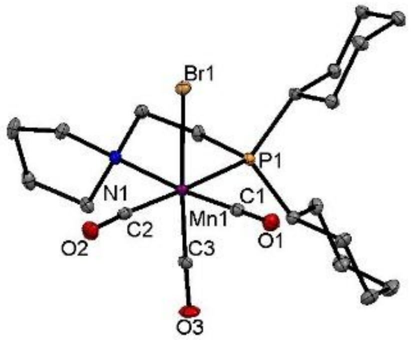

Figure S 157. *fac*-[Mn($P^{Cy}N^{Pyr}$)$(CO)_3$Br] **(1i)**

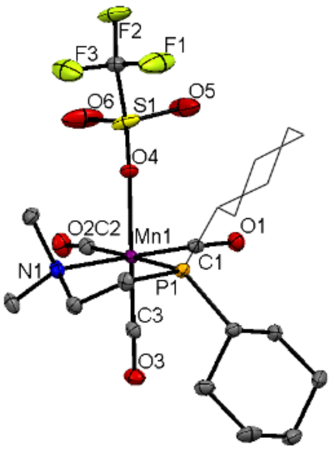

Figure S 158. *fac*-[Mn($P^{Cy}N^{Me}$)$(CO)_3$OTf] **(2g)**

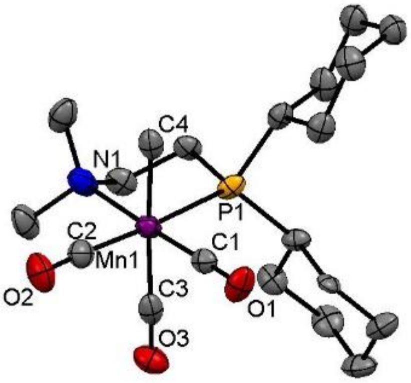

Figure S 159. *fac*-[Mn($P^{Cy}N^{Me}$)$(CO)_3$Me] **(PN1)**

Table 5. Crystallographic Data of 1a, 1b, 1i, 2g, PN1

	1a[a]	1b	1i	2g	PN1
Formula	$C_{19}H_{20}BrMnNO_3P$	$C_{21}H_{24}BrMnNO_3P$	$C_{21}H_{34}BrMnNO_3P$	$C_{20}H_{32}F_3MnNO_6PS$	$C_{20}H_{35}MnNO_3P$
**CCDC**	**2423049**	**2423050**	**2423051**	**2423052**	**2423053**
T [K]	100	100	100	100	100
Crystal System	Monoclinic	Monoclinic	Triclinic	Monoclinic	Triclinic
Space group	C2	P 2_1/n	P-1	P 2_1/n	P-1
a, Å	17.732(2)	9.7614(11)	7.3777(5)	9.1485(15)	7.4269(12)
b, Å	7.8691(8)	14.6455(16)	11.8631(7)	14.862(3)	11.3184(19)
c, Å	14.0687(14)	14.8093(16)	13.5418(8)	18.723(3)	13.1922(21)
α, °	90	90	95.657(2)	90	105.498(5)
β, °	90.140(4)	102.875(2)	101.725(2)	101.783(5)	93.094(5)
γ, °	90	90	106.338(2)	90	94.466(5)
Volume, Å^3	1963.0(4)	2063.9(4)	1098.21(12)	2492.0(7)	1062.1(3)
Z	4	4	2	4	2
ρ_{calc} [g/cm^3]	1.611	1.623	1.555	1.486	1.324
μ (mm-1)	2.807	2.675	2.515	0.735	0.715
F (000)	960.0	1024.0	532.0	1160.0	0.715
Radiation	MoKα (λ = 0.71073)	MoKα (λ = 0.71073)	MoKα (λ = 0.71073)	MoKα (λ = 0.71073)	MoKα (λ = 0.71073)
Reflections collected	8752	10308	6509	9995	6163
Unique reflections	8365	8519	5295	7624	3949
R(int)	0.0181	0.0297	0.0280	0.0385	0.0527
R1 (I > 2 σ(I))	0.0204	0.0431	0.0433	0.0600	0.1012
wR_2	0.0432	0.0620	0.0610	0.1061	0.1235
GOOF	0.967	1.024	1.029	0.878	1.026

[a]The structure was already published in the *Journal of Chemical Crystallography* in 2004 (CCDC 235489).[12]

Table 6. Selected Bond Distances (Å) and Angles (°) of 1a, 1b, 1i, 2g and PN1.

1a		1b		1i	
Mn1-Br1	2.5271(4)	Mn1-Br1	2.5291(5)	Mn1-Br1	2.5425(3)
Mn1-P1	2.3214(5)	Mn1-P1	2.3230(5)	Mn1-P1	2.3426(5)
Mn1-N1	2.204(1)	Mn1-N1	2.239(1)	Mn1-N1	2.186(2)
Mn1-C17	1.847(2)	Mn1-C3	1.796(1)	Mn1-C1	1.7972(1)
Mn1-C18	1.791(2)	Mn1-C1	1.791(1)	Mn1-C2	1.8414(1)
Mn1-C19	1.793(2)	Mn1-C2	1.831(1)	Mn1-C3	1.774(2)
C17-O1	1.142(2)	C3-O3	1.149(1)	C1-O1	1.150(2)
C18-O2	1.151(2)	C1-O1	1.152(2)	C2-O2	1.141(2)
C19-O3	1.148(2)	C2-O2	1.146(1)	C3-O3	1.189(2)
P1-Mn1-N1	83.54(4)	P1-Mn1-N1	82.17(3)	P1-Mn1-N1	84.26
P1-Mn1-C17	176.28(5)	P1-Mn1-C2	176.03(4)	P1-Mn1-C2	175.21(5)
Br1-Mn1-C18	177.13(5)	Br1-Mn1-C3	174.50(4)	Br1-Mn1-C3	176.45(7)
N1-Mn1-C19	174.31(6)	N1-Mn1-C1	169.59(5)	N1-Mn1-C1	174.51(6)
**2g**		**PN1**			
Mn1-O4	2.088(1)	Mn1-C4	2.065(9)		
Mn1-P1	2.342(8)	Mn1-P1	2.3162(8)		
Mn1-N1	2.189(1)	Mn1-N1	2.210(2)		
Mn1-C1	1.811(2)	Mn1-C2	1.801(3)		
Mn1-C2	1.849(2)	Mn1-C3	1.881(9)		
Mn1-C3	1.780(2)	Mn1-C1	1.767(2)		
C1-O1	1.146(2)	C1-O1	1.1539(1)		

C2-O2	1.144(2)	C3-O2	1.0297(1)
C3-O3	1.158(2)	C2-O3	1.1421(1)
P1-Mn1-N1	83.84(4)	P1-Mn1-N1	83.65(7)
P1-Mn1-C2	176.7(5)	P1-Mn1-C2	176.4(1)
O4-Mn1-C3	178.34(6)	C3-Mn1-C4	171.4(4)
N1-Mn1-C1	176.14(6)	N1-Mn1-C1	177.3(1)

12 References

(1) Perin, D. D. ; A. W. L. F. Purification of Labratory Chemicals, 3rd Ed.; *Pergamon: New York* **1998**

(2) Bruker computer programs: APEX3, SAINT and SADABS; Bruker AXS Inc., Madison, WI, 2020.

(3) Sheldrick, G. M. Crystal structure refinement with SHELXL. *Acta Crystallogr.* **2015**, A71, 3−8.

(4) Sheldrick, G. M. Crystal structure refinement with SHELXL. *Acta Crystallogr.* **2015**, C71, 3−8.

(5) C. F. Macrae, P. R. Edgington, P. McCabe, E. Pidcock, G. P. Shields, R. Taylor, M. Towler and J. van de Streek. *J. Appl. Cryst.* **2006**, *39*, 453–457.

(6) Jiménez, M. V.; Pérez-Torrente, J. J.; Bartolomé, M. I.; Oro, L. A. Convenient Methods for the Synthesis of a Library of Hemilabile Phosphines. *Synthesis (Stuttg)* **2009**, 1916–1922.

(7) E. McEwen, W.; H. Smith, J.; J. Woo, E. Role of through Space 2p-3d Overlap in the Alkylation of (Omega.-N,N-Dimethylaminoalkyl)Diphenylphosphines and in the Alkaline Decomposition of Related Quaternary Phosphonium Salts. *J. Am. Chem. Soc.* **2002**, *102*, 2746–2751.

(8) K. Anderson, G.; Kumar, R. Platinum(II) Complexes of Unsymmetrical, Potentially Bidentate Ligands. *Inorg. Chem.* **2002**, *23*, 4064–4068.

(9) Stoppioni, P.; Vaira, M. Di; Maitlis, P. M. Pentamethylcyclopentadienylrhodium Complexes with Tripod Tetradentate Ligands and Bidentate 'Mixed' Ligands. *J. Chem. Soc., Dalton Trans.* **1982**, 1147–1154.

(10) Arakawa, M.; Suzuki, N.; Kishi, S.; Hasegawa, M.; Satoh, K.; Horn, E.; Fukuda, Y. Synthesis, Crystal Structure, and Chromotropic Properties of Mixed-Ligand Nickel(II) Complexes with 1,3-Diketonate and P-N Bidentate Ligands. *Bull. Chem. Soc. Jpn* **2008**, *81*, 127–135.

(11) Werner, H.; Hampp, A.; Peters, K.; Peters, E. M.; Walz, L.; Schnering, H. G. von. Synthese, Struktur Und Reaktivität Labiler Rhodium(I)-Komplexe Mit IPr2PC2H4NMe2 Und IPr2PC2H4OMe Als Phosphanliganden / Synthesis, Structure and Reactivity of Labile Rhodium(I) Complexes Containing IPr2PC2H4NMe2 and IPr2PC2H4OMe as Phosphine Ligands. *Zeitschrift für Naturforschung B* **1990**, *45*, 1548–1558.

(12) Bauer, J. A. K.; Becker, T. M.; Orchin, M. The Preparation and Crystal Structures of Some Tricarbonylmanganese(I) Octahedral Complexes Containing the 1,1-Dimethylamino-2,2-Diphenylphosphinoethane Ligand. *J. Chem. Crystallogr.* **2004**, *34*, 843–849.

(13) Burt, R. J.; Chatt, J.; Hussain, W.; Leigh, G. J. A Convenient Synthesis of 1,2-Bis(Dichlorophosphino)Ethane, 1,2-Bis(Dimethylphosphino)Ethane and 1,2-Bis(Diethylphosphino)Ethane. *J. Organomet. Chem.* **1979**, *182*, 203–206.

(14) Garduño, J. A.; Flores-Alamo, M.; García, J. J. Manganese-Catalyzed Transfer Hydrogenation of Nitriles with 2-Butanol as the Hydrogen Source. *ChemCatChem* **2019**, *11*, 5330–5338.

(15) Angelici, R. J.; Basolo, F.; Poe, A. J. Metal Carbonyls. VI. Isomerism of Disubstituted Manganese Pentacarbonyl Bromide. *J. Am. Chem. Soc.***1963**, *85*, 2215–2219.

(16) Abel, E. W.; Wilkinson, G. Carbonyl Halides of Manganese and Some Related Compounds. *J. Chem. Soc.* **1959**, 1501-1505.

(17) Hieber, W.; Hoefler, M.; Muschi, J. Metal Carbonyls. CXLII. Bisphosphine-Substituted Carbonylmanganates(-I) and Their Derivatives. *Chem. Ber.* **1965**, *98*, 311–320.
